# Design, synthesis and in vitro anticancer activity of some new lomefloxacin derivatives

**DOI:** 10.1038/s41598-024-56313-w

**Published:** 2024-03-14

**Authors:** Mina E. Adly, Ehab M. Gedawy, Afaf A. El-Malah, Omneya M. Khalil

**Affiliations:** 1 Pharmaceutical Organic Chemistry Department, Faculty of Pharmacy, Cairo University, 33 Kasr El-Aini Street, Cairo, 11562 Egypt; 2 Pharmaceutical Chemistry Department, Faculty of Pharmacy, Badr University in Cairo, Cairo, 11829 Egypt

**Keywords:** Lomefloxacin, Anticancer activity, Topoisomerase II, Medicinal chemistry, Organic chemistry, Chemical synthesis

## Abstract

Our main goal was to design and synthesize novel lomefloxacin derivatives that inhibit the topoisomerase II enzyme, leading to potent anticancer activity. Lomefloxacin derivatives substituted at position 3 and 7 were synthesized and screened for cytotoxic activity utilizing 60 different human cancer cell lines. Furthermore, compounds **3a,b,c,e** that revealed potent broad-spectrum anticancer activity (with mean percent GI more than 47%) were further evaluated using five dose concentrations and calculating the GI_50_. Compound **3e** was then evaluated for cell cycle analysis and demonstrated cell cycle arrest at the G2-M phase. Moreover, the mechanism of action was determined by determining the topoisomerase inhibitory activity and the molecular modeling study. Compounds **3a,b,c,e** showed broad spectrum anticancer activity. Lomefloxacin derivative **5f** showed selective cytotoxic activity against melanoma SK-MEL-5 cell line. Compound **3e** demonstrated comparable topoisomerase II inhibition to doxorubicin with IC_50_ of 0.98 µM.

## Introduction

Lomefloxacin, a second generation fluroquinolone is known to possess potent antibacterial activity^1^. Several antibacterial fluoroquinolones have shown over the years to be cytotoxic to cancer cells thus representing a new interesting class of anticancer agents^2–4^. Since cancer patients treated with traditional anticancer agents are often immunodeficient, developing compounds with anticancer activity as well as antimicrobial activity may prove to be useful^5^. Lomefloxacin has proven to induce apoptosis in leukemia^6^ and melanoma cell lines^7^. Moreover, the anticancer activity of fluoroquinolones originates from their abilities to powerfully inhibit topoisomerase II^8^. Topoisomerase II enzyme is a nuclear enzyme that solve DNA topological problems that arise during DNA replication. Hence, inhibition of Topo II enzyme results in cell death and apoptosis^9^.

**Figure 1:**
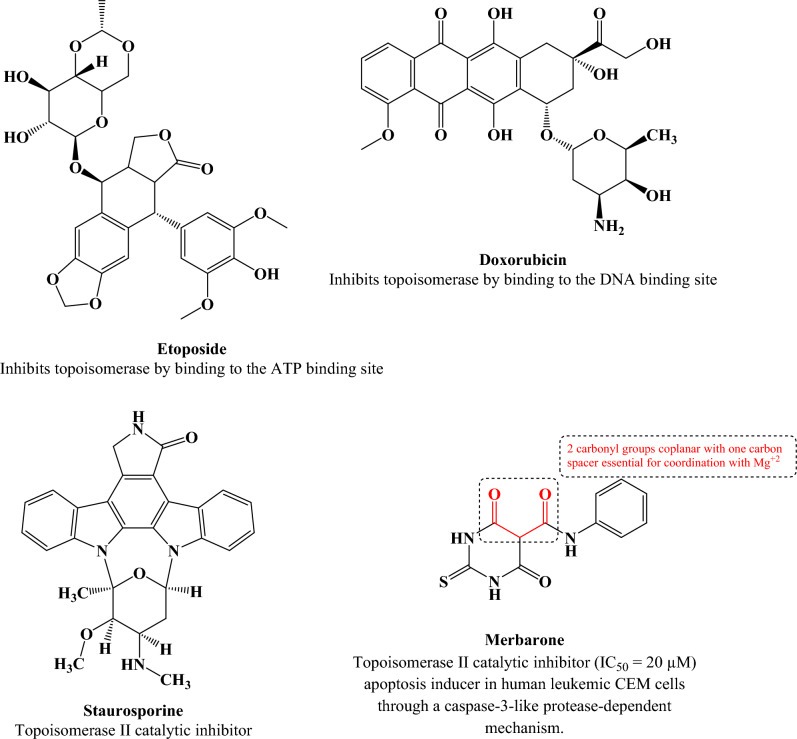
Known inhibitors of topoisomerase II. Generated using ChemDraw 2021 (https://perkinelmerinformatics.com/products/research/chemdraw).

A new class of topoisomerase II inhibitors known as the catalytic inhibitors such as merbarone and staurosporine (Fig. 1) that act by inhibiting of topoisomerase II-mediated DNA cleavage^10–12^. Moreover, merbarone acts via chelation of magnesium which is important for the catalysis of the topoisomerase II enzyme^13^.

**Figure 2:**
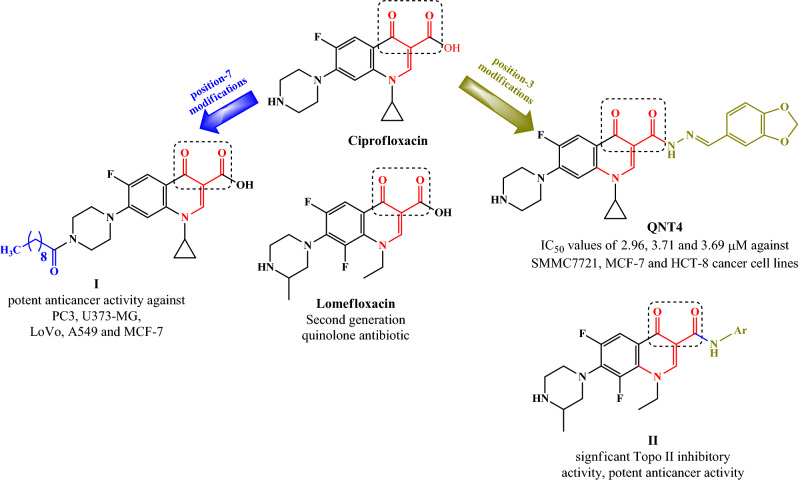
structure modifications of different fluoroquinolones. Generated using ChemDraw 2021 (https://perkinelmerinformatics.com/products/research/chemdraw).

Several structural modifications were reported for different fluoroquinolones to optimize their anticancer activity, such modifications were focused on position-7 and position-3 of the quinolone nucleus. In the last decade various 7-substituted ciprofloxacin derivatives (**I**) (Fig. 2) were reported to exhibit potent anticancer activity via their inhibitory activity against human topoisomerase enzymes^14,15^. Regarding position-3 modification, several fluoroquinolone derivatives were reported to possess potent anticancer activity such as, ciprofloxacin hydrazone derivative (QNT4), was reported to induce cell apoptosis in human hepatocarcinoma cells by inhibiting topoisomerase II activity^16^. Additionally, different amide derivatives of lomefloxacin (**II**) (Fig. 2) revealed significant inhibitory activity against topoisomerase II enzymes at 100 µM and remarkable cytotoxic activity with IC_50_ ranging between 1.60 and 6.90 µM against several cell lines^17^.

**Figure 3:**
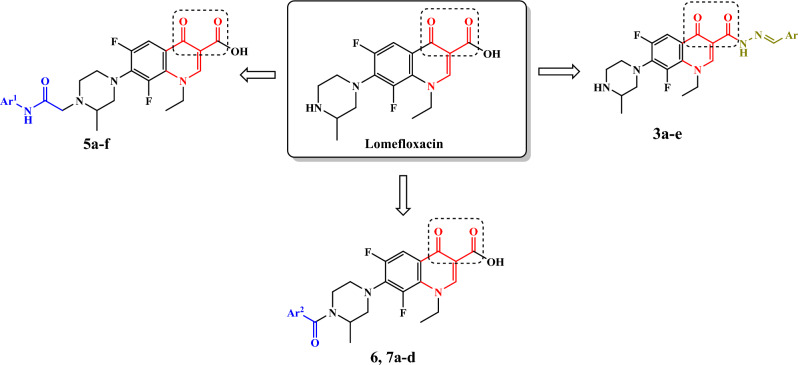
Design strategies for the target compounds. Generated using ChemDraw 2021 (https://perkinelmerinformatics.com/products/research/chemdraw).

In this study, modifications were made at position 3 and 7 of lomefloxacin while retaining the dicarbonyl system that seems to be essential for the topoisomerase II inhibitory activity. To modify position 3, lomefloxacin (**1**) (Fig. 3) was reacted with hydrazine hydrate to afford compound **2** followed by reaction with several aldehydes. Modifications of position-7 were done through alkylation or acylation of N-4 of piperazine moiety with several chloroacetanilides, formamide and acid chlorides.

## Results and discussion

### Chemistry

**Figure 4:**
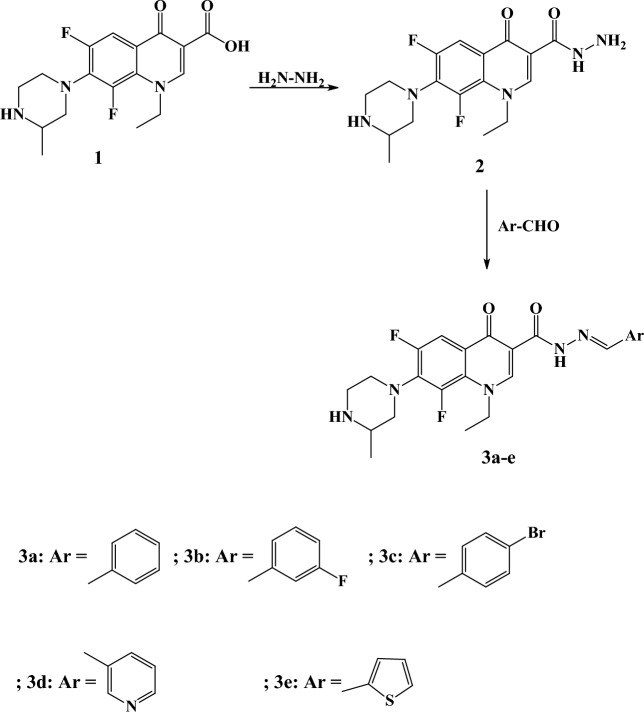
Synthesis of compounds 3a-e. Generated using ChemDraw 2021 (https://perkinelmerinformatics.com/products/research/chemdraw).

**Figure 5:**
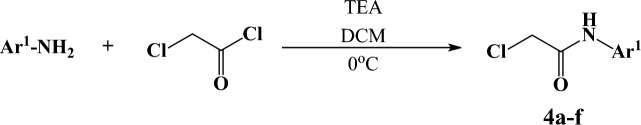
Synthesis of intermediate compounds 4a-f. Generated using ChemDraw 2021 (https://perkinelmerinformatics.com/products/research/chemdraw).

**Figure 6:**
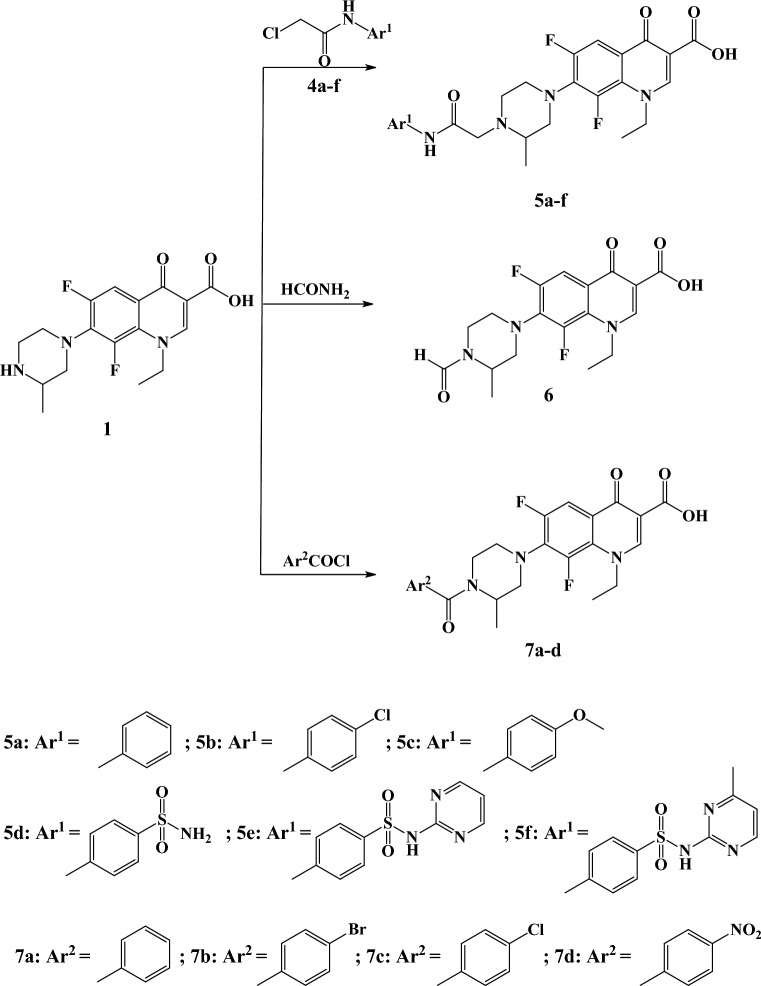
Synthesis of compounds **5a-f**, **6**, **7a-d**. Generated using ChemDraw 2021 (https://perkinelmerinformatics.com/products/research/chemdraw).

**Figure 7:**
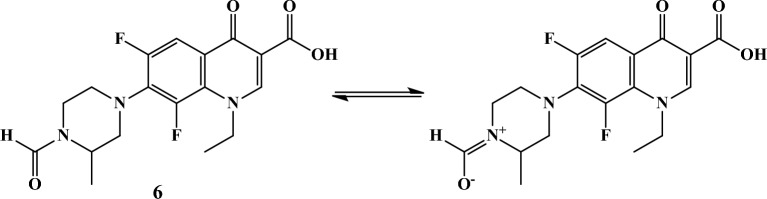
Tautomerism for compound **6**, Generated using ChemDraw 2021 (https://perkinelmerinformatics.com/products/research/chemdraw).

An outline for the synthesis of the target compounds is shown in Figs. 4, 5, 6. Lomefloxacin **1** was subjected to direct hydrazinolysis to yield lomefloxacin hydrazide **2**. Condensation of **2** with several aldehydes afforded compounds **3a**–**e**. ^1^H NMR spectra for compounds **3a**–**e** demonstrated disappearance of NH_2_ and appearance of NH signal at δ 13.29–13.45 ppm as well as OH signal at δ 2.20–7.71 ppm due to presence of these compounds as two tautomers (enol amide and keto amide). Moreover, N=CH proton appeared as two singlet peaks at δ 8.31–8.72 ppm. ^13^C NMR showed an additional aliphatic carbon atom due to the presence of a chiral carbon at 16.50–19.40 ppm. Chloroacetanilides **4a**–**f** were synthesized according to the previously reported procedure^18,19^. Target compounds **5a**–**f** were synthesized by reacting the 2-chloro-N-phenylacetamides **4a**–**f** with lomefloxacin in presence of triethylamine. ^1^H NMR showed the appearance of a singlet peak at δ 3.31–4.71 ppm corresponding to the CH_2_ of 2-chloro-*N*-phenylacetamide group. Moreover, a D_2_O exchangeable peak appeared at δ 8.93–10.09 ppm corresponding to the NH group. Formylation of lomefloxacin with formamide yielded compound **6**. ^1^H NMR showed the disappearance of NH proton and the appearance of the CH of the formyl group at δ 8.05 and 8.16 ppm as two singlet peaks due to the presence of tautomerism as shown in Fig. 7. Reaction of lomefloxacin with several benzoyl chlorides in presence of triethyl amine yielded compounds **7a-d**. ^1^H NMR spectra for compounds **7a**–**d** demonstrated the disappearance of the NH peak as well as the appearance of the aromatic protons for the benzoyl group for compound **7a** as multiplet peaks in the range δ 7.42–7.49 ppm and two doublets at for compounds **7b**–**d** δ 7.24–8.13 ppm.

### Biological evaluation

#### In vitro anticancer screening

All newly synthesized compounds were selected and screened for anticancer activity against 60 human cancer cell lines representing 9 types of tumors including leukemia, melanoma, lung, colon, CNS, ovarian, renal, prostate, and breast cancers at the National Cancer Institute (NCI), Bethesda, USA. Screening was performed utilizing a single dose (10^−5^ M concentration) and cells were incubated for 48 h. The end point was determined using sulforhodamine B (SRB) dye. The results are summarized in Table 1. Compounds **3a**–**e** showed potent broad-spectrum anticancer activity against all cancer subpanels with percent inhibition reaching 192.34%. Compound **5b** showed moderate anticancer activity against melanoma UACC-62 and renal cancer UO-31 cell lines with percent inhibition of 40.83% and 31.43% respectively. Interestingly, compound **5f** showed very high potency and selectivity against the melanoma SK-MEL-5 cell line with percent inhibition reached 193.30%. Moreover, compound **6d** showed moderate cytotoxicity against leukemia HL-60(TB) with percent inhibition of 24.69% and 44.84% against leukemia SR cell line. It also showed weak anticancer activity against prostate and renal cancer cell lines with percent inhibition ranging from 11.96 to 27.19%.

**Table 1:** Percent growth inhibition for synthesized compounds at 10^–5^ Molar concentration.

Percent growth inhibition for synthesized compounds at 10^–5^ Molar concentration																
Cell line	**3a**	**3b**	**3c**	**3d**	**3e**	**5a**	**5b**	**5c**	**5d**	**5e**	**5f**	**6**	**7a**	**7b**	**7c**	**7d**
Leukemia																
CCRF-CEM	**152.11**	**147.04**	**70.29**	**54.34**	32.84	3.25	2.48	− 5.55	− 4.97	− 14.64	− 1.98	− 3.91	2.26	− 1.9	− 7.66	− 0.37
HL-60(TB)	**81.89**	34.5	**51.26**	30.48	− 5.14	− 4.08	− 6.96	− 3.17	− 3.16	− 7.56	− 15.1	− 9.74	− 12.04	− 34.92	− 6.9	24.69
K-562	**139.44**	**152.14**	**112.55**	**71.91**	**138.98**	12.86	14.21	5.75	− 0.61	1.07	− 4.63	− 5.37	7.84	0.24	1.62	2.06
MOLT-4	**124.54**	**125.96**	**78.03**	**65.62**	24.92	− 7.4	1	− 2.47	− 7.57	− 5.97	− 22.75	− 17.44	− 6.57	− 19	− 6.42	− 20.06
RPMI-8226	**142.38**	**142.2**	**114.78**	13.38	**136.56**	− 2.33	− 8.08	− 5.59	− 16.76	− 32.67	− 9.03	− 18.32	− 6.81	− 7.01	− 21.57	− 1.55
SR	**114.6**	**129.21**	**95.65**	**66.39**	**104.21**	− 9.18	− 2.85	− 5.16	− 16.02	− 9.06	− 3.09	27.81	− 3.58	1.41	8.55	44.84
Non-small cell lung cancer																
A549/ATCC	**183.91**	**166.62**	**54.7**	14.41	**56.02**	2.07	6.12	− 0.21	− 3.23	− 11.09	− 11.08	− 4.19	− 2.25	1.07	1.75	− 2.01
EKVX	**65.67**	49.25	13.14	37.21	**65.93**	− 3.9	3.04	− 7.93	− 3.99	− 13.2	− 11.14	− 11.27	1.21	− 5.54	− 8.62	− 0.63
HOP-62	31.44	13.53	11.24	40.63	27.59	− 1	− 5.92	− 4.97	− 3.53	− 9.73	− 2.83	− 0.57	4.44	5.56	2.13	7.44
HOP-92	44.25	48.76	38.77	**74.18**	46.18	2.95	9.45	− 1.37	− 16.68	− 30.54	− 18.59	− 12.43	31.8	− 6.06	− 2.65	0.34
NCI-H226	12.6	2.9	2.69	**58.16**	23.97	− 0.19	3.83	− 1.02	− 2.54	− 1.8	2.92	4.91	2.44	1.95	7.33	3.95
NCI-H23	31.52	23.84	9.03	**50.93**	10.24	8.68	7.93	0.84	− 2.12	− 7.59	− 2.71	− 3.85	4.39	− 4.04	− 4.21	− 2.59
NCI-H322M	47.55	16.61	14.23	41.92	15.93	14.29	13.8	5.28	− 3.68	− 4.5	− 3.58	2.38	14.41	5.64	7.81	12.67
NCI-H460	**172.99**	**179.01**	**150.76**	**62.96**	**76.36**	0.79	9.4	− 0.83	− 1.21	− 6.19	− 3.89	− 3.29	4.91	1.2	1.66	− 2.86
NCI-H522	**175.33**	**129.8**	21.53	**61.46**	28.95	7.5	8.89	6.89	6.33	− 4.74	− 5.28	− 5.32	9.08	10.01	9.51	0.62
Colon cancer																
COLO 205	**174.42**	**82.46**	**67.92**	46.79	**141.69**	− 5.85	− 6.29	− 12.44	− 5.07	− 20.79	− 18.18	− 4.74	− 7.19	− 1.38	− 4.05	− 6.77
HCC-2998	**182.9**	**180.57**	**74.08**	**50.37**	**71.41**	0.15	− 9.48	− 0.09	− 6.8	− 10.67	− 15.13	− 6.51	5.26	− 8.81	− 1.94	− 10.43
HCT-116	42.45	28.84	32.32	**66.49**	5.52	0.13	0.67	− 0.34	− 2.4	− 2.72	− 3.77	− 5.49	− 1.61	− 2.89	0.17	2.1
HCT-15	**186.47**	**181.8**	**118.86**	1.76	**75.02**	− 1.48	1.39	− 0.82	− 3.93	2.43	− 0.5	− 5.81	− 3.21	1.3	− 3.42	− 7.33
HT29	**192.02**	**187.2**	**131.72**	**56.33**	**176.69**	− 7.83	− 12.3	− 13.24	− 3.58	− 12.81	− 16.82	− 14.29	− 3.23	− 4.81	− 6.78	− 6.53
KM12	**189.41**	**126.81**	**52.08**	20.67	16.51	0.07	3.94	− 0.28	− 4.42	− 3.69	− 0.45	− 1.64	1.89	3.51	0.72	1.24
SW-620	**181.09**	**184.6**	**174.12**	**73.87**	**116.75**	− 4.88	1.08	− 3.09	0.37	− 1.92	3.4	3.48	− 1.3	0	− 1.98	− 0.01
CNS cancer																
SF-268	**61.11**	**60.61**	37.39	**62.97**	20.88	6.33	6.49	8.23	− 0.76	1.55	1.74	2.98	9.47	6.79	7.02	7.01
SF-295	**91.13**	24.32	25.46	15.05	14.68	4.71	12.99	2	− 3.43	− 11.24	− 3.69	− 2.93	6.9	6.71	3.13	2.82
SF-539	**186.9**	**104.61**	28.6	**68.19**	15.83	0.89	4.4	3.21	0.93	− 0.7	0.79	− 3.11	10.69	9.67	5.06	8.54
SNB-19	**81.07**	**159.44**	45.51	**69.51**	27.05	0.85	1.6	− 6.7	3.07	− 2.2	1.26	− 1.08	− 1.91	4.45	6.42	3.31
SNB-75	7.18	9.9	14.43	41.11	11.52	13.9	17.66	12.77	11.93	6.39	4.26	5.57	21.72	2.37	9.12	13.88
U251	**192.34**	**174.32**	**98.62**	**74.58**	46.72	− 1.53	− 1.16	0.32	− 6.82	− 4.57	− 10.03	− 6.42	2.73	− 4.98	− 4.53	− 5.45
Melanoma																
LOX IMVI	**187.29**	**190.86**	**178.25**	**82.02**	**184.14**	11.49	9.13	14	4.12	− 2.22	4.41	6.49	19.08	− 0.56	4	9.8
MALME-3M	**178.01**	**167.38**	41.33	40.03	**136.7**	2.09	4.32	3.82	− 8.53	− 6.45	− 11.41	− 5.24	4.96	− 4.73	1.6	0.88
M14	**149.68**	**107.07**	**57.28**	10.29	**50.57**	− 5.39	2.06	2.85	− 4.63	− 6.56	− 3.5	− 9.34	9.98	− 3.76	− 4.3	− 3.21
MDA-MB-435	**188.12**	**174.68**	**83.32**	49.01	7.77	2.9	5.85	6.19	1.15	− 0.83	− 3.41	− 1.01	4.07	3.15	6.54	− 0.18
SK-MEL-2	6.57	− 3.54	2.32	44.07	− 2.78	− 22.14	− 11.68	− 4.11	2.33	− 16.26	− 19.05	− 4.5	0.16	− 3.22	− 6.07	− 9.07
SK-MEL-28	**178.84**	**164.33**	8.78	23.1	10.12	3	6.48	5.79	− 5.52	− 4.31	− 6.69	− 5.46	4.44	− 1.67	2.92	− 0.18
SK-MEL-5	**88.14**	47.41	17.59	34.79	30.23	0.76	16.12	− 2.73	− 4.62	− 3.99	**193.3**	− 1.24	6.55	1.22	2.21	0.86
UACC-257	**60.36**	11.01	− 4.28	1.56	− 8.11	− 1.74	− 1.93	− 0.82	− 3.02	− 13.04	− 13.65	− 1.32	− 3.04	− 2.8	− 4.71	− 4.58
UACC-62	**127.89**	35.18	− 1.04	48.61	3.47	21.79	40.83	12.56	9.32	7.88	5.79	7.29	25.01	17.53	18.89	10.93
Ovarian cancer																
IGROV1	42.04	30.61	28.69	**71.73**	32.95	− 3.05	0.82	− 3.57	− 7.42	− 9.15	0.3	− 3.41	3.14	5.97	1.62	− 7.64
OVCAR-3	**174.97**	**183.28**	47.14	**59.75**	31.28	− 4.41	− 3.04	− 8.05	− 9.26	− 12.06	− 12.83	− 2.63	3.69	− 4.89	− 0.96	− 0.39
OVCAR-4	29.29	**76.16**	18.29	**51.85**	23.8	17.61	22.19	8.21	3.58	− 3.82	− 3.86	− 0.77	20.46	16.95	18.94	8.82
OVCAR-5	32.98	9.76	5.97	31.29	32.79	1.71	− 0.73	0.25	− 3.32	− 0.63	1.48	− 6.59	5.65	2.96	2.81	9.27
OVCAR-8	**71.56**	**105.39**	42.19	**70.01**	**56.24**	1.7	7.15	3.62	− 2.61	− 8.06	− 4.66	− 6.45	4.61	− 2.4	− 2.26	− 4.17
NCI/ADR-RES	44.04	21.65	16.72	2.29	32.13	3.34	8.06	− 2.65	0.27	− 8.73	− 6.91	− 4.96	20.41	9.07	13.83	3.34
SK-OV-3	− 4.05	− 8.38	− 9.09	16.16	9.74	9.27	9.6	− 1.51	− 8.06	− 13.96	− 12.93	− 3.29	− 2.06	1.83	2.14	− 4.89
Renal cancer																
786-0	**167.36**	**139.73**	36.11	**54.07**	32.21	7.47	− 3.61	4.78	− 0.21	− 11.24	− 11.85	1.77	14.65	0.58	− 3.71	0.6
A498	0.4	0.84	1.99	40.26	− 4.34	26.63	27.11	24.07	− 3.99	− 13.39	− 11.32	− 0.84	19.1	13.81	19.23	8.25
ACHN	43.18	25.59	11.22	13.6	12.76	2.19	13.98	0.45	− 4.25	− 3.9	− 1.56	− 3.51	5.78	2.27	4.45	2.14
CAKI-1	**54.19**	33.34	31.5	**67.79**	**53.49**	13.02	13.65	11.42	3.88	7.59	11.43	4.66	15.45	13.64	14.9	17.61
RXF 393	**161.32**	**121.57**	38.75	40.7	**83.76**	− 1.39	0.61	− 5.43	− 15	− 7.04	0.98	− 7.78	6.68	6.35	0.35	− 1.25
SN12C	38.27	**104.46**	47.84	69.38	29.96	− 2.53	2.23	− 0.79	− 5.2	3.07	1.96	− 5.98	− 4.77	1.45	3.15	0.88
TK-10	17.31	− 0.87	0.41	29.03	16.09	− 8.31	− 20.44	− 7.73	− 0.75	− 8.44	− 28.97	− 0.31	13.01	− 2.08	− 8.18	− 7.27
UO-31	**52.54**	**79.41**	31.64	12.5	33.17	23.49	31.43	19.14	12.94	9.64	16.7	12.88	28.5	21.38	23.9	27.19
Prostate cancer																
PC-3	**51.09**	30.4	31.65	49.06	23.69	6.83	12.07	4.07	1.64	− 1.74	2.04	− 0.57	13	5.63	9.65	11.96
DU-145	**187.94**	**187.5**	**68.56**	**57.53**	**51.71**	− 3.88	− 3.5	− 9.4	− 6	− 3.31	− 4.6	− 9.97	− 4.71	− 2.33	− 5.05	− 4.57
Breast cancer																
MCF7	**184.35**	**183.53**	**177.31**	**80.93**	**177.45**	7.33	10.63	1.91	5.22	5.24	3.33	− 1.84	6.26	8.87	9.09	5.47
MDA-MB-231/ATCC	**170.45**	**149.57**	45.23	**64.24**	**59.05**	12.9	15.68	13.66	0.31	− 4.28	− 0.24	0.72	16.61	13.26	14.26	8.63
HS 578T	30.29	18.72	26.95	**57.81**	13.95	8.88	11.24	8.53	0.41	2.04	0.4	− 0.41	18.05	4.22	5.57	8.17
BT-549	22.21	17.14	8.32	23.15	38.43	− 6.08	0.23	3.31	0.7	− 16.34	− 32.08	− 9.77	3.33	− 23.49	− 5.29	− 4.45
T-47D	36.35	27.66	35.95	**52.64**	22.18	4.6	11.2	− 0.95	− 7.26	− 8.7	1.12	− 3.85	18.14	6.95	5.3	4.73
MDA-MB-468	**69.56**	28.44	17.51	**69.34**	44.91	7.38	7.4	3.75	− 4.5	− 2.12	3.63	2.93	4.54	3.31	3.92	2.79

N.B: Bold values are growth inhibition percent more than 50%

Compounds **3a**,**b**,**c**,**e** demonstrated a mean growth inhibition more than 47% and therefore were subjected to further analysis at the National Cancer Institute (USA) by determining their growth inhibition 50 (GI_50_) or the concentration required to obtain 50 percent growth inhibition and were compared to two known anticancer agents acting via topoisomerase II inhibition which are etoposide and doxorubicin. The results are reported as Log_10_ GI_50_ for easier demonstration. The synthesized compounds showed a mean value Log_10_ GI_50_ ranging between − 5.74 and − 5.77 which was very comparable to etoposide which demonstrated Log_10_ GI_50_ = − 5.22 but was less than that of doxorubicin Log_10_ GI_50_ = − 7.18 as shown in Table 2

**Table 2:** Log_10_ GI_50_ (Growth inhibition 50) obtained from five doses (10^–4^, 10^–5^, 10^–6^, 10^–7^, 10^–8^ Molar) for test compounds **3a,b,c,e.**

Log_10_ GI_50_ for synthesized compounds (Log_10_ for the concentrations required for 50% inhibition of cell growth)						
Cell line	**3a**	**3b**	**3c**	**3e**	Etoposide	Doxorubicin
Leukemia						
CCRF-CEM	N/A	− 5.81	− 5.83	− 5.82	− 5.9	− 7.2
HL-60(TB)	− 5.74	− 5.73	− 5.73	− 5.72	− 6.1	− 7.3
K-562	− 5.86	− 5.98	− 5.82	− 5.75	− 4.9	− 7
MOLT-4	− 5.76	− 5.76	− 5.77	− 5.75	− 6.3	− 7.7
RPMI-8226	− 5.73	− 5.75	− 5.73	− 5.79	− 5.7	− 7.3
SR	− 5.86	− 5.88	− 5.86	− 5.72	− 6.7	N/A
Non-small cell lung cancer						
A549/ATCC	− 5.77	− 5.76	− 5.77	− 5.74	− 5.4	− 7.3
EKVX	− 5.77	− 5.8	− 5.8	− 5.83	− 4.6	− 6.7
HOP-62	− 5.76	− 5.78	− 5.77	− 5.8	− 5.7	− 7.2
HOP-92	− 5.93	− 5.95	− 5.92	− 5.96	− 5	− 7.5
NCI-H226	− 5.71	− 5.71	− 5.67	− 5.76	− 5.5	− 7.3
NCI-H23	− 5.77	− 5.74	− 5.74	− 5.75	− 5.2	− 7.2
NCI-H322M	− 5.75	− 5.77	− 5.77	− 5.8	− 4.1	− 6.8
NCI-H460	− 5.74	− 5.77	− 5.79	− 5.76	− 6.3	− 7.9
NCI-H522	− 5.75	− 5.75	− 5.73	− 5.75	− 5.2	− 9.4
Colon cancer						
COLO 205	− 5.77	− 5.77	− 5.75	− 5.75	− 4.4	− 6.9
HCC-2998	− 5.75	− 5.73	− 5.73	− 5.75	− 5	− 6.8
HCT-116	− 5.77	− 5.76	− 5.81	− 5.75	− 4.9	− 7
HCT-15	− 5.84	− 5.84	− 5.84	− 5.83	− 4.8	− 6.6
HT29	− 5.82	− 5.81	− 5.78	− 5.78	− 4.5	− 6.6
KM12	− 5.72	− 5.74	− 5.73	− 5.76	− 4.7	− 6.9
SW-620	− 5.75	− 5.77	− 5.77	− 5.77	− 5.2	− 7.2
CNS cancer						
SF-268	− 5.71	− 5.74	− 5.75	− 5.76	− 5.1	− 7.1
SF-295	− 5.74	− 5.74	− 5.73	− 5.73	− 5.2	− 7.1
SF-539	− 5.77	− 5.8	− 5.77	− 5.79	− 5.5	− 7.2
SNB-19	− 5.77	− 5.77	− 5.77	− 5.79	− 5.1	− 7.1
SNB-75	− 5.75	− 5.82	− 5.8	− 5.83	− 4.9	− 6.7
U251	− 5.8	− 5.79	− 5.78	− 5.78	− 5.5	− 7.3
Melanoma						
LOX IMVI	− 5.78	− 5.77	− 5.76	− 5.77	− 5.6	− 7.4
MALME-3M	− 5.79	− 5.77	− 5.75	− 5.78	− 5	N/A
M14	− 5.75	− 5.74	− 5.72	− 5.74	− 6.1	− 7.1
MDA-MB-435	− 5.72	− 5.77	− 5.76	− 5.75	− 4.5	− 6.6
SK-MEL-2	− 5.74	− 5.7	− 5.7	− 5.71	− 4.7	− 9.8
SK-MEL-28	− 5.75	N/A	N/A	N/A	− 4.7	− 6.6
SK-MEL-5	− 5.75	− 5.76	− 5.71	− 5.75	− 5.3	− 7.2
UACC-257	− 5.76	− 5.78	− 5.77	− 5.74	− 4.4	− 7.3
UACC-62	− 5.76	− 5.75	− 5.73	− 5.79	− 5.2	− 7
Ovarian cancer						
IGROV1	− 5.77	− 5.8	− 5.8	− 5.79	− 4.5	− 10.6
OVCAR-3	− 5.74	− 5.74	− 5.75	− 5.75	− 4.4	− 6.6
OVCAR-4	− 5.69	− 5.7	− 5.61	− 5.71	− 3.9	− 6.6
OVCAR-5	− 5.78	− 5.77	− 5.76	− 5.8	− 4.6	− 6.6
OVCAR-8	− 5.76	− 5.75	− 5.76	− 5.74	− 4.9	− 6.8
NCI/ADR-RES	− 5.75	− 5.74	− 5.71	− 5.74	− 4.2	− 6.6
SK-OV-3	− 5.74	− 5.75	− 5.71	− 5.75	− 4.8	− 6.6
Renal cancer						
786-0	− 5.77	− 5.79	− 5.77	− 5.75	− 5.9	− 7.2
A498	− 5.72	− 5.89	− 5.75	− 5.44	− 5.1	N/A
ACHN	− 5.76	− 5.79	− 5.77	− 5.78	− 6.1	− 7.1
CAKI-1	− 5.76	− 5.76	− 5.78	− 5.78	− 5.4	− 6.6
RXF 393	− 5.81	− 5.77	− 5.79	− 5.83	− 4.8	N/A
SN12C	− 5.77	− 5.79	− 5.8	− 5.81	− 5.3	− 7.1
TK-10	− 5.77	− 5.76	− 5.76	− 5.76	− 5.3	− 7.4
UO-31	− 5.81	− 5.84	− 5.81	− 5.82	− 4.4	− 7.3
Prostate cancer						
PC-3	− 5.8	− 5.79	− 5.79	− 5.84	− 6.2	− 6.6
DU-145	− 5.73	− 5.78	− 5.76	− 5.78	− 6.1	− 7.2
Breast cancer						
MCF7	− 5.75	− 5.79	− 5.81	− 5.77	− 5.7	− 7.9
MDA-MB-231/ATCC	− 5.77	− 5.77	− 5.74	− 5.82	− 5.8	− 6.6
HS 578T	− 5.74	− 5.36	− 5.7	− 4.85	− 6.1	− 6.7
BT-549	− 5.8	− 5.79	− 5.8	− 4.91	− 5.6	− 7
T-47D	− 5.82	− 5.79	− 5.78	− 5.77	− 6	− 6.6
MDA-MB-468	− 5.79	− 5.78	− 5.8	− 5.78	N/A	N/A
Mean Log_10_ GI_50_	− 5.77	− 5.77	− 5.76	− 5.74	− 5.22	− 7.18

N/A: not available.

#### Cell cycle analysis

**Figure 8:**
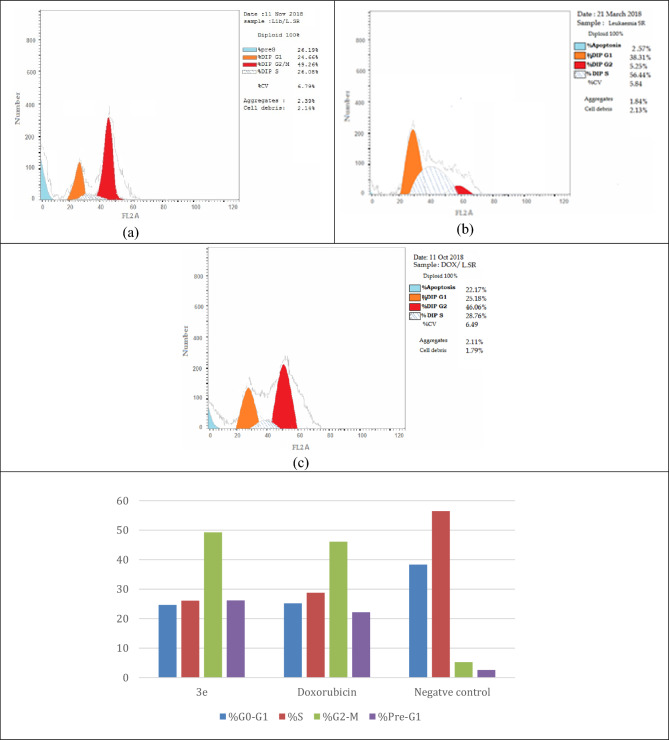
Effect of compound **3e** (**a**) on DNA-ploidy flow cytometric analysis of leukemia SR cells in comparison with negative control (**b**) and doxorubicin (**c**). Analysis performed using Cell Quest software 5.2.1 (https://www.bdbiosciences.com, Becton Dickinson Immunocytometry Systems, San Jose, CA).

Compound **3e** was found to be one of the most potent compounds during the anticancer activity screening and therefore was further evaluated by cell cycle analysis and apoptosis determination. Significant alterations in the cell cycle phases were observed when leukemia SR cells were treated with compound **3e** as shown in Fig. 8. Results demonstrated a decrease in the percentage of cells at the G0-G1 and S phases in comparison to control. Compound **3e** showed 24.66% and 26.08% of cells. This decrease in the percentage of cells at G0-G1 and S phases was found to be comparable to that induced by doxorubicin (25.18% and 28.76%). On the other hand, compound **3e** caused a significant increase in the G2-M phase with 49.26% in comparison to control (5.25%) and comparable to doxorubicin (46.06%). Finally, the percentage of cells at the pre-G1 phase was increased by by 10.19 folds by compound **3e** which is higher than doxorubicin which caused 8.62 folds increase. Based on the above findings, it can be concluded that compound **3e** exhibited cell arrest at G2-M phase leading to inhibition of cell proliferation and induced apoptosis.

#### Determination of apoptosis using annexin-V

**Figure 9:**
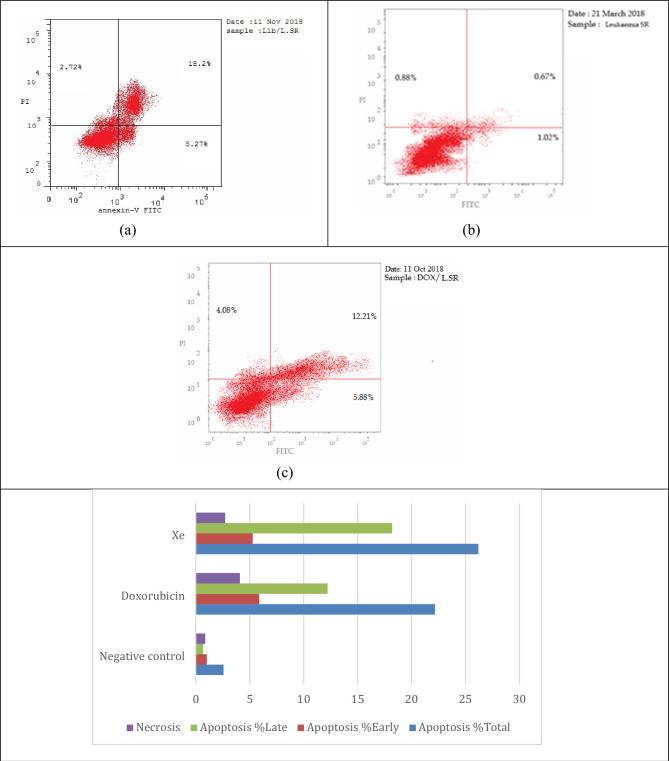
Effect of compound **3e** (**a**) on the percentage of Annexin-V-FITC-positive staining in leukemia SR cells in comparison with negative control (**b**) and doxorubicin (**c**). Analysis performed using BD FACS Calibur (https://www.bdbiosciences.com, BD Biosciences, San Jose, CA).

The evaluation of the apoptotic effect of compound **3e** showed a significant elevation in the percentage of the early and late apoptosis in comparison to control with 10.19- and 27.16-folds, respectively more than control leukemia SR cells. The necrosis percent induced by compound **3e** and doxorubicin was 2.72% and 4.08% which were noticeably higher than control that showed only 0.88% as demonstrated in Fig. 9.

#### Topoisomerase II inhibitory activity

**Figure 10:**
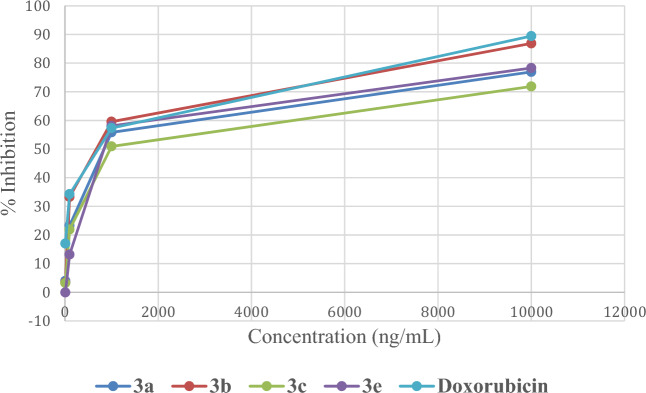
Dose response curve presentation showing IC_50_ against Topoisomerase IIα for compound **3a,b,c,e**

The inhibitory activity of topoisomerase IIα was evaluated for compounds **3a,b,c,e** using topoisomerase enzyme-linked immunosorbent assay (ELISA) kit since they were the most potent compounds among the synthesized series. Four concentrations were used and IC_50_ was calculated. Doxorubicin, a known inhibitor of topoisomerase enzymes, was used as reference. the results were reported in Fig. 10. Compound **3e** was the most potent and had comparable topoisomerase inhibitory activity to doxorubicin with IC_50_ of 0.98 µM.

#### Molecular modeling study

**Figure 11:**
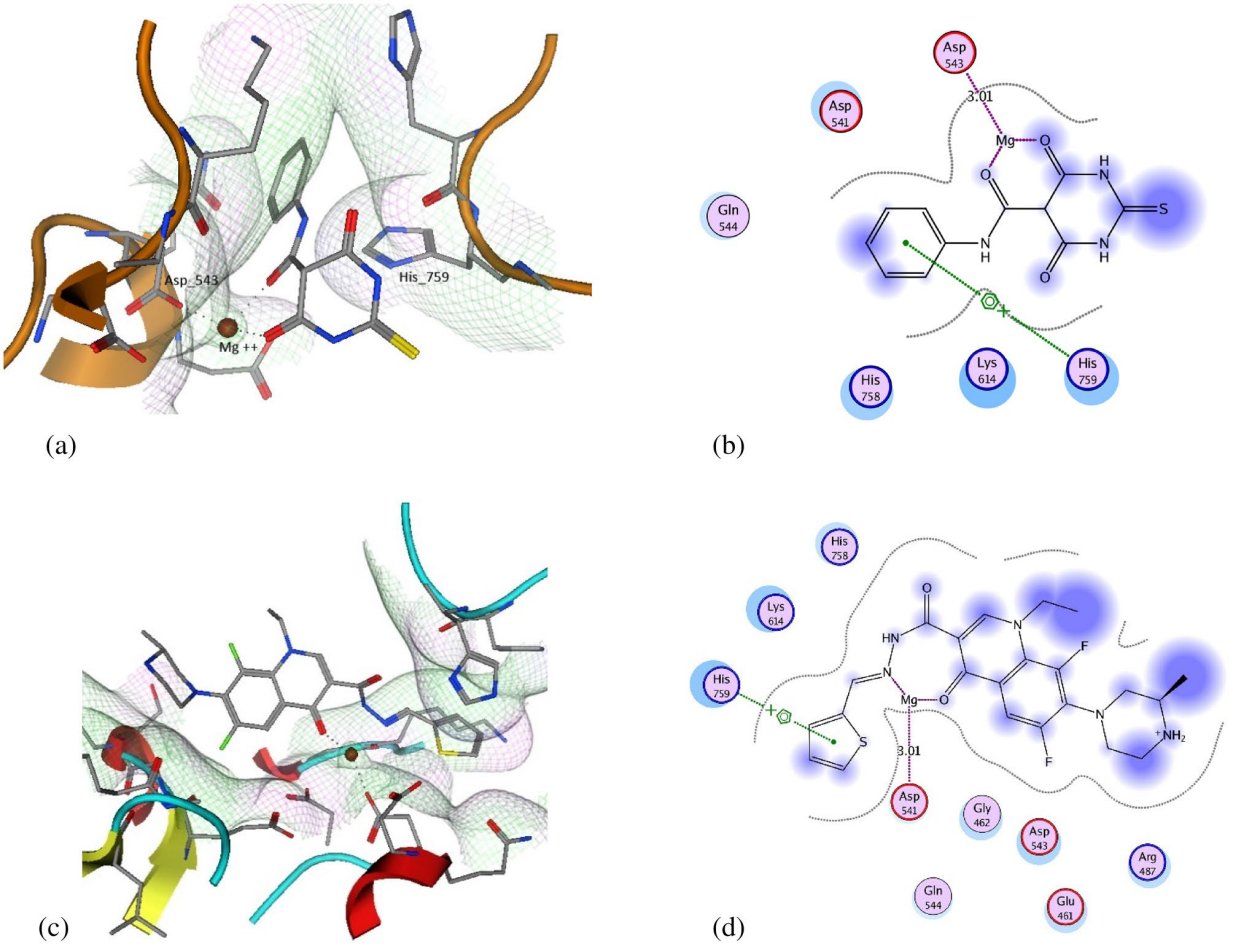
(**a**) 3D interaction of merbarone with DNA binding site of topoisomerase IIα, (**b**) 2D interaction of merbarone with DNA binding site of topoisomerase IIα, (**c**) 3D interaction of compound **3e** with DNA binding site of topoisomerase IIα and (**d**) 2D interaction of compound **3e** with DNA binding site of topoisomerase IIα. Generated by MOE (Molecular Operating Environment, http://www.chemcomp.com) 2008.10 software.

Out of several topoisomerase II structures available we selected (PDB ID: 4FM9)^20^ for this study which has topoisomerase IIα co-crystallized with DNA. The DNA chains were removed from the active site. Validation of the molecular modeling setup was performed by docking of merbarone and comparing the docking results to a previously reported study^21^. the binding pattern of merbarone indicated that the used setup was suitable for the molecular modeling study. An energy score (S) of − 20.72 kcal/mol was demonstrated by merbarone. Merbarone was also able to reproduce the coordinate bond interactions with Mg^2+^ and arene cation interaction with His 759. Compound **3e** was able to produce a similar binding pattern by forming a coordinate bond with Mg^2+^ with the carbonyl groups at position 3 and 4 as well as arene cation interaction with His 759 with the thiophene moiety. Compound **3e** yielded an energy score (S) of − 22.19 kcal/mol (Fig. 11).

## Conclusion

A series of C-3 and C-7 lomefloxacin derivatives were synthesized and characterized by their spectral data. Screening for anticancer activity utilizing single dose revealed that compounds **3a**–**e** showed potent anticancer activity against all cancer types utilized. Compound **5f** showed highly selective anticancer activity against the melanoma SK-MEL-5 cell line. compound **3e** exhibited cell arrest at G2-M phase. Moreover, compound **3e** showed high topoisomerase inhibitory activity with IC_50_ of 0.98 µM. This study showed that the newly synthesized hydrazide derivatives of lomefloxacin **3a,b,c,e** represent a promising lead for the synthesis of highly potent anticancer agents that acts through topoisomerase II inhibition.

## Materials and methods

### General

Melting points were determined on Stuart SMP10 apparatus and the values given were uncorrected. IR spectra were determined as KBr discs on Shimadzu IR 8400 s spectrophotometer, Faculty of Pharmacy, Cairo University, Egypt and values were represented in cm^−1^. ^1^H NMR spectra were carried out using: Bruker 400-BB 400 MHz using tetramethylsilane (TMS) as internal standard and chemical shift values were recorded in ppm on δ scale, Micro Analytical Unit, Faculty of Pharmacy, Cairo University, Egypt. ^13^C NMR spectra were carried out using: Bruker 400-BB 100 MHz using tetramethylsilane (TMS) as internal standard and chemical shift values were recorded in ppm on δ scale, Micro Analytical Unit, Faculty of Pharmacy, Cairo University, Egypt. Mass spectra were run at 70 eV on Hewlett Packard 5988 spectrometer, Micro Analytical Center, Cairo University, Egypt. Element analyses were carried out at the Regional Center for Mycology and Biotechnology, Faculty of Pharmacy, Al Azhar University, Egypt. Progress of the reactions was monitored by TLC using aluminum sheets precoated with UV fluorescent silica gel (Merck 60 F254) and spots were visualized using UV lamp. The solvent system used was dichloromethane: toluene: ethanol [9: 5: 1]. Chemical Structures were generated using chemdraw software 2021^22^. Molecular modeling study was done using Molecular Operating Environment (MOE 2008.10) software^23^. Evaluation of the cytotoxic activity was performed at The National Cancer Institute (United States of America). Evaluation of cell cycle analysis, apoptosis and topoisomerase IIα inhibitory activity was performed at the confirmatory diagnostic unit, VACSERA.

### Chemistry

#### 1-Ethyl-6,8-difluoro-7-(3-methylpiperazin-1-yl)-4-oxo-1,4-dihydroquinoline-3-carbohydrazide (**2**)

Lomefloxacin hydrochloride salt **1** (3.87 g, 0.01 mol) and hydrazine hydrate (100%, 25 mL) were heated under reflux for 24 h. The reaction mixture was allowed to cool. The formed precipitate was filtered, dried and recrystallized from 70% ethanol to afford **2**.

Yellowish white solid; mp: 212–14 °C; yield: 1.94 (53%); IR (KBr) νmax:: 3421 (NH), 3336, 3313 (NH_2_), 1650–1640 (2C=O overlapped), 1585 (C=C); ^1^H NMR (400 MHz, DMSO-d_6_): δ 0.97 (d, 3H, *J* = 6.0 Hz, CH_3_), 1.38 (t, 3H, *J* = 6.0 Hz, CH_3_), 2.70–2.72 (m, 1H, CH), 2.80–2.90 (m, 4H, 2CH_2_), 2.98–3.10 (m, 2H, CH_2_), 3.22 (s, 2H, NH_2_, D_2_O exchangeable), 4.25–4.29 (m, 2H, CH_2_), 6.63 (s, 1H, NH, D_2_O exchangeable), 7.71 (s, 1H, ArH), 8.59 (s, 1H, ArH), 10.75 (s, 1H, NH, D_2_O exchangeable) ppm.

#### *N*′-Arylidene-1-ethyl-6,8-difluoro-7-(3-methylpiperazin-1-yl)-4-oxo-1,4-dihydroquinoline-3-carbohydrazide (**3a**–**e**)

The target compounds **3a-e** were synthesized by dissolving lomefloxacin hydrazide **2** (0.50 g, 1.40 mmol) and the appropriate aldehyde (1.40 mmol) in absolute ethanol (20 mL) containing 0.20 mL glacial acetic acid and the mixture was heated under reflux for 12 h. The mixture was allowed to cool, the formed solid was filtered and recrystallized from toluene to afford a yellow solid **3a**–**e**.

##### *N'*-Benzylidene-1-ethyl-6,8-difluoro-7-(3-methylpiperazin-1-yl)-4-oxo-1,4-dihydroquinoline-3-carbohydrazide (**3a**)

Yellow solid; mp: > 262–64 °C; yield: 0.39 g, 63%; IR (KBr) νmax: 3444 (NH), 3298 (NH), 1690 (C=O), 1666 (C=O), 1535 (C=C) cm^−1^; ^1^H NMR (400 MHz, DMSO-d_6_): δ 1.09 (d, 3H, *J* = 4.0 Hz, CH_3_), 1.43 (t, 3H, *J* = 7.2 Hz, CH_3_), 2.88–3.06 (m, 4H, 2CH_2_), 3.20–3.29 (m, 3H, CH_2_ and CH), 4.49–4.53 (q, 2H, *J* = 7.2 Hz, CH_2_), 6.10 (s, 0.5H, OH, D_2_O exchangeable, taut B), 7.39–7.40 (m, 1.5H, ArH, taut A), 7.46–7.48 (m, 1.5H, ArH, taut B), 7.71–7.72 (d, 1H, *J* = 7.2 Hz, ArH, taut A), 7.75–7.77 (d, 1H, *J* = 7.6 Hz, ArH, taut B), 8.14 (s, 1H, ArH), 8.32 (s, 0.5H, N=CH, taut A), 8.44 (s, 0.5H, N=CH, taut B), 8.74 (s, 1H, ArH), 9.60 (s, 1H, NH, D_2_O exchangeable), 13.33 (s, 0.5H, NH, D_2_O exchangeable, taut A) ppm; ^13^C NMR (400 MHz, DMSO-d_6_): δ 16.5, 18.8, 21.9, 47.9, 49.9, 50.7, 53.6, 57.1 (Aliphatic Cs), 102.5, 109.3, 126.51, 127.6, 127.8, 129.2, 129.3, 130.5, 132.9, 134.9, 135.7, 137.2, 141.0, 142.3, 148.1, 148.8, 161.5, 167.8 (Aromatic Cs), 172.9 (C=O), 174.0 (C = O) ppm; MS [m/z, %]: 454.23 [M+1^ך^·^+^, 1.76], 453.27 [M^ך^·^+^, 5.55], 450.20 [100]; Anal. Calcd for C_24_H_25_F_2_N_5_O_2_ (453.48): C, 63.56; H, 5.79; N, 15.44. Found: C, 63.43; H, 5.79; N, 15.70.

##### 1-Ethyl-6,8-difluoro-N′-(3-fluorobenzylidene)-7-(3-methylpiperazin-1-yl)-4-oxo-1,4-dihydroquinoline-3-carbohydrazide (**3b**)

Yellow solid; mp: 238–40 °C; yield: 0.39 g, 63%; IR (KBr) νmax: 3448 (NH), 3302 (NH), 1680 (C=O), 1675 (C=O), 1593 (C=C) cm^−1^; ^1^H NMR (400 MHz, DMSO-d_6_): δ 1.04–1.06 (d, 3H, *J* = 6.4 Hz CH_3_), 1.42–1.45 (t, 3H, *J* = 7.2 Hz CH_3_), 2.85–2.97 (m, 4H, 2CH_2_), 3.16–3.17 (m, 3H, CH_2_ and CH), 4.53–4.55 (m, 2H, CH_2_), 4.78 (s, 0.5H, OH, D_2_O exchangeable, taut B), 7.22–7.28 (m, 1H, ArH), 7.51–7.59 (m, 3H, ArH), 8.13 (s, 1H, ArH), 8.33 (s, 0.5H, N=CH, taut A), 8.48 (s, 0.5H, N=CH, taut B), 8.76 (s, 1H, ArH), 9.70 (s, 1H, NH, D_2_O exchangeable), 13.39 (s, 0.5H, NH, D_2_O exchangeable, taut A) ppm; ^13^C NMR (400 MHz, DMSO-d_6_): δ 16.5, 19.0, 21.7, 50.0, 51.2, 53.7, 56.5, 59.8 (Aliphatic Cs), 102.7, 109.2, 117.0, 120.3, 122.9, 124.0, 131.4, 132.4, 140.7, 141.2, 144.7, 147.0, 149.0, 155.5, 161.9 (Aromatic Cs), 172.6 (C=O), 174.0 (C=O) ppm; MS [m/z, %]: 472.57 [M+1^ך^·^+^, 21.42], 471.57 [M^ך^·^+^, 72.57], 282.30 [100]. Anal. Calcd for C_24_H_24_F_3_N_5_O_2_ (471.47): C, 61.14; H, 5.13; N, 14.85. Found: C, 61.38; H, 5.27; N, 15.03.

##### *N'*-(4-Bromobenzylidene)-1-ethyl-6,8-difluoro-7-(3-methylpiperazin-1-yl)-4-oxo-1,4-dihydroquinoline-3-carbohydrazide (**3c**)

Yellow solid; mp: 247–49 °C; yield: 0.48 g, 66%; IR (KBr) νmax: 3433 (NH), 3278 (NH), 1690 (C=O), 1670 (C=O), 1550 (C=C) cm^−1^; ^1^H NMR (400 MHz, DMSO-d_6_): δ 0.99 (d, 3H, *J* = 4.0 Hz, CH_3_), 1.44 (t, 3H, *J* = 4.0 Hz, CH_3_), 2.74–2.78 (m, 1H, CH), 2.85–2.92 (m, 2H, CH_2_), 3.10–3.11 (m, 4H, 2CH_2_), 4.53–4.54 (q, 2H,* J* = 4.0 Hz, CH_2_), 7.17 (d, 1H, *J* = 7.2 Hz, ArH, taut A), 7.24 (d, 1H, *J* = 7.2 Hz, ArH, taut B), 7.65–7.68 (m, 2H, ArH), 7.71 (s, 0.5H, OH, D_2_O exchangeable, taut B), 8.11 (s, 1H, ArH), 8.31 (s, 0.5H, N=CH, taut A), 8.42 (s, 0.5H, N=CH, taut B), 8.75 (s, 1H, ArH), 9.59 (s, 1H, NH, D_2_O exchangeable), 13.36 (s, 0.5H, NH, D_2_O exchangeable, taut A) ppm.; ^13^C NMR (400 MHz, DMSO-d_6_): δ 16.5, 19.2, 21.8, 50.3, 50.7, 53.7, 53.8, 57.6 (Aliphatic Cs), 102.5, 109.2, 122.3, 123.7, 128.3, 129.5, 130.7, 132.3, 132.3, 132.5, 134.2, 134.9, 140.8, 141.1, 146.9, 148.9, 161.6, 161.6 (Aromatic Cs), 172.7 (C=O), 174.0 (C=O) ppm.; MS [m/z, %]: 534.78 [M+2^ך^·^+^, 14.21], 533.78 [M+1^ך^·^+^, 17.89], 532.78 [M^ך^·^+^, 13.28], 299.28 [100]; Anal. Calcd for C_24_H_24_BrF_2_N_5_O_2_ (532.38): C, 54.14; H, 4.54; N, 13.15. Found: C, 54.35; H, 4.66; N, 13.37.

##### 1-Ethyl-6,8-difluoro-7-(3-methylpiperazin-1-yl)-4-oxo-*N'*-(pyridin-3-ylmethylene)-1,4-dihydroquinoline-3-carbohydrazide (**3d**)

Yellow solid; mp: 241–43 °C; yield: 0.22 g, 36%; IR (KBr) νmax: 3425 (NH), 3298 (NH), 1690 (C=O), 1670 (C=O), 1604 (C=C) cm^−1^; ^1^H NMR (400 MHz, DMSO-d_6_): δ 1.04 (d, 3H, *J* = 6.4 Hz, CH_3_), 1.45 (t, 3H, *J* = 7.0 Hz, CH_3_), 2.81–2.86 (m, 1H, CH), 2.91–2.99 (m, 2H, CH_2_), 3.12–3.19 (m, 4H, 2CH_2_), 4.39 (s, 0.5H, OH, D_2_O exchangeable, taut B), 4.54–4.57 (q, 2H, *J* = 7.0 Hz, CH_2_), 7.48–7.53 (m, 1H, ArH, taut A), 8.09–8.14 (m, 1H, ArH, taut B), 8.16 (s, 1H, ArH), 8.39 (s, 0.5H, N=CH, taut A), 8.52 (s, 0.5H, N=CH, taut B), 8.57 (dd, 0.5H, *J* = 3.3 Hz, *J* = 1.4 Hz, ArH, taut A), 8.62 (dd, 0.5H, *J* = 3.3 Hz, *J* = 1.4 Hz, ArH, taut B), 8.77 (s, 1H, ArH), 8.85 (d, 0.5H, *J* = 1.4 Hz, ArH, taut A), 8.87 (d, 0.5H, *J* = 1.4 Hz, ArH, taut B), 9.76 (s, 1H, NH, D_2_O exchangeable), 13.45 (s, 0.5H, NH, D_2_O exchangeable, taut A) ppm.; ^13^C NMR (400 MHz, DMSO-d_6_): δ 16.5, 18.9, 21.9, 50.0, 50.7, 53.7, 53.8, 57.2 (Aliphatic Cs), 102.7, 109.2, 122.2, 124.4, 124.5, 127.7, 129.5, 130.8, 131.6, 132.8, 133.9, 139.1, 140.7, 145.5, 148.2, 148.9, 149.2, 149.9, 151.1, 161.6 (Aromatic Cs), 172.8 (C=O), 174.0 (C=O) ppm.; MS [m/z, %]: 455.47 [M+1^ך^·^+^, 16.53], 454.47 [M^ך^·^+^, 27.44], 43.01 [100]; Anal. Calcd for C_23_H_24_F_2_N_6_O_2_ (454.47): C, 60.78; H, 5.32; N, 18.49. Found: C, 61.04; H, 5.53; N, 18.32.

##### 1-Ethyl-6,8-difluoro-7-(3-methylpiperazin-1-yl)-4-oxo-*N'*-(thiophen-2-ylmethylene)-1,4-dihydroquinoline-3-carbohydrazide (**3e**)

Yellow solid; mp: 250–52 °C; yield: 0.37 g, 58%; IR (KBr) νmax: 3421 (NH), 3294 (NH), 1670 (C=O), 1640 (C=O), 1600 (C=C) cm^−1^; ^1^H NMR (400 MHz, DMSO-d_6_): δ 0.98 (d, 3H, *J* = 4.0 Hz, CH_3_), 1.43 (t, 3H, *J* = 4.0 Hz, CH_3_), 2.20 (s, 0.5H, OH, D_2_O exchangeable, taut B), 2.74–2.78 (m, 1H, CH), 2.84–2.90 (m, 2H, CH_2_), 3.08–3.10 (m, 4H, 2CH_2_), 4.54–4.56 (q, 2H, *J* = 4.0 Hz, CH_2_), 7.13 (dd, 0.5H, *J* = 3.6 Hz, *J* = 1.4 Hz, ArH, taut A), 7.16 (dd, 0.5H, *J* = 3.6 Hz, *J* = 1.4 Hz, ArH, taut B), 7.31 (dd, 0.5H, *J* = 2.9 Hz, *J* = 0.8 Hz, ArH, taut A) 7.45 (dd, 0.5H, *J* = 2.9 Hz, *J* = 0.8 Hz, ArH, taut B), 7.60 (d, 0.5H, *J* = 5.0 Hz, ArH, taut A), 7.69 (d, 0.5H, *J* = 5.0 Hz, ArH, taut B), 8.04 (s, 1H, ArH), 8.57 (s, 0.5H, N=CH, taut A), 8.72 (s, 0.5H, N=CH, taut B) 8.73 (s, 1H, ArH), 9.51 (s, 1H, NH, D_2_O exchangeable), 13.29 (s, 0.5H, NH, D_2_O exchangeable, taut A) ppm; ^13^C NMR (400 MHz, DMSO-d_6_) δ 16.5, 19.4, 21.9, 50.5, 50.7, 53.6, 53.8, 57.8 (Aliphatic Cs), 102.3, 109.3, 122.0, 122.7, 127.6, 128.3, 128.7, 129.4, 131.5, 136.9, 137.9, 139.5, 140.5, 140.7, 143.4, 148.7, 156.8, 161.4 (Aromatic Cs), 172.7 (C=O), 173.9 (C=O) ppm; MS [m/z, %]: 460.07 [M+1^ך^·^+^, 7.01], 459.07 [M^ך^·^+^, 8.69], 334.96 [100]. Anal. Calcd for C_22_H_23_F_2_N_5_O_2_S (459.51): C, 57.50; H, 5.05; N, 15.24. Found: C, 57.68; H, 5.21; N, 15.48.

#### General procedure for the synthesis of compounds** 4a–f**

The selected substituted aniline derivatives (0.01 mol) and triethylamine (0.01 mol) were dissolved in dichloromethane (15 mL) in an ice bath. Chloroacetyl chloride (0.01 mol) in dichloromethane (10 mL) was added dropwise to the reaction mixture. The temperature was maintained at 0 °C through out the addition process. The reaction mixture was stirred for 12 h. Dichloromethane was evaporated and the precipitate was washed several times with distilled water and recrystallized from distilled water to afford intermediates **4a**–**f**^18,19^.

#### General procedure for the synthesis of compounds** 5a–f**

A mixture of lomefloxacin hydrochloride **1** (0.38 g, 0.001 mol), the appropriate 2-choloro-N-substituted acetamides **4a-f** (0.001 mol) and triethylamine (0.20 g, 0.002 mol) in absolute ethanol (20 mL) was heated under reflux for 24 h. The formed precipitate was filtered off and recrystallized from 70% ethanol to give compounds **5a-f**.

##### 1-Ethyl-6,8-difluoro-7-(3-methyl-4-(2-oxo-2-(phenylamino)ethyl)piperazin-1-yl)-4-oxo-1,4-dihydroquinoline-3-carboxylic acid (**5a**)

White solid; mp: 248–50 °C; yield: 0.24 g, 50%; IR (KBr) νmax: 3444–2835 (OH), 3267 (NH), 1728 (C=O), 1689 (2C=O overlapped), 1519 (C=C). cm^−1^; ^1^H NMR (400 MHz, DMSO-d_6_): δ 1.09, 1.15 (d, 3H, *J* = 6.2 Hz, CH_3_), 1.45 (t, 3H, *J* = 6.2 Hz, CH_3_), 2.71–2.83 (m, 2H, CH_2_), 2.93–2.96 (m, 1H, CH), 3.13–3.24 (m, 4H, 2CH_2_), 3.39 (s, 2H, CH_2_), 4.57–4.60 (q, 2H, *J* = 6.2 Hz, CH_2_), 7.07 (t, 1H, *J* = 8.0 Hz, ArH), 7.32 (t, 2H, *J* = 8.0 Hz, ArH), 7.66 (d, 2H,* J* = 8.0 Hz, ArH), 7.85 (d, 1H, *J* = 11.2 Hz, ArH), 8.92 (s, 1H, ArH), 9.75 (s, 1H, NH, D_2_O exchangeable) ppm.; ^13^C NMR (400 MHz, DMSO-d_6_) δ 15.8, 16.4, 16.5, 51.1, 52.8, 54.1, 55.7, 57.4, 58.1 (Aliphatic Cs), 107.4, 112.0, 120.7 (d,* J* = 32.0 Hz), 123.9, 127.8 (d,* J* = 28.0 Hz), 129.1, 134.2, 138.9, 144.9, 147.4, 151.6, 153.7 (Aromatic Cs), 166.0 (C=O), 169.6 (C=O), 175.9 (C=O) ppm.; MS [m/z, %]: 485.45 [M+1^ך^·^+^, 0.53], 484.45 [M^ך^·^+^, 0.92], 364.35 [100]. Anal. Calcd for C_25_H_26_F_2_N_4_O_4_ (484.50): C, 61.98; H, 5.41; N, 11.56. Found: C, 62.21; H, 5.49; N, 11.80.

##### 7-(4-(2-((4-Chlorophenyl)amino)-2-oxoethyl)-3-methylpiperazin-1-yl)-1-ethyl-6,8-difluoro-4-oxo-1,4-dihydroquinoline-3-carboxylic acid (**5b**)

White solid; mp: 218–20 °C; yield: 0.29 g, 56%; IR (KBr) νmax: 3421–2727 (OH), 3286 (NH), 1716 (C=O), 1690 (C=O), 1666 (C=O), 1558 (C=C). cm^−1^; ^1^H NMR (400 MHz, DMSO-d_6_): δ 1.04–1.09 (m, 3H, CH_3_), 1.44 (t, 3H, *J* = 4.0 Hz, CH_3_), 2.71–2.80 (m, 1H, CH), 2.92–2.99 (m, 2H, CH_2_), 3.12–3.45 (m, 4H, 2CH_2_), 3.40 (s, 2H, CH_2_), 4.57–4.58 (q, 2H, *J* = 4.0 Hz, CH_2_), 7.37 (d, 2H, *J* = 8.8 Hz, ArH), 7.71 (d, 2H, *J* = 8.8 Hz, ArH), 7.82 (d, 1H, *J* = 10.2 Hz, ArH), 8.91 (s, 1H, ArH), 9.88 (s, 1H, NH, D_2_O exchangeable) ppm.; ^13^C NMR (400 MHz, DMSO-d_6_): δ 16.3, 16.4, 18.7, 45.8, 50.6, 52.7, 54.2, 55.7, 58.1 (Aliphatic Cs), 107.2, 107.4, 120.5 (d,* J* = 36.0 Hz), 121.6, 127.6 (d,* J* = 40.0 Hz), 128.9, 134.1, 134.2, 137.8, 147.3, 151.4, 156.1 (Aromatic Cs), 166.1 (C=O), 169.9 (C=O), 175.8 (C=O) ppm; MS [m/z, %]: 520.39 [M+2^ך^·^+^, 4.27], 519.55 [M+1^ך^·^+^, 7.91], 518.70 [M^ך^·^+^, 13.59], 55.09 [100]. Anal. Calcd for C_25_H_25_ClF_2_N_4_O_4_ (518.94): C, 57.86; H, 4.86; N, 10.80. Found: C, 58.04; H, 5.70; N, 11.68.

##### 1-Ethyl-6,8-difluoro-7-(4-(2-((4-methoxyphenyl)amino)-2-oxoethyl)-3-methylpiperazin-1-yl)-4-oxo-1,4-dihydroquinoline-3-carboxylic acid (**5c**)

White solid; mp: 196–98 °C; yield: 0.19 g, 37%; IR (KBr) νmax: 3421–2646 (OH), 3282 (NH), 1724 (C=O), 1680 (C=O),1658 (C=O), 1558 (C=C) cm^−1^; ^1^H NMR (400 MHz, DMSO-d_6_): δ 1.08 (d, 3H, *J* = 4.0 Hz, CH_3_), 1.45 (t, 3H, *J* = 4.0 Hz, CH_3_), 2.69–2.72 (m, 1H, CH), 2.78–2.98 (m, 4H, 2CH_2_), 3.10–3.20 (m, 2H, CH_2_), 3.44 (s, 3H, CH_3_), 3.73 (s, 2H, CH_2_), 4.57–4.59 (q, 2H, *J* = 4.0 Hz, CH_2_), 6.89 (d, 2H, *J* = 8.8 Hz, ArH), 7.55 (d, 2H, *J* = 8.8, ArH), 7.82–7.86 (m, 1H, ArH), 8.91 (s, 1H, ArH), 9.61 (s, 1H, NH, D_2_O exchangeable) ppm; ^13^C NMR (400 MHz, DMSO-d_6_): δ 16.4, 19.2, 46.1, 51.2, 52.8, 54.1, 55.7, 57.4, 58.1 (Aliphatic Cs), 107.3, 107.5, 114.2, 120.7 (d,* J* = 28.0 Hz), 121.6, 127.7 (d,* J* = 28.0 Hz), 132.1, 134.2, 134.4, 151.6, 153.7, 155.9 (Aromatic Cs), 166.0 (C=O), 169.1 (C=O), 175.9 (C=O) ppm.; MS [m/z, %]: 515.10 [M+1^ך^·^+^, 0.75], 514.09 [M^ך^·^+^, 2.26], 294.96 [100]. Anal. Calcd for C_26_H_28_F_2_N_4_O_5_ (514.52): C, 60.69; H, 5.49; N, 10.89. Found: C, 60.92; H, 5.60; N, 11.03.

##### 1-Ethyl-6,8-difluoro-7-(3-methyl-4-(2-oxo-2-((4-sulfamoylphenyl)amino)ethyl)piperazin-1-yl)-4-oxo-1,4-dihydroquinoline-3-carboxylic acid (**5d**)

White solid; mp: 228–30 °C; yield: 0.26 g, 46%; IR (KBr) νmax: 3317–2831 (OH), 3317,3232 (NH_2_ and NH), 1716 (C=O), 1678 (2C=O overlapped), 1593 (C=C) cm^−1^; ^1^H NMR (400 MHz, DMSO-d_6_): δ 1.08, 1.21 (d, 3H, *J* = 4.0 Hz, CH_3_), 1.45 (t, 3H, *J* = 4.0 Hz, CH_3_), 2.73–2.77 (m, 1H, CH), 2.81–2.97 (m, 2H, CH_2_), 3.04–3.27 (m, 4H, 2CH_2_), 3.31 (s, 2H, CH_2_), 4.59–4.60 (q, 2H, *J* = 4.0 Hz, CH_2_), 7.27 (s, 2H, NH_2_, D_2_O exchangeable), 7.77 (d, 2H, *J* = 8.8 Hz, ArH), 7.85 (d, 2H, *J* = 8.8 Hz, ArH), 7.90 (s, 1H, ArH), 8.93 (d, 1H, ArH, *J* = 8.0 Hz), 10.09 (s, 1H, NH, D_2_O exchangeable) ppm; ^13^C NMR (400 MHz, DMSO-d_6_): δ 16.4, 17.1, 44.5, 48.7, 51.3, 54.3, 55.6, 58.1 (Aliphatic Cs), 107.3, 107.5, 119.5, 120.6, 121.2 (d,* J* = 36.0 Hz), 127.0, 127.6, 139.00, 141.9, 151.5 (d,* J* = 40.0 Hz), 153.7, 156.2 (Aromatic Cs), 165.9 (C=O), 170.3 (C=O), 175.8 (C=O) ppm; MS [m/z, %]: 564.40 [M+1^ך^·^+^, 6.68], 463.43 [M^ך^·^+^, 4.64], 44.99 [100]. Anal. Calcd for C_25_H_27_F_2_N_5_O_6_S (563.57): C, 53.28; H, 4.83; N, 12.43. Found: C, 53.13; H, 4.97; N, 12.70.

##### 1-Ethyl-6,8-difluoro-7-(3-methyl-4-(2-oxo-2-((4-(N-(pyrimidin-2-yl)sulfamoyl)phenyl)amino)ethyl)piperazin-1-yl)-4-oxo-1,4-dihydroquinoline-3-carboxylic acid (**5e**)

White solid; mp: 282–84 °C; yield: 0.20 g, 31%; IR (KBr) νmax: 3456–2854 (OH), 3317, 3332 (NH), 3290 (NH), 1730 (C=O), 1700 (C=O), 1650 (C=O), 1597 (C=C) cm^−1^; ^1^H NMR (400 MHz, DMSO-d_6_): δ 1.03–1.06 (m, 3H, CH_3_), 1.26 (t, 3H, CH_3_), 2.72–2.74 (m, 2H, CH_2_), 2.90–2.92 (m, 1H, CH), 3.52–3.56 (m, 4H, 2CH_2_), 4.71 (s, 2H, CH_2_), 4.94–5.01 (m, 2H, CH_2_), 6.87–6.90 (m, 1H, ArH), 7.20 (d, 2H, *J* = 8.5 Hz, ArH), 7.57 (d, 2H, *J* = 8.5 Hz, ArH), 7.87 (s, 1H, ArH), 8.45–8.46 (m, 1H, ArH), 8.68 (s, 1H, ArH), 8.79–8.80 (m, 1H, ArH), 8.93 (s, 1H, NH, D_2_O exchangeable), 10.25 (s, 1H, NH, D_2_O exchangeable), 10.70 (s, 1H, OH, D_2_O exchangeable) ppm; ^13^C NMR (400 MHz, DMSO-d_6_): δ 7.93, 10.41, 16.41, 45.18, 46.03, 49.34, 54.68, 57.51, 60.68 (Aliphatic Cs), 104.19, 107.74, 111.52, 116.86, 118.32, 119.53 (d,* J* = 80.0 Hz), 123.19, 126.62, 128.03 (d,* J* = 38.0 Hz), 139.47, 140.78, 147.37, 151.70, 153.13, 158.37 (Aromatic Cs), 164.70 (C=O), 165.37 (C=O), 180.21 (C=O) ppm; MS [m/z, %]: 642.45 [M+1^ך^·^+^, 3.48], 641.39 [M^ך^·^+^, 8.64], 85.89 [100]. Anal. Calcd for C_29_H_29_F_2_N_7_O_6_S (641.65): C, 54.28; H, 4.56; N, 15.28. Found: C, 54.37; H, 4.70; N, 15.49.

##### 1-Ethyl-6,8-difluoro-7-(3-methyl-4-(2-((4-(N-(4-methylpyrimidin-2-yl)sulfamoyl)phenyl)amino)-2-oxoethyl)piperazin-1-yl)-4-oxo-1,4-dihydroquinoline-3-carboxylic acid (**5f**)

White solid; mp: 247–49 °C; yield: 0.17 g, 26%; IR (KBr) νmax: 3464–2993 (OH), 3271, 3332 (NH), 3194 (NH), 1740 (C=O), 1720 (C=O), 1680 (C=O), 1593 (C=C) cm^−1^; ^1^H NMR (400 MHz, DMSO-d_6_): δ 0.98–1.02 (m, 3H, CH_3_), 1.26–1.29 (m, 3H, CH_3_), 2.19–2.21 (m, 1H, CH), 2.33–2.39 (m, 2H, CH_2_), 2.47 (s, 3H, CH_3_), 2.60–2.63 (m, 2H, CH_2_), 3.52–3.56 (m, 2H, CH_2_), 4.65 (s, 2H, CH_2_), 4.88–4.94 (m, 2H, CH_2_), 6.78 (d, 2H, *J* = 8.5 Hz, ArH), 7.19 (d, 2H, *J* = 8.5 Hz, ArH), 7.55 (d, 1H, *J* = 8.5 Hz, ArH), 7.60–7.63 (m, 1H, ArH), 7.77–7.78 (m, 1H, ArH), 8.25–8.27 (m, 1H, ArH), 8.93 (s, 1H, NH, D_2_O exchangeable), 10.19 (s, 1H, NH, D_2_O exchangeable) 10.74 (s, 1H, OH, D_2_O exchangeable) ppm; ^13^C NMR (400 MHz, DMSO-d_6_): δ 7.9, 10.5, 16.4, 23.9, 25.0, 45.9, 54.7, 55.3, 56.9, 57.4 (Aliphatic Cs), 107.6, 108.7, 118.3, 119.1 (d,* J* = 32.0 Hz), 126.6, 128.6, 128.9 (d,* J* = 48.0 Hz), 138.5, 139.3, 140.8, 141.3, 150.5, 152.6, 153.9, 157.6, 162.9 (Aromatic Cs), 165.1 (C=O), 167.3 (C=O), 175.4 (C=O) ppm; MS [m/z, %]: 656.78 [M+1^ך^·^+^, 2.25], 655.73 [M^ך^·^+^, 1.81], 387.01 [100]. Anal. Calcd for C_30_H_31_F_2_N_7_O_6_S (655.67): C, 54.95; H, 4.77; N, 14.95. Found: C, 55.12; H, 4.93; N, 15.12.

#### General procedure for the synthesis of compound** 6**

##### 1-Ethyl-6,8-difluoro-7-(4-formyl-3-methylpiperazin-1-yl)-4-oxo-1,4-dihydroquinoline-3-carboxylic acid (**6**)

A solution of lomefloxacin hydrochloride **1** (0.38 g, 0.001 mol) in formamide (5 mL) was heated over a steam bath for 2 h. The reaction mixture was allowed to stand overnight at 5 °C. The formed precipitate was filtered off and recrystallized from methanol to afford compound **5** as a white solid.

White solid; mp: 289–91 °C; yield: 0.19 g, 50%; IR (KBr) νmax: 3024–2588 (OH), 2773 (CH aldehyde), 1732 (C=O), 1662 (C=O), 1612 (C=C) cm^−1^; ^1^H NMR (400 MHz, DMSO-d_6_): δ 1.28, 1.37 (d, 3H, *J* = 6.8 Hz, CH_3_), 1.45 (t, 3H, *J* = 6.8 Hz, CH_3_), 3.20–3.29 (m, 3H, CH_2_ and CH), 3.41–3.63 (m, 4H, 2CH_2_), 4.60 (q, 2H, *J* = 6.8 Hz, CH_2_), 7.89 (d, 1H, *J* = 10.6 Hz, ArH), 8.05, 8.16 (s, 1H, CHO), 8.95 (s, 1H, ArH), 14.74 (s, 1H, OH, D_2_O exchangeable) ppm; ^13^C NMR (400 MHz, DMSO-d_6_): δ 15.3, 16.4, 17.07, 50.1, 50.7, 51.6, 54.3, 55.2 (Aliphatic Cs), 101.0, 107.4, 107.6, 121.6 (d,* J* = 40.0 Hz), 127.9 (d,* J* = 51.0 Hz), 128.4, 134.4, 151.8 (Aromatic Cs), 161.6 (C=O), 165.9 (C=O), 176.0 (C=O) ppm; MS [m/z, %]: 380.09 [M+1^ך^·^+^, 10.75], 378.99 [M^ך^·^+^, 39.55], 334.88 [100]. Anal. Calcd for C_18_H_19_F_2_N_3_O_4_ (379.36): C, 56.99; H, 5.05; N, 11.08. Found: C, 57.12; H, 5.28; N, 11.34.

#### General procedure for the synthesis of compounds** 7a–d**

A mixture of lomefloxacin hydrochloride **1** (0.38 g, 0.001 mol) and triethylamine (0.20 g, 0.002 mol) was stirred in 20 mL dichloromethane till lomefloxacin dissolves completely and then the mixture was cooled in an ice bath to 0 °C. The appropriate benzoyl chloride derivatives (0.001 mol) were dissolved in 10 mL dichloromethane and were added dropwise to the reaction mixture. The reaction mixture was stirred for 6 h. The formed white solid was filtered off and recrystallized from 70% ethanol to give compounds **7a–d**.

##### 7-(4-Benzoyl-3-methylpiperazin-1-yl)-1-ethyl-6,8-difluoro-4-oxo-1,4-dihydroquinoline-3-carboxylic acid (**7a**)

White solid; mp: 290–92 °C; yield: 0.41 g, 91%; IR (KBr) νmax: 3421–2854 (OH), 1732 (C=O), 1660–1640 (2C=O overlapped), 1523 (C=C) cm^−1^; ^1^H NMR (400 MHz, DMSO-d_6_): δ 1.36 (d, 3H, *J* = 6.9 Hz, CH_3_), 1.45 (t, 3H, *J* = 4.0 Hz, CH_3_), 3.40–3.43 (m, 4H, 2CH_2_), 3.45–3.46 (m, 3H, CH_2_ and CH), 4.58–4.60 (q, 2H, *J* = 6.9 Hz, CH_2_), 7.42–7.45 (m, 2H, ArH), 7.47–7.49 (m, 3H, ArH), 7.88 (d, 1H, *J* = 11.7 Hz, ArH), 8.94 (s, 1H, ArH), 14.87 (s, 1H, OH, D_2_O exchangeable) ppm.; ^13^C NMR (400 MHz, DMSO-d_6_): δ 16.4, 35.9, 49.1, 50.9, 54.2, 54.3, 55.4 (Aliphatic Cs), 107.5, 115.3, 121.5, 127.1, 128.9, 129.0, 129.7, 129.9, 133.2, 134.3, 136.6, 151.7 (Aromatic Cs), 166.0 (C=O), 167.8 (C=O), 169.9 (C=O) ppm; MS [m/z, %]: 456.15 [M+1^ך^·^+^, 8.74], 455.15 [M^ך^·^+^, 25.41], 105.05 [100]. Anal. Calcd for C_24_H_23_F_2_N_3_O_4_ (455.45): C, 63.29; H, 5.09; N, 9.23. Found: C, 63.40; H, 5.27; N, 9.44.

##### 7-(4-(4-Bromobenzoyl)-3-methylpiperazin-1-yl)-1-ethyl-6,8-difluoro-4-oxo-1,4-dihydroquinoline-3-carboxylic acid (**7b**)

White solid; mp: 271–73 °C; yield: 0.48 g, 91%; IR (KBr) νmax: 3086–2553 (OH), 1728 (C=O), 1690–1678 (2C=O overlapped), 1554 (C=C) cm^−1^; ^1^H NMR (400 MHz, DMSO-d_6_): δ 1.40 (d, 3H, *J* = 6.8 Hz, CH_3_), 1.50 (t, 3H, *J* = 6.8 Hz, CH_3_), 3.16–3.19 (m, 1H, CH), 3.31–3.33 (m, 4H, 2CH_2_), 3.43–3.46 (m, 2H, CH_2_), 4.38–4.43 (q, 2H, *J* = 7.2 Hz, CH_2_), 7.24 (d, 2H, *J* = 8.0 Hz, ArH), 7.51 (d, 2H, *J* = 8.0 Hz, ArH), 7.92 (dd, 1H, *J* = 9.6 Hz, *J* = 2.0 Hz, ArH), 8.55 (s, 1H, ArH), 14.48 (s, 1H, OH, D_2_O exchangeable) ppm; ^13^C NMR (400 MHz, DMSO-d_6_): δ 15.7, 16.3, 49.3, 51.2, 54.5, 54.7, 55.6 (Aliphatic Cs), 108.4, 108.8, 116.8, 124.2, 128.46, 131.9, 134.0, 134.6, 137.7, 150.2, 156.6, 166.43 (Aromatic Cs), 169.6 (C=O), 173.6 (C=O), 176.2 (C=O) ppm; MS [m/z, %]: 535.10 [M+2^ך^·^+^, 17.00], 534.10 [M+1^ך^·^+^, 6.11], 533.10 [M^ך^·^+^, 16.79], 70.05 [100]. Anal. Calcd for C_24_H_22_BrF_2_N_3_O_4_ (533.08): C, 53.95; H, 4.15; N, 7.86. Found: C, 54.12; H, 4.33; N, 8.09.

##### 7-(4-(4-Chlorobenzoyl)-3-methylpiperazin-1-yl)-1-ethyl-6,8-difluoro-4-oxo-1,4-dihydroquinoline-3-carboxylic acid (**7c**)

White solid; mp: 282–84 °C; yield: 0.43 g, 88%; IR (KBr) νmax: 3271–2553 (OH), 1728 (C=O), 1685–1670 (2C=O overlapped), 1554 (C=C) cm^−1^; ^1^H NMR (400 MHz, DMSO-d_6_): δ 1.40 (d, 3H, *J* = 6.8 Hz, CH_3_), 1.50 (t, 3H, *J* = 6.8 Hz, CH_3_,), 3.16–3.19 (m, 1H, CH), 3.31–3.33 (m, 4H, 2CH_2_), 3.43–3.46 (m, 2H, CH_2_), 4.39–4.42 (q, 2H, *J* = 7.2 Hz, CH_2_), 7.30 (d, 2H,* J* = 8.4 Hz, ArH), 7.35 (d, 2H, *J* = 8.4, ArH), 7.93 (dd, 1H, *J* = 9.6 Hz, *J* = 2.0 Hz, ArH), 8.55 (s, 1H, ArH), 14.47 (s, 1H, OH, D_2_O exchangeable) ppm; ^13^C NMR (400 MHz, DMSO-d_6_): δ 11.6, 14.7, 46.4, 49.8, 49.9, 50.9, 58.0 (Aliphatic Cs), 103.6, 103.8, 104.0, 105.5, 117.7, 123.5, 124.2, 129.4, 131.2, 145.6, 149.34, 161.68 (Aromatic Cs), 164.9 (C=O), 170.1 (C=O), 171.5 (C=O) ppm; MS [m/z, %]: 491.40 [M+1^ך^·^+^, 4.85], 490.40 [M+1^ך^·^+^, 4.5], 489.40 [M^ך^·^+^, 12.69], 70.05 [100]. Anal. Calcd for C_24_H_22_ClF_2_N_3_O_4_ (489.90): C, 58.84; H, 4.53; N, 8.58. Found: C, 58.70; H, 4.72; N, 8.90.

##### 1-Ethyl-6,8-difluoro-7-(3-methyl-4-(4-nitrobenzoyl)piperazin-1-yl)-4-oxo-1,4-dihydroquinoline-3-carboxylic acid (**7d**)

White solid; mp: 215–17 °C; yield: 0.46 g, 92%; IR (KBr) νmax: 3109–2673 (OH), 1728 (C=O), 1693–1680 (2C=O overlapped), 1523 (C=C) cm^−1^; ^1^H NMR (400 MHz, DMSO-d_6_): δ 1.38 (d, 3H, *J* = 4.0 Hz, CH_3_), 1.44 (t, 3H, *J* = 4.0 Hz, CH_3_), 3.34–3.40 (m, 3H, CH_2_ and CH), 3.46–3.49 (m, 4H, 2CH_2_), 4.57–4.59 (q, 2H, *J* = 4.0 Hz, CH_2_), 7.73 (d, 2H, *J* = 8.2 Hz, ArH), 7.87 (d, 1H, *J* = 11.0 Hz, ArH), 8.13 (d, 2H, *J* = 8.2 Hz, ArH), 8.94 (s, 1H, ArH), 14.83 (s, 1H, OH, D_2_O exchangeable) ppm.; ^13^C NMR (400 MHz, DMSO-d_6_): δ 16.4, 26.2, 45.7, 51.0, 52.5, 54.3, 55.50 (Aliphatic Cs), 107.5, 123.9, 124.3, 127.7, 128.5, 130.9, 139.7, 142.9, 148.2, 149.8, 151.7, 165.9 (Aromatic Cs), 166.8 (C=O), 167.9 (C=O), 175.9 (C=O) ppm; MS [m/z, %]: 501.15 [M+1^ך^·^+^, 11.44], 500.15 [M^ך^·^+^, 36.46], 70.05 [100]. Anal. Calcd for C_24_H_22_F_2_N_4_O_6_ (500.45): C, 57.60; H, 4.43; N, 11.20. Found: C, 57.43; H, 4.59; N, 11.37.

### Biological evaluation

#### In vitro anticancer screening

In this study, all the 16 newly synthesized compounds were submitted to the National Cancer Institute (USA) for anticancer evaluation and were selected and evaluated using 60 different human tumor cell lines representing lung, colon, CNS, ovarian, renal, prostate, breast cancers as well as leukemia and melanoma. A single dose (10^–5^ Molar) was used for the initial screening for evaluation of anticancer activity. The 60 human cancer cell lines were provided by the national cancer institute biomedical research campus in Frederick, Maryland.

The most potent 4 compounds were subjected to further testing using 5 dose testing (10^–4^, 10^–5^, 10^–6^, 10^–7^, 10^–8^ Molar) and GI_50_ was determined (The concentration required for 50% inhibition of cell growth).

Doxorubicin and etoposide were used as reference standard potent anticancer drugs which act as topoisomerase II inhibitors.

60 different human cancer cell lines were utilized to screen the newly synthesized compounds for anticancer activity according to the previously reported standard procedure^24–26^ as follows:

1. 96 well microtiter plates were used to inoculate the cells at optical density ranging from 4000–5000 cells/well depending on the replication time of individual cell lines. Roswell Park Memorial Institute culture medium (RPMI 1640 medium) containing 2 mM l-glutamine and fetal bovine serum was used to grow the cells at 37 °C, 5% CO_2_, 95% air and 100% relative humidity for 24 h.
2. Fixation of cells in two plates of each cell line in situ was done using trichloroacetic acid (TCA)*.* This represented the measurement of cell population at the time of drug addition (Tz).
3. Dimethyl sulfoxide (DMSO) was used to dissolve the synthesized compounds at 400-fold the desired final maximum concentration and frozen prior to use. This frozen concentrate was melted and diluted to twice the final concentration with complete medium containing 50 µg/mL gentamycin at time of drug addition.
4. The final compound concentration was obtained by adding 100 µL of the synthesized compounds to the appropriate microtiter wells already containing 100 µL followed by incubation of the plates at 37 °C, 5% CO_2_, 95% air and 100% relative humidity for 48 h.
5. Fixation of the cells was achieved by gentle addition of 50 µL of cold 50% (w/v) TCA followed by incubation for 60 min at 4 °C. The supernatant was then discarded and the plates are washed 5 times with tap water and air dried.
6. Sulphorhodamine B (SRB) solution (100 µL) at 0.4% (w/v) in 1% acetic acid was added to each plate followed by incubation for 10 min at room temperature. The unbound dye was then removed by washing five times with 1% acetic acid and plates were air dried.
7. Dissolution of the bound stain was done using 10 µM trizma base and the absorbance was read at 415 nm wavelength using an automated plate reader.
8. The percentage growth inhibition was calculated as follows:
  $$\left[\frac{\left({\text{Ti}}-{\text{Tz}}\right)}{{\text{C}}-{\text{Tz}}}\right] \times 100 \; for \;concentrations \;which \;Ti\ge Tz$$
  $$\left[\frac{\left({\text{Ti}}-{\text{Tz}}\right)}{{\text{Tz}}}\right] \times 100 \; for \;concentrations \; which \;Ti<Tz$$

- Time zero (Tz)
- Control growth (C)
- Test growth in the presence of drug (Ti)

For IC_50_ determination a serial dilution was made in order to obtain the desired concentrations (10^–4^, 10^–5^, 10^–6^, 10^–7^, 10^–8^ Molar).

#### Cell cycle analysis

The effect of compound **3e** on cell cycle progression was analyzed by flow cytometric analysis using the following procedure:

1. Leukemia SR cells were treated with compound **3e** at concentration of IC_50_ 1.90 µM and incubated for 24 h. The cells were washed twice using Ice-cold phosphate buffer saline (PBS).
2. Centrifugation was used to collect the cells, then the cells were fixed in 70% (v/v) ethanol, washed with PBS and re-suspended with 0.10 mg/mL RNase.
3. After that, cells were stained with 40 mg/mL propidium iodide (PI) and evaluated by flow cytometry using FACSCalibur (Becton Dickinson). Cell cycle distributions was calculated by Cell Quest software (Becton Dickinson) and the results are reported in Table 3^27^.

**Table 3:** Cell cycle analysis of control and compound **3e** for leukemia SR cells.

Cell cycle phase	Percentage of apoptotic cells			
	%G0-G1	%S	%G2-M	%Pre-G1
**3e**	24.66	26.08	49.26	26.19
Doxorubicin	25.18	28.76	46.06	22.17
Control	38.31	56.44	5.25	2.57

#### Determination of apoptosis using Annexin-V

Annexin-V-FITC and propidium iodide was used to evaluate the pro-apoptotic effect of compounds **3e** using the following procedure:

1. IC_50_ (1.90 µM) of compound **3e** was added to Leukemia SR cells and the cells were incubated for 24 h after treatment.
2. The cells were then collected by trypsinization and 0.5 × 10^6^ cells were washed twice with PBS and stained with 5 µL annexin-V-FITC and 5 µL PI in 1 × binding buffer for 15 min at room temperature in the dark.
3. FACS Calibur flow cytometer (BD Biosciences, San Jose, CA was used for analysis of apoptosis and the results were reported in Table 4.

**Table 4:** Apoptosis results of control and compound **3e** for leukemia SR cells.

	Apoptosis			Necrosis
	%Total	%Early	%Late	
**3e**	26.19	5.27	18.20	2.72
Doxorubicin	22.17	5.88	12.21	4.08
Control	2.57	1.02	0.67	0.88

#### Topoisomerase II inhibitory activity

Compound **3a,b,c,e** were evaluated for topoisomerase IIα inhibitory activity utilizing the human DNA topoisomerase ELISA kit (MBS2885049) and following the provided procedure:

1. Standards and the tested compound were dissolved in sample diluent and tenfold serial dilution was achieved.
2. Biotin-conjugated antibody and avidin conjugated Horseradish Peroxidase (HRP-avidin) were diluted to tenfolds.
3. 100 µL of each concentration of standard or test compounds were added to each well and incubated at 37 °C for 1 h.
4. Removal of the liquid in each well was done.
5. To each well 100 µL of Biotin-conjugated antibody solution was added followed by incubation for 1 h at 37 °C.
6. The microtiter plate was allowed to aspirate and washed 3 times.
7. 100 µL of avidin conjugated Horseradish Peroxidase (HRP-avidin) solution was added to each well and incubatation for 1 h at 37 °C was achieved.
8. The plate was aspirated and washed 5 times.
9. Addition of 90 µL of 3,3′,5,5′-tetramethylbenzidine (TMB) substrate to each well followed by plate incubated for 30 min at 37 °C and protection from light was done.
10. 50 µL stop solution was added. The absorbance was measured spectrophotometrically within 5 min at 450 nm the results are reported in Table 5.

**Table 5:** Topoisomeraese IIα IC_50_ for compounds **3a,b,c,e in µM.**

Compounds	Topoisomerase IIα
**3a**	1.74
**3b**	2.41
**3c**	1.68
**3e**	0.98
Doxorubicin	0.61

#### Molecular modeling study

Molecular Operating Environment (MOE, 10.2008) software^23^ was used to carry out the molecular modeling studies. All minimizations were done with MOE with MMFF94x force field and the formal and partial charges were automatically calculated. The protein data bank^28^ was utilized to download the x-ray crystallographic structures of topoisomerase IIα co-crystallized with DNA^20^. Topoisomerase IIα was prepared was prepared for docking study by removal of DNA chains, water molecules. Protonate 3D protocol in MOE with default options was used to prepare the topoisomerase enzyme. Triangle matcher placement method and London dG scoring function were used for the docking protocol. Docking of the reference topoisomerase inhibitor merbarone was done first and compared to a previously reported study in order to validate the procedure^21^. The MOE validated setup was utilized in order to predict the binding interactions and affinity of the synthesized compounds at the active site. Reference compound merbarone was used to compare its binding score and binding interactions with compound **3e**.

### Supplementary Information

Supplementary Information 1.

Supplementary Information 2.

Supplementary Information 3.

Supplementary Information 4.

Supplementary Information 5.

Supplementary Information 6.

Supplementary Information 7.

Supplementary Information 8.

Supplementary Information 9.

Supplementary Information 10.

Supplementary Information 11.

Supplementary Information 12.

Supplementary Information 13.

Supplementary Information 14.

Supplementary Information 15.

Supplementary Information 16.

Supplementary Information 17.

Supplementary Information 18.

Supplementary Information 19.

Supplementary Information 20.

Supplementary Information 21.

## Data Availability

All data generated or analyzed during this study are included in this published article and its supplementary information files.
