# Supplementary figures and images for: Design, synthesis and in vitro anticancer activity of some new lomefloxacin derivatives
Source: Sci Rep. 2024 Mar 14;14:6175. doi: 10.1038/s41598-024-56313-w (PMC10940605; doi:10.1038/s41598-024-56313-w)

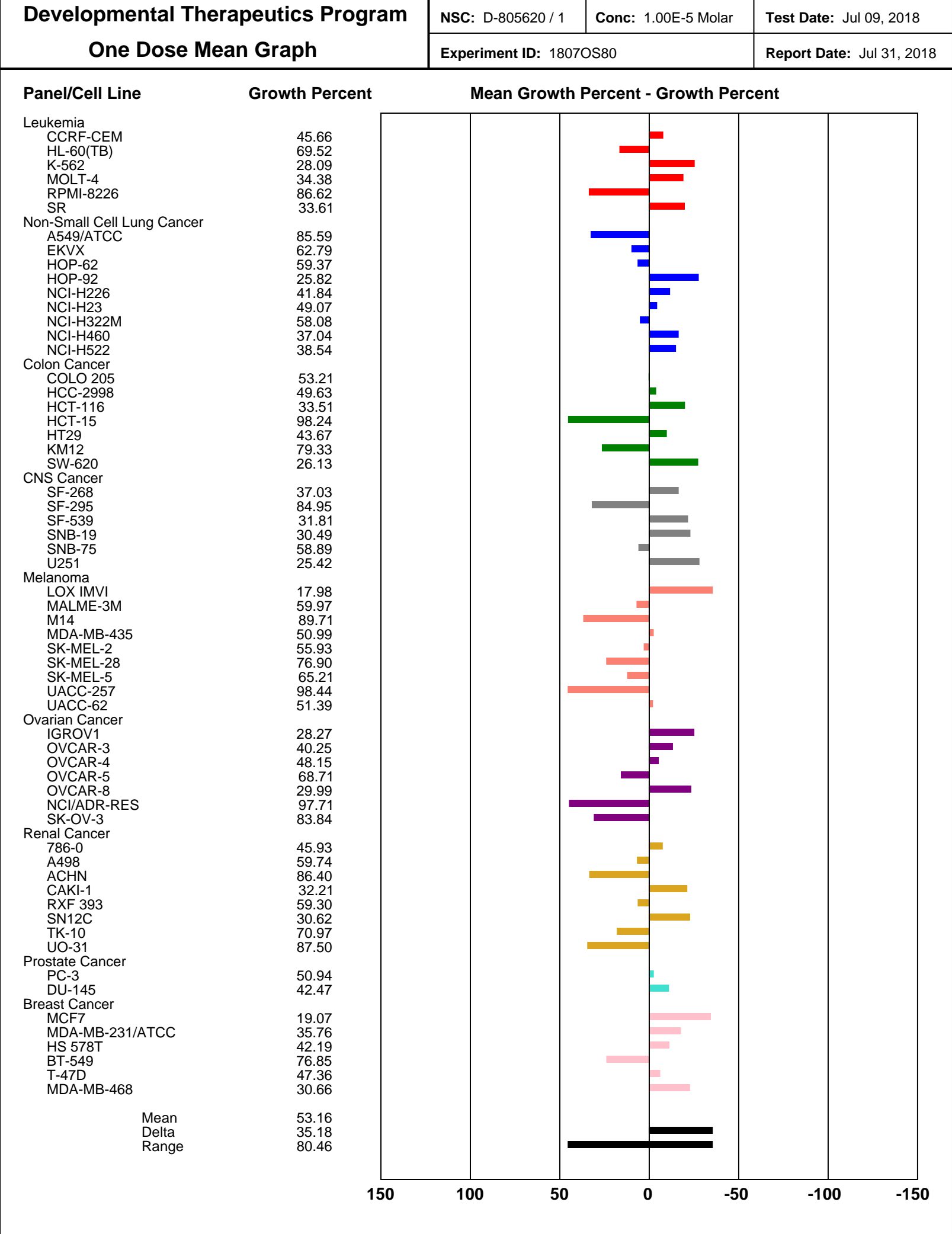

Supplement: Supplementary file 1 — Supplementary Information 1. [file 41598_2024_56313_MOESM1_ESM.pdf]

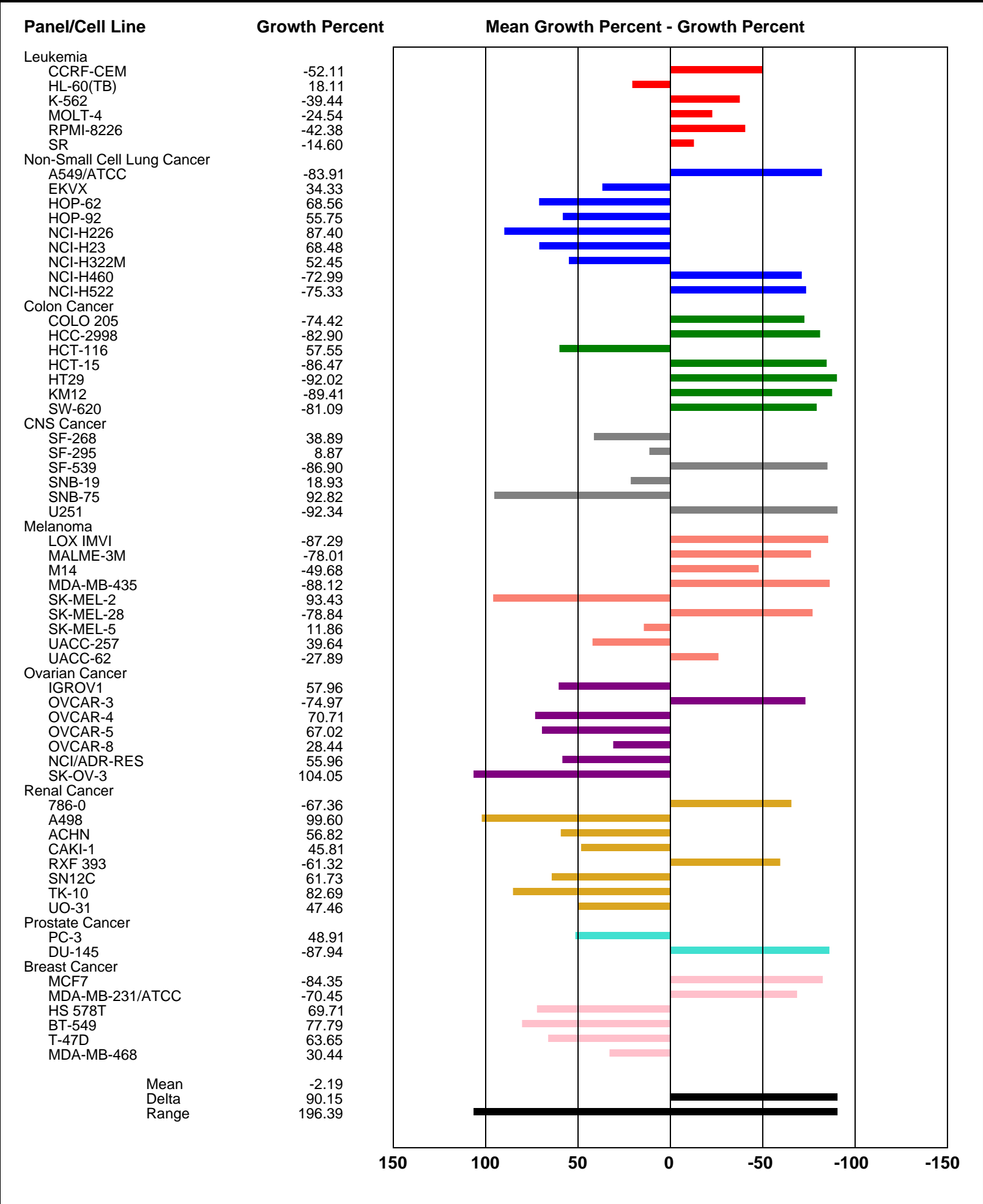

Supplement: Supplementary file 3 — Supplementary Information 3. [file 41598_2024_56313_MOESM3_ESM.pdf]

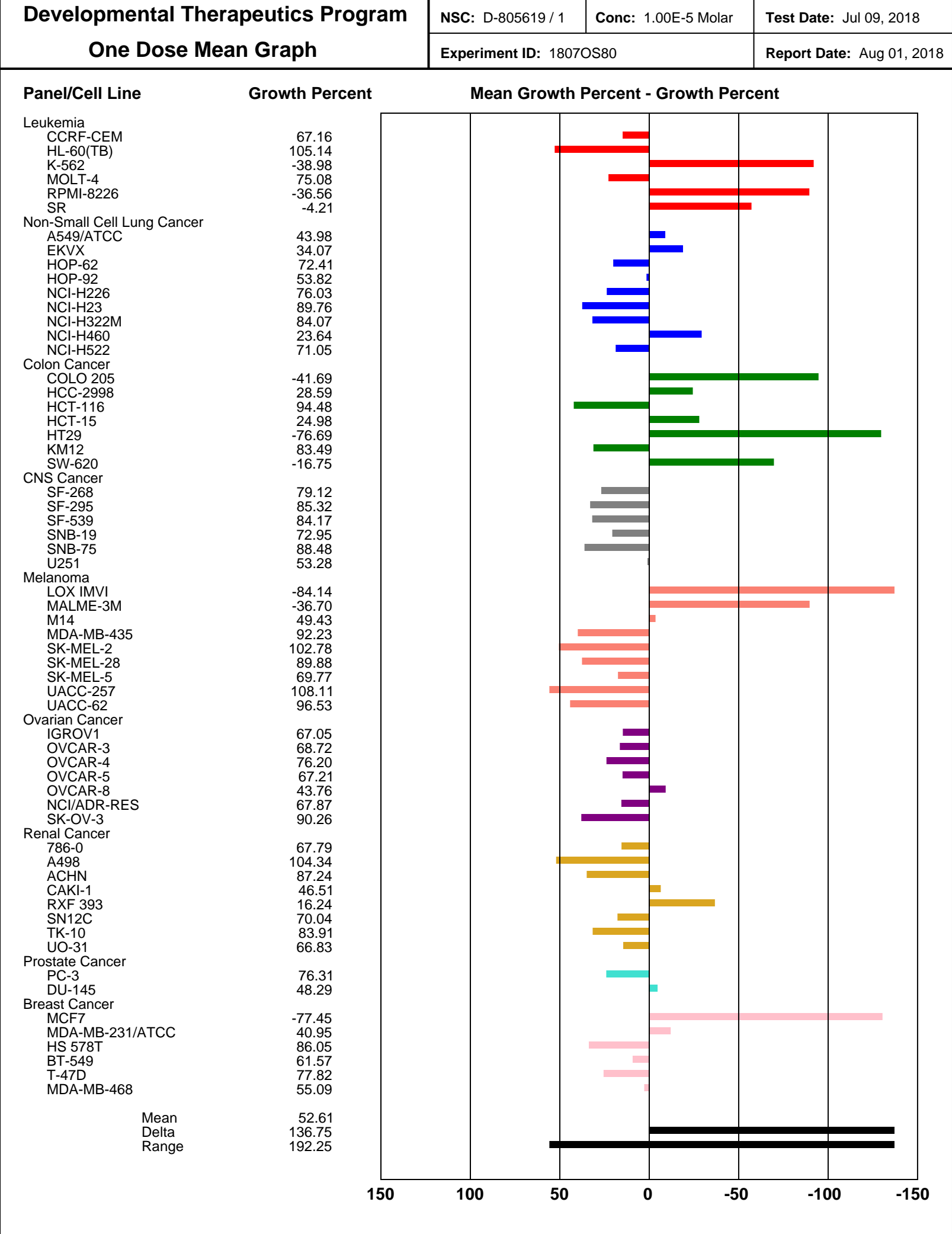

Supplement: Supplementary file 7 — Supplementary Information 7. [file 41598_2024_56313_MOESM7_ESM.pdf]

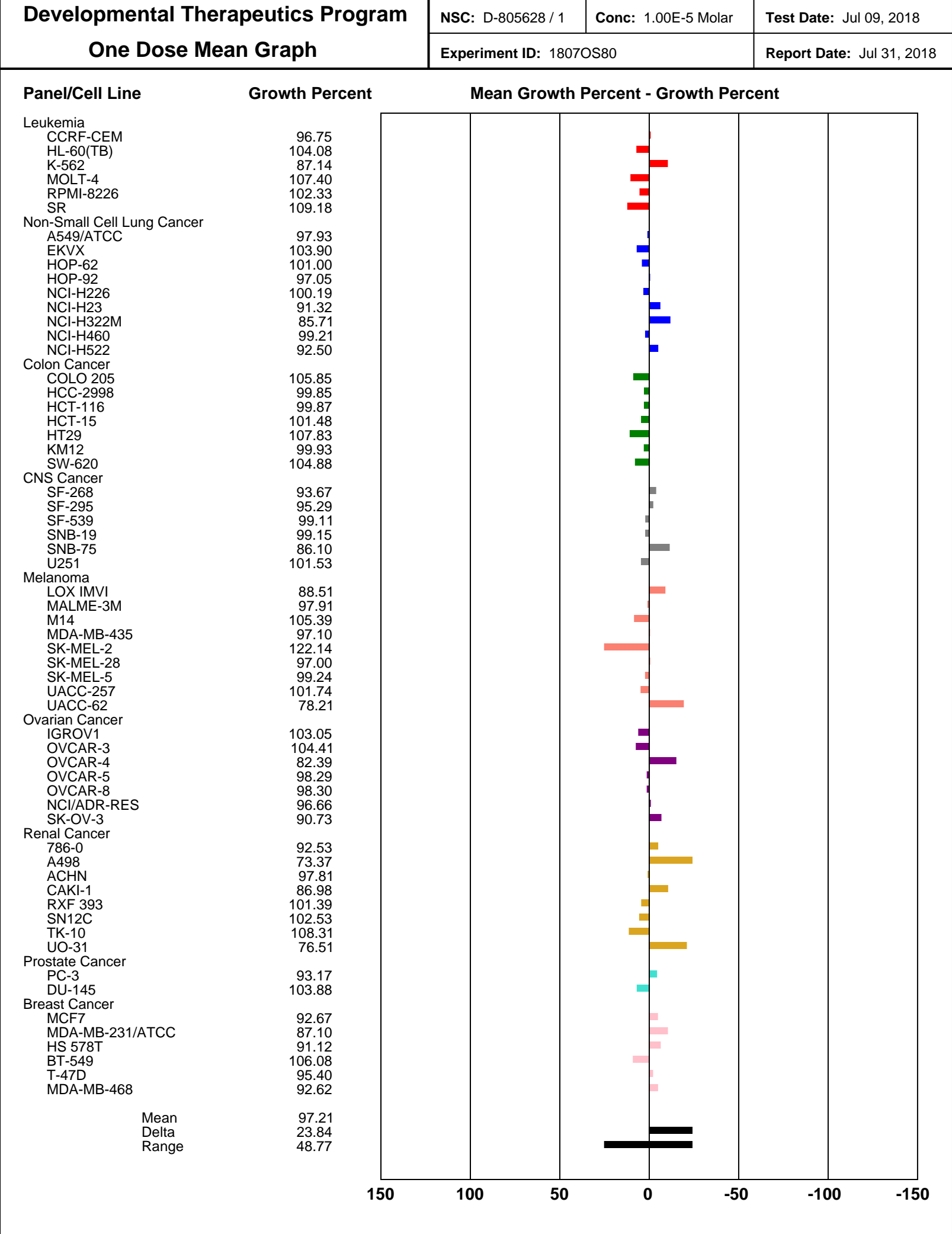

Supplement: Supplementary file 8 — Supplementary Information 8. [file 41598_2024_56313_MOESM8_ESM.pdf]

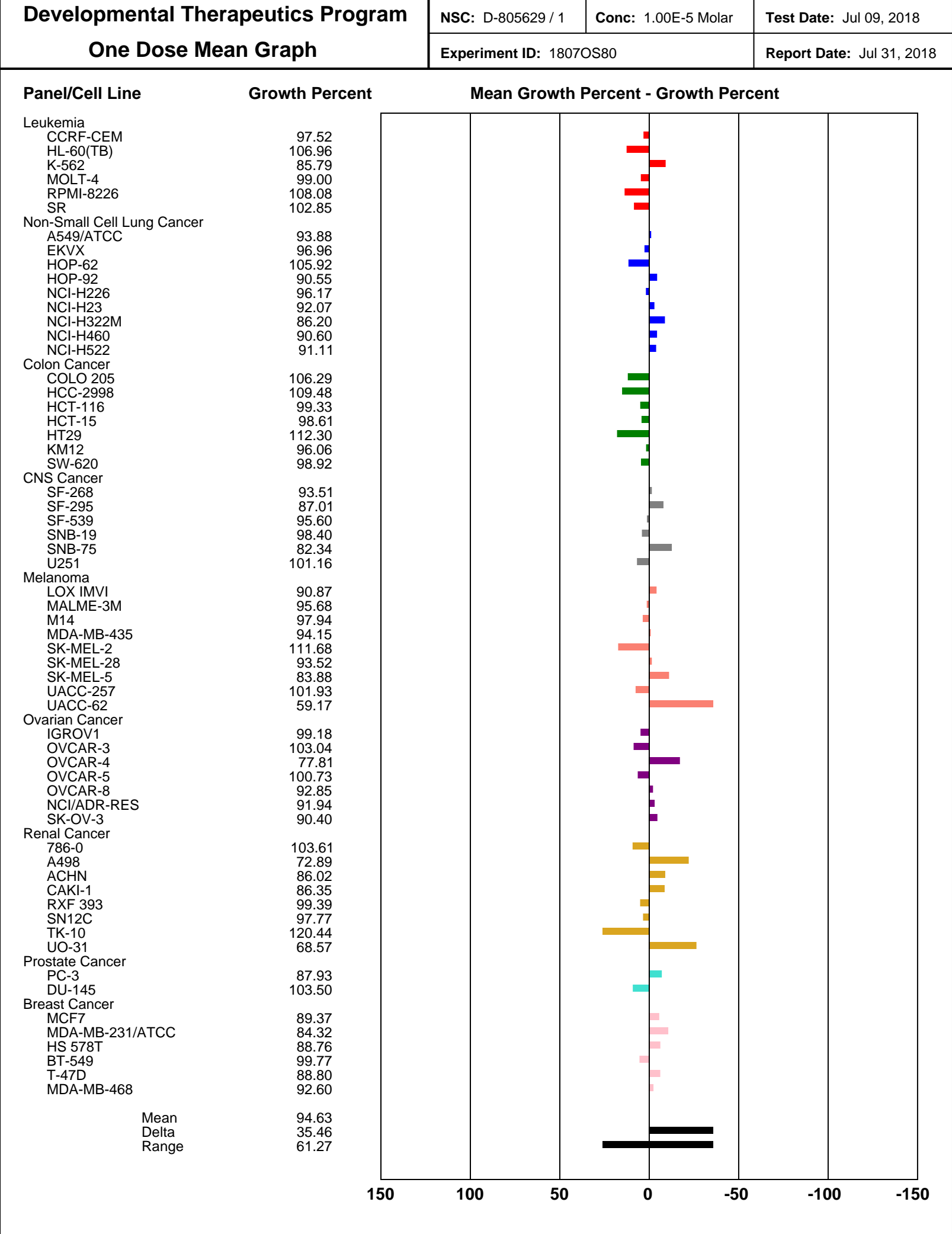

Supplement: Supplementary file 9 — Supplementary Information 9. [file 41598_2024_56313_MOESM9_ESM.pdf]

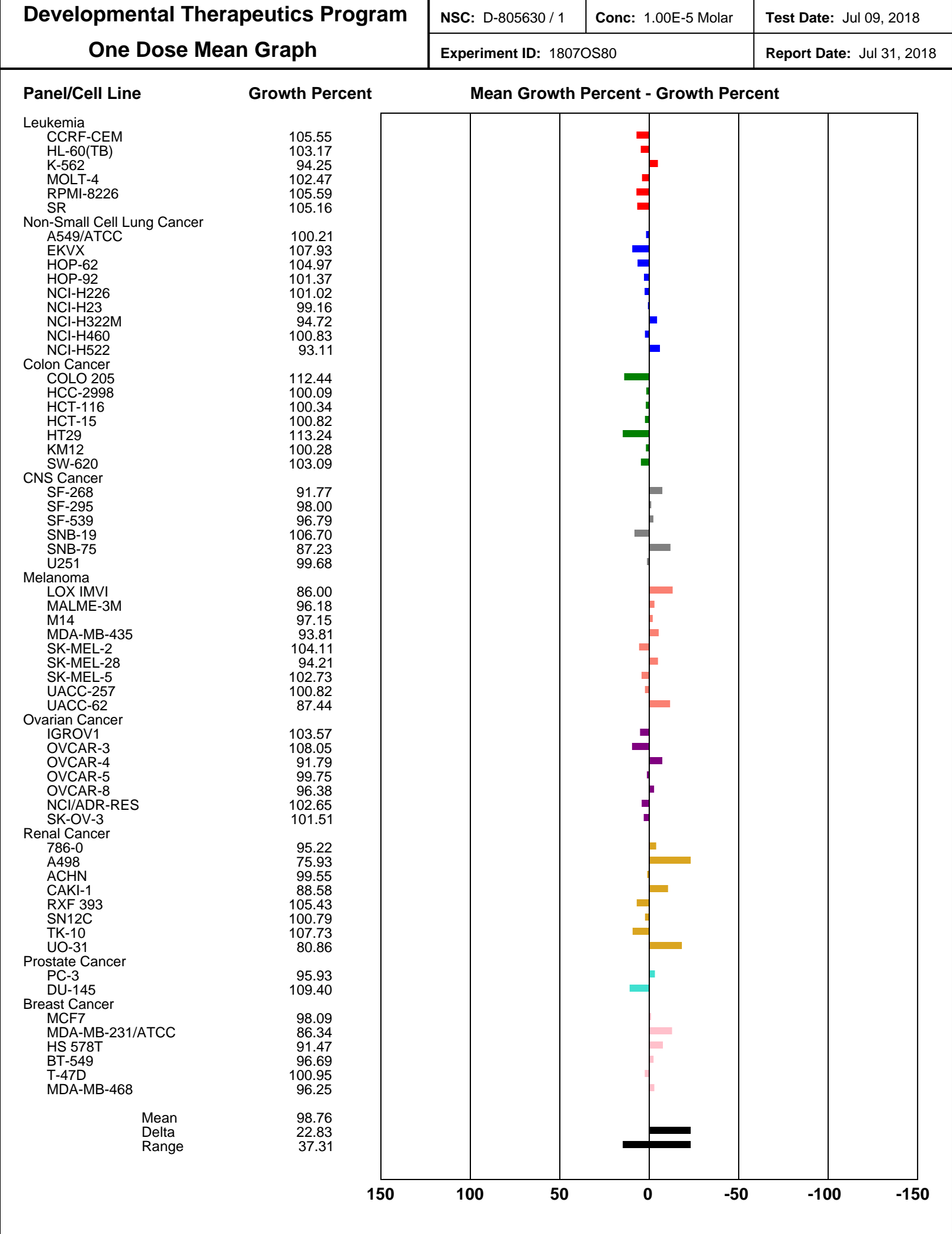

Supplement: Supplementary file 10 — Supplementary Information 10. [file 41598_2024_56313_MOESM10_ESM.pdf]

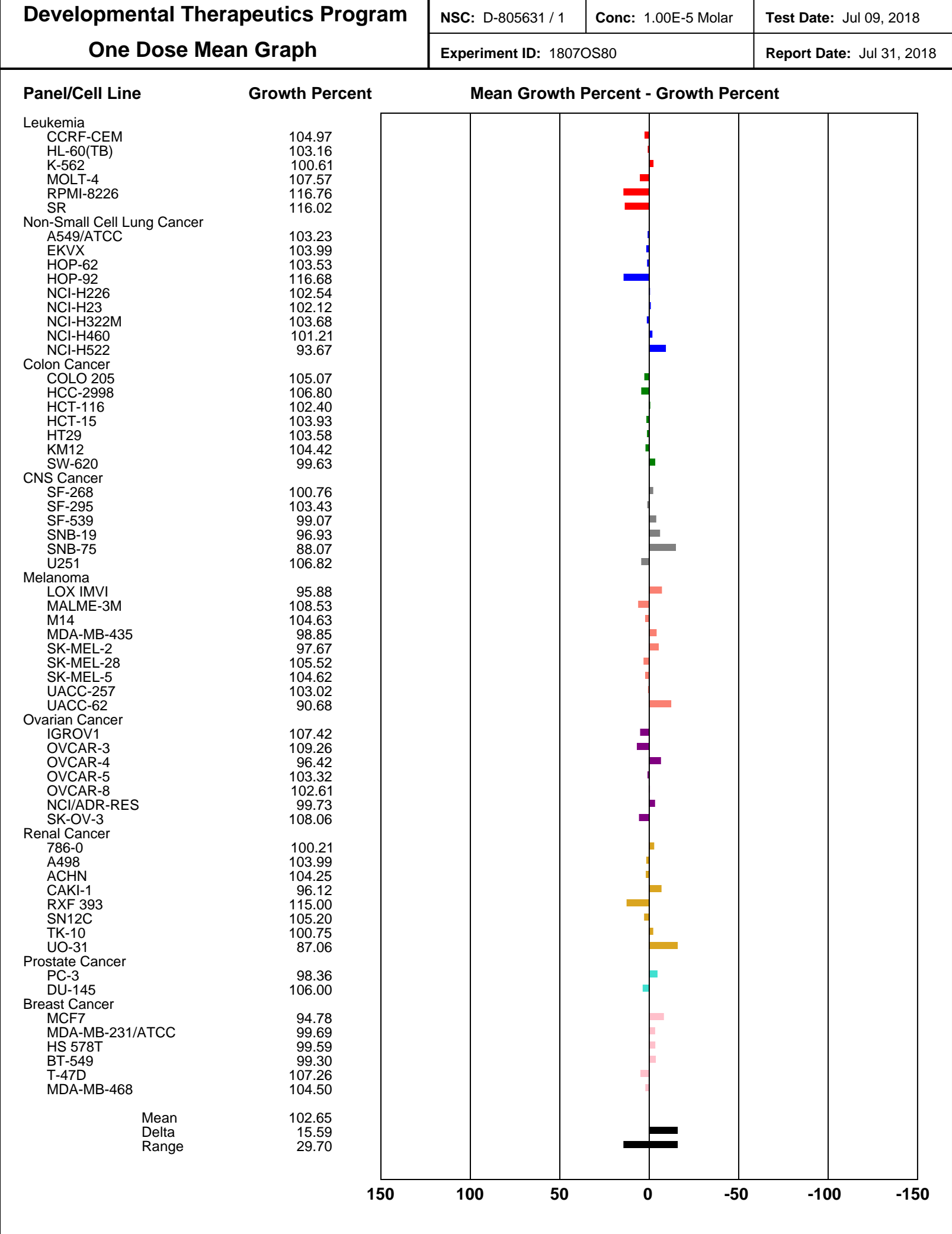

Supplement: Supplementary file 11 — Supplementary Information 11. [file 41598_2024_56313_MOESM11_ESM.pdf]

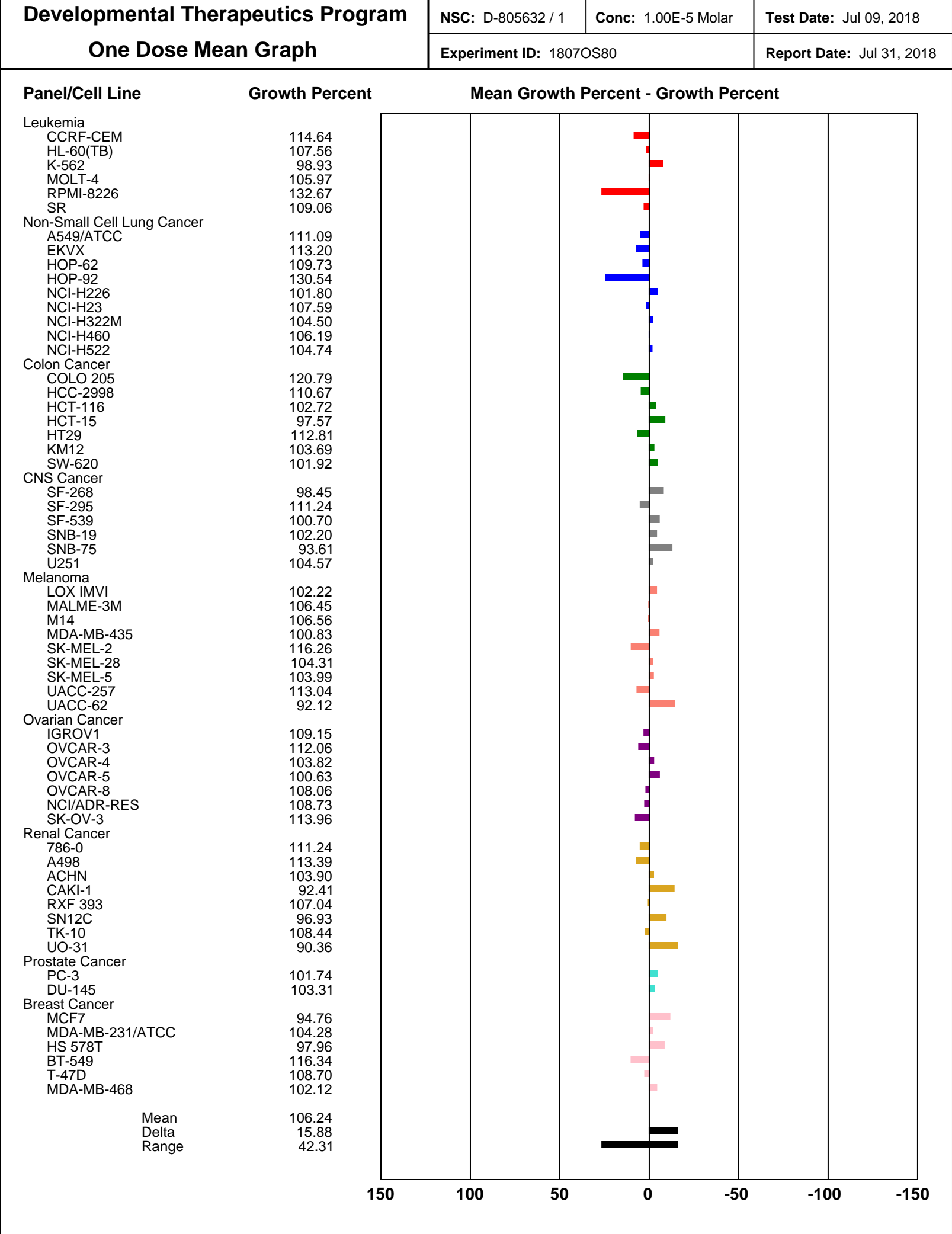

Supplement: Supplementary file 12 — Supplementary Information 12. [file 41598_2024_56313_MOESM12_ESM.pdf]

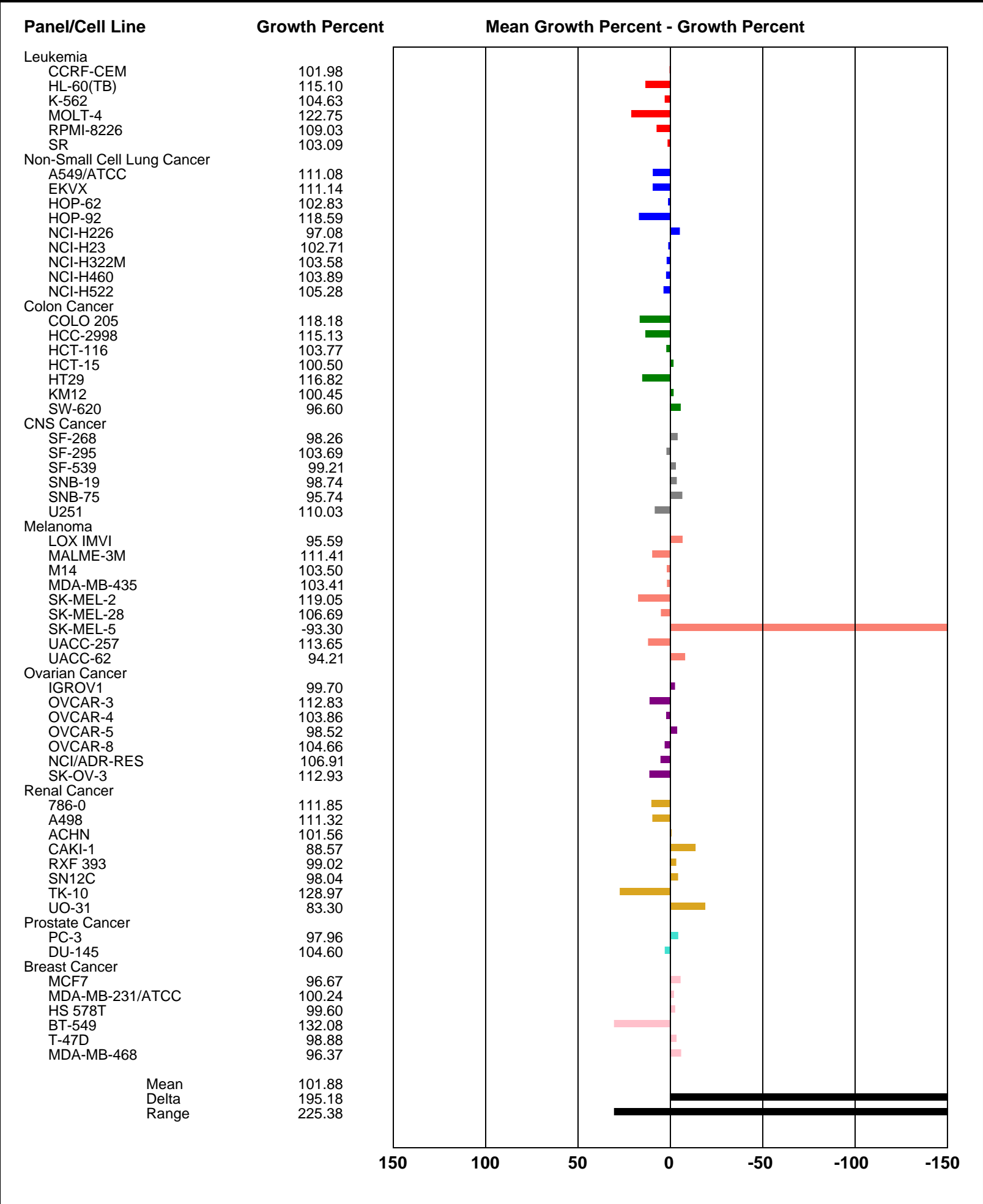

Supplement: Supplementary file 13 — Supplementary Information 13. [file 41598_2024_56313_MOESM13_ESM.pdf]

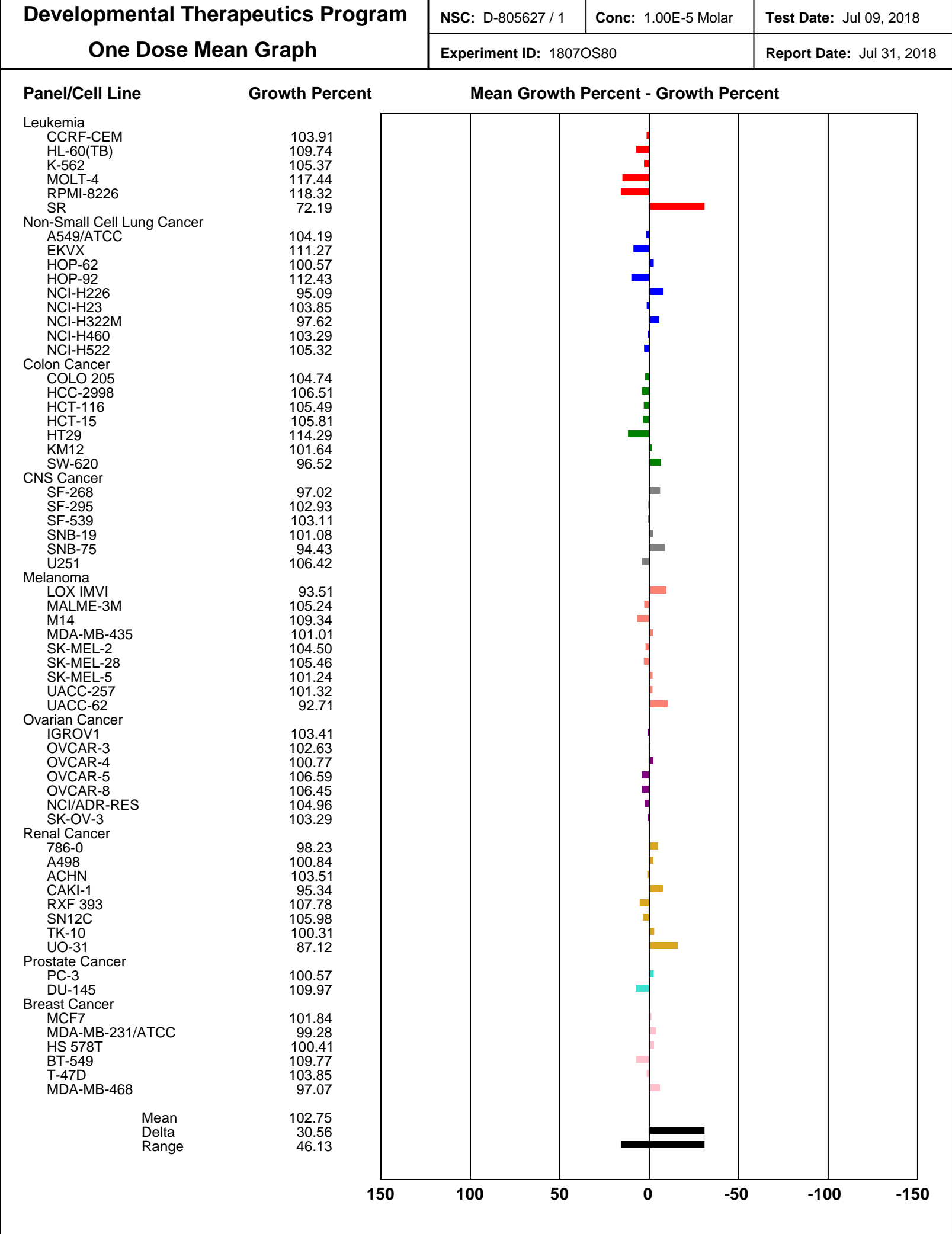

Supplement: Supplementary file 14 — Supplementary Information 14. [file 41598_2024_56313_MOESM14_ESM.pdf]

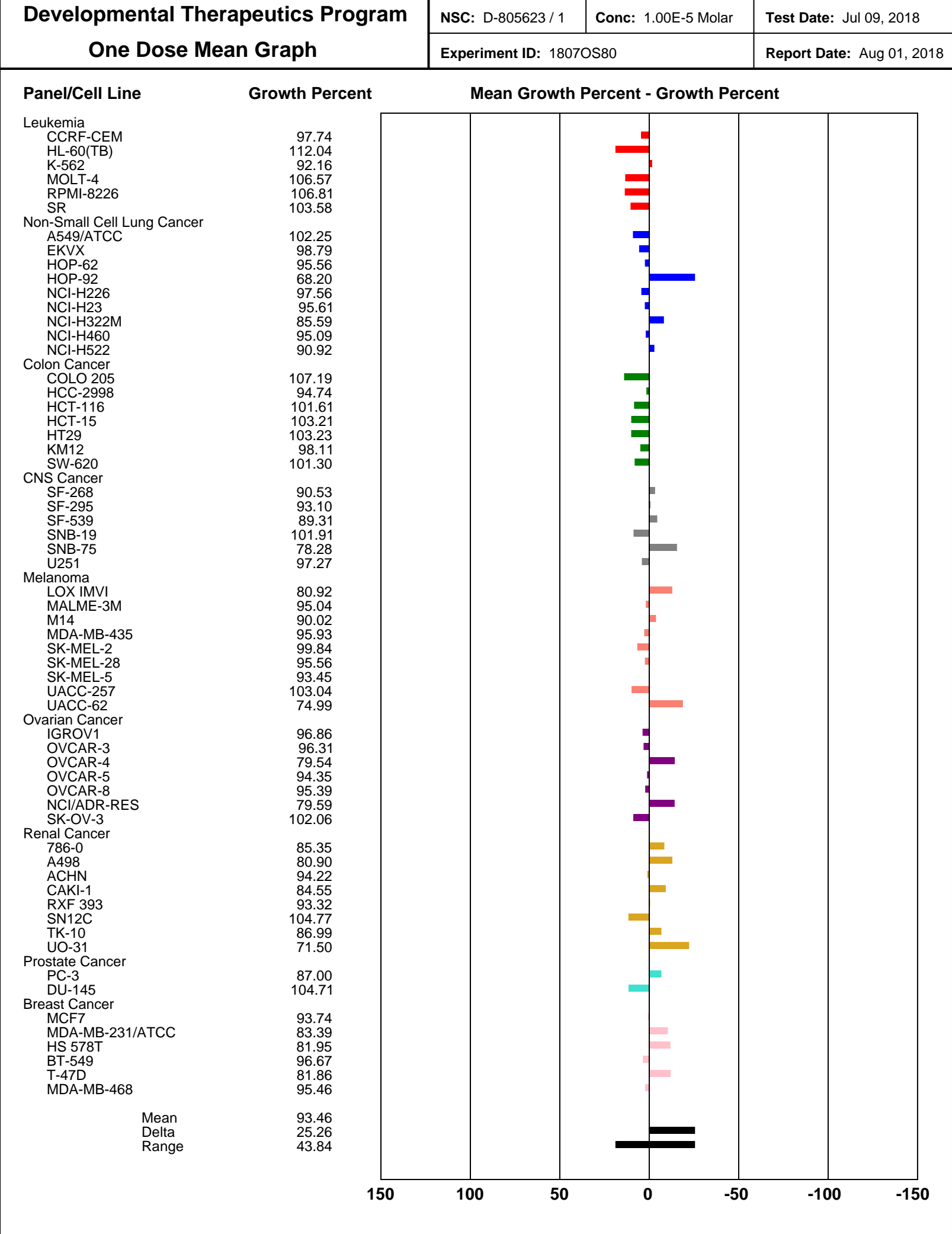

Supplement: Supplementary file 15 — Supplementary Information 15. [file 41598_2024_56313_MOESM15_ESM.pdf]

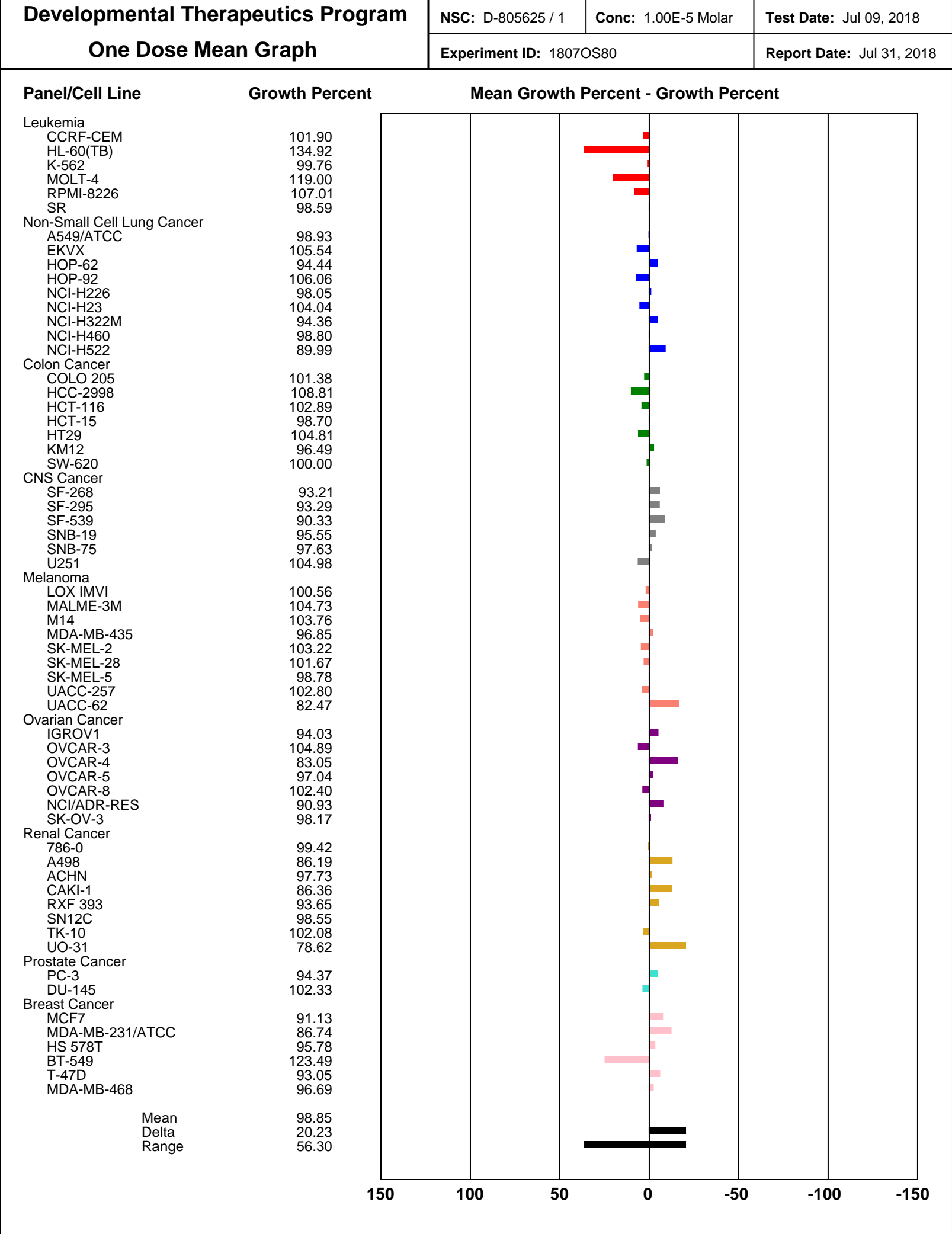

Supplement: Supplementary file 16 — Supplementary Information 16. [file 41598_2024_56313_MOESM16_ESM.pdf]

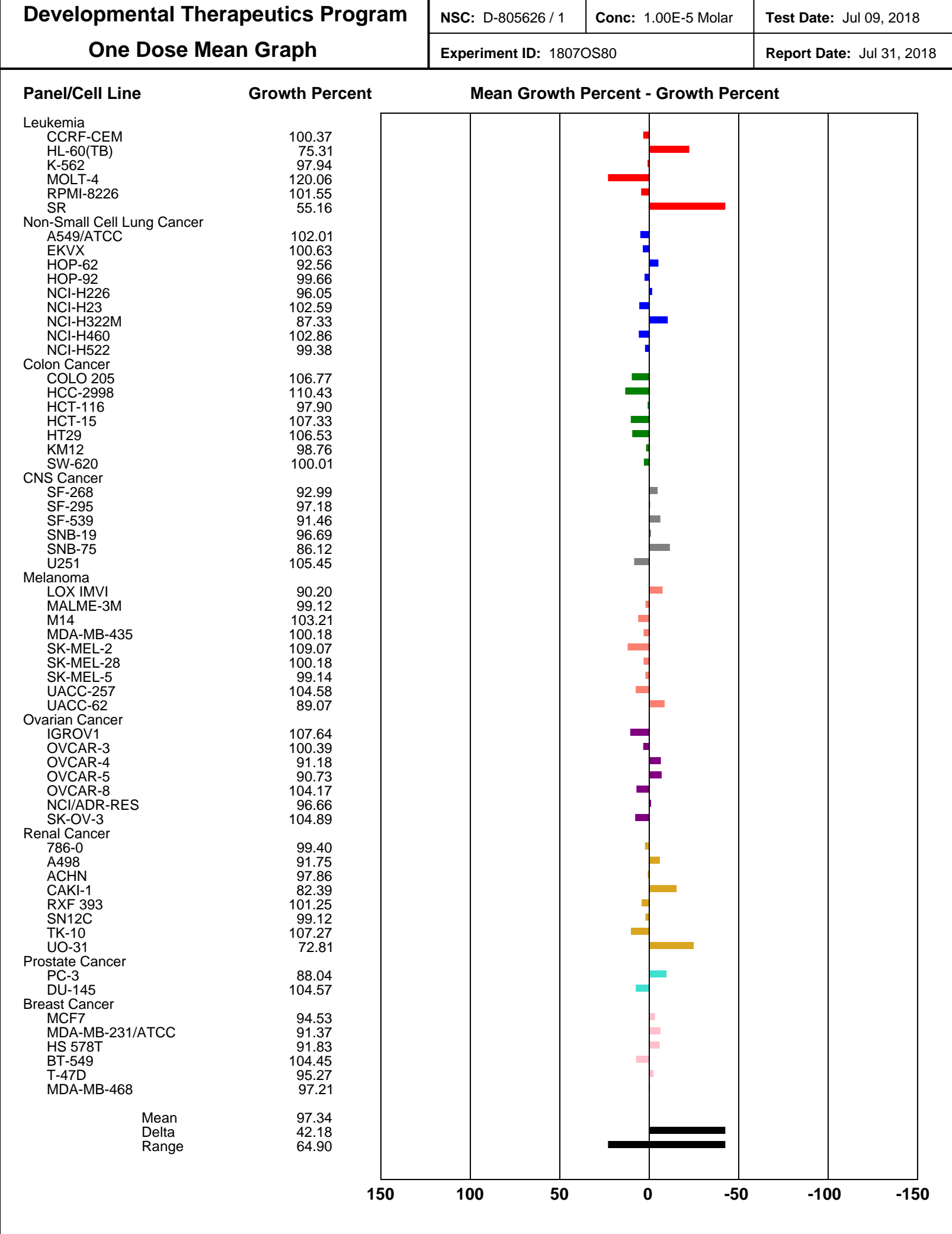

Supplement: Supplementary file 17 — Supplementary Information 17. [file 41598_2024_56313_MOESM17_ESM.pdf]

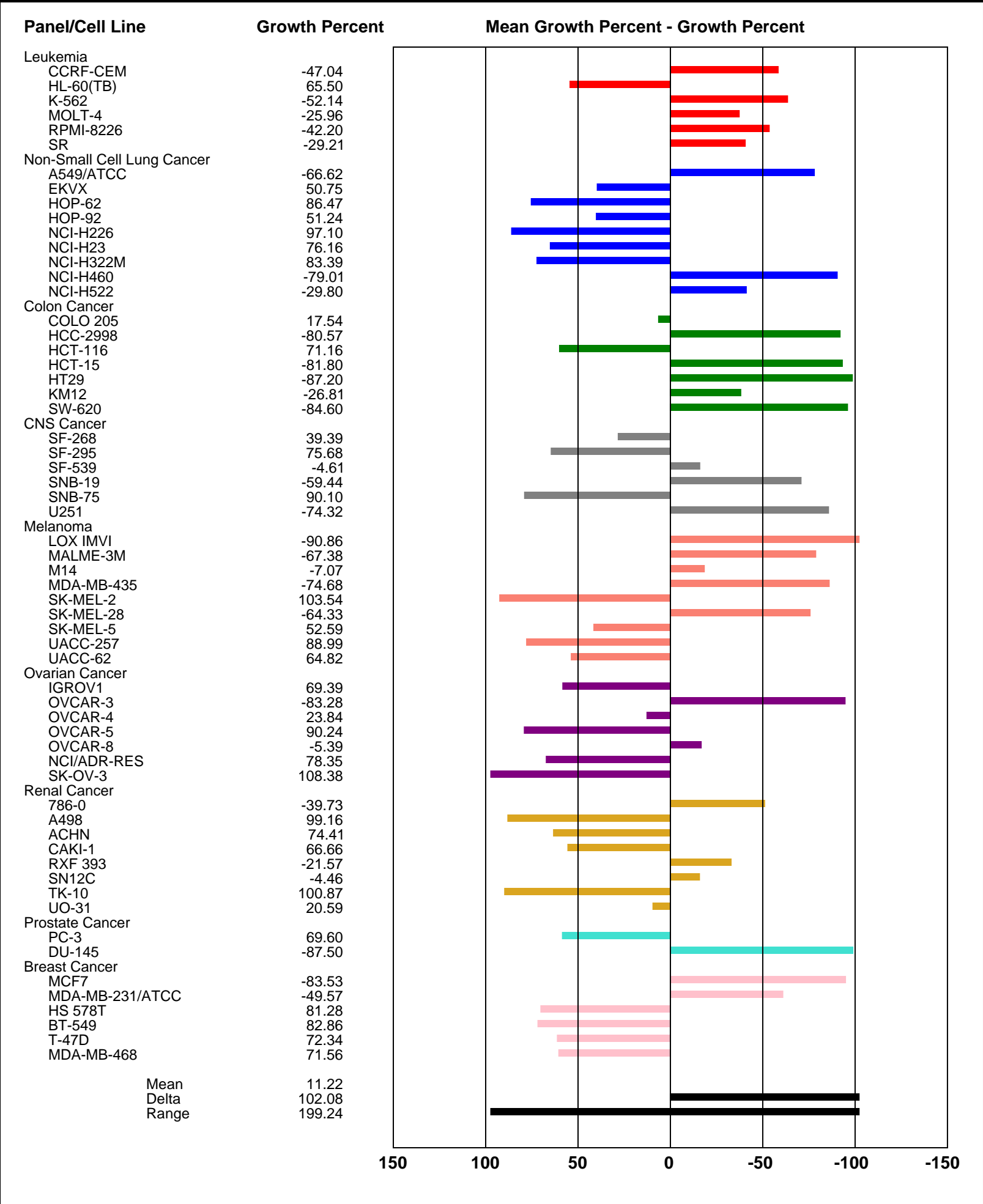

Supplement: Supplementary file 18 — Supplementary Information 18. [file 41598_2024_56313_MOESM18_ESM.pdf]

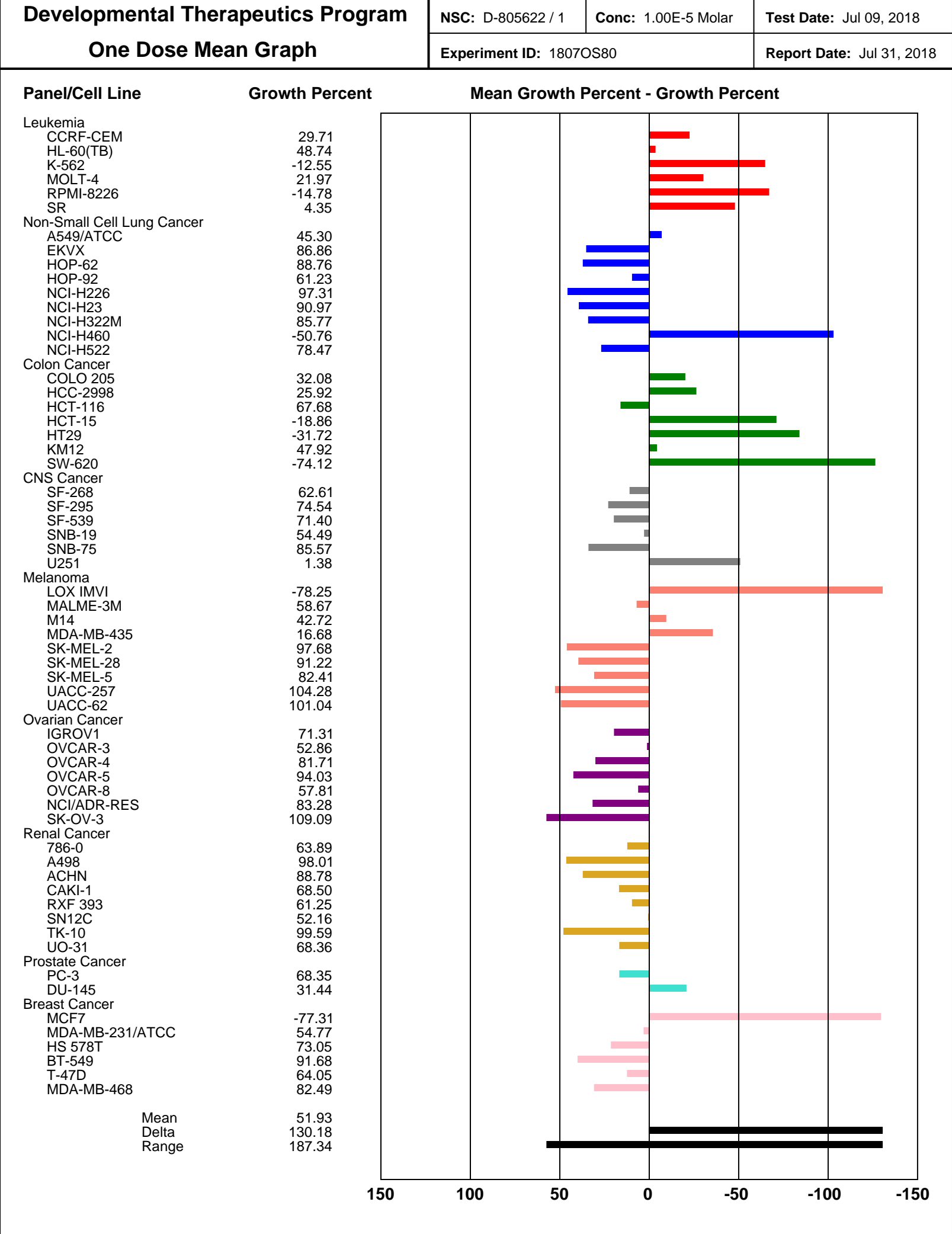

Supplement: Supplementary file 19 — Supplementary Information 19. [file 41598_2024_56313_MOESM19_ESM.pdf]

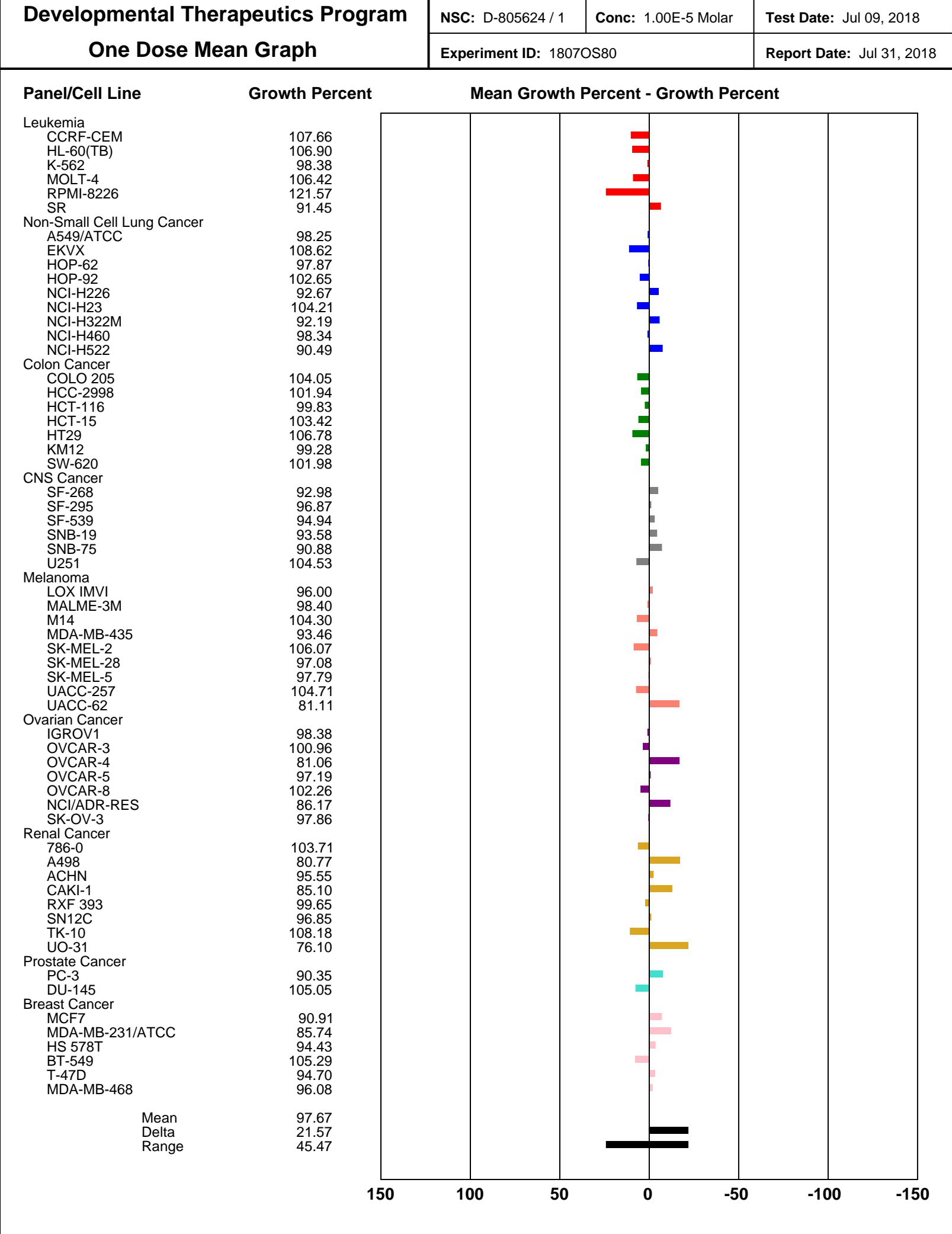

Supplement: Supplementary file 20 — Supplementary Information 20. [file 41598_2024_56313_MOESM20_ESM.pdf]
