# Supplementary material for: Design, synthesis and in vitro anticancer activity of some new lomefloxacin derivatives
Source: Sci Rep. 2024 Mar 14;14:6175. doi: 10.1038/s41598-024-56313-w (PMC10940605; doi:10.1038/s41598-024-56313-w)

National Cancer Institute Developmental Therapeutics Program
Dose Response Curves

NSC: D - 805618 / 1	SSPL: 0YYN	EXP. ID: 1808NS93
Report Date: October 03, 2018	Test Date: August 27, 2018	

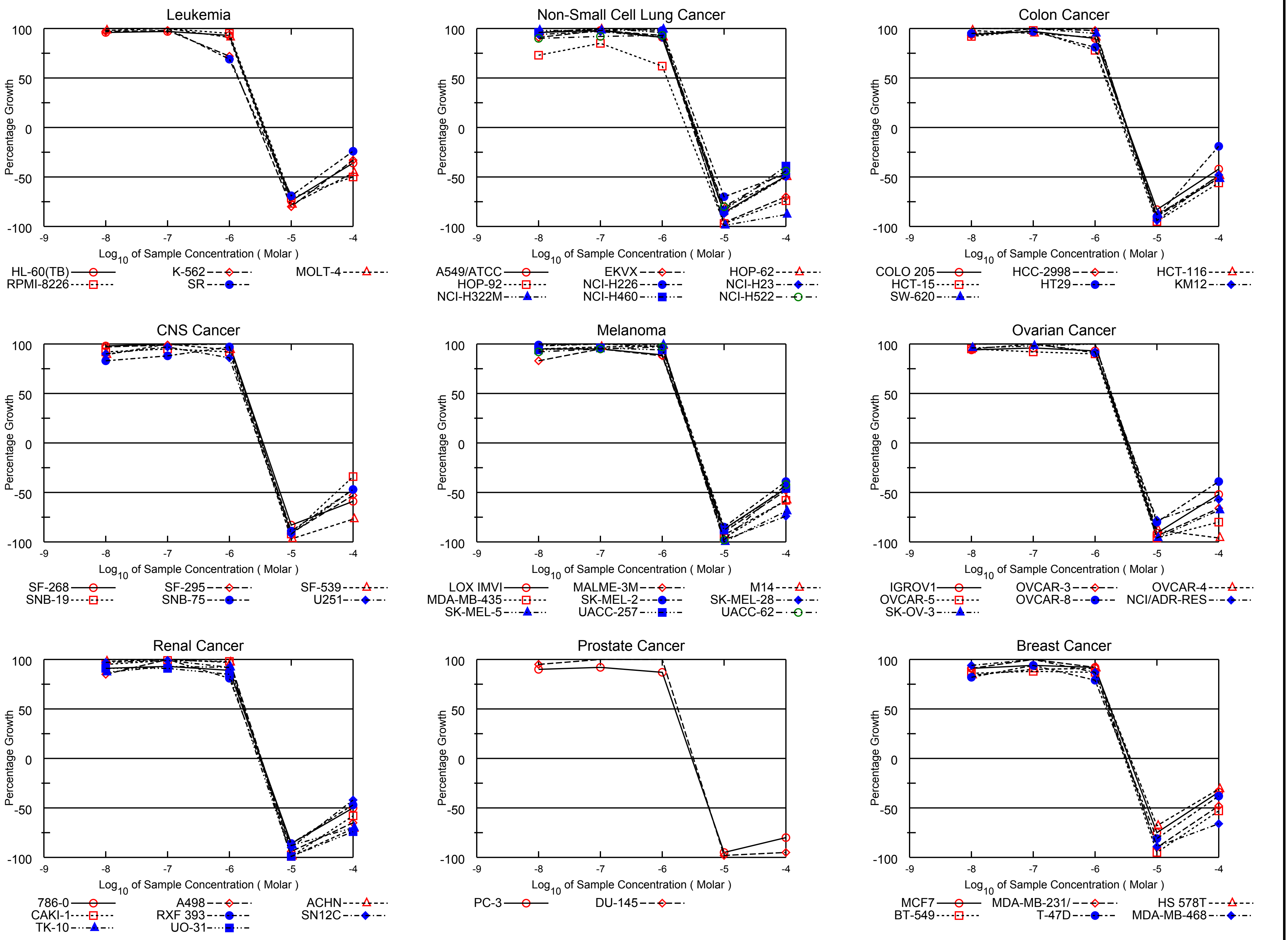

National Cancer Institute Developmental Therapeutics Program
In-Vitro Testing Results

NSC : D - 805618 / 1	Experiment ID : 1808NS93	Test Type : 08	Units : Molar
Report Date : October 03, 2018	Test Date : August 27, 2018	QNS :	MC :
COMI : L1a	Stain Reagent : SRB Dual-Pass Related	SSPL : 0YYN	

			Log10 Concentration													
	Time		Mean Optical Densities						Percent Growth							
Panel/Cell Line	Zero	Ctrl	-8.0	-7.0	-6.0	-5.0	-4.0	-8.0	-7.0	-6.0	-5.0	-4.0	GI50	TGI	LC50
**Leukemia**															
HL-60(TB)	0.832	3.187	3.083	3.122	3.023	0.214	0.535	96	97	93	-74	-36	1.81E-6	3.60E-6	.
K-562	0.235	2.493	2.417	2.443	1.855	0.048	0.158	97	98	72	-80	-33	1.39E-6	2.98E-6	.
MOLT-4	0.643	2.854	2.820	2.860	2.656	0.141	0.347	98	100	91	-78	-46	1.75E-6	3.45E-6	.
RPMI-8226	0.879	2.482	2.505	2.530	2.394	0.258	0.443	101	103	95	-71	-50	1.86E-6	3.73E-6	.
SR	0.315	1.077	1.132	1.075	0.843	0.097	0.238	107	100	69	-69	-24	1.38E-6	3.16E-6	.
**Non-Small Cell Lung Cancer**															
A549/ATCC	0.404	2.209	2.142	2.166	2.045	0.056	0.206	96	98	91	-86	-49	1.70E-6	3.26E-6	.
EKVX	1.068	2.714	2.567	2.675	2.593	0.040	0.316	91	98	93	-96	-70	1.68E-6	3.09E-6	5.69E-6
HOP-62	0.602	1.944	1.888	1.933	1.820	0.112	0.303	96	99	91	-81	-50	1.72E-6	3.37E-6	.
HOP-92	1.285	1.938	1.761	1.838	1.689	0.043	0.337	73	85	62	-97	-74	1.19E-6	2.46E-6	5.08E-6
NCI-H226	0.903	2.364	2.304	2.390	2.336	0.270	0.478	96	102	98	-70	-47	1.93E-6	3.83E-6	.
NCI-H23	0.741	2.440	2.330	2.384	2.288	0.094	0.374	94	97	91	-87	-50	1.70E-6	3.24E-6	.
NCI-H322M	0.748	2.189	2.161	2.163	2.181	0.010	0.092	98	98	99	-99	-88	1.78E-6	3.17E-6	5.68E-6
NCI-H460	0.265	2.610	2.524	2.657	2.514	0.043	0.161	96	102	96	-84	-39	1.80E-6	3.42E-6	.
NCI-H522	0.801	2.516	2.337	2.373	2.393	0.157	0.447	90	92	93	-80	-44	1.77E-6	3.43E-6	.
**Colon Cancer**															
COLO 205	0.418	1.951	1.854	1.902	1.802	0.072	0.243	94	97	90	-83	-42	1.71E-6	3.32E-6	.
HCC-2998	0.806	2.724	2.587	2.724	2.681	0.081	0.396	93	100	98	-90	-51	1.80E-6	3.32E-6	6.13E-6
HCT-116	0.223	2.203	2.154	2.097	2.022	0.025	0.114	98	95	91	-89	-49	1.69E-6	3.20E-6	.
HCT-15	0.273	1.770	1.650	1.736	1.444	0.013	0.119	92	98	78	-95	-56	1.45E-6	2.83E-6	5.49E-6
HT29	0.205	1.740	1.658	1.694	1.443	0.020	0.167	95	97	81	-90	-19	1.51E-6	2.96E-6	.
KM12	0.439	2.155	2.166	2.306	2.248	0.028	0.230	101	109	105	-94	-48	1.90E-6	3.38E-6	.
SW-620	0.300	2.118	2.173	2.208	2.020	0.038	0.145	103	105	95	-88	-52	1.76E-6	3.31E-6	6.22E-6
**CNS Cancer**															
SF-268	0.652	1.993	1.962	2.022	2.055	0.113	0.271	98	102	105	-83	-59	1.96E-6	3.62E-6	6.69E-6
SF-295	1.431	3.305	3.250	3.296	3.307	0.134	0.667	97	99	100	-91	-53	1.83E-6	3.35E-6	6.12E-6
SF-539	0.860	2.550	2.364	2.575	2.450	0.028	0.195	89	101	94	-97	-77	1.70E-6	3.11E-6	5.69E-6
SNB-19	0.662	2.460	2.325	2.365	2.312	0.053	0.438	92	95	92	-92	-34	1.69E-6	3.16E-6	.
SNB-75	1.019	2.119	1.928	1.990	2.088	0.112	0.541	83	88	97	-89	-47	1.79E-6	3.33E-6	.
U251	0.437	2.125	1.960	2.074	1.882	0.034	0.234	90	97	86	-92	-47	1.58E-6	3.03E-6	.
**Melanoma**															
LOX IMVI	0.371	2.761	2.633	2.643	2.499	0.050	0.200	95	95	89	-87	-46	1.67E-6	3.21E-6	.
MALME-3M	0.709	1.321	1.216	1.290	1.247	0.068	0.366	83	95	88	-90	-48	1.63E-6	3.11E-6	.
M14	0.429	1.949	1.871	1.901	1.912	0.032	0.180	95	97	98	-93	-58	1.78E-6	3.26E-6	5.97E-6
MDA-MB-435	0.442	2.122	2.115	2.197	2.237	0.017	0.184	100	104	107	-96	-58	1.90E-6	3.36E-6	5.92E-6
SK-MEL-2	1.100	2.635	2.612	2.673	2.581	0.163	0.667	99	103	97	-85	-39	1.80E-6	3.40E-6	.
SK-MEL-28	1.080	2.857	2.825	2.881	2.850	0.022	0.276	98	101	100	-98	-74	1.78E-6	3.19E-6	5.72E-6
SK-MEL-5	0.867	2.916	2.964	2.906	2.889	-0.003	0.266	102	100	99	-100	-69	1.76E-6	3.14E-6	5.60E-6
UACC-257	0.912	2.142	2.073	2.096	2.070	0.113	0.494	94	96	94	-88	-46	1.75E-6	3.30E-6	.
UACC-62	0.671	2.764	2.603	2.665	2.694	0.016	0.387	92	95	97	-98	-42	1.74E-6	3.14E-6	.
**Ovarian Cancer**															
IGROV1	0.408	1.897	1.802	1.843	1.791	0.038	0.197	94	96	93	-91	-52	1.71E-6	3.20E-6	6.00E-6
OVCAR-3	0.616	1.975	2.002	2.116	1.986	0.040	0.210	102	110	101	-94	-66	1.82E-6	3.30E-6	5.97E-6
OVCAR-4	0.630	1.612	1.565	1.725	1.732	0.073	0.026	95	112	112	-88	-96	2.04E-6	3.62E-6	6.43E-6
OVCAR-5	0.622	1.502	1.454	1.428	1.416	0.025	0.128	95	92	90	-96	-80	1.64E-6	3.05E-6	5.66E-6
OVCAR-8	0.390	1.948	1.947	1.977	1.816	0.079	0.236	100	102	91	-80	-39	1.75E-6	3.42E-6	.
NCI/ADR-RES	0.560	1.961	1.974	2.015	1.846	0.121	0.241	101	104	92	-78	-57	1.76E-6	3.46E-6	6.81E-6
SK-OV-3	0.868	1.998	1.958	1.975	2.007	0.033	0.280	96	98	101	-96	-68	1.81E-6	3.25E-6	5.83E-6
**Renal Cancer**															
786-0	0.538	2.320	2.168	2.189	2.133	0.078	0.267	91	93	89	-86	-50	1.68E-6	3.25E-6	6.27E-6
A498	1.562	2.307	2.196	2.340	2.347	0.095	0.547	85	104	105	-94	-65	1.90E-6	3.38E-6	6.02E-6
ACHN	0.343	1.484	1.459	1.498	1.447	0.003	0.099	98	101	97	-99	-71	1.73E-6	3.12E-6	5.61E-6
CAKI-1	0.557	2.745	2.625	2.713	2.703	0.003	0.235	95	99	98	-99	-58	1.75E-6	3.14E-6	5.62E-6
RXF 393	1.136	1.989	1.992	1.986	1.826	0.164	0.597	100	100	81	-86	-47	1.53E-6	3.06E-6	.
SN12C	0.653	2.702	2.642	2.687	2.544	0.063	0.382	97	99	92	-90	-42	1.70E-6	3.20E-6	.
TK-10	0.684	1.800	1.660	1.719	1.711	0.082	0.208	87	93	92	-88	-70	1.71E-6	3.24E-6	6.14E-6
UO-31	0.818	2.331	2.192	2.188	2.102	0.007	0.216	91	91	85	-99	-74	1.55E-6	2.89E-6	5.41E-6
**Prostate Cancer**															
PC-3	0.632	2.566	2.375	2.418	2.318	0.033	0.130	90	92	87	-95	-80	1.60E-6	3.01E-6	5.67E-6
DU-145	0.443	1.635	1.575	1.776	1.705	0.008	0.021	95	112	106	-98	-95	1.88E-6	3.30E-6	5.80E-6
**Breast Cancer**															
MCF7	0.741	3.083	2.866	2.950	2.898	0.184	0.488	91	94	92	-75	-34	1.79E-6	3.55E-6	.
MDA-MB-231/ATCC	0.695	1.576	1.499	1.612	1.508	0.068	0.361	91	104	92	-90	-48	1.70E-6	3.20E-6	.
HS 578T	1.223	2.385	2.197	2.274	2.277	0.386	0.845	84	90	91	-68	-31	1.80E-6	3.71E-6	.
BT-549	1.095	2.259	2.099	2.118	2.109	0.052	0.516	86	88	87	-95	-53	1.60E-6	3.00E-6	5.65E-6
T-47D	0.561	1.307	1.171	1.265	1.150	0.107	0.349	82	94	79	-81	-38	1.52E-6	3.12E-6	.
MDA-MB-468	0.720	1.204	1.175	1.245	1.140	0.082	0.242	94	108	87	-89	-66	1.62E-6	3.12E-6	6.02E-6

National Cancer Institute Developmental Therapeutics Program Mean Graphs	NSC : D - 805618/1	Units :Molar	SSPL :0YYN	EXP. ID :1808NS93
	Report Date :October 03, 2018		Test Date :August 27, 2018	

Panel/Cell Line	Log_{10}GI50	Log_{10}TGI	Log_{10}LC50
**Leukemia**			
HL-60(TB)	-5.74	-5.44	
K-562	-5.86	-5.53	
MOLT-4	-5.76	-5.46	
RPMI-8226	-5.73	-5.43	
SR	-5.86	-5.50	
**Non-Small Cell Lung Cancer**			
A549/ATCC	-5.77	-5.49	
EKVX	-5.77	-5.51	-5.25
HOP-62	-5.76	-5.47	
HOP-92	-5.93	-5.61	-5.29
NCI-H226	-5.71	-5.42	
NCI-H23	-5.77	-5.49	
NCI-H322M	-5.75	-5.50	-5.25
NCI-H460	-5.74	-5.47	
NCI-H522	-5.75	-5.46	
**Colon Cancer**			
COLO 205	-5.77	-5.48	
HCC-2998	-5.75	-5.48	-5.21
HCT-116	-5.77	-5.49	
HCT-15	-5.84	-5.55	-5.26
HT29	-5.82	-5.53	
KM12	-5.72	-5.47	
SW-620	-5.75	-5.48	-5.21
**CNS Cancer**			
SF-268	-5.71	-5.44	-5.17
SF-295	-5.74	-5.48	-5.21
SF-539	-5.77	-5.51	-5.25
SNB-19	-5.77	-5.50	
SNB-75	-5.75	-5.48	
U251	-5.80	-5.52	
**Melanoma**			
LOX IMVI	-5.78	-5.49	
MALME-3M	-5.79	-5.51	
M14	-5.75	-5.49	-5.22
MDA-MB-435	-5.72	-5.47	-5.23
SK-MEL-2	-5.74	-5.47	
SK-MEL-28	-5.75	-5.50	-5.24
SK-MEL-5	-5.75	-5.50	-5.25
UACC-257	-5.76	-5.48	
UACC-62	-5.76	-5.50	
**Ovarian Cancer**			
IGROV1	-5.77	-5.49	-5.22
OVCAR-3	-5.74	-5.48	-5.22
OVCAR-4	-5.69	-5.44	-5.19
OVCAR-5	-5.78	-5.52	-5.25
OVCAR-8	-5.76	-5.47	
NCI/ADR-RES	-5.75	-5.46	-5.17
SK-OV-3	-5.74	-5.49	-5.23
**Renal Cancer**			
786-0	-5.77	-5.49	-5.20
A498	-5.72	-5.47	-5.22
ACHN	-5.76	-5.51	-5.25
CAKI-1	-5.76	-5.50	-5.25
RXF 393	-5.81	-5.51	
SN12C	-5.77	-5.49	
TK-10	-5.77	-5.49	-5.21
UO-31	-5.81	-5.54	-5.27
**Prostate Cancer**			
PC-3	-5.80	-5.52	-5.25
DU-145	-5.73	-5.48	-5.24
**Breast Cancer**			
MCF7	-5.75	-5.45	
MDA-MB-231/ATCC	-5.77	-5.49	
HS 578T	-5.74	-5.43	
BT-549	-5.80	-5.52	-5.25
T-47D	-5.82	-5.51	
MDA-MB-468	-5.79	-5.51	-5.22
_MID	-5.77	-5.49	-5.23
Delta	0.16	0.12	0.06
Range	0.24	0.19	0.12

GI50

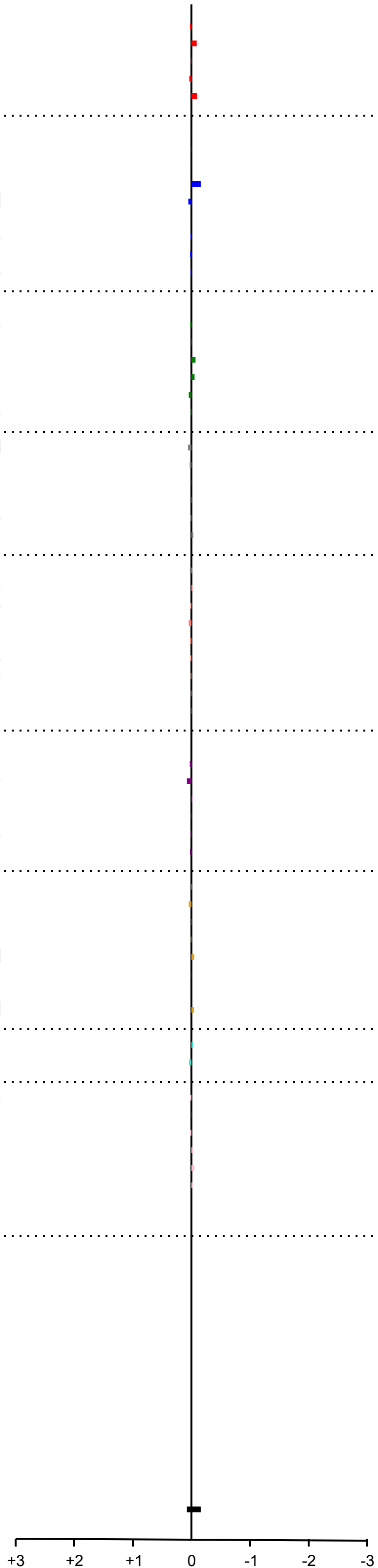

TGI

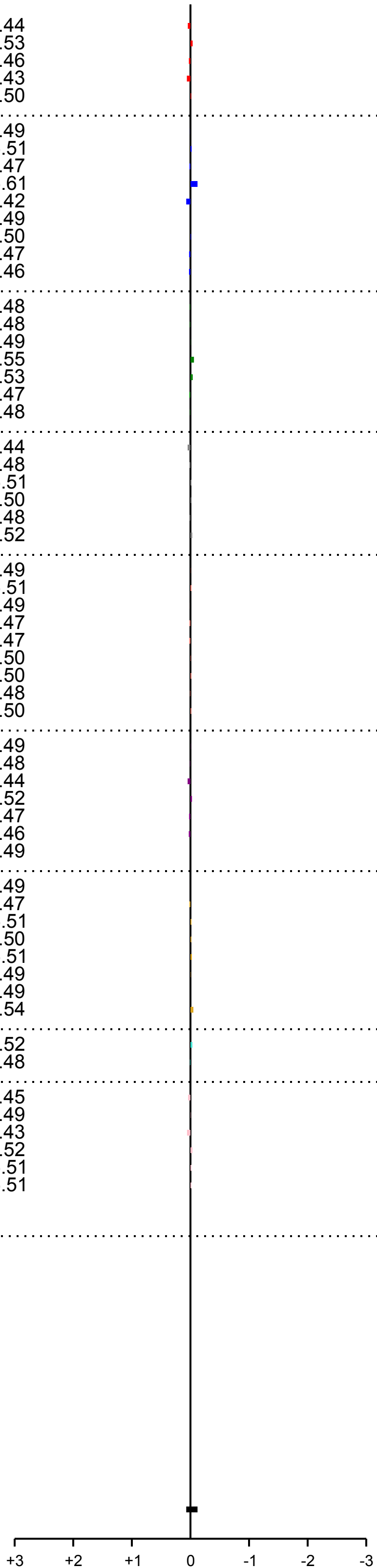

LC50

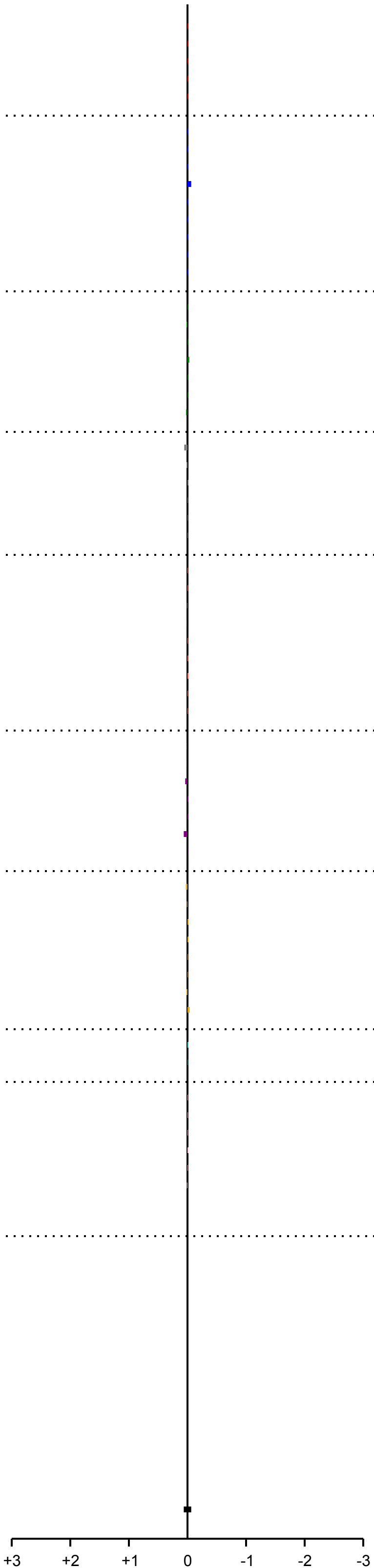

National Cancer Institute Developmental Therapeutics Program Dose Response Curves	NSC : D - 805618/1	SSPL:0YYN	EXP. ID:1808NS93
	Report Date:October 03, 2018	Test Date:August 27, 2018	

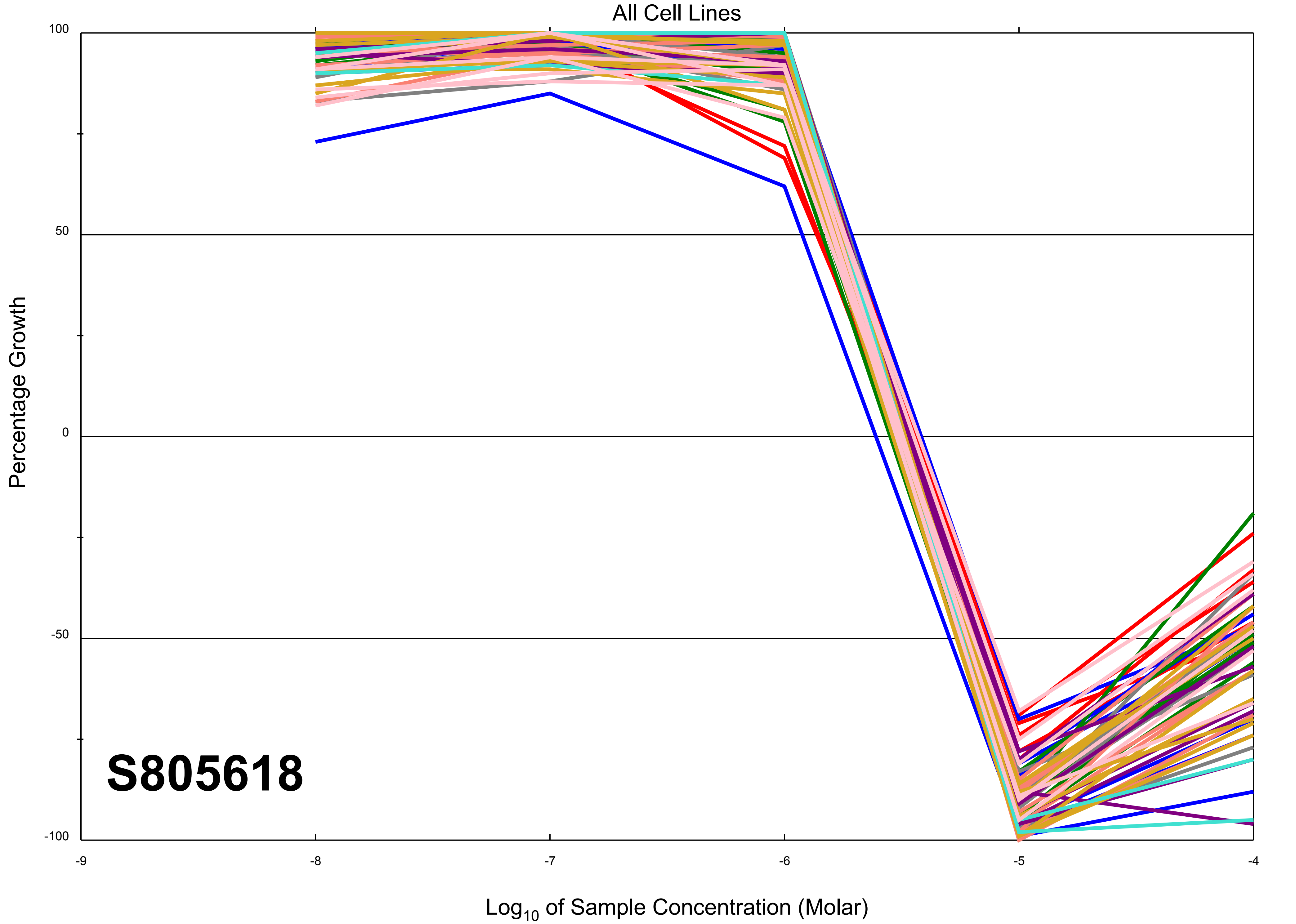

Supplement: Supplementary file 2 — Supplementary Information 2. [file 41598_2024_56313_MOESM2_ESM.pdf]
