# Supplementary material for: Design, synthesis and in vitro anticancer activity of some new lomefloxacin derivatives
Source: Sci Rep. 2024 Mar 14;14:6175. doi: 10.1038/s41598-024-56313-w (PMC10940605; doi:10.1038/s41598-024-56313-w)

National Cancer Institute Developmental Therapeutics Program Dose Response Curves	NSC: D - 805621 / 1	SSPL: 0YYN	EXP. ID: 1809NS99
	Report Date: October 03, 2018	Test Date: September 10, 2018	

Leukemia

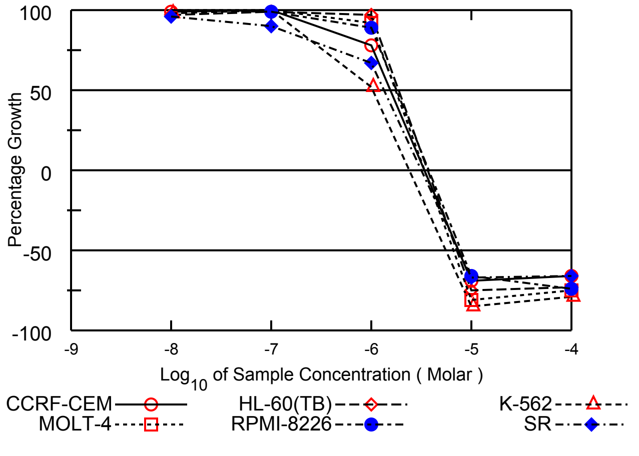

Non-Small Cell Lung Cancer

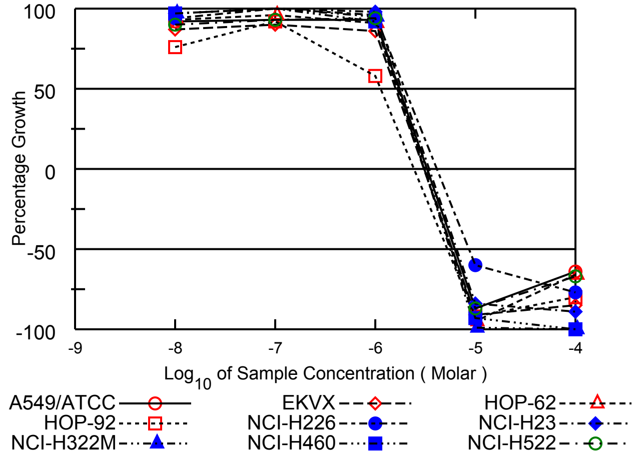

Colon Cancer

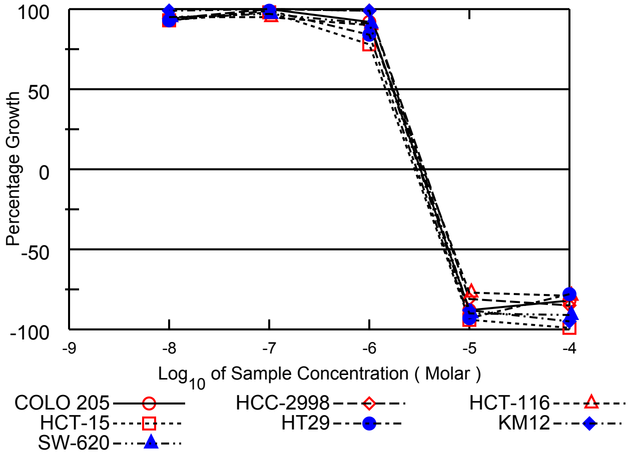

CNS Cancer

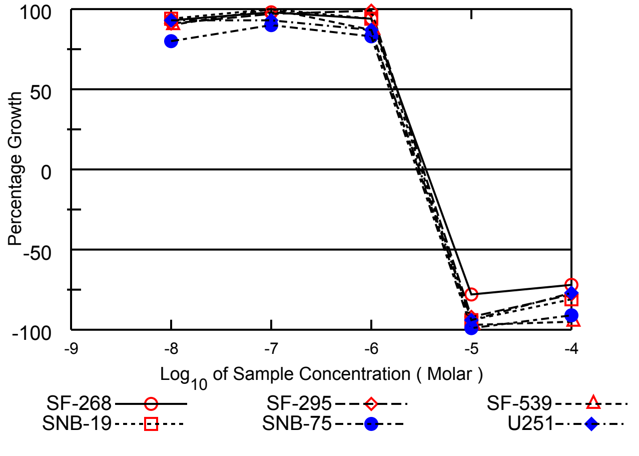

Melanoma

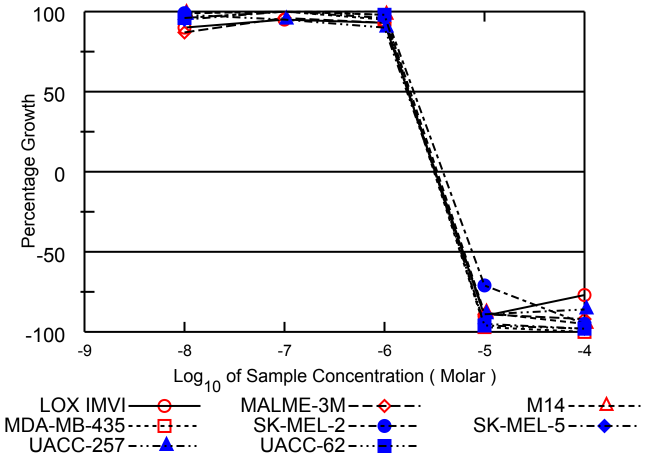

Ovarian Cancer

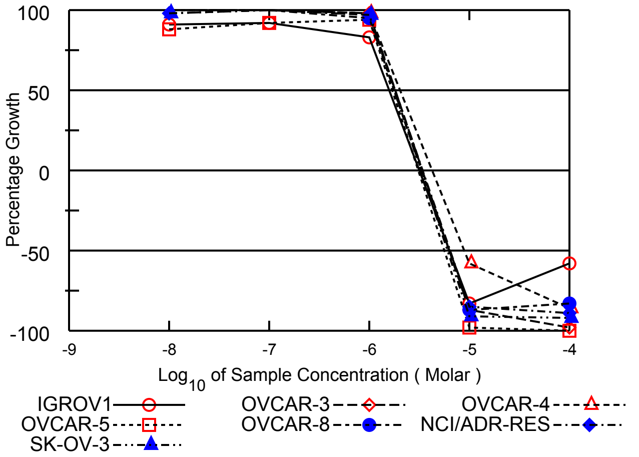

Renal Cancer

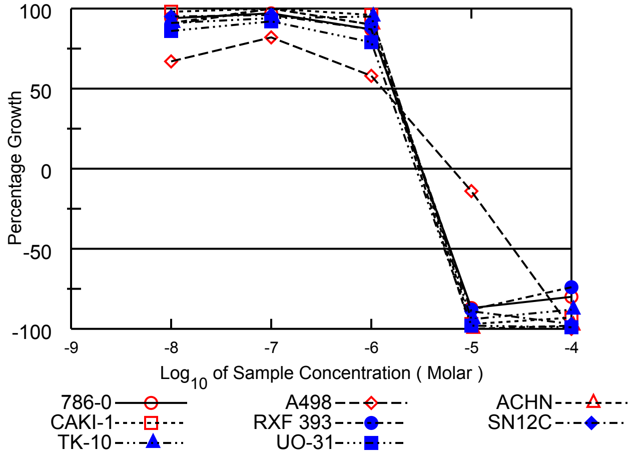

Prostate Cancer

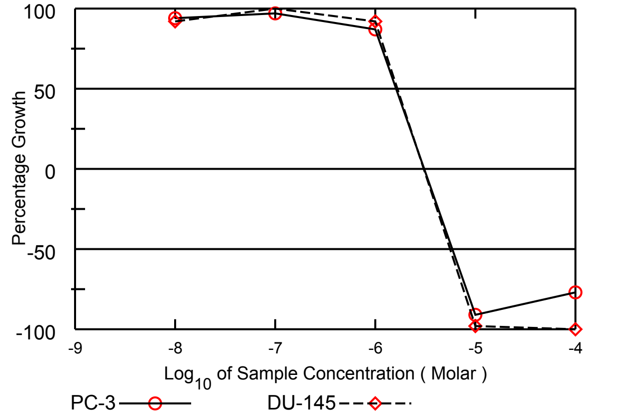

Breast Cancer

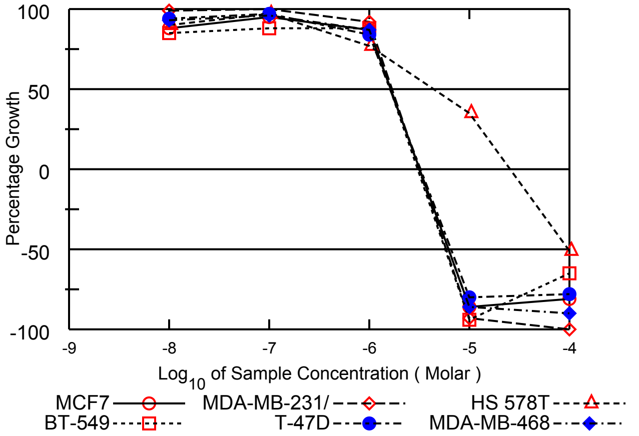

National Cancer Institute Developmental Therapeutics Program
In-Vitro Testing Results

NSC : D - 805621 / 1	Experiment ID : 1809NS99	Test Type : 08	Units : Molar
Report Date : October 03, 2018	Test Date : September 10, 2018	QNS :	MC :
COMI : L1d	Stain Reagent : SRB Dual-Pass Related	SSPL : 0YYN	

			Log10 Concentration												
	Time		Mean Optical Densities					Percent Growth							
Panel/Cell Line	Zero	Ctrl	-8.0	-7.0	-6.0	-5.0	-4.0	-8.0	-7.0	-6.0	-5.0	-4.0	GI50	TGI	LC50
Leukemia															
CCRF-CEM	0.469	2.216	2.197	2.226	1.831	0.146	0.160	99	101	78	-69	-66	1.55E-6	3.39E-6	7.43E-6
HL-60(TB)	0.957	3.271	3.210	3.240	3.202	0.240	0.257	97	99	97	-75	-73	1.88E-6	3.66E-6	7.16E-6
K-562	0.288	2.778	2.727	2.795	1.589	0.043	0.062	98	101	52	-85	-79	1.04E-6	2.40E-6	5.55E-6
MOLT-4	0.645	3.013	3.006	3.021	2.828	0.121	0.161	100	100	92	-81	-75	1.75E-6	3.40E-6	6.60E-6
RPMI-8226	0.853	2.832	2.845	2.807	2.617	0.293	0.221	101	99	89	-66	-74	1.79E-6	3.77E-6	7.92E-6
SR	0.278	1.092	1.062	1.007	0.820	0.091	0.094	96	90	67	-67	-66	1.33E-6	3.14E-6	7.43E-6
Non-Small Cell Lung Cancer															
A549/ATCC	0.418	2.277	2.123	2.150	2.147	0.056	0.151	92	93	93	-87	-64	1.73E-6	3.29E-6	6.25E-6
EKVX	0.659	1.561	1.447	1.468	1.438	0.058	0.102	87	90	86	-91	-85	1.60E-6	3.06E-6	5.86E-6
HOP-62	0.565	2.026	1.926	1.965	1.894	0.029	0.190	93	96	91	-95	-66	1.66E-6	3.09E-6	5.74E-6
HOP-92	1.193	1.893	1.724	1.839	1.597	0.090	0.238	76	92	58	-92	-80	1.12E-6	2.42E-6	5.21E-6
NCI-H226	1.559	2.526	2.471	2.565	2.487	0.625	0.357	94	104	96	-60	-77	1.97E-6	4.13E-6	8.64E-6
NCI-H23	0.553	2.058	2.014	2.074	2.027	0.088	0.059	97	101	98	-84	-89	1.83E-6	3.45E-6	6.50E-6
NCI-H322M	0.565	1.910	1.871	1.965	1.839	0.008	-0.014	97	104	95	-99	-100	1.70E-6	3.09E-6	5.60E-6
NCI-H460	0.210	2.262	2.197	2.274	2.090	0.015	-0.005	97	101	92	-93	-100	1.68E-6	3.14E-6	5.86E-6
NCI-H522	0.854	2.525	2.357	2.411	2.431	0.115	0.282	90	93	94	-87	-67	1.76E-6	3.32E-6	6.28E-6
Colon Cancer															
COLO 205	0.460	1.968	1.970	1.999	1.847	0.057	0.085	100	102	92	-88	-82	1.71E-6	3.25E-6	6.17E-6
HCC-2998	0.696	2.417	2.318	2.409	2.393	0.131	0.106	94	100	99	-81	-85	1.86E-6	3.53E-6	6.70E-6
HCT-116	0.176	2.087	1.999	1.987	1.905	0.041	0.037	95	95	90	-77	-79	1.75E-6	3.47E-6	6.90E-6
HCT-15	0.220	1.520	1.433	1.499	1.238	0.013	0.003	93	98	78	-94	-99	1.46E-6	2.84E-6	5.54E-6
HT29	0.270	2.049	1.926	2.025	1.759	0.020	0.061	93	99	84	-93	-78	1.55E-6	2.98E-6	5.73E-6
KM12	0.466	2.488	2.468	2.549	2.472	0.056	0.026	99	103	99	-88	-95	1.83E-6	3.39E-6	6.26E-6
SW-620	0.275	1.829	1.745	1.779	1.689	0.027	0.024	95	97	91	-90	-91	1.68E-6	3.17E-6	5.99E-6
CNS Cancer															
SF-268	0.658	2.162	2.063	2.126	2.068	0.147	0.187	93	98	94	-78	-72	1.80E-6	3.52E-6	6.90E-6
SF-295	1.051	3.124	2.947	3.066	3.094	0.087	0.235	91	97	99	-92	-78	1.80E-6	3.29E-6	6.03E-6
SF-539	0.676	2.307	2.148	2.336	2.095	0.017	0.034	90	102	87	-97	-95	1.59E-6	2.96E-6	5.53E-6
SNB-19	0.618	2.277	2.184	2.285	2.170	0.036	0.117	94	100	94	-94	-81	1.70E-6	3.15E-6	5.81E-6
SNB-75	0.897	1.587	1.446	1.516	1.472	0.013	0.083	80	90	83	-99	-91	1.52E-6	2.87E-6	5.41E-6
U251	0.488	2.374	2.247	2.249	2.133	0.031	0.112	93	93	87	-94	-77	1.61E-6	3.03E-6	5.73E-6
Melanoma															
LOX IMVI	0.501	2.971	2.734	2.847	2.794	0.049	0.118	90	95	93	-90	-77	1.71E-6	3.21E-6	6.02E-6
MALME-3M	0.654	1.421	1.319	1.392	1.364	0.072	0.050	87	96	93	-89	-92	1.72E-6	3.23E-6	6.10E-6
M14	0.444	1.859	1.839	1.859	1.835	0.053	0.021	99	100	98	-88	-95	1.82E-6	3.37E-6	6.25E-6
MDA-MB-435	0.515	2.450	2.373	2.466	2.352	0.014	-0.002	96	101	95	-97	-100	1.71E-6	3.12E-6	5.68E-6
SK-MEL-2	1.524	3.063	3.048	3.122	3.075	0.439	0.070	99	104	101	-71	-95	1.97E-6	3.86E-6	7.53E-6
SK-MEL-5	0.927	3.163	3.062	3.152	3.082	0.048	0.021	95	100	96	-95	-98	1.75E-6	3.19E-6	5.83E-6
UACC-257	0.638	1.749	1.727	1.698	1.634	0.070	0.090	98	95	90	-89	-86	1.67E-6	3.17E-6	6.04E-6
UACC-62	0.688	2.468	2.405	2.572	2.440	0.025	0.017	96	106	98	-96	-98	1.77E-6	3.20E-6	5.78E-6
Ovarian Cancer															
IGROV1	0.390	1.861	1.727	1.737	1.616	0.065	0.163	91	92	83	-83	-58	1.58E-6	3.16E-6	6.30E-6
OVCAR-3	0.390	1.530	1.563	1.608	1.510	0.053	0.007	103	107	98	-87	-98	1.82E-6	3.40E-6	6.34E-6
OVCAR-4	1.096	2.185	2.183	2.236	2.158	0.455	0.154	100	105	98	-58	-86	2.02E-6	4.22E-6	8.82E-6
OVCAR-5	0.565	1.554	1.436	1.473	1.499	0.014	-0.013	88	92	94	-98	-100	1.70E-6	3.10E-6	5.65E-6
OVCAR-8	0.378	1.836	1.846	1.871	1.757	0.049	0.063	101	102	95	-87	-83	1.76E-6	3.32E-6	6.25E-6
NCI/ADR-RES	0.504	1.811	1.791	1.876	1.773	0.075	0.057	98	105	97	-85	-89	1.81E-6	3.41E-6	6.42E-6
SK-OV-3	0.937	2.222	2.202	2.227	2.190	0.081	0.074	98	100	97	-91	-92	1.78E-6	3.28E-6	6.04E-6
Renal Cancer															
786-0	0.571	2.543	2.428	2.479	2.285	0.075	0.112	94	97	87	-87	-80	1.63E-6	3.16E-6	6.13E-6
A498	1.738	2.325	2.132	2.217	2.076	1.494	0.001	67	82	58	-14	-100	1.28E-6	6.36E-6	2.62E-5
ACHN	0.289	1.420	1.320	1.424	1.312	0.001	0.007	91	100	90	-100	-98	1.63E-6	2.99E-6	5.47E-6
CAKI-1	0.555	2.850	2.803	2.843	2.760	0.017	0.039	98	100	96	-97	-93	1.73E-6	3.15E-6	5.71E-6
RXF 393	0.825	1.530	1.460	1.529	1.462	0.102	0.213	90	100	90	-88	-74	1.69E-6	3.22E-6	6.15E-6
SN12C	0.467	2.076	1.988	2.016	1.867	0.050	0.014	95	96	87	-89	-97	1.62E-6	3.11E-6	5.99E-6
TK-10	0.829	2.025	1.921	1.949	1.967	0.047	0.099	91	94	95	-94	-88	1.73E-6	3.18E-6	5.83E-6
UO-31	0.607	2.077	1.872	1.964	1.772	0.011	0.006	86	92	79	-98	-99	1.46E-6	2.79E-6	5.35E-6
Prostate Cancer															
PC-3	0.452	2.203	2.094	2.152	1.984	0.041	0.102	94	97	87	-91	-77	1.62E-6	3.09E-6	5.90E-6
DU-145	0.284	1.317	1.230	1.339	1.237	0.005	-0.029	92	102	92	-98	-100	1.67E-6	3.05E-6	5.57E-6
Breast Cancer															
MCF7	0.363	2.233	2.014	2.139	1.988	0.052	0.068	88	95	87	-86	-81	1.64E-6	3.18E-6	6.20E-6
MDA-MB-231/ATCC	0.530	1.325	1.317	1.367	1.259	0.039	-0.020	99	105	92	-93	-100	1.68E-6	3.14E-6	5.86E-6
HS 578T	1.601	2.565	2.471	2.539	2.343	1.939	0.780	90	97	77	35	-51	4.40E-6	2.55E-5	9.66E-5
BT-549	0.938	2.220	2.026	2.067	2.065	0.060	0.324	85	88	88	-94	-65	1.62E-6	3.05E-6	5.75E-6
T-47D	0.628	1.405	1.358	1.378	1.278	0.127	0.137	94	97	84	-80	-78	1.61E-6	3.25E-6	6.57E-6
MDA-MB-468	0.731	1.339	1.298	1.318	1.263	0.099	0.077	93	96	87	-86	-90	1.64E-6	3.18E-6	6.17E-6

National Cancer Institute Developmental Therapeutics Program	NSC : D - 805621/1	Units :Molar	SSPL :0YYN	EXP. ID :1809NS99
Mean Graphs	Report Date :October 03, 2018		Test Date :September 10, 2018	

Panel/Cell Line	Log_{10}GI50	Log_{10}TGI	Log_{10}LC50
**Leukemia**			
CCRF-CEM	-5.81	-5.47	-5.13
HL-60(TB)	-5.73	-5.44	-5.15
K-562	-5.98	-5.62	-5.26
MOLT-4	-5.76	-5.47	-5.18
RPMI-8226	-5.75	-5.42	-5.10
SR	-5.88	-5.50	-5.13
**Non-Small Cell Lung Cancer**			
A549/ATCC	-5.76	-5.48	-5.20
EKVX	-5.80	-5.51	-5.23
HOP-62	-5.78	-5.51	-5.24
HOP-92	-5.95	-5.62	-5.28
NCI-H226	-5.71	-5.38	-5.06
NCI-H23	-5.74	-5.46	-5.19
NCI-H322M	-5.77	-5.51	-5.25
NCI-H460	-5.77	-5.50	-5.23
NCI-H522	-5.75	-5.48	-5.20
**Colon Cancer**			
COLO 205	-5.77	-5.49	-5.21
HCC-2998	-5.73	-5.45	-5.17
HCT-116	-5.76	-5.46	-5.16
HCT-15	-5.84	-5.55	-5.26
HT29	-5.81	-5.53	-5.24
KM12	-5.74	-5.47	-5.20
SW-620	-5.77	-5.50	-5.22
**CNS Cancer**			
SF-268	-5.74	-5.45	-5.16
SF-295	-5.74	-5.48	-5.22
SF-539	-5.80	-5.53	-5.26
SNB-19	-5.77	-5.50	-5.24
SNB-75	-5.82	-5.54	-5.27
U251	-5.79	-5.52	-5.24
**Melanoma**			
LOX IMVI	-5.77	-5.49	-5.22
MALME-3M	-5.77	-5.49	-5.21
M14	-5.74	-5.47	-5.20
MDA-MB-435	-5.77	-5.51	-5.25
SK-MEL-2	-5.70	-5.41	-5.12
SK-MEL-5	-5.76	-5.50	-5.23
UACC-257	-5.78	-5.50	-5.22
UACC-62	-5.75	-5.49	-5.24
**Ovarian Cancer**			
IGROV1	-5.80	-5.50	-5.20
OVCAR-3	-5.74	-5.47	-5.20
OVCAR-4	-5.70	-5.37	-5.05
OVCAR-5	-5.77	-5.51	-5.25
OVCAR-8	-5.75	-5.48	-5.20
NCI/ADR-RES	-5.74	-5.47	-5.19
SK-OV-3	-5.75	-5.48	-5.22
**Renal Cancer**			
786-0	-5.79	-5.50	-5.21
A498	-5.89	-5.20	-4.58
ACHN	-5.79	-5.52	-5.26
CAKI-1	-5.76	-5.50	-5.24
RXF 393	-5.77	-5.49	-5.21
SN12C	-5.79	-5.51	-5.22
TK-10	-5.76	-5.50	-5.23
UO-31	-5.84	-5.55	-5.27
**Prostate Cancer**			
PC-3	-5.79	-5.51	-5.23
DU-145	-5.78	-5.52	-5.25
**Breast Cancer**			
MCF7	-5.79	-5.50	-5.21
MDA-MB-231/ATCC	-5.77	-5.50	-5.23
HS 578T	-5.36	-4.59	-4.01
BT-549	-5.79	-5.52	-5.24
T-47D	-5.79	-5.49	-5.18
MDA-MB-468	-5.78	-5.50	-5.21
_MID	-5.77	-5.47	-5.18
Delta	0.21	0.15	0.1
Range	0.62	1.03	1.27

GI50

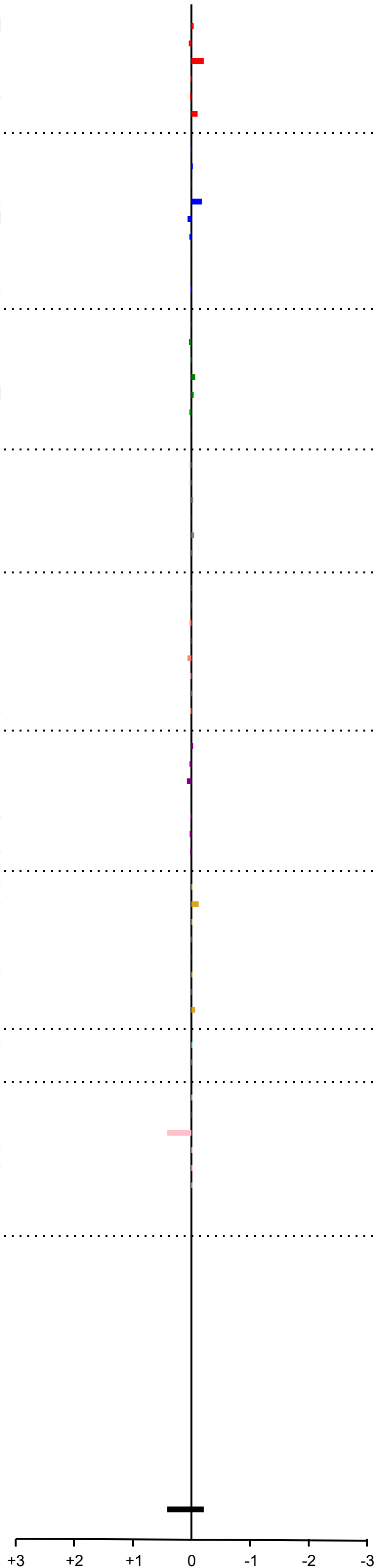

TGI

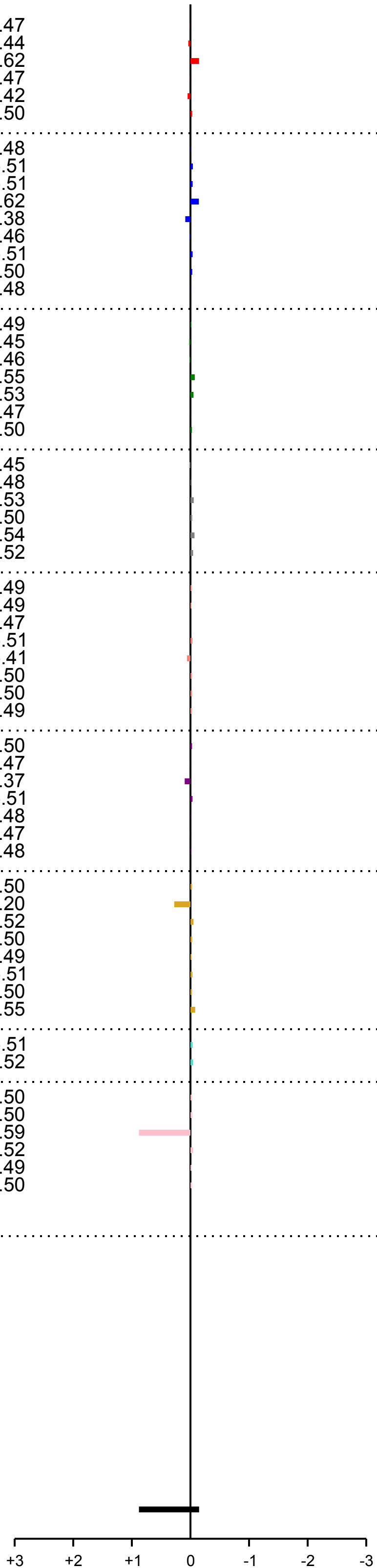

LC50

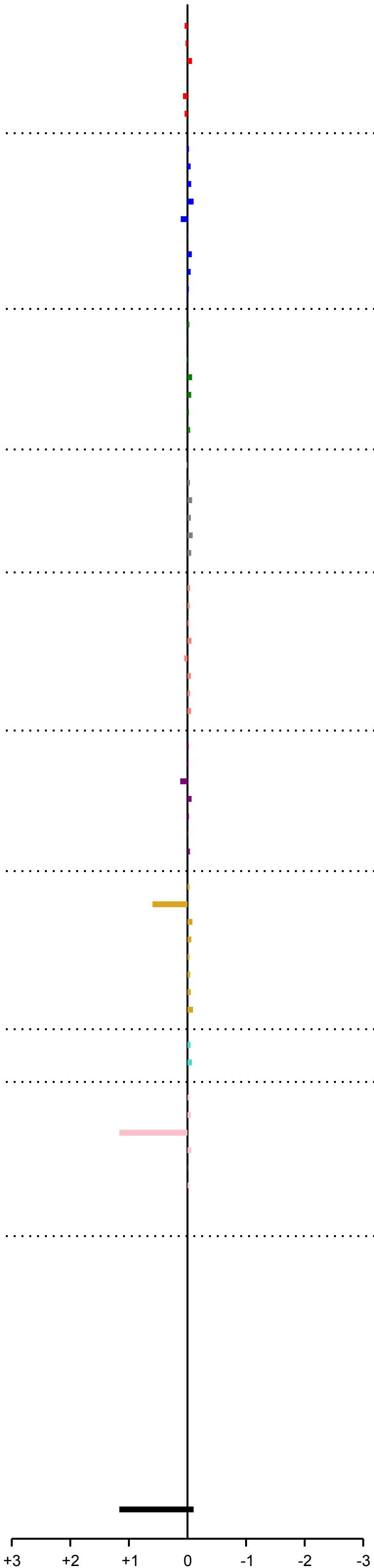

National Cancer Institute Developmental Therapeutics Program<br>Dose Response Curves	NSC : D - 805621/1	SSPL:0YYN	EXP. ID:1809NS99
	Report Date:October 03, 2018	Test Date:September 10, 2018	

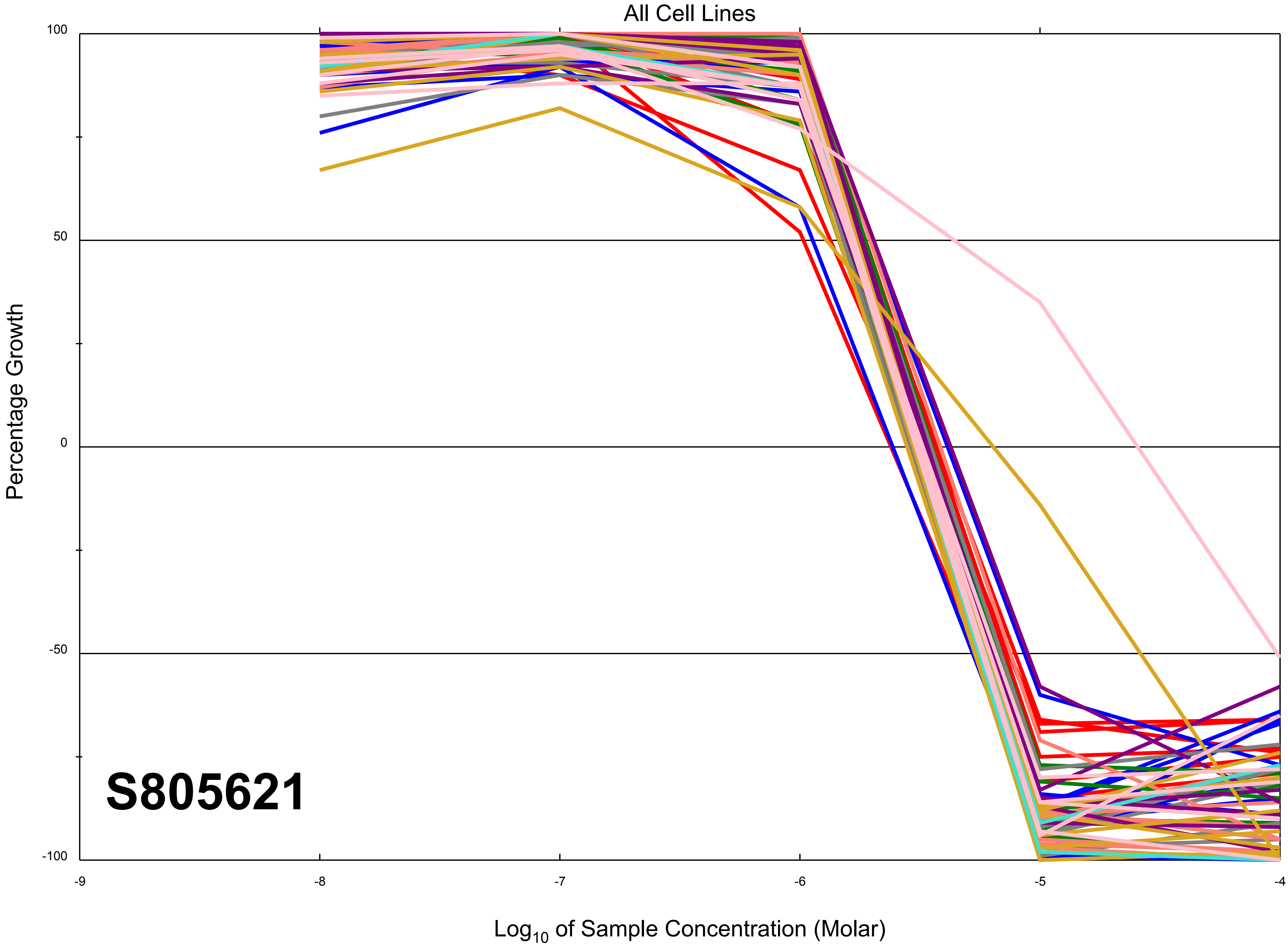

Supplement: Supplementary file 4 — Supplementary Information 4. [file 41598_2024_56313_MOESM4_ESM.pdf]
