# Supplementary material for: Design, synthesis and in vitro anticancer activity of some new lomefloxacin derivatives
Source: Sci Rep. 2024 Mar 14;14:6175. doi: 10.1038/s41598-024-56313-w (PMC10940605; doi:10.1038/s41598-024-56313-w)

National Cancer Institute Developmental Therapeutics Program
Dose Response Curves

NSC: D - 805622 / 1	SSPL: 0YYN	EXP. ID: 1809NS99
Report Date: October 03, 2018	Test Date: September 10, 2018	

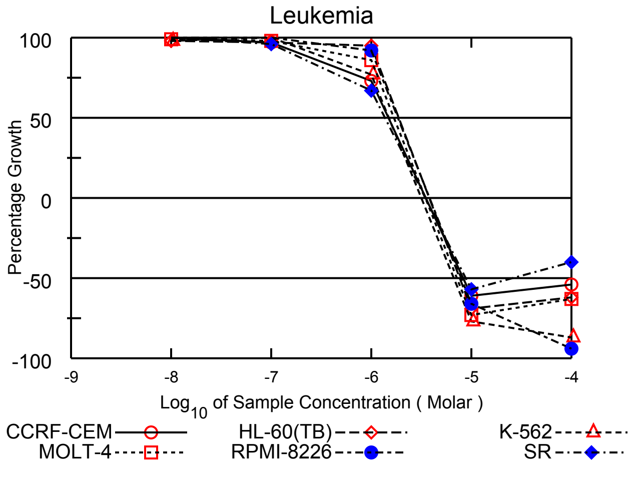

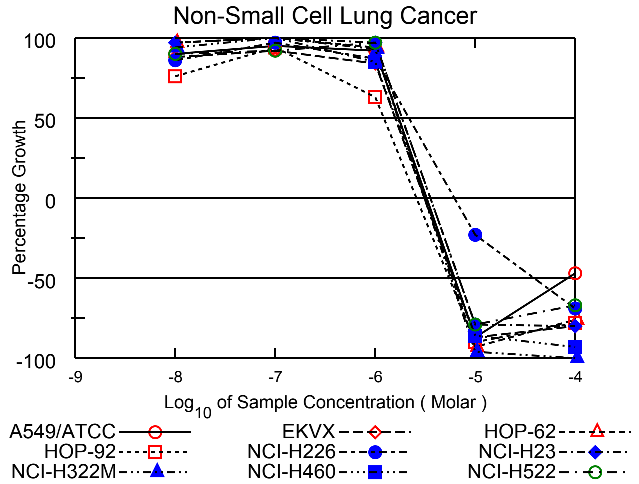

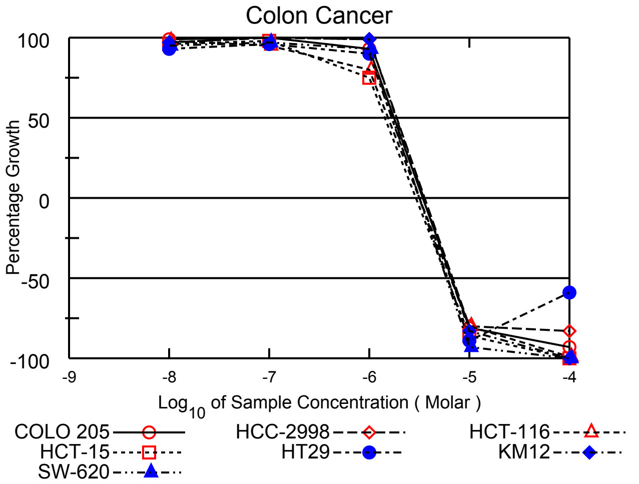

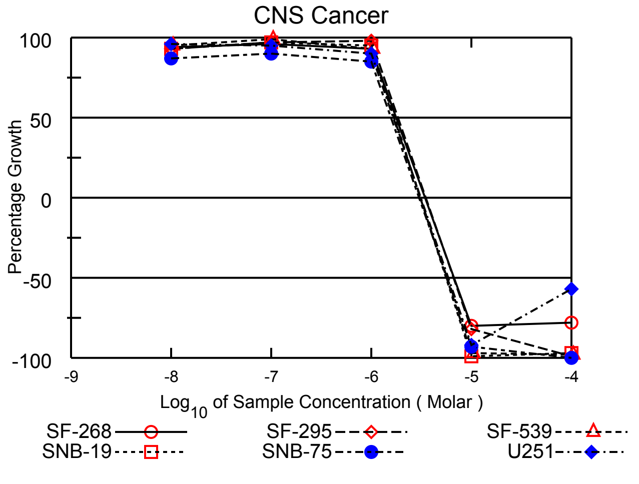

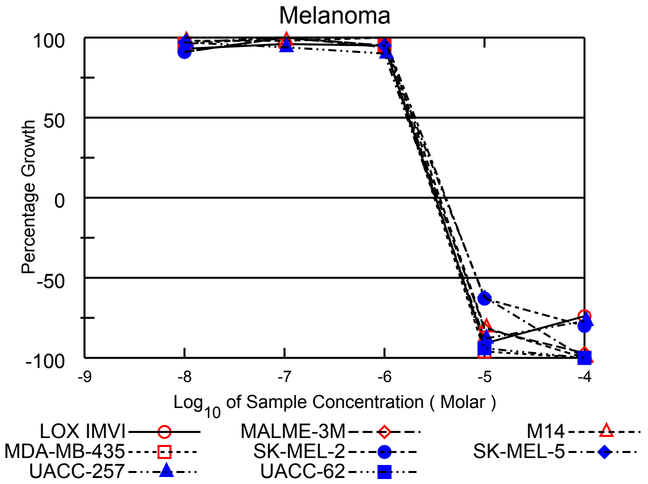

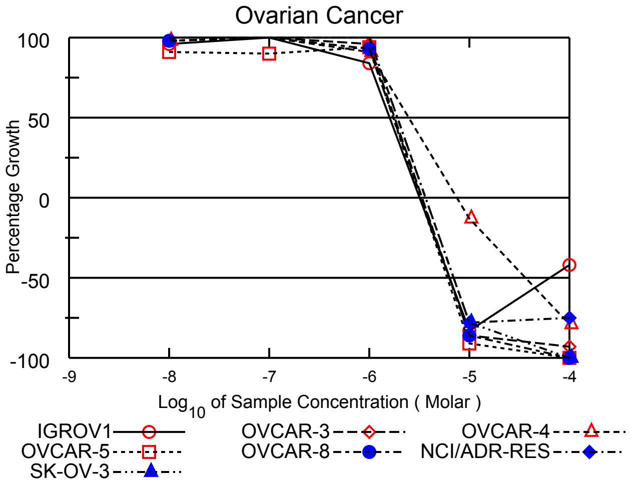

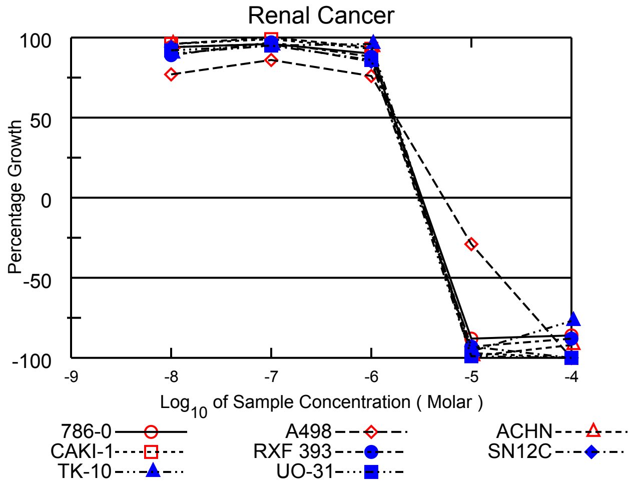

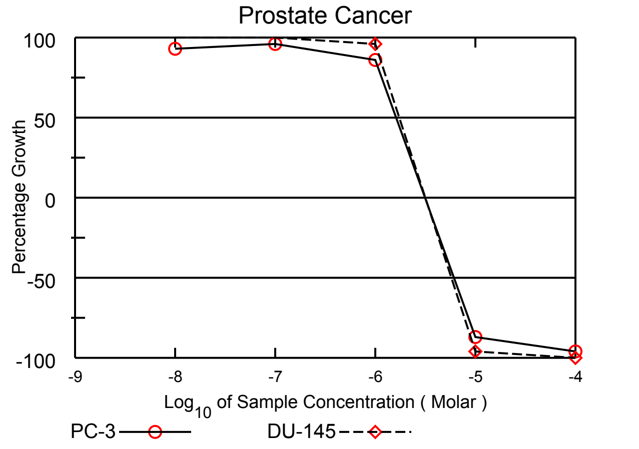

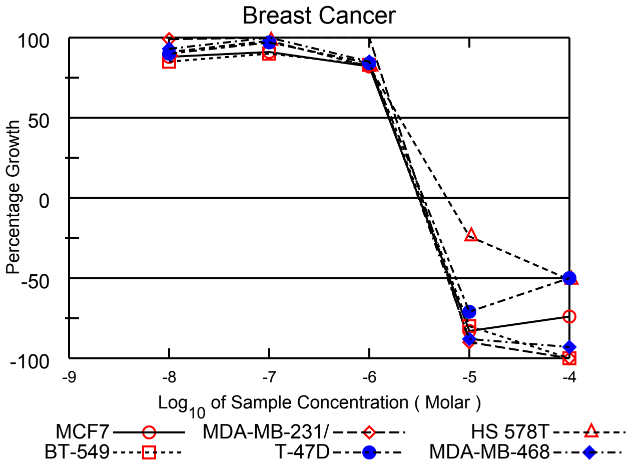

National Cancer Institute Developmental Therapeutics Program
In-Vitro Testing Results

NSC : D - 805622 / 1	Experiment ID : 1809NS99	Test Type : 08	Units : Molar
Report Date : October 03, 2018	Test Date : September 10, 2018	QNS :	MC :
COMI : L1e	Stain Reagent : SRB Dual-Pass Related	SSPL : 0YYN	

Log10 Concentration

Panel/Cell Line	Time Zero	Ctrl	Mean Optical Densities					Percent Growth					GI50	TGI	LC50
			-8.0	-7.0	-6.0	-5.0	-4.0	-8.0	-7.0	-6.0	-5.0	-4.0			
Leukemia															
CCRF-CEM	0.469	2.064	2.073	2.015	1.636	0.185	0.216	101	97	73	-61	-54	1.49E-6	3.52E-6	8.34E-6
HL-60(TB)	0.957	3.273	3.217	3.209	3.151	0.299	0.361	98	97	95	-69	-62	1.88E-6	3.80E-6	7.68E-6
K-562	0.288	2.805	2.743	2.805	2.227	0.067	0.039	98	100	77	-77	-87	1.50E-6	3.17E-6	6.70E-6
MOLT-4	0.645	2.991	2.966	2.955	2.655	0.175	0.241	99	98	86	-73	-63	1.68E-6	3.47E-6	7.17E-6
RPMI-8226	0.853	2.697	2.693	2.699	2.558	0.287	0.054	100	100	92	-66	-94	1.85E-6	3.82E-6	7.89E-6
SR	0.278	1.111	1.109	1.075	0.840	0.120	0.168	100	96	67	-57	-40	1.38E-6	3.49E-6	.
Non-Small Cell Lung Cancer															
A549/ATCC	0.418	2.258	2.065	2.159	2.102	0.056	0.221	90	95	92	-87	-47	1.71E-6	3.26E-6	.
EKVX	0.659	1.520	1.417	1.455	1.385	0.086	0.131	88	92	84	-87	-80	1.59E-6	3.11E-6	6.08E-6
HOP-62	0.565	1.935	1.893	1.943	1.852	0.038	0.137	97	101	94	-93	-76	1.72E-6	3.17E-6	5.87E-6
HOP-92	1.193	1.843	1.688	1.804	1.602	0.121	0.268	76	94	63	-90	-78	1.21E-6	2.58E-6	5.48E-6
NCI-H226	1.559	2.598	2.457	2.566	2.459	1.195	0.476	86	97	87	-23	-69	2.15E-6	6.13E-6	3.78E-5
NCI-H23	0.553	1.997	1.960	2.051	1.947	0.115	0.109	97	104	97	-79	-80	1.84E-6	3.54E-6	6.82E-6
NCI-H322M	0.565	1.900	1.814	1.910	1.804	0.022	-0.045	94	101	93	-96	-100	1.68E-6	3.10E-6	5.70E-6
NCI-H460	0.210	2.315	2.315	2.356	2.008	0.030	0.015	100	102	85	-86	-93	1.61E-6	3.16E-6	6.18E-6
NCI-H522	0.854	2.483	2.315	2.355	2.431	0.180	0.285	90	92	97	-79	-67	1.85E-6	3.55E-6	6.84E-6
Colon Cancer															
COLO 205	0.460	2.008	2.000	2.044	1.893	0.088	0.030	99	102	93	-81	-93	1.76E-6	3.42E-6	6.64E-6
HCC-2998	0.696	2.307	2.254	2.347	2.295	0.137	0.117	97	103	99	-80	-83	1.88E-6	3.57E-6	6.77E-6
HCT-116	0.176	2.044	2.001	1.957	1.666	0.036	0.002	98	95	80	-80	-99	1.54E-6	3.16E-6	6.50E-6
HCT-15	0.220	1.456	1.408	1.437	1.152	0.031	-0.002	96	98	75	-86	-100	1.44E-6	2.93E-6	5.99E-6
HT29	0.270	2.034	1.919	1.964	1.861	0.029	0.112	93	96	90	-89	-59	1.68E-6	3.18E-6	6.04E-6
KM12	0.466	2.462	2.409	2.505	2.436	0.082	-0.037	97	102	99	-83	-100	1.86E-6	3.50E-6	6.62E-6
SW-620	0.275	1.826	1.752	1.778	1.725	0.021	-0.027	95	97	93	-93	-100	1.71E-6	3.18E-6	5.91E-6
CNS Cancer															
SF-268	0.658	2.161	2.075	2.100	2.061	0.130	0.148	94	96	93	-80	-78	1.78E-6	3.45E-6	6.69E-6
SF-295	1.051	3.105	2.955	3.037	3.061	0.192	0.012	93	97	98	-82	-99	1.85E-6	3.51E-6	6.65E-6
SF-539	0.676	2.297	2.208	2.280	2.192	0.024	0.011	95	99	93	-97	-98	1.69E-6	3.10E-6	5.69E-6
SNB-19	0.618	2.253	2.144	2.209	2.170	0.006	0.017	93	97	95	-99	-97	1.70E-6	3.08E-6	5.58E-6
SNB-75	0.897	1.563	1.478	1.495	1.462	0.059	-0.069	87	90	85	-93	-100	1.57E-6	2.99E-6	5.71E-6
U251	0.488	2.318	2.248	2.230	2.137	0.039	0.208	96	95	90	-92	-57	1.66E-6	3.12E-6	5.87E-6
Melanoma															
LOX IMVI	0.501	2.882	2.725	2.781	2.760	0.047	0.131	93	96	95	-91	-74	1.75E-6	3.25E-6	6.04E-6
MALME-3M	0.654	1.417	1.352	1.421	1.369	0.119	0.017	91	101	94	-82	-97	1.77E-6	3.42E-6	6.59E-6
M14	0.444	1.874	1.838	1.843	1.893	0.084	-0.039	98	98	101	-81	-100	1.91E-6	3.59E-6	6.75E-6
MDA-MB-435	0.515	2.374	2.304	2.371	2.288	0.022	-0.065	96	100	95	-96	-100	1.73E-6	3.15E-6	5.76E-6
SK-MEL-2	1.524	3.015	2.883	3.051	3.008	0.566	0.299	91	102	100	-63	-80	2.02E-6	4.10E-6	8.33E-6
SK-MEL-5	0.927	3.147	3.074	3.154	3.039	0.353	-0.039	97	100	95	-62	-100	1.94E-6	4.03E-6	8.39E-6
UACC-257	0.638	1.722	1.689	1.660	1.616	0.078	0.149	97	94	90	-88	-77	1.68E-6	3.21E-6	6.13E-6
UACC-62	0.688	2.431	2.428	2.535	2.511	0.042	-0.048	100	106	105	-94	-100	1.88E-6	3.36E-6	6.01E-6
Ovarian Cancer															
IGROV1	0.390	1.782	1.732	1.806	1.563	0.066	0.225	96	102	84	-83	-42	1.60E-6	3.19E-6	.
OVCAR-3	0.390	1.525	1.545	1.558	1.479	0.056	0.027	102	103	96	-86	-93	1.79E-6	3.38E-6	6.36E-6
OVCAR-4	1.096	2.181	2.165	2.262	2.082	0.957	0.230	98	107	91	-13	-79	2.48E-6	7.53E-6	3.65E-5
OVCAR-5	0.565	1.577	1.484	1.478	1.517	0.050	-0.040	91	90	94	-91	-100	1.73E-6	3.22E-6	6.00E-6
OVCAR-8	0.378	1.830	1.799	1.824	1.728	0.055	-0.045	98	100	93	-86	-100	1.74E-6	3.32E-6	6.32E-6
NCI/ADR-RES	0.504	1.786	1.799	1.847	1.811	0.113	0.127	101	105	102	-78	-75	1.95E-6	3.69E-6	7.01E-6
SK-OV-3	0.937	2.128	2.162	2.186	2.143	0.209	-0.051	103	105	101	-78	-100	1.93E-6	3.68E-6	7.00E-6
Renal Cancer															
786-0	0.571	2.503	2.395	2.435	2.313	0.070	0.081	94	96	90	-88	-86	1.68E-6	3.21E-6	6.13E-6
A498	1.738	2.498	2.325	2.393	2.318	1.234	-0.048	77	86	76	-29	-100	1.78E-6	5.30E-6	1.97E-5
ACHN	0.289	1.361	1.322	1.356	1.294	0.003	0.022	96	100	94	-99	-92	1.69E-6	3.06E-6	5.56E-6
CAKI-1	0.555	2.831	2.743	2.803	2.666	0.015	-0.017	96	99	93	-97	-100	1.68E-6	3.08E-6	5.64E-6
RXF 393	0.825	1.509	1.436	1.486	1.428	0.061	0.103	89	97	88	-93	-88	1.63E-6	3.07E-6	5.81E-6
SN12C	0.467	2.036	1.940	1.978	1.802	0.033	-0.034	94	96	85	-93	-100	1.57E-6	3.01E-6	5.74E-6
TK-10	0.829	2.020	1.905	1.963	1.973	0.037	0.191	90	95	96	-96	-77	1.74E-6	3.17E-6	5.78E-6
UO-31	0.607	1.940	1.834	1.873	1.752	0.007	-0.017	92	95	86	-99	-100	1.56E-6	2.92E-6	5.44E-6
Prostate Cancer															
PC-3	0.452	2.144	2.030	2.085	1.909	0.058	0.017	93	96	86	-87	-96	1.62E-6	3.14E-6	6.10E-6
DU-145	0.284	1.295	1.314	1.365	1.259	0.013	-0.104	102	107	96	-96	-100	1.74E-6	3.18E-6	5.79E-6
Breast Cancer															
MCF7	0.363	2.179	1.964	2.024	1.845	0.062	0.094	88	91	82	-83	-74	1.56E-6	3.13E-6	6.31E-6
MDA-MB-231/ATCC	0.530	1.323	1.312	1.445	1.325	0.053	-0.042	99	115	100	-90	-100	1.84E-6	3.37E-6	6.16E-6
HS 578T	1.601	2.703	2.606	2.682	2.501	1.213	0.789	91	98	82	-24	-51	1.99E-6	5.90E-6	9.39E-5
BT-549	0.938	2.177	1.985	2.056	1.966	0.187	-0.067	85	90	83	-80	-100	1.59E-6	3.23E-6	6.54E-6
T-47D	0.628	1.422	1.342	1.395	1.291	0.181	0.311	90	97	84	-71	-50	1.65E-6	3.47E-6	7.30E-6
MDA-MB-468	0.731	1.280	1.245	1.294	1.196	0.089	0.052	93	102	85	-88	-93	1.59E-6	3.09E-6	6.03E-6

National Cancer Institute Developmental Therapeutics Program<br>Mean Graphs	NSC : D - 805622/1	Units :Molar	SSPL :0YYN	EXP. ID :1809NS99
	Report Date :October 03, 2018		Test Date :September 10, 2018	

Panel/Cell Line	Log_{10}GI50	Log_{10}TGI	Log_{10}LC50
**Leukemia**			
CCRF-CEM	-5.83	-5.45	-5.08
HL-60(TB)	-5.73	-5.42	-5.11
K-562	-5.82	-5.50	-5.17
MOLT-4	-5.77	-5.46	-5.14
RPMI-8226	-5.73	-5.42	-5.10
SR	-5.86	-5.46	
**Non-Small Cell Lung Cancer**			
A549/ATCC	-5.77	-5.49	
EKVX	-5.80	-5.51	-5.22
HOP-62	-5.77	-5.50	-5.23
HOP-92	-5.92	-5.59	-5.26
NCI-H226	-5.67	-5.21	-4.42
NCI-H23	-5.74	-5.45	-5.17
NCI-H322M	-5.77	-5.51	-5.24
NCI-H460	-5.79	-5.50	-5.21
NCI-H522	-5.73	-5.45	-5.16
**Colon Cancer**			
COLO 205	-5.75	-5.47	-5.18
HCC-2998	-5.73	-5.45	-5.17
HCT-116	-5.81	-5.50	-5.19
HCT-15	-5.84	-5.53	-5.22
HT29	-5.78	-5.50	-5.22
KM12	-5.73	-5.46	-5.18
SW-620	-5.77	-5.50	-5.23
**CNS Cancer**			
SF-268	-5.75	-5.46	-5.17
SF-295	-5.73	-5.46	-5.18
SF-539	-5.77	-5.51	-5.24
SNB-19	-5.77	-5.51	-5.25
SNB-75	-5.80	-5.52	-5.24
U251	-5.78	-5.51	-5.23
**Melanoma**			
LOX IMVI	-5.76	-5.49	-5.22
MALME-3M	-5.75	-5.47	-5.18
M14	-5.72	-5.44	-5.17
MDA-MB-435	-5.76	-5.50	-5.24
SK-MEL-2	-5.70	-5.39	-5.08
SK-MEL-5	-5.71	-5.39	-5.08
UACC-257	-5.77	-5.49	-5.21
UACC-62	-5.73	-5.47	-5.22
**Ovarian Cancer**			
IGROV1	-5.80	-5.50	
OVCAR-3	-5.75	-5.47	-5.20
OVCAR-4	-5.61	-5.12	-4.44
OVCAR-5	-5.76	-5.49	-5.22
OVCAR-8	-5.76	-5.48	-5.20
NCI/ADR-RES	-5.71	-5.43	-5.15
SK-OV-3	-5.71	-5.43	-5.15
**Renal Cancer**			
786-0	-5.77	-5.49	-5.21
A498	-5.75	-5.28	-4.70
ACHN	-5.77	-5.51	-5.25
CAKI-1	-5.78	-5.51	-5.25
RXF 393	-5.79	-5.51	-5.24
SN12C	-5.80	-5.52	-5.24
TK-10	-5.76	-5.50	-5.24
UO-31	-5.81	-5.54	-5.26
**Prostate Cancer**			
PC-3	-5.79	-5.50	-5.21
DU-145	-5.76	-5.50	-5.24
**Breast Cancer**			
MCF7	-5.81	-5.50	-5.20
MDA-MB-231/ATCC	-5.74	-5.47	-5.21
HS 578T	-5.70	-5.23	-4.03
BT-549	-5.80	-5.49	-5.18
T-47D	-5.78	-5.46	-5.14
MDA-MB-468	-5.80	-5.51	-5.22
_MID	-5.76	-5.46	-5.14
Delta	0.16	0.13	0.12
Range	0.31	0.47	1.23

GI50

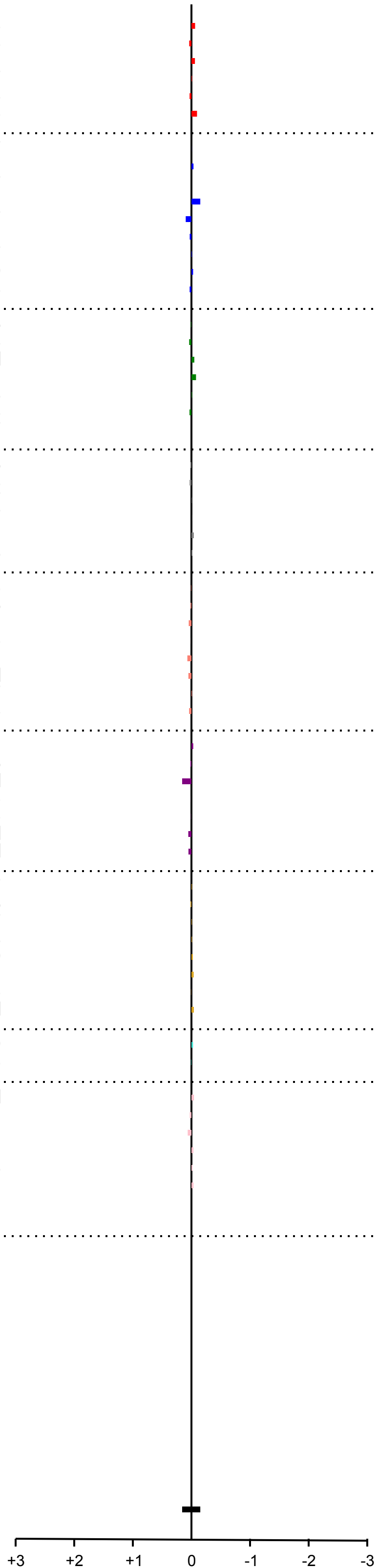

TGI

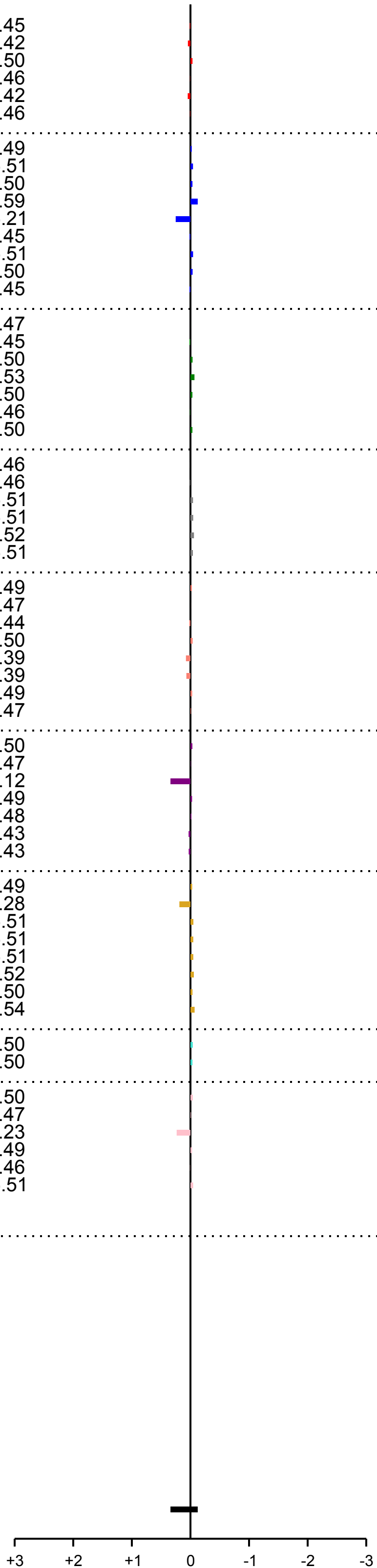

LC50

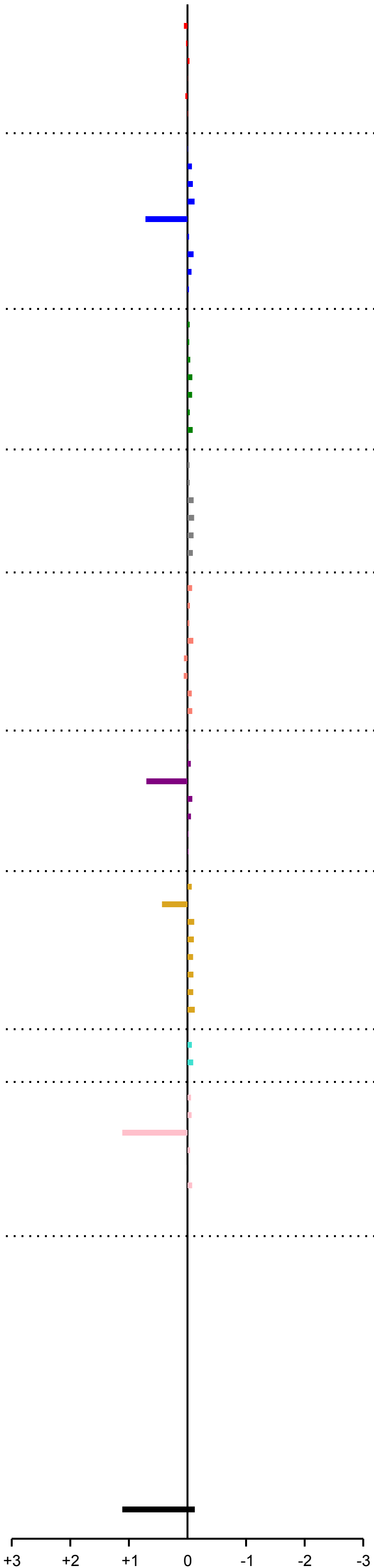

National Cancer Institute Developmental Therapeutics Program<br>Dose Response Curves	NSC : D - 805622/1	SSPL:0YYN	EXP. ID:1809NS99
	Report Date:October 03, 2018	Test Date:September 10, 2018	

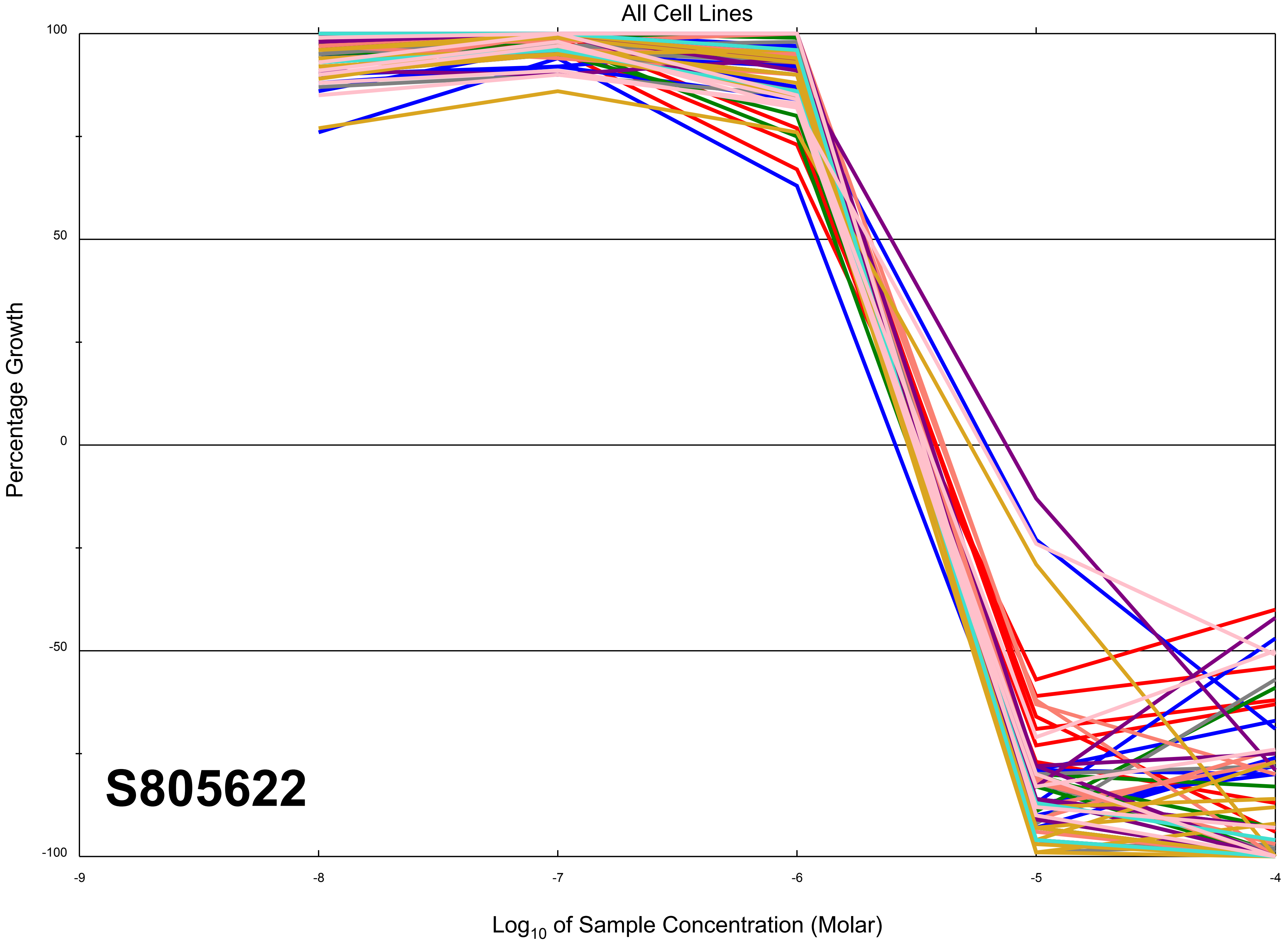

Supplement: Supplementary file 5 — Supplementary Information 5. [file 41598_2024_56313_MOESM5_ESM.pdf]
