# Supplementary material for: Design, synthesis and in vitro anticancer activity of some new lomefloxacin derivatives
Source: Sci Rep. 2024 Mar 14;14:6175. doi: 10.1038/s41598-024-56313-w (PMC10940605; doi:10.1038/s41598-024-56313-w)

National Cancer Institute Developmental Therapeutics Program<br>Dose Response Curves	NSC: D - 805619 / 1	SSPL: 0YYN	EXP. ID: 1809NS99
	Report Date: October 03, 2018	Test Date: September 10, 2018	

Leukemia

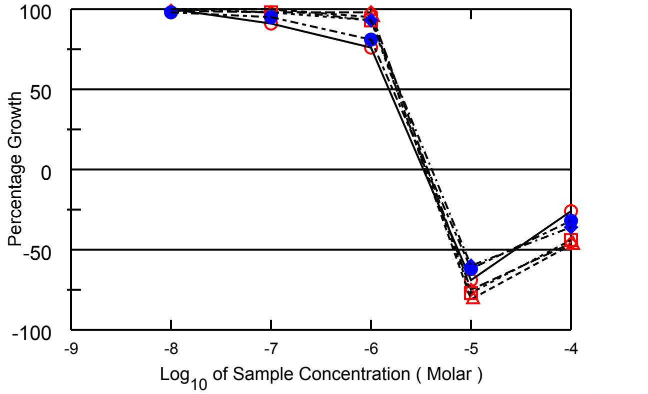

CCRF-CEM, HL-60(TB), K-562, MOLT-4, RPMI-8226, SR

Non-Small Cell Lung Cancer

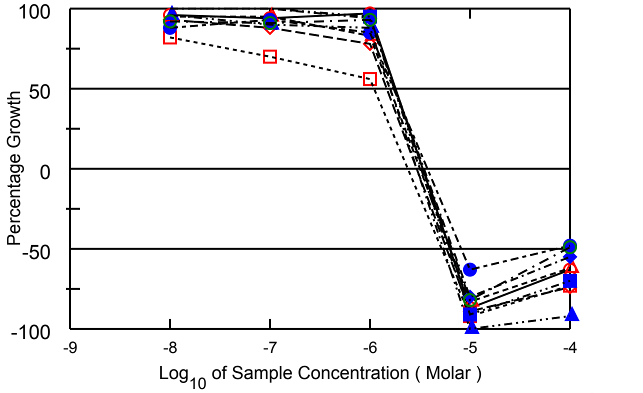

A549/ATCC, EKVX, HOP-62, HOP-92, NCI-H226, NCI-H23, NCI-H322M, NCI-H460, NCI-H522

Colon Cancer

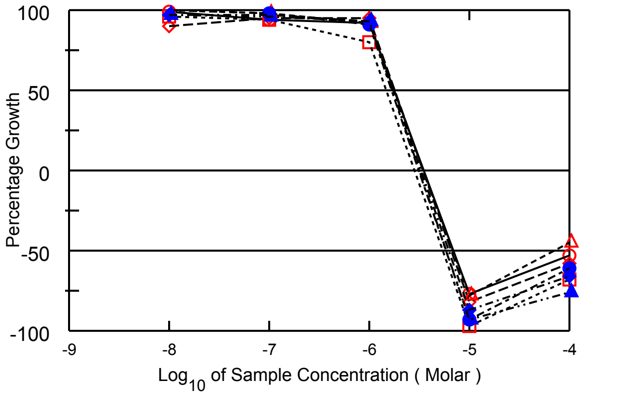

COLO 205, HCC-2998, HCT-116, HCT-15, HT29, KM12, SW-620

CNS Cancer

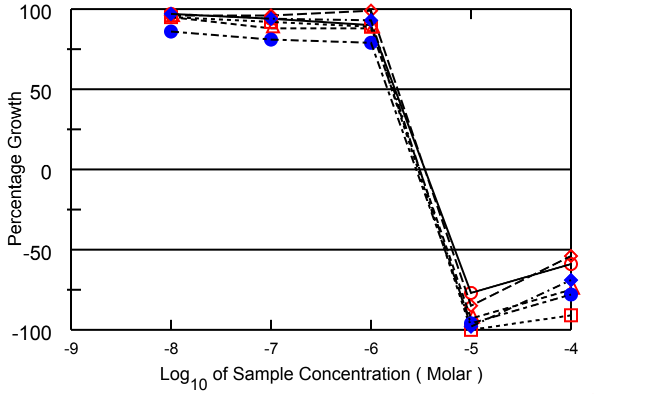

SF-268, SF-295, SF-539, SNB-19, SNB-75, U251

Melanoma

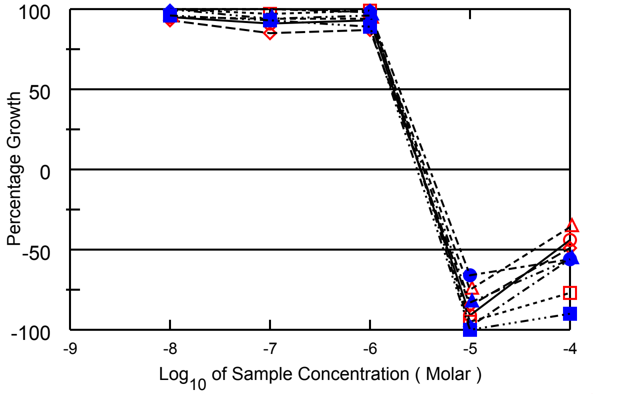

LOX IMVI, MALME-3M, M14, MDA-MB-435, SK-MEL-2, SK-MEL-5, UACC-257, UACC-62

Ovarian Cancer

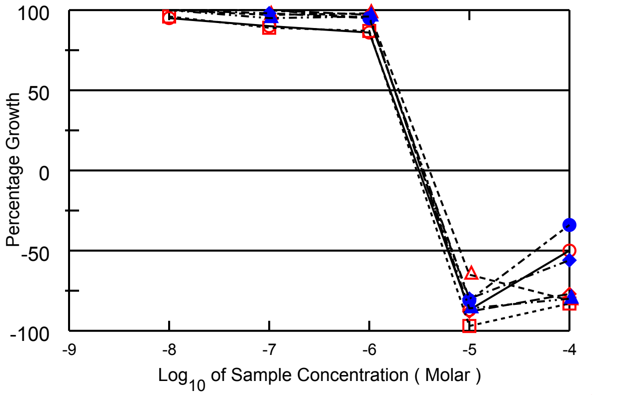

IGROV1, OVCAR-3, OVCAR-4, OVCAR-5, OVCAR-8, NCI/ADR-RES, SK-OV-3

Renal Cancer

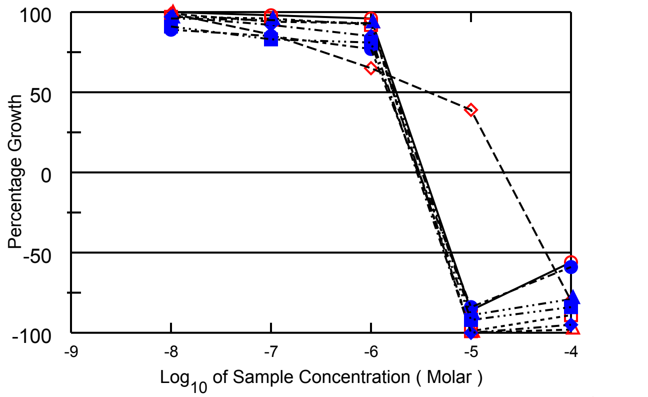

786-0, A498, ACHN, CAKI-1, RXF 393, SN12C, TK-10, UO-31

Prostate Cancer

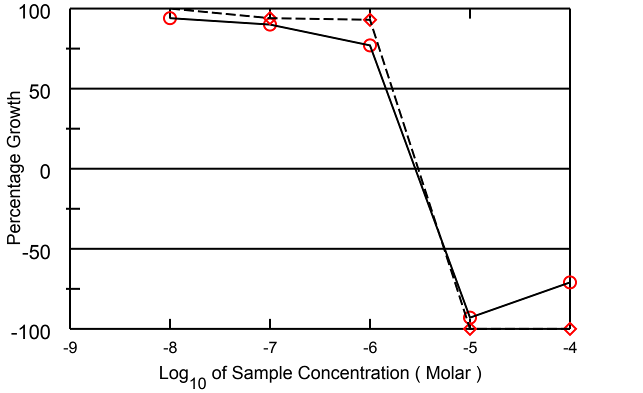

PC-3, DU-145

Breast Cancer

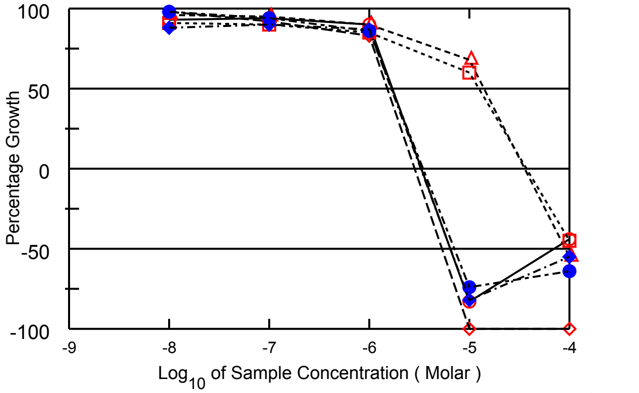

MCF7, MDA-MB-231/, HS 578T, BT-549, T-47D, MDA-MB-468

National Cancer Institute Developmental Therapeutics Program
In-Vitro Testing Results

NSC : D - 805619 / 1	Experiment ID : 1809NS99	Test Type : 08	Units : Molar
Report Date : October 03, 2018	Test Date : September 10, 2018	QNS :	MC :
COMI : L1b	Stain Reagent : SRB Dual-Pass Related	SSPL : 0YYN	

			Log10 Concentration													
	Time		Mean Optical Densities						Percent Growth							
Panel/Cell Line	Zero	Ctrl	-8.0	-7.0	-6.0	-5.0	-4.0	-8.0	-7.0	-6.0	-5.0	-4.0	GI50	TGI		LC50
**Leukemia**																
CCRF-CEM	0.469	2.321	2.318	2.157	1.880	0.147	0.345	100	91	76	-69	-26	1.52E-6	3.35E-6		.
HL-60(TB)	0.957	3.295	3.272	3.239	3.260	0.236	0.512	99	98	98	-75	-46	1.90E-6	3.68E-6		.
K-562	0.288	2.809	2.855	2.802	2.683	0.054	0.152	102	100	95	-81	-47	1.80E-6	3.45E-6		.
MOLT-4	0.645	3.051	3.056	2.998	2.879	0.149	0.364	100	98	93	-77	-44	1.79E-6	3.52E-6		.
RPMI-8226	0.853	2.911	2.880	2.816	2.510	0.328	0.579	98	95	81	-62	-32	1.64E-6	3.69E-6		.
SR	0.278	1.106	1.149	1.107	1.050	0.110	0.179	105	100	93	-60	-36	1.91E-6	4.04E-6		.
**Non-Small Cell Lung Cancer**																
A549/ATCC	0.418	2.276	2.206	2.167	2.219	0.055	0.154	96	94	97	-87	-63	1.80E-6	3.37E-6		6.30E-6
EKVX	0.659	1.578	1.511	1.464	1.375	0.072	0.174	93	88	78	-89	-74	1.47E-6	2.93E-6		5.83E-6
HOP-62	0.565	2.078	2.007	1.996	1.826	0.096	0.216	95	95	83	-83	-62	1.59E-6	3.17E-6		6.33E-6
HOP-92	1.193	1.920	1.791	1.706	1.597	0.096	0.320	82	70	56	-92	-73	1.09E-6	2.38E-6		5.19E-6
NCI-H226	1.559	2.598	2.476	2.527	2.442	0.577	0.815	88	93	85	-63	-48	1.72E-6	3.75E-6		.
NCI-H23	0.553	2.016	2.012	2.012	1.931	0.109	0.247	100	100	94	-80	-55	1.79E-6	3.47E-6		6.71E-6
NCI-H322M	0.565	1.927	1.876	1.792	1.760	-0.002	0.048	96	90	88	-100	-92	1.59E-6	2.93E-6		5.41E-6
NCI-H460	0.210	2.277	2.372	2.330	2.177	0.019	0.062	105	103	95	-91	-70	1.75E-6	3.25E-6		6.02E-6
NCI-H522	0.854	2.484	2.359	2.342	2.377	0.157	0.433	92	91	93	-82	-49	1.77E-6	3.42E-6		.
**Colon Cancer**																
COLO 205	0.460	2.135	2.115	2.043	2.005	0.108	0.219	99	94	92	-77	-53	1.78E-6	3.52E-6		6.96E-6
HCC-2998	0.696	2.411	2.245	2.330	2.329	0.123	0.291	90	95	95	-82	-58	1.80E-6	3.44E-6		6.57E-6
HCT-116	0.176	2.064	2.112	2.026	1.908	0.038	0.098	103	98	92	-78	-45	1.76E-6	3.46E-6		.
HCT-15	0.220	1.593	1.543	1.517	1.314	0.007	0.071	96	94	80	-97	-68	1.47E-6	2.82E-6		5.42E-6
HT29	0.270	2.054	2.065	2.020	1.890	0.019	0.105	101	98	91	-93	-61	1.67E-6	3.12E-6		5.84E-6
KM12	0.466	2.606	2.546	2.550	2.450	0.059	0.158	97	97	93	-87	-66	1.73E-6	3.27E-6		6.20E-6
SW-620	0.275	1.876	1.829	1.819	1.768	0.020	0.067	97	96	93	-93	-76	1.71E-6	3.17E-6		5.89E-6
**CNS Cancer**																
SF-268	0.658	2.090	2.049	1.998	1.941	0.150	0.270	97	94	90	-77	-59	1.73E-6	3.44E-6		6.86E-6
SF-295	1.051	3.198	3.113	3.115	3.176	0.158	0.487	96	96	99	-85	-54	1.85E-6	3.45E-6		6.45E-6
SF-539	0.676	2.466	2.368	2.253	2.252	0.051	0.167	95	88	88	-93	-75	1.62E-6	3.07E-6		5.81E-6
SNB-19	0.618	2.395	2.302	2.250	2.199	-0.009	0.055	95	92	89	-100	-91	1.61E-6	2.96E-6		5.44E-6
SNB-75	0.897	1.583	1.489	1.453	1.441	0.036	0.194	86	81	79	-96	-78	1.47E-6	2.83E-6		5.47E-6
U251	0.488	2.305	2.246	2.199	2.175	0.008	0.149	97	94	93	-98	-69	1.67E-6	3.06E-6		5.58E-6
**Melanoma**																
LOX IMVI	0.501	2.911	2.790	2.705	2.738	0.043	0.279	95	91	93	-91	-44	1.71E-6	3.19E-6		.
MALME-3M	0.654	1.487	1.425	1.363	1.380	0.100	0.333	93	85	87	-85	-49	1.64E-6	3.21E-6		.
M14	0.444	1.964	1.895	1.871	1.873	0.110	0.284	95	94	94	-75	-36	1.82E-6	3.59E-6		.
MDA-MB-435	0.515	2.500	2.525	2.434	2.472	0.028	0.116	101	97	99	-95	-77	1.78E-6	3.24E-6		5.88E-6
SK-MEL-2	1.524	3.034	3.063	3.037	3.005	0.512	0.669	102	100	98	-66	-56	1.96E-6	3.95E-6		7.95E-6
SK-MEL-5	0.927	3.210	3.191	3.214	3.198	0.021	0.406	99	100	99	-98	-56	1.78E-6	3.19E-6		5.73E-6
UACC-257	0.638	1.742	1.742	1.677	1.694	0.108	0.278	100	94	96	-83	-56	1.80E-6	3.43E-6		6.53E-6
UACC-62	0.688	2.454	2.382	2.338	2.265	-0.003	0.071	96	93	89	-100	-90	1.61E-6	2.96E-6		5.44E-6
**Ovarian Cancer**																
IGROV1	0.390	1.903	1.833	1.745	1.694	0.052	0.197	95	90	86	-87	-50	1.62E-6	3.15E-6		.
OVCAR-3	0.390	1.523	1.558	1.530	1.492	0.047	0.092	103	101	97	-88	-77	1.80E-6	3.35E-6		6.23E-6
OVCAR-4	1.096	2.198	2.236	2.167	2.171	0.380	0.207	103	97	98	-65	-81	1.96E-6	3.97E-6		8.05E-6
OVCAR-5	0.565	1.603	1.565	1.487	1.469	0.017	0.095	96	89	87	-97	-83	1.59E-6	2.97E-6		5.56E-6
OVCAR-8	0.378	1.909	1.925	1.902	1.831	0.072	0.251	101	100	95	-81	-34	1.80E-6	3.47E-6		.
NCI/ADR-RES	0.504	1.791	1.804	1.771	1.725	0.099	0.220	101	98	95	-80	-56	1.80E-6	3.48E-6		6.70E-6
SK-OV-3	0.937	2.223	2.246	2.163	2.175	0.132	0.185	102	95	96	-86	-80	1.79E-6	3.37E-6		6.35E-6
**Renal Cancer**																
786-0	0.571	2.449	2.440	2.404	2.382	0.082	0.251	100	98	96	-86	-56	1.80E-6	3.38E-6		6.37E-6
A498	1.738	2.353	2.349	2.267	2.135	1.976	0.344	99	86	65	39	-80	3.64E-6	2.11E-5		5.57E-5
ACHN	0.289	1.471	1.465	1.397	1.391	-0.007	0.007	99	94	93	-100	-98	1.67E-6	3.04E-6		5.51E-6
CAKI-1	0.555	2.901	2.841	2.803	2.710	0.007	0.059	97	96	92	-99	-89	1.66E-6	3.03E-6		5.55E-6
RXF 393	0.825	1.541	1.465	1.431	1.377	0.130	0.337	89	85	77	-84	-59	1.47E-6	3.00E-6		6.13E-6
SN12C	0.467	2.212	2.156	2.076	1.951	-0.008	0.023	97	92	85	-100	-95	1.55E-6	2.88E-6		5.37E-6
TK-10	0.829	1.967	1.918	1.915	1.886	0.095	0.170	96	95	93	-89	-79	1.72E-6	3.25E-6		6.13E-6
UO-31	0.607	2.060	1.926	1.817	1.791	0.052	0.096	91	83	81	-92	-84	1.52E-6	2.96E-6		5.75E-6
**Prostate Cancer**																
PC-3	0.452	2.289	2.187	2.104	1.872	0.031	0.130	94	90	77	-93	-71	1.45E-6	2.84E-6		5.58E-6
DU-145	0.284	1.336	1.361	1.276	1.259	0.001	-0.005	102	94	93	-100	-100	1.67E-6	3.03E-6		5.52E-6
**Breast Cancer**																
MCF7	0.363	2.375	2.228	2.248	2.178	0.061	0.205	93	94	90	-83	-44	1.71E-6	3.31E-6		.
MDA-MB-231/ATCC	0.530	1.263	1.251	1.202	1.141	-0.006	0.001	98	92	83	-100	-100	1.52E-6	2.85E-6		5.34E-6
HS 578T	1.601	2.549	2.507	2.502	2.453	2.249	0.722	96	95	90	68	-55	1.41E-5	3.59E-5		9.12E-5
BT-549	0.938	2.155	2.050	2.035	1.974	1.662	0.512	91	90	85	60	-45	1.23E-5	3.69E-5	>	1.00E-4
T-47D	0.628	1.430	1.411	1.384	1.319	0.162	0.224	98	94	86	-74	-64	1.68E-6	3.45E-6		7.06E-6
MDA-MB-468	0.731	1.324	1.253	1.267	1.248	0.131	0.327	88	90	87	-82	-55	1.66E-6	3.27E-6		6.46E-6

National Cancer Institute Developmental Therapeutics Program Mean Graphs	NSC : D - 805619/1	Units :Molar	SSPL :0YYN	EXP. ID :1809NS99
	Report Date :October 03, 2018		Test Date :September 10, 2018	

Panel/Cell Line	Log_{10}GI50	Log_{10}TGI	Log_{10}LC50
**Leukemia**			
CCRF-CEM	-5.82	-5.47	
HL-60(TB)	-5.72	-5.43	
K-562	-5.75	-5.46	
MOLT-4	-5.75	-5.45	
RPMI-8226	-5.79	-5.43	
SR	-5.72	-5.39	
**Non-Small Cell Lung Cancer**			
A549/ATCC	-5.74	-5.47	-5.20
EKVX	-5.83	-5.53	-5.23
HOP-62	-5.80	-5.50	-5.20
HOP-92	-5.96	-5.62	-5.28
NCI-H226	-5.76	-5.43	
NCI-H23	-5.75	-5.46	-5.17
NCI-H322M	-5.80	-5.53	-5.27
NCI-H460	-5.76	-5.49	-5.22
NCI-H522	-5.75	-5.47	
**Colon Cancer**			
COLO 205	-5.75	-5.45	-5.16
HCC-2998	-5.75	-5.46	-5.18
HCT-116	-5.75	-5.46	
HCT-15	-5.83	-5.55	-5.27
HT29	-5.78	-5.51	-5.23
KM12	-5.76	-5.49	-5.21
SW-620	-5.77	-5.50	-5.23
**CNS Cancer**			
SF-268	-5.76	-5.46	-5.16
SF-295	-5.73	-5.46	-5.19
SF-539	-5.79	-5.51	-5.24
SNB-19	-5.79	-5.53	-5.26
SNB-75	-5.83	-5.55	-5.26
U251	-5.78	-5.51	-5.25
**Melanoma**			
LOX IMVI	-5.77	-5.50	
MALME-3M	-5.78	-5.49	
M14	-5.74	-5.44	
MDA-MB-435	-5.75	-5.49	-5.23
SK-MEL-2	-5.71	-5.40	-5.10
SK-MEL-5	-5.75	-5.50	-5.24
UACC-257	-5.74	-5.46	-5.19
UACC-62	-5.79	-5.53	-5.26
**Ovarian Cancer**			
IGROV1	-5.79	-5.50	
OVCAR-3	-5.75	-5.48	-5.21
OVCAR-4	-5.71	-5.40	-5.09
OVCAR-5	-5.80	-5.53	-5.26
OVCAR-8	-5.74	-5.46	
NCI/ADR-RES	-5.74	-5.46	-5.17
SK-OV-3	-5.75	-5.47	-5.20
**Renal Cancer**			
786-0	-5.75	-5.47	-5.20
A498	-5.44	-4.68	-4.25
ACHN	-5.78	-5.52	-5.26
CAKI-1	-5.78	-5.52	-5.26
RXF 393	-5.83	-5.52	-5.21
SN12C	-5.81	-5.54	-5.27
TK-10	-5.76	-5.49	-5.21
UO-31	-5.82	-5.53	-5.24
**Prostate Cancer**			
PC-3	-5.84	-5.55	-5.25
DU-145	-5.78	-5.52	-5.26
**Breast Cancer**			
MCF7	-5.77	-5.48	
MDA-MB-231/ATCC	-5.82	-5.55	-5.27
HS 578T	-4.85	-4.45	-4.04
BT-549	-4.91	-4.43	> -4.00
T-47D	-5.77	-5.46	-5.15
MDA-MB-468	-5.78	-5.49	-5.19
_MID	-5.74	-5.44	-5.14
Delta	0.22	0.18	0.14
Range	1.11	1.19	1.28

GI50

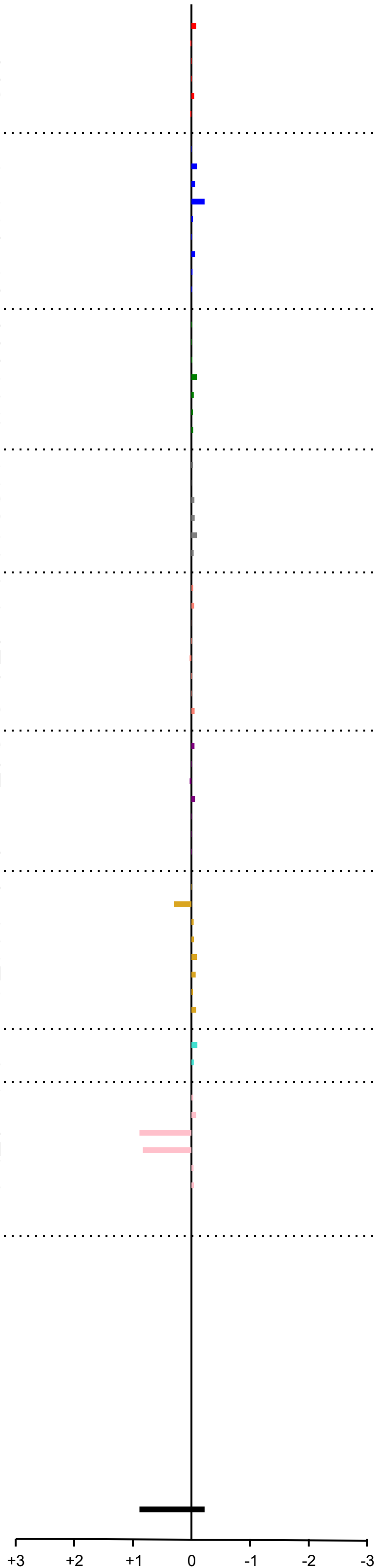

TGI

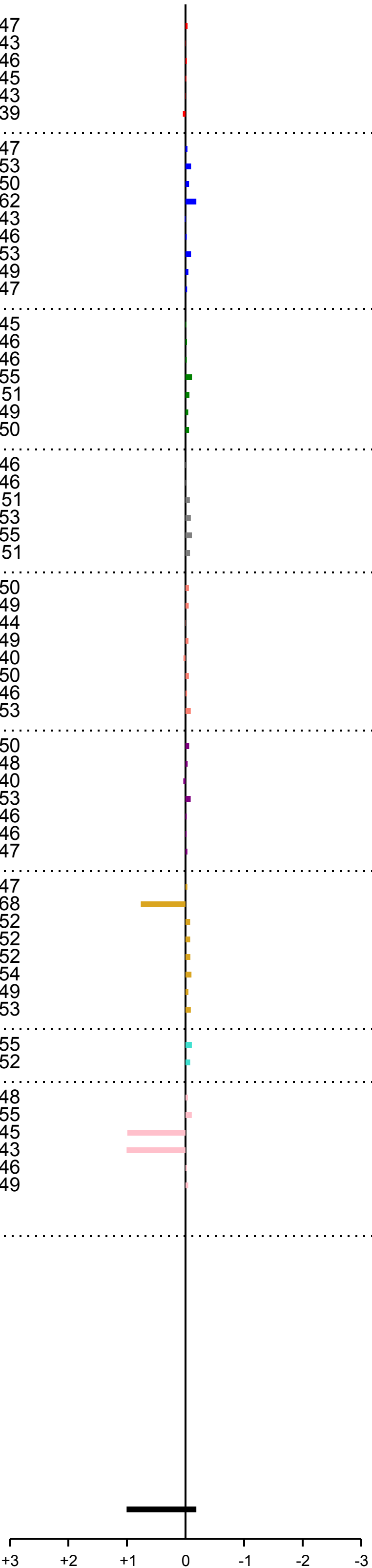

LC50

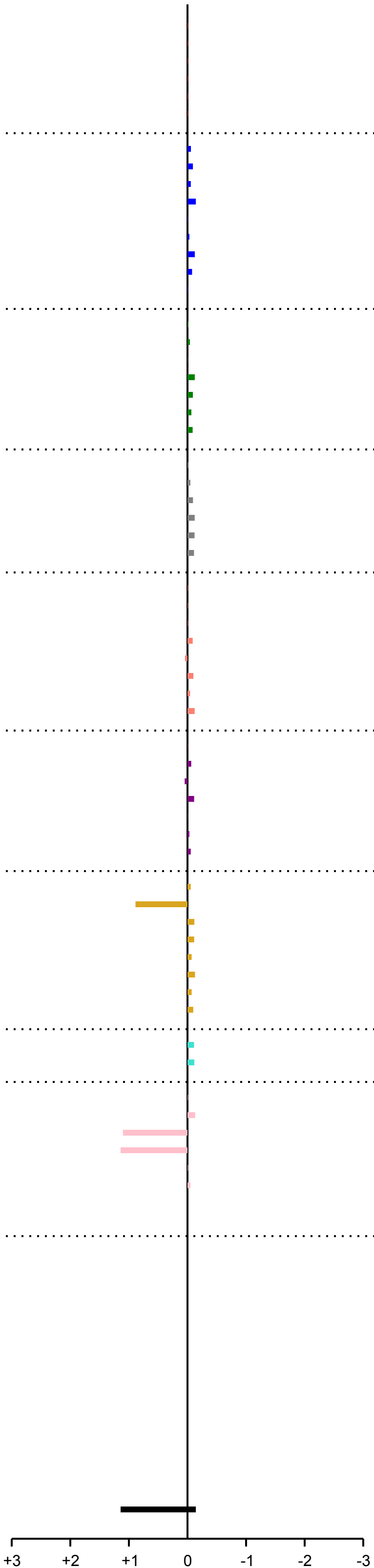

National Cancer Institute Developmental Therapeutics Program<br>Dose Response Curves	NSC : D - 805619/1	SSPL:0YYN	EXP. ID:1809NS99
	Report Date:October 03, 2018	Test Date:September 10, 2018	

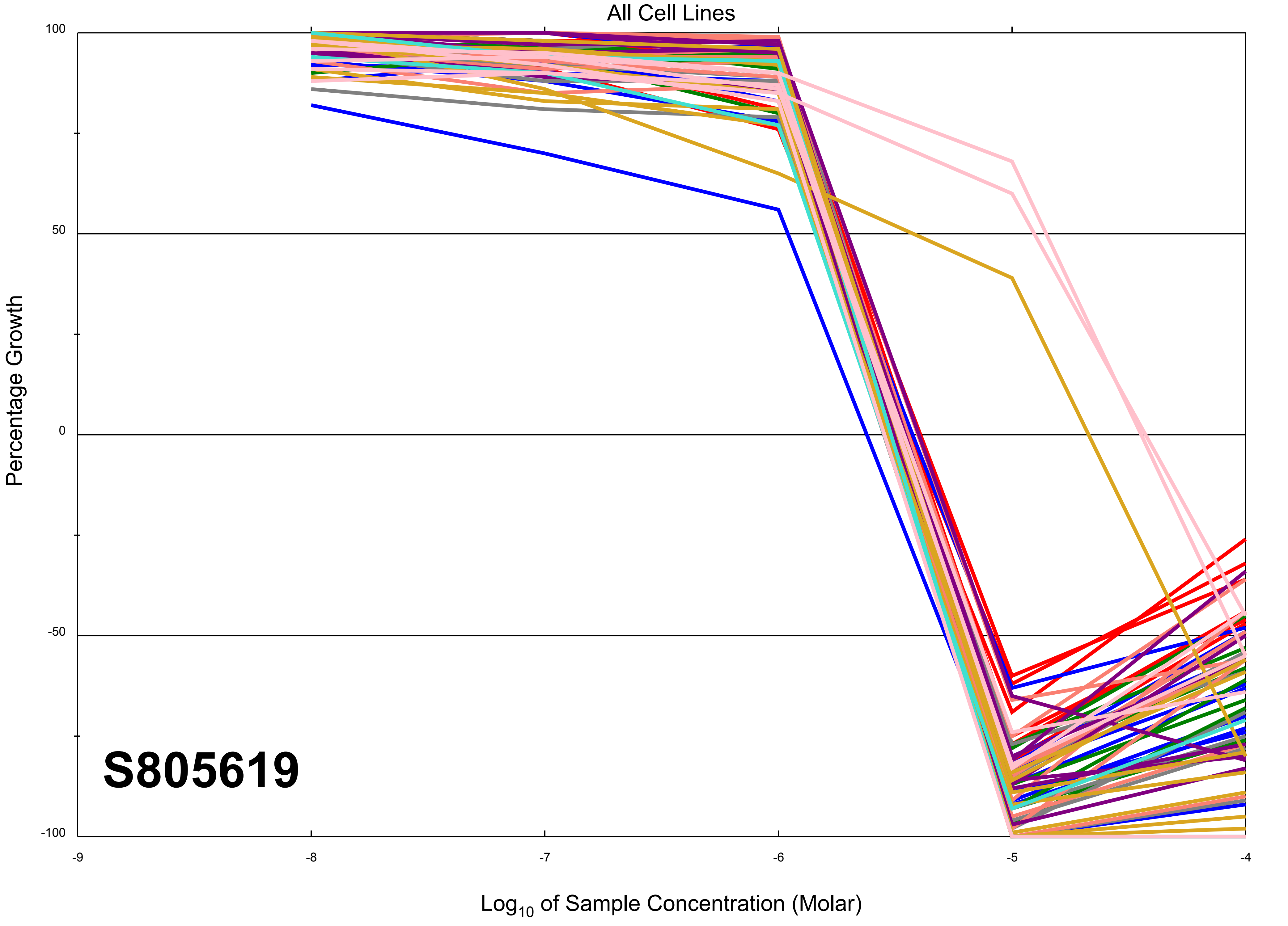

Supplement: Supplementary file 6 — Supplementary Information 6. [file 41598_2024_56313_MOESM6_ESM.pdf]
