# Supplementary material for: Design, synthesis and in vitro anticancer activity of some new lomefloxacin derivatives
Source: Sci Rep. 2024 Mar 14;14:6175. doi: 10.1038/s41598-024-56313-w (PMC10940605; doi:10.1038/s41598-024-56313-w)
Supplement: Supplementary file 21 — Supplementary Information 21. [file 41598_2024_56313_MOESM21_ESM.pdf]

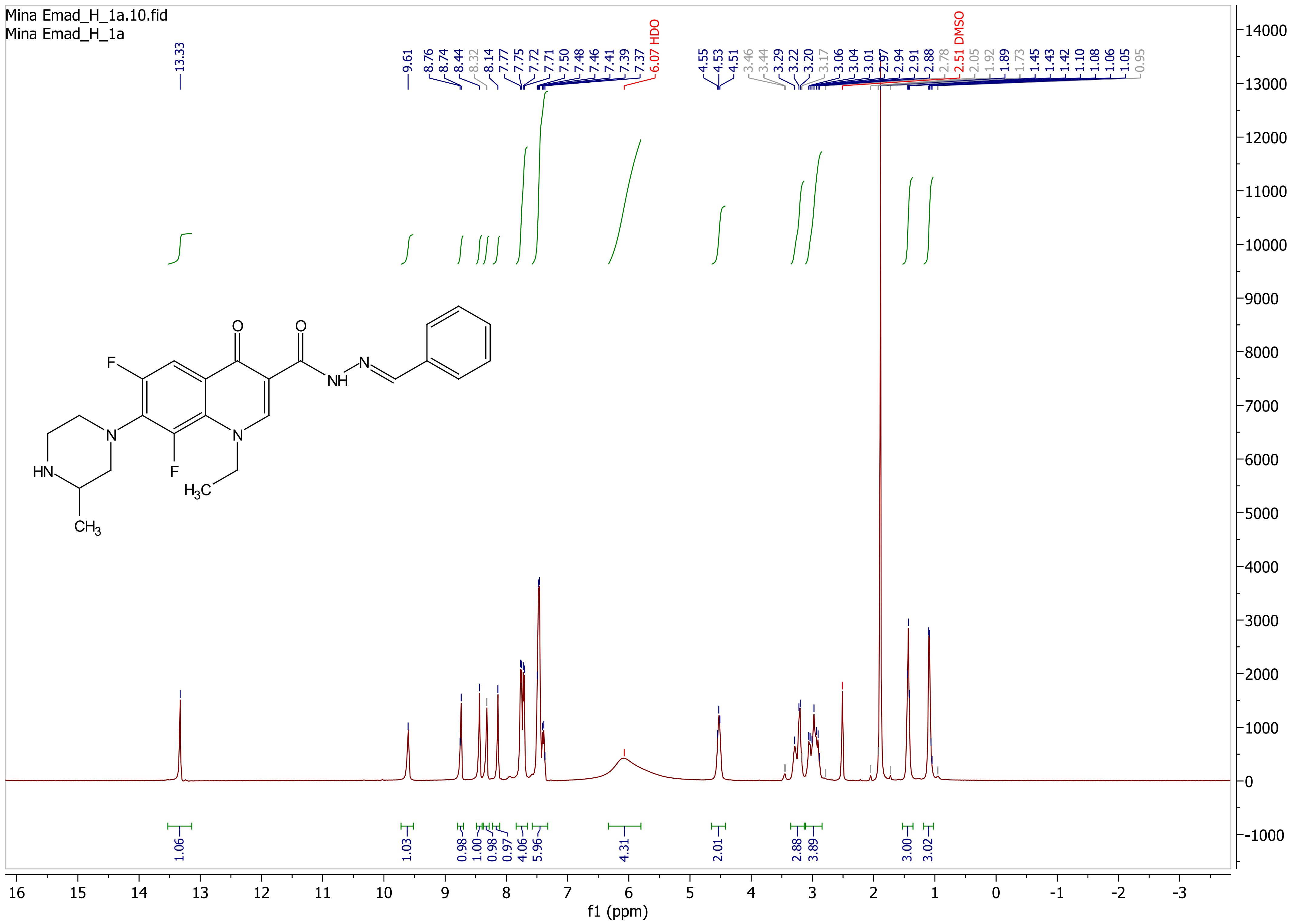
Mina Emad_H_1a.10.fid
Mina Emad_H_1a
13.33
9.61
8.76
8.74
8.44
8.32
8.14
7.77
7.75
7.72
7.71
7.50
7.48
7.46
7.41
7.39
7.37
6.07 HDO
4.55
4.53
4.51
3.46
3.44
3.29
3.22
3.20
3.17
3.06
3.04
3.01
2.97
2.94
2.91
2.88
2.78
2.51 DMSO
2.05
1.92
1.89
1.73
1.45
1.43
1.42
1.10
1.08
1.06
1.05
0.95
1.06
1.03
0.98
1.00
0.98
0.97
4.06
5.96
4.31
2.01
2.88
3.89
3.00
3.02
F
O
O
NH
N
HN
N
F
N
H3C
CH3
f1 (ppm)

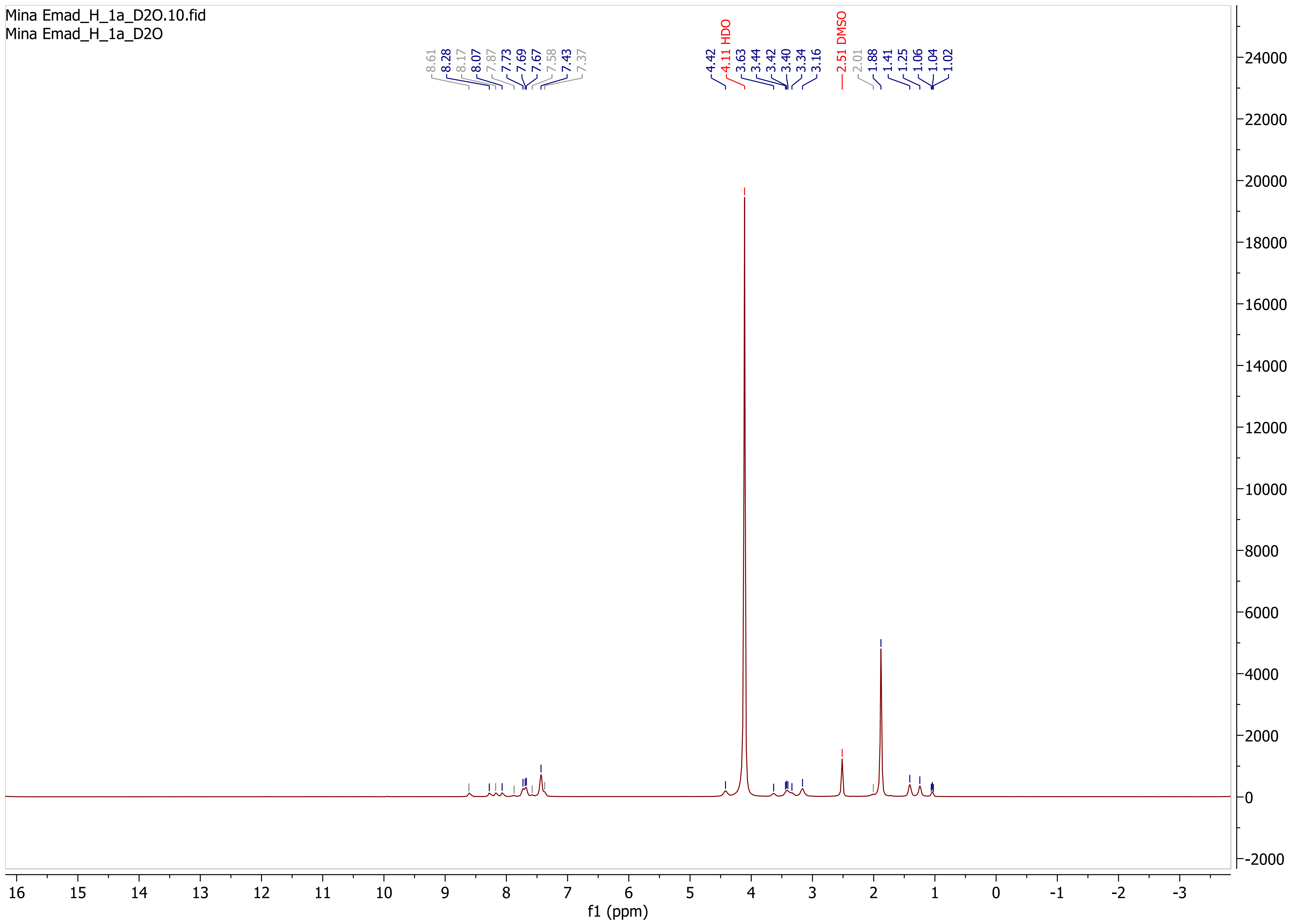
Mina Emad_H_1a_D2O.10.fid
Mina Emad_H_1a_D2O
8.61
8.28
8.17
8.07
7.87
7.73
7.69
7.67
7.58
7.43
7.37
4.42
4.11 HDO
3.63
3.44
3.42
3.40
3.34
3.16
2.51 DMSO
2.01
1.88
1.41
1.25
1.06
1.04
1.02
f1 (ppm)

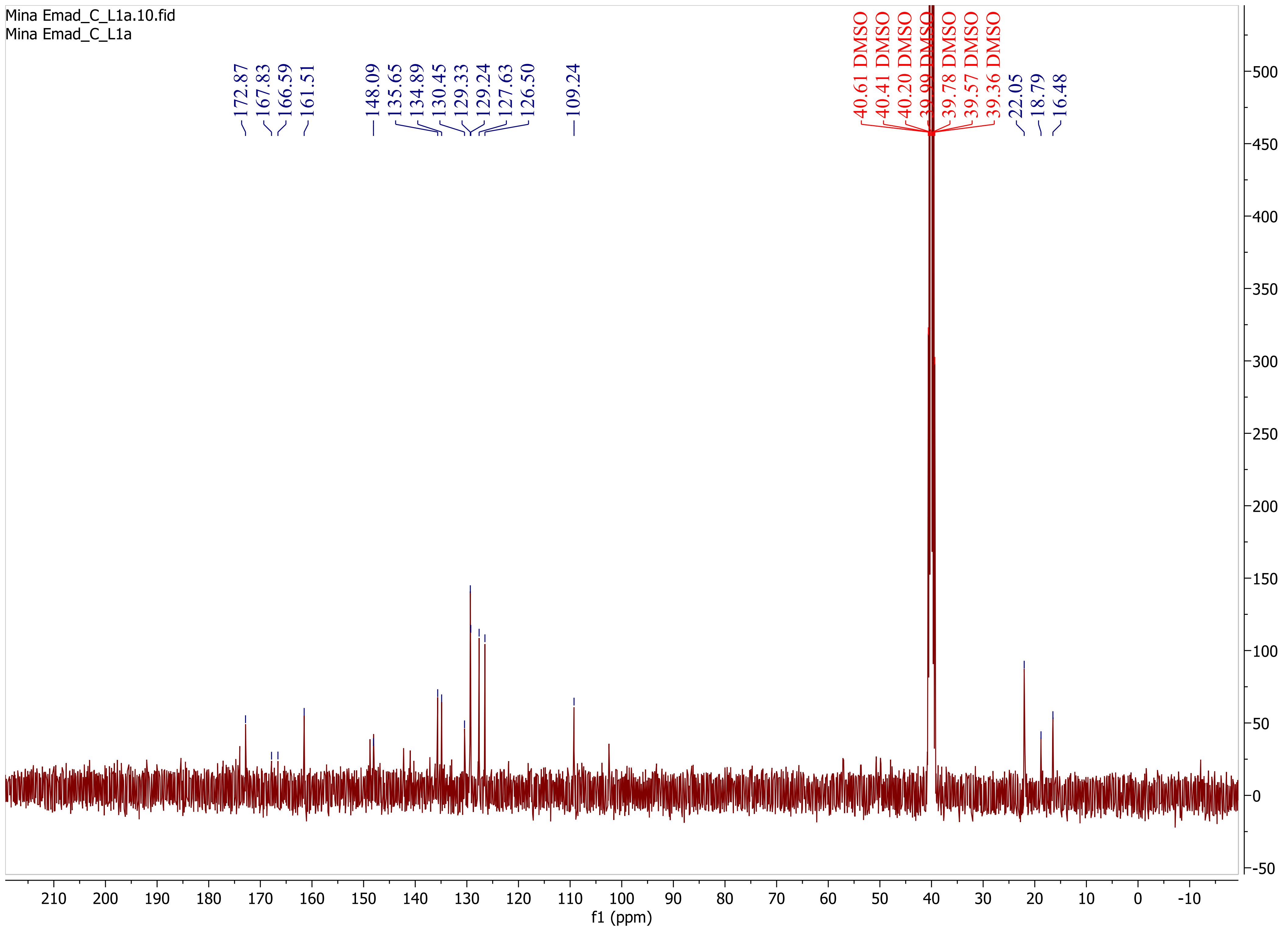

Mina Emad_C_L1a.10.fid
Mina Emad_C_L1a
172.87
167.83
166.59
161.51
148.09
135.65
134.89
130.45
129.33
129.24
127.63
126.50
109.24
40.61 DMSO
40.41 DMSO
40.20 DMSO
39.99 DMSO
39.78 DMSO
39.57 DMSO
39.36 DMSO
22.05
18.79
16.48
f1 (ppm)

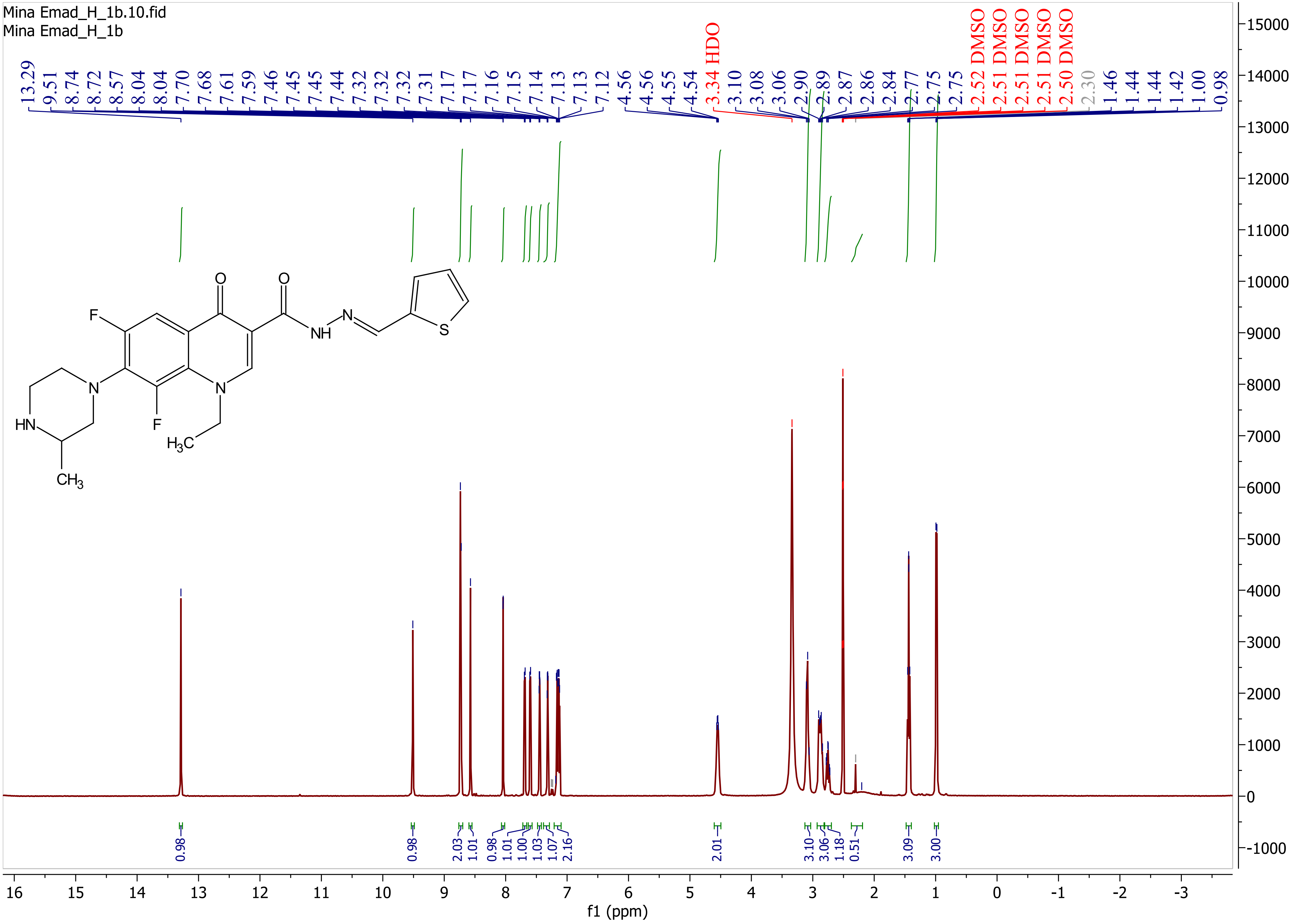

Mina Emad_H_1b.10.fid
Mina Emad_H_1b
13.29 9.51 8.74 8.72 8.57 8.04 8.04 7.70 7.68 7.61 7.59 7.46 7.45 7.45 7.44 7.32 7.32 7.32 7.31 7.17 7.17 7.16 7.15 7.14 7.13 7.13 7.12 4.56 4.56 4.55 4.54
3.34 HDO
3.10 3.08 3.06 2.90 2.89 2.87 2.86 2.84 2.77 2.75 2.75
2.52 DMSO 2.51 DMSO 2.51 DMSO 2.51 DMSO 2.50 DMSO
2.30 1.46 1.44 1.44 1.42 1.00 0.98
0.98 0.98 2.03 1.01 0.98 1.01 1.00 1.03 1.07 2.16 2.01 3.10 3.06 1.18 0.51 3.09 3.00
F F O O NH N S N N HN CH3 H3C
f1 (ppm)

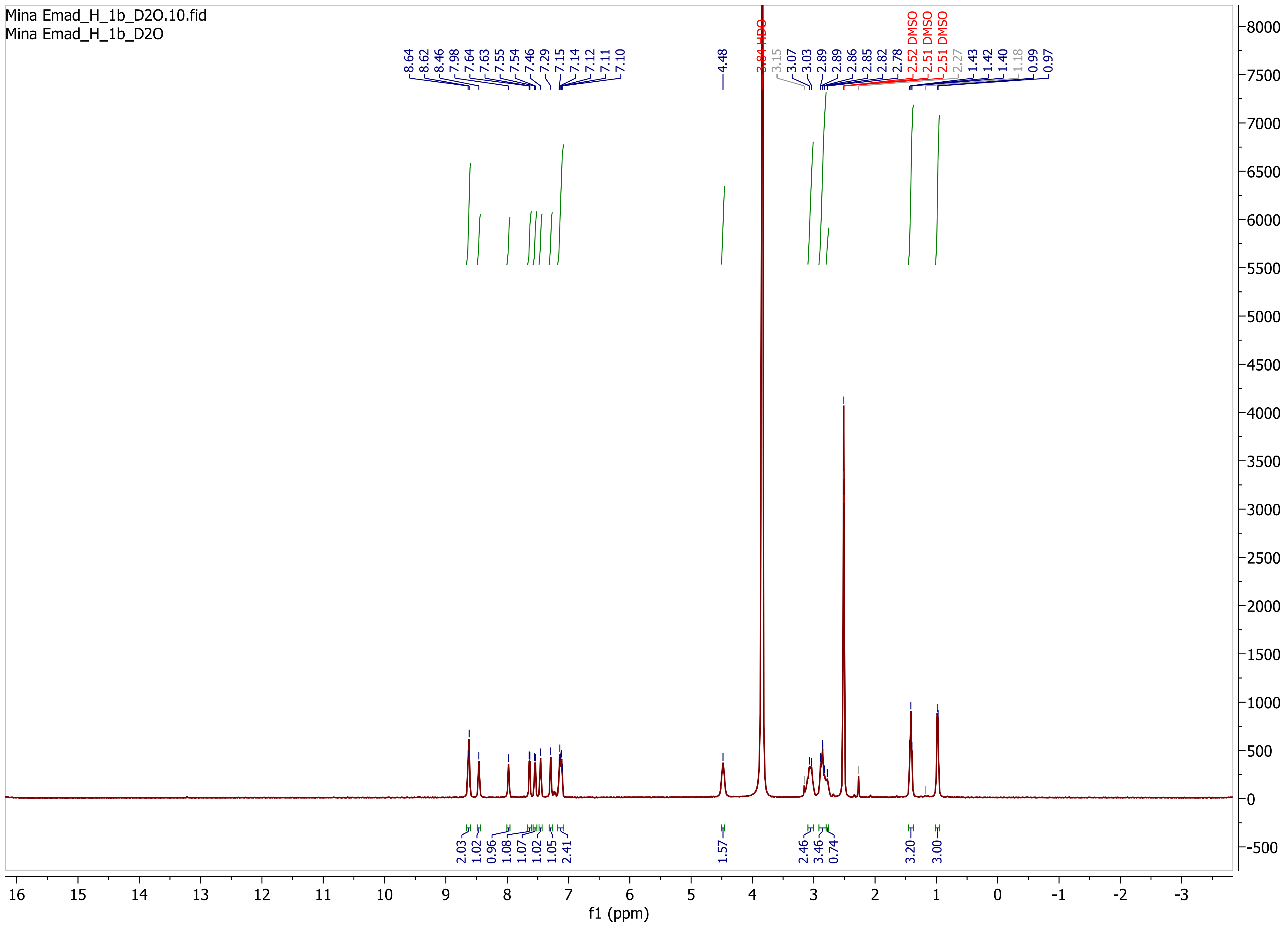
Mina Emad_H_1b_D2O.10.fid
Mina Emad_H_1b_D2O
8.64
8.62
8.46
7.98
7.64
7.63
7.55
7.54
7.46
7.29
7.15
7.14
7.12
7.11
7.10
4.48
3.84 HDO
3.15
3.07
3.03
2.89
2.89
2.86
2.85
2.82
2.78
2.52 DMSO
2.51 DMSO
2.51 DMSO
2.27
1.43
1.42
1.40
1.18
0.99
0.97
2.03
1.02
0.96
1.08
1.07
1.02
1.05
2.41
1.57
2.46
3.46
0.74
3.20
3.00
f1 (ppm)

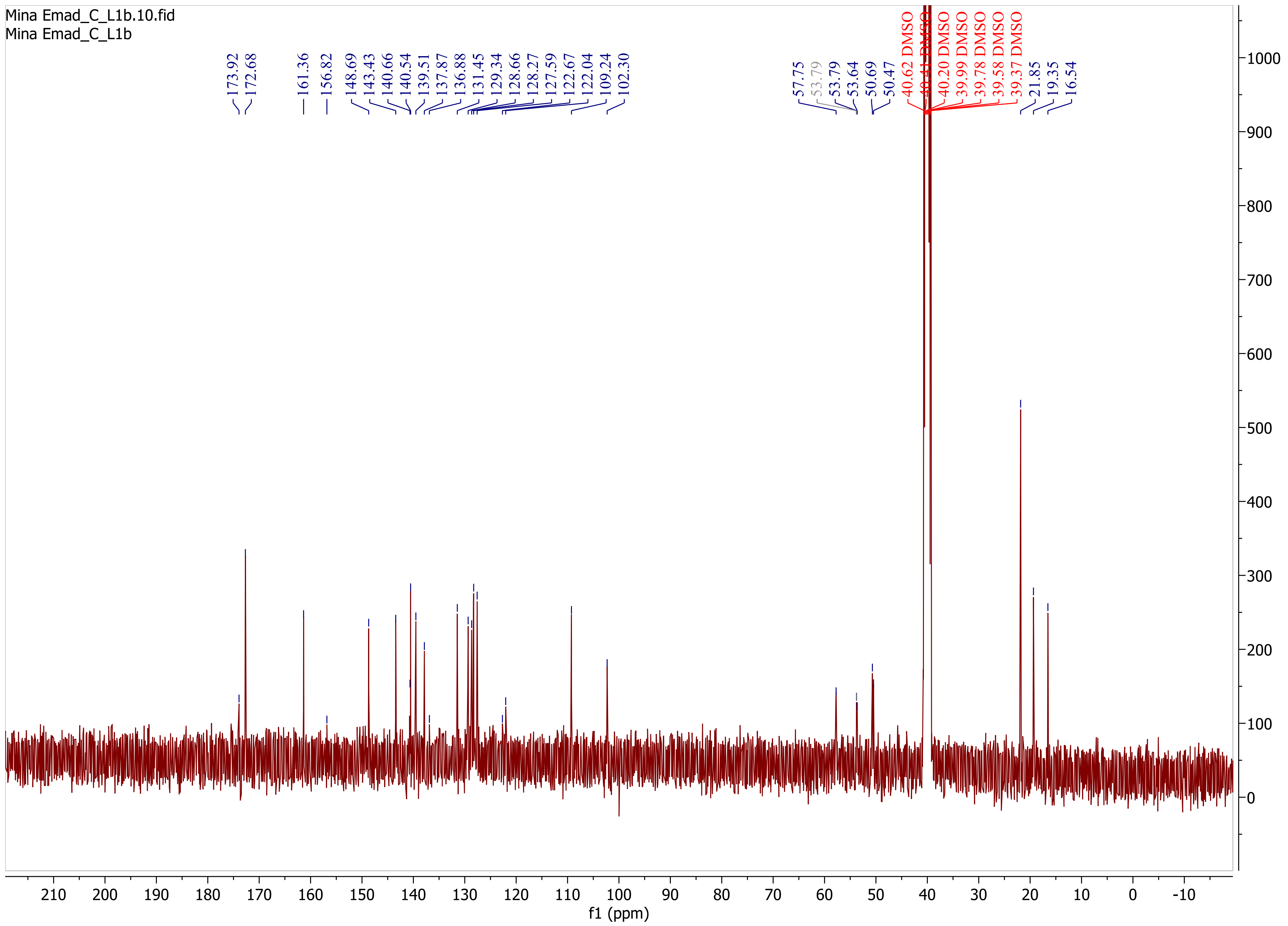
Mina Emad_C_L1b.10.fid
Mina Emad_C_L1b
173.92
172.68
161.36
156.82
148.69
143.43
140.66
140.54
139.51
137.87
136.88
131.45
129.34
128.66
128.27
127.59
122.67
122.04
109.24
102.30
57.75
53.79
53.79
53.64
50.69
50.47
40.62 DMSO
40.41 DMSO
40.20 DMSO
39.99 DMSO
39.78 DMSO
39.58 DMSO
39.37 DMSO
21.85
19.35
16.54
f1 (ppm)

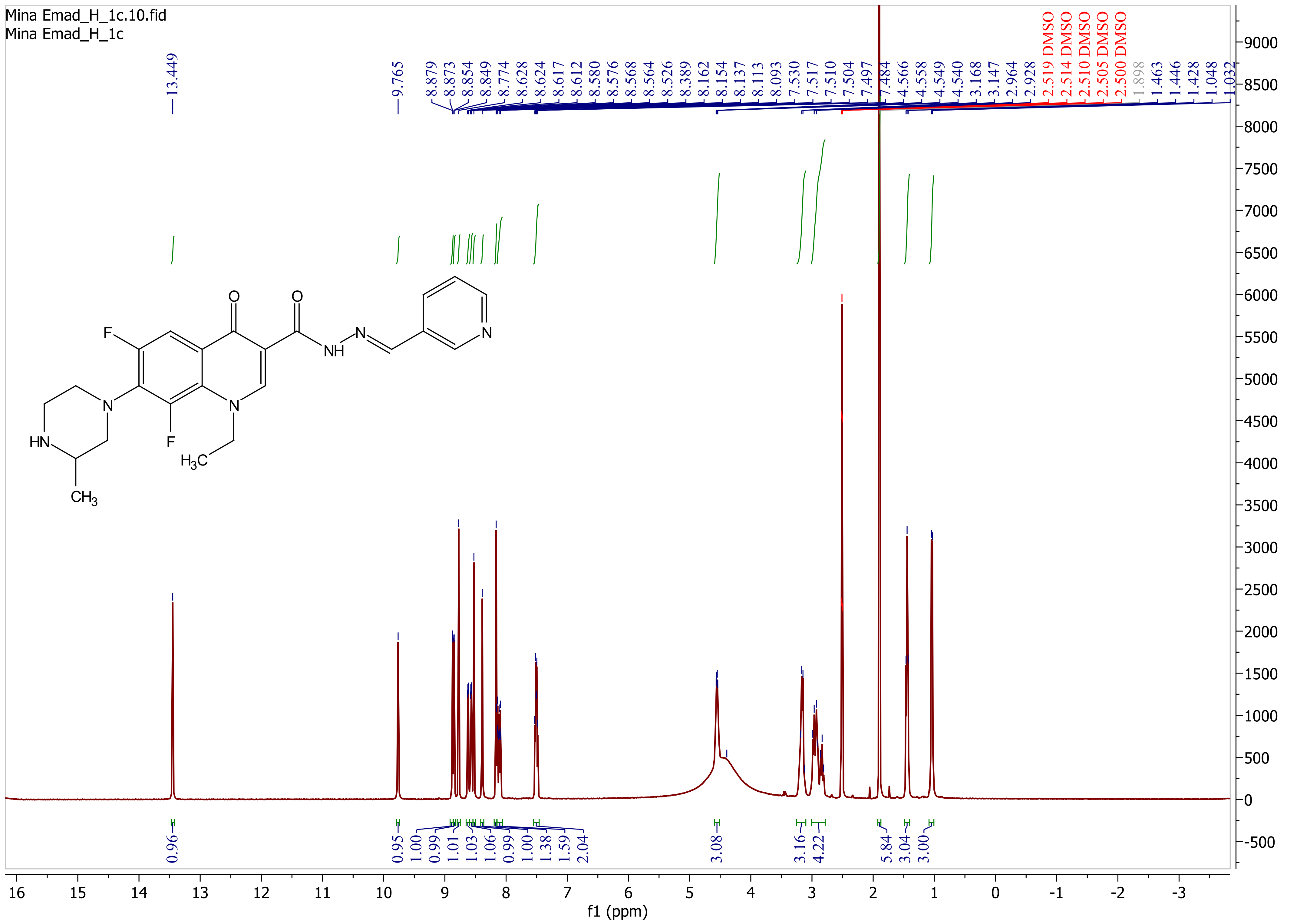
Mina Emad_H_1c.10.fid
Mina Emad_H_1c
13.449
9.765
8.879
8.873
8.854
8.849
8.774
8.628
8.624
8.617
8.612
8.580
8.576
8.568
8.564
8.526
8.389
8.162
8.154
8.137
8.113
8.093
7.530
7.517
7.510
7.504
7.497
7.484
4.566
4.558
4.549
4.540
3.168
3.147
2.964
2.928
2.519 DMSO
2.514 DMSO
2.510 DMSO
2.505 DMSO
2.500 DMSO
1.898
1.463
1.446
1.428
1.048
1.032
0.96
0.95
1.00
0.99
1.01
1.03
1.06
0.99
1.00
1.38
1.59
2.04
3.08
3.16
4.22
5.84
3.04
3.00
F
O
O
N
NH
N
N
HN
N
F
H_3C
CH_3
f1 (ppm)

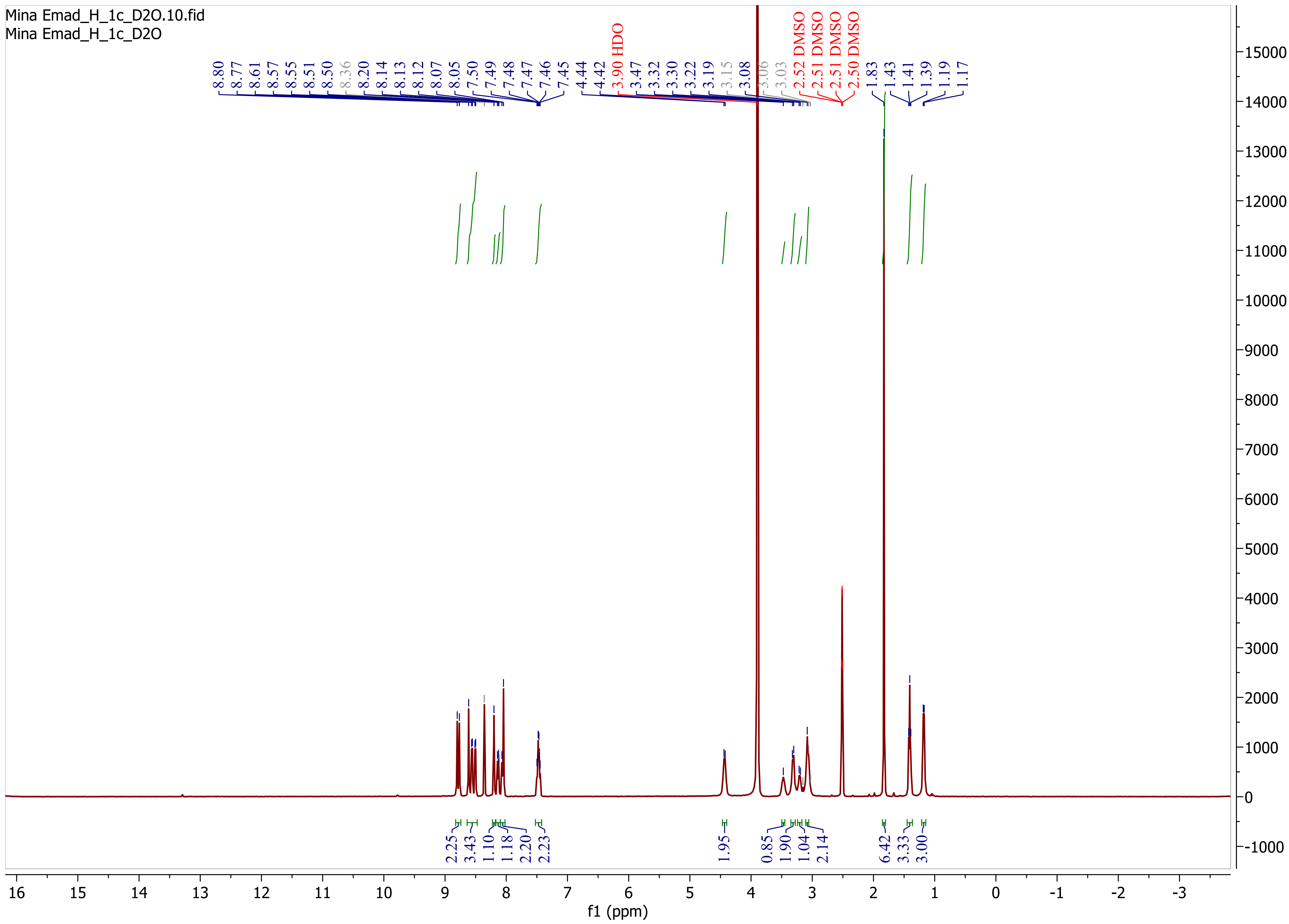
Mina Emad_H_1c_D2O.10.fid
Mina Emad_H_1c_D2O
8.80 8.77 8.61 8.57 8.55 8.51 8.50 8.36 8.20 8.14 8.13 8.12 8.07 8.05 7.50 7.49 7.48 7.47 7.46 7.45 4.44 4.42 3.90 HDO 3.47 3.32 3.30 3.22 3.19 3.15 3.08 3.06 3.03 2.52 DMSO 2.51 DMSO 2.51 DMSO 2.50 DMSO 1.83 1.43 1.41 1.39 1.19 1.17
2.25 3.43 1.10 1.18 2.20 2.23 1.95 0.85 1.90 1.04 2.14 6.42 3.33 3.00
f1 (ppm)

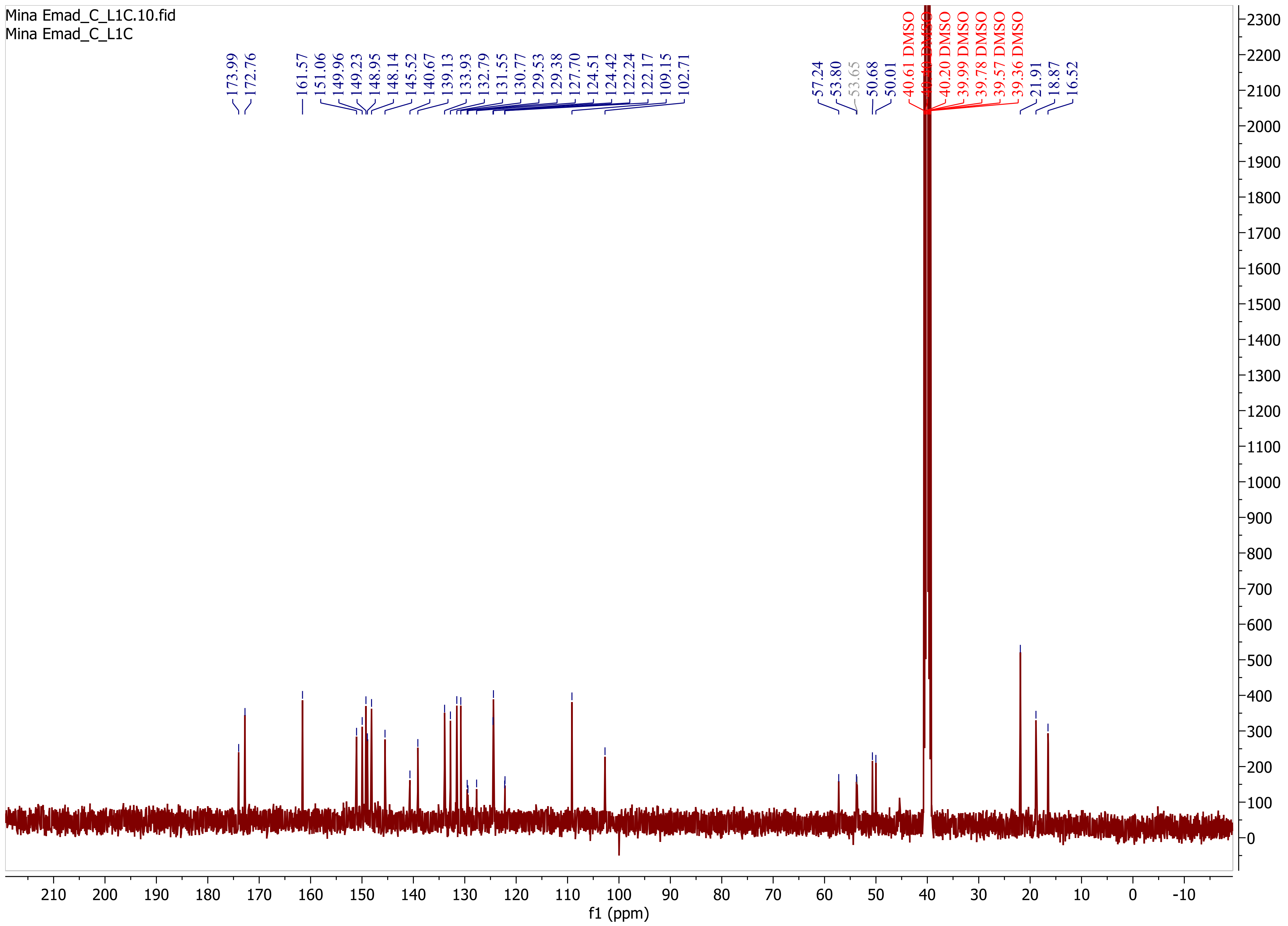
Mina Emad_C_L1C.10.fid
Mina Emad_C_L1C
173.99
172.76
161.57
151.06
149.96
149.23
148.95
148.14
145.52
140.67
139.13
133.93
132.79
131.55
130.77
129.53
129.38
127.70
124.51
124.42
122.24
122.17
109.15
102.71
57.24
53.80
53.65
50.68
50.01
40.61 DMSO
40.40 DMSO
40.20 DMSO
39.99 DMSO
39.78 DMSO
39.57 DMSO
39.36 DMSO
21.91
18.87
16.52
f1 (ppm)

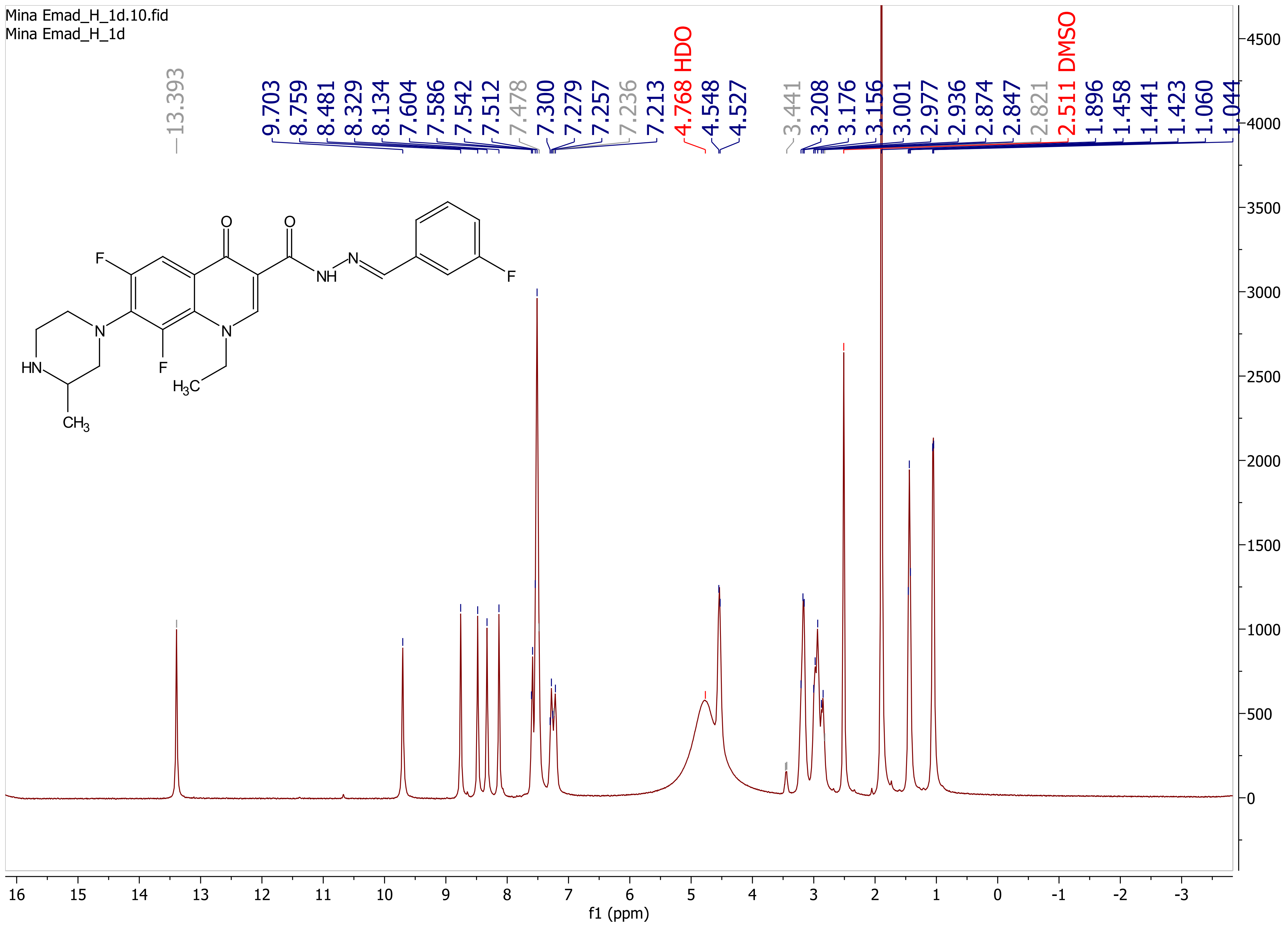
Mina Emad_H_1d.10.fid
Mina Emad_H_1d
13.393
9.703
8.759
8.481
8.329
8.134
7.604
7.586
7.542
7.512
7.478
7.300
7.279
7.257
7.236
7.213
4.768 HDO
4.548
4.527
3.441
3.208
3.176
3.156
3.001
2.977
2.936
2.874
2.847
2.821
2.511 DMSO
1.896
1.458
1.441
1.423
1.060
1.044
f1 (ppm)

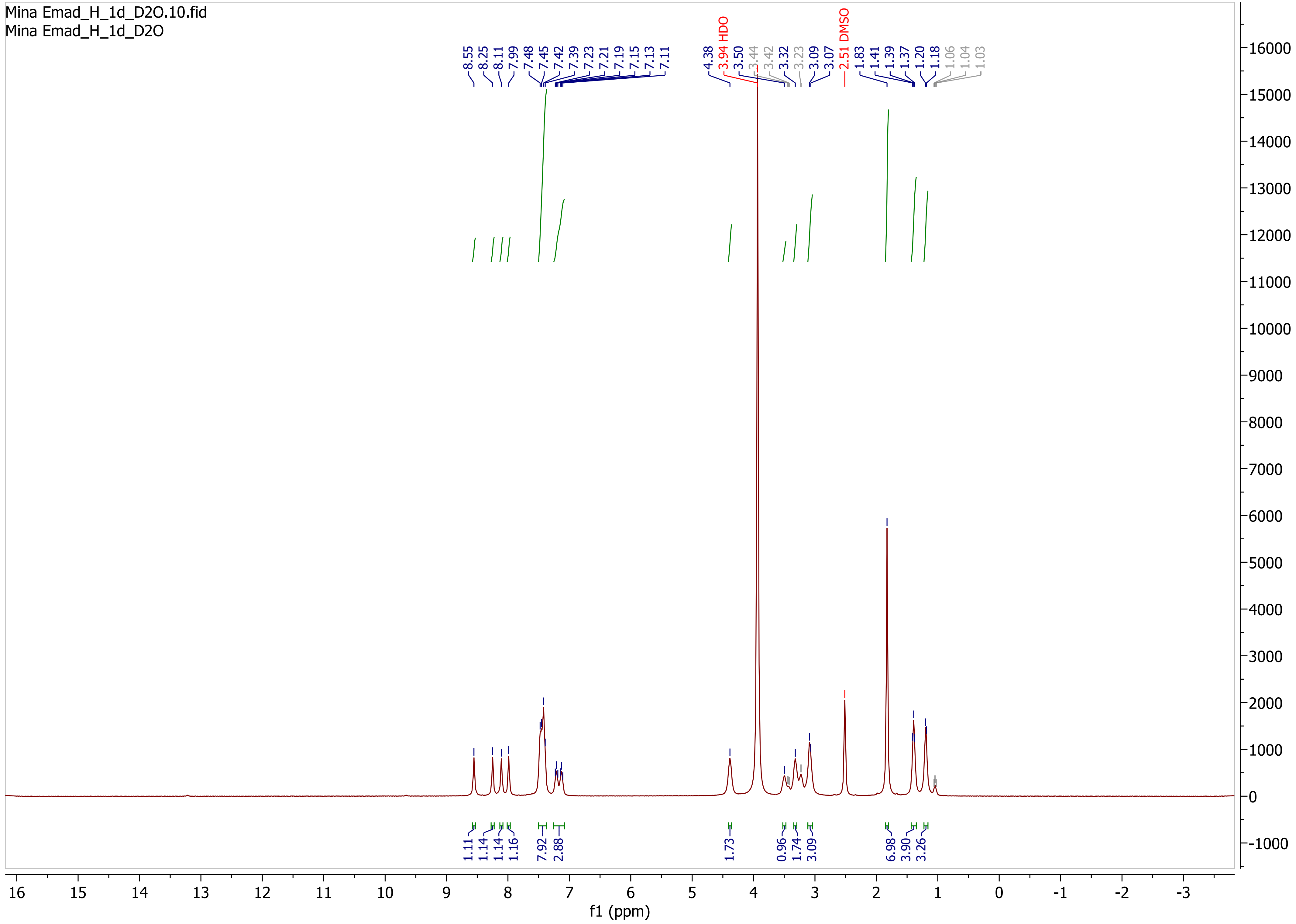
Mina Emad_H_1d_D2O.10.fid
Mina Emad_H_1d_D2O
8.55
8.25
8.11
7.99
7.48
7.45
7.42
7.39
7.23
7.21
7.19
7.15
7.13
7.11
4.38
3.94 HDO
3.50
3.44
3.42
3.32
3.23
3.09
3.07
2.51 DMSO
1.83
1.41
1.39
1.37
1.20
1.18
1.06
1.04
1.03
1.11
1.14
1.14
1.16
7.92
2.88
1.73
0.96
1.74
3.09
6.98
3.90
3.26
f1 (ppm)

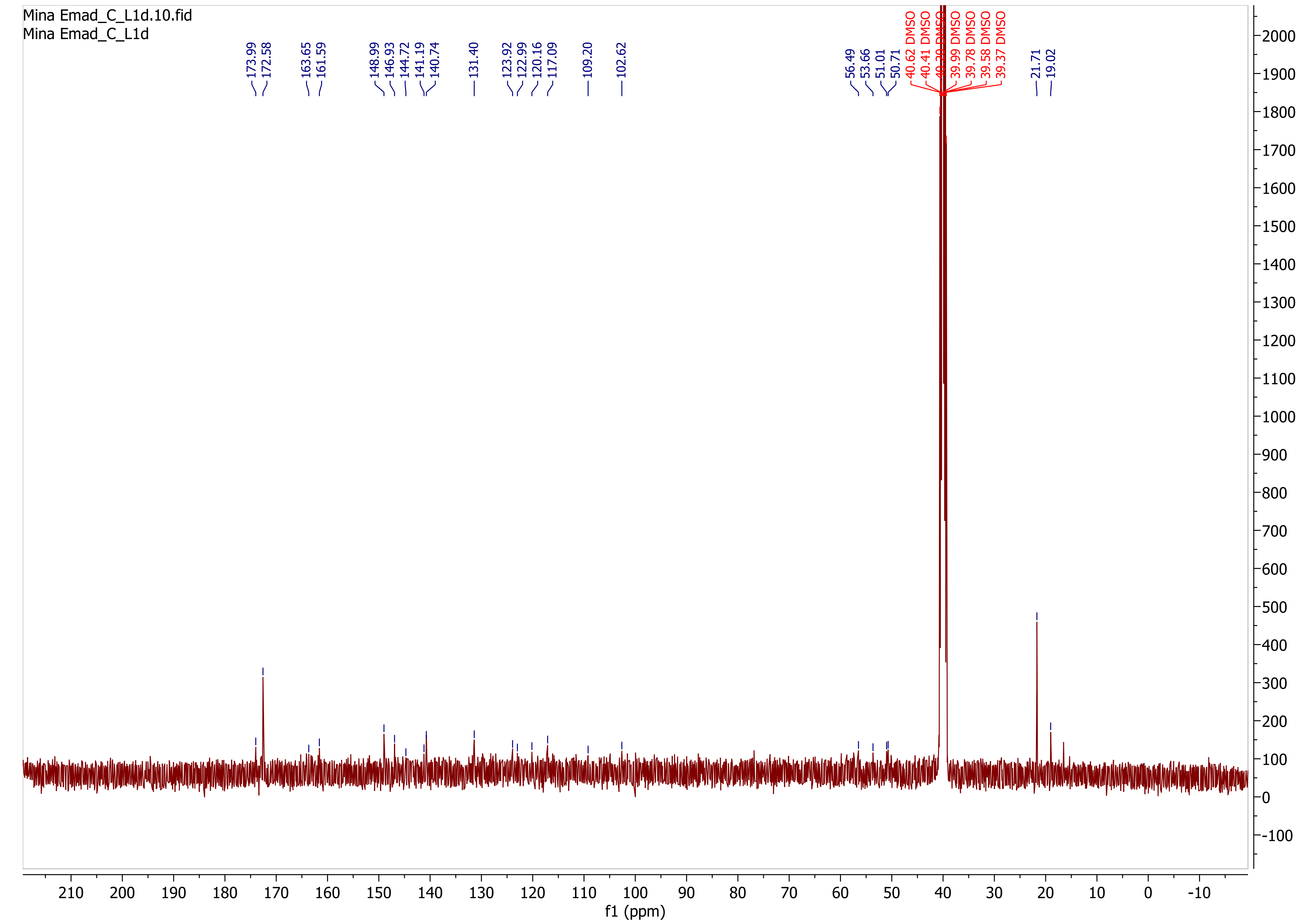

Mina Emad_C_L1d.10.fid
Mina Emad_C_L1d
173.99
172.58
163.65
161.59
148.99
146.93
144.72
141.19
140.74
131.40
123.92
122.99
120.16
117.09
109.20
102.62
56.49
53.66
51.01
50.71
40.62 DMSO
40.41 DMSO
39.99 DMSO
39.78 DMSO
39.58 DMSO
39.37 DMSO
21.71
19.02
2000
1900
1800
1700
1600
1500
1400
1300
1200
1100
1000
900
800
700
600
500
400
300
200
100
0
-100
210
200
190
180
170
160
150
140
130
120
110
100
90
80
70
60
50
40
30
20
10
0
-10
f1 (ppm)

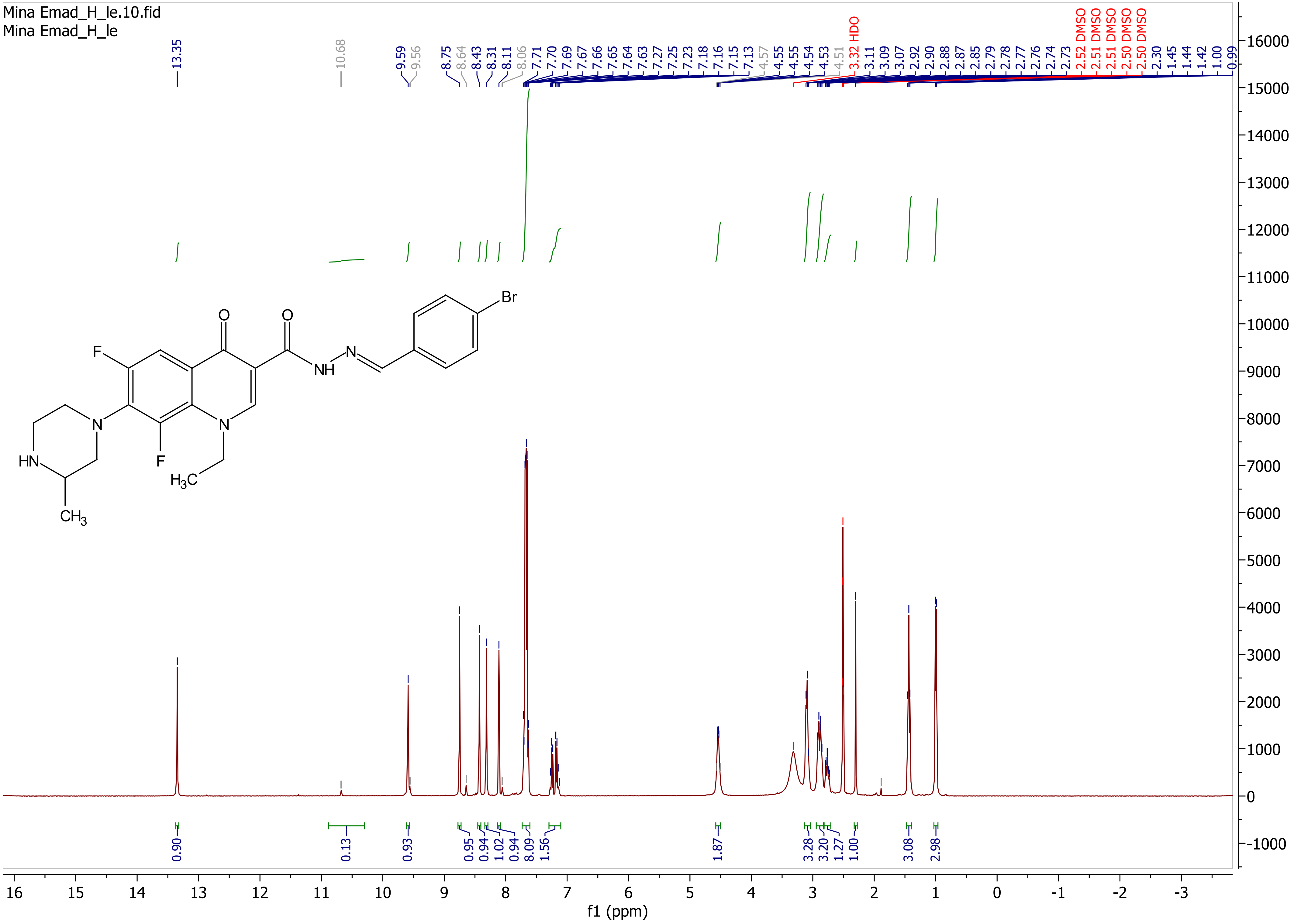
Mina Emad_H_le.10.fid
Mina Emad_H_le
13.35
10.68
9.59 9.56
8.75 8.64 8.43 8.31 8.11 8.06 7.71 7.70 7.69 7.67 7.66 7.65 7.64 7.63 7.27 7.25 7.23 7.18 7.16 7.15 7.13
4.57 4.55 4.55 4.54 4.53 4.51
3.32 HDO
3.11 3.09 3.07 2.92 2.90 2.88 2.87 2.85 2.79 2.78 2.77 2.76 2.74 2.73
2.52 DMSO 2.51 DMSO 2.50 DMSO 2.50 DMSO
2.30 1.45 1.44 1.42 1.00 0.99
0.90 0.13 0.93 0.95 0.94 1.02 0.94 8.09 1.56 1.87 3.28 3.20 1.27 1.00 3.08 2.98
f1 (ppm)
Br
O
O
F
NH
N
N
N
HN
F
H_3C
CH_3

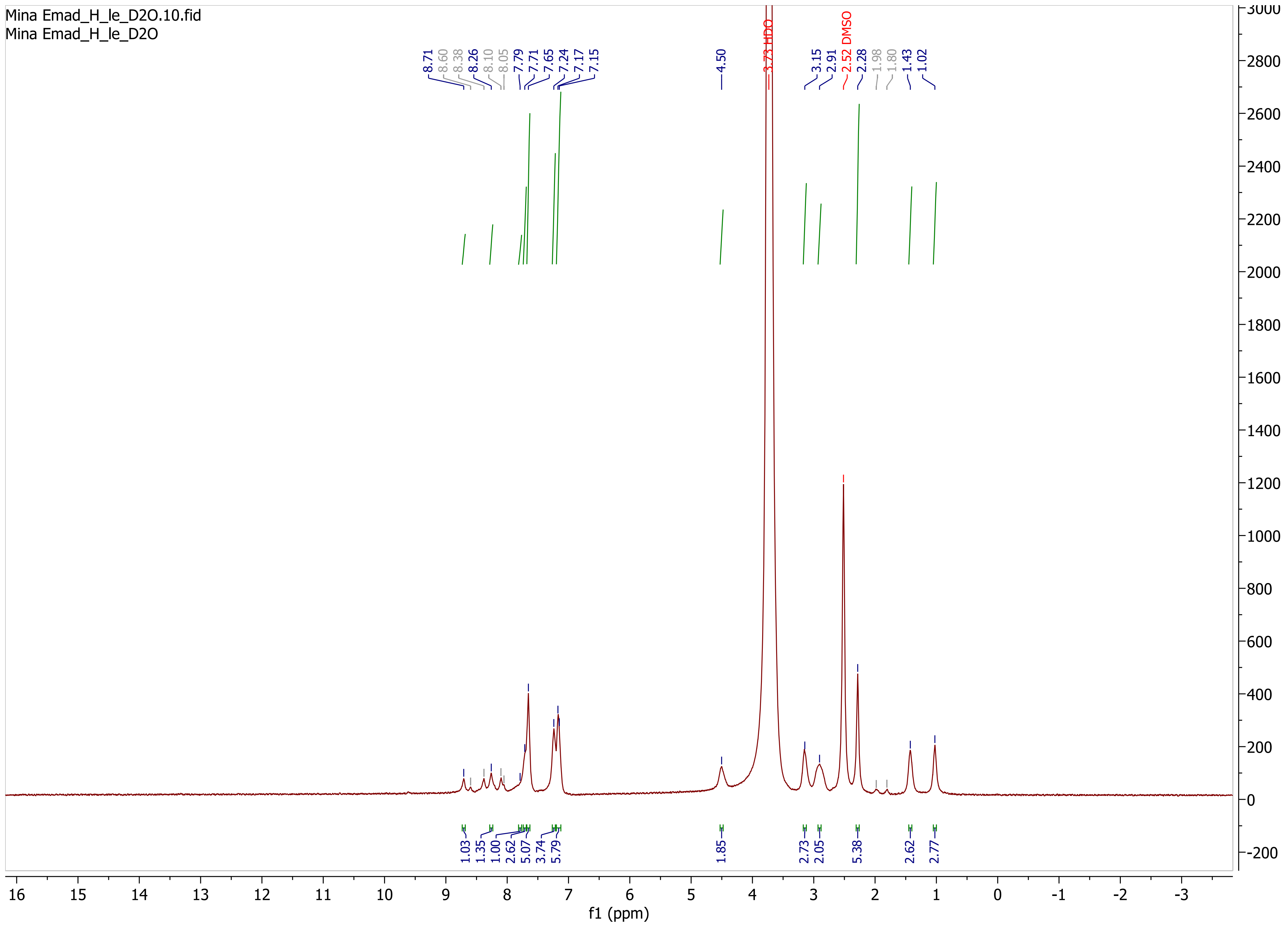
Mina Emad_H_le_D2O.10.fid
Mina Emad_H_le_D2O
8.71
8.60
8.38
8.26
8.10
8.05
7.79
7.71
7.65
7.24
7.17
7.15
4.50
3.73 HDO
3.15
2.91
2.52 DMSO
2.28
1.98
1.80
1.43
1.02
1.03
1.35
1.00
2.62
5.07
3.74
5.79
1.85
2.73
2.05
5.38
2.62
2.77
f1 (ppm)

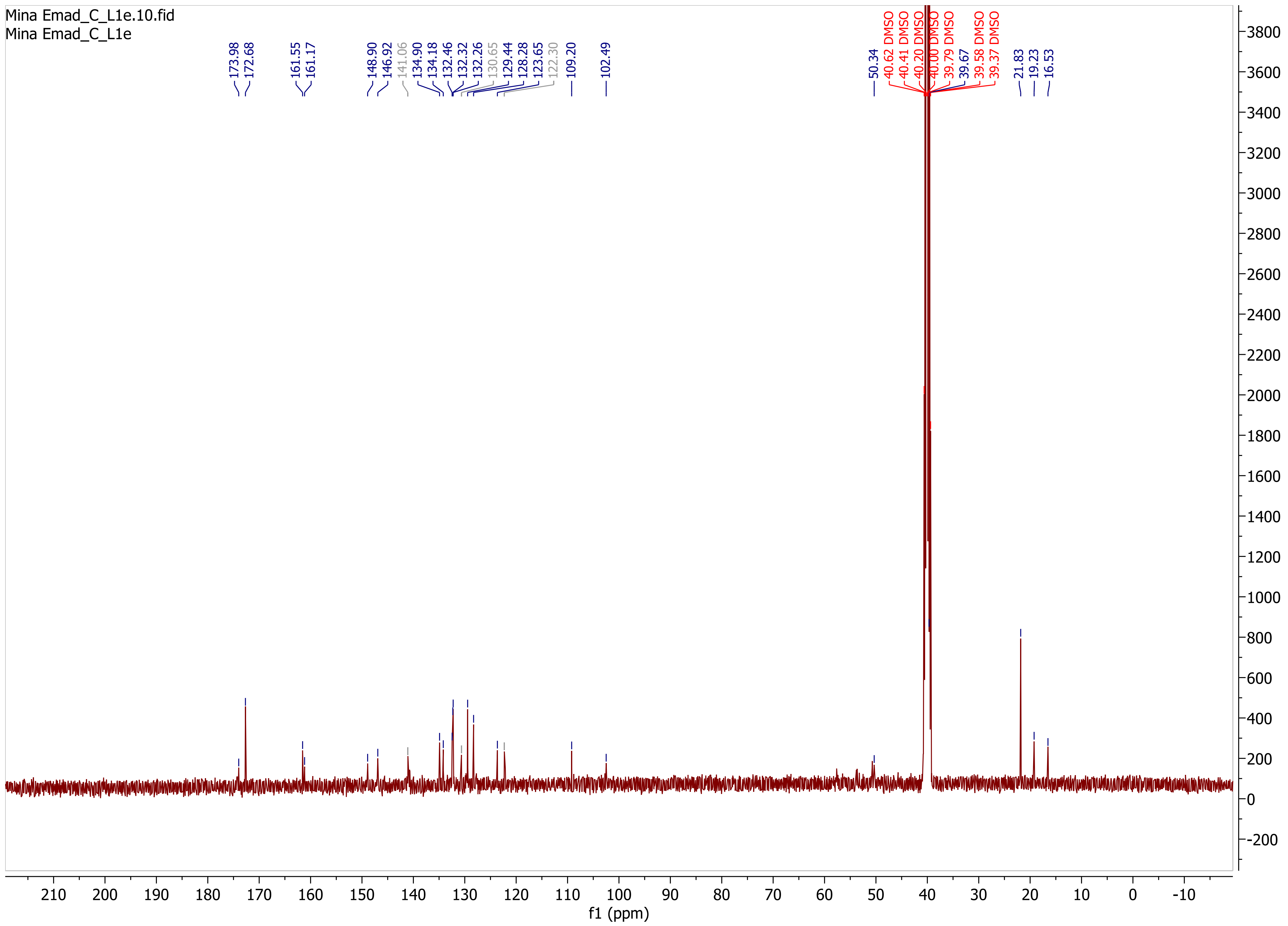
Mina Emad_C_L1e.10.fid
Mina Emad_C_L1e
173.98
172.68
161.55
161.17
148.90
146.92
141.06
134.90
134.18
132.46
132.32
132.26
130.65
129.44
128.28
123.65
122.30
109.20
102.49
50.34
40.62 DMSO
40.41 DMSO
40.20 DMSO
40.00 DMSO
39.79 DMSO
39.67
39.58 DMSO
39.37 DMSO
21.83
19.23
16.53
f1 (ppm)

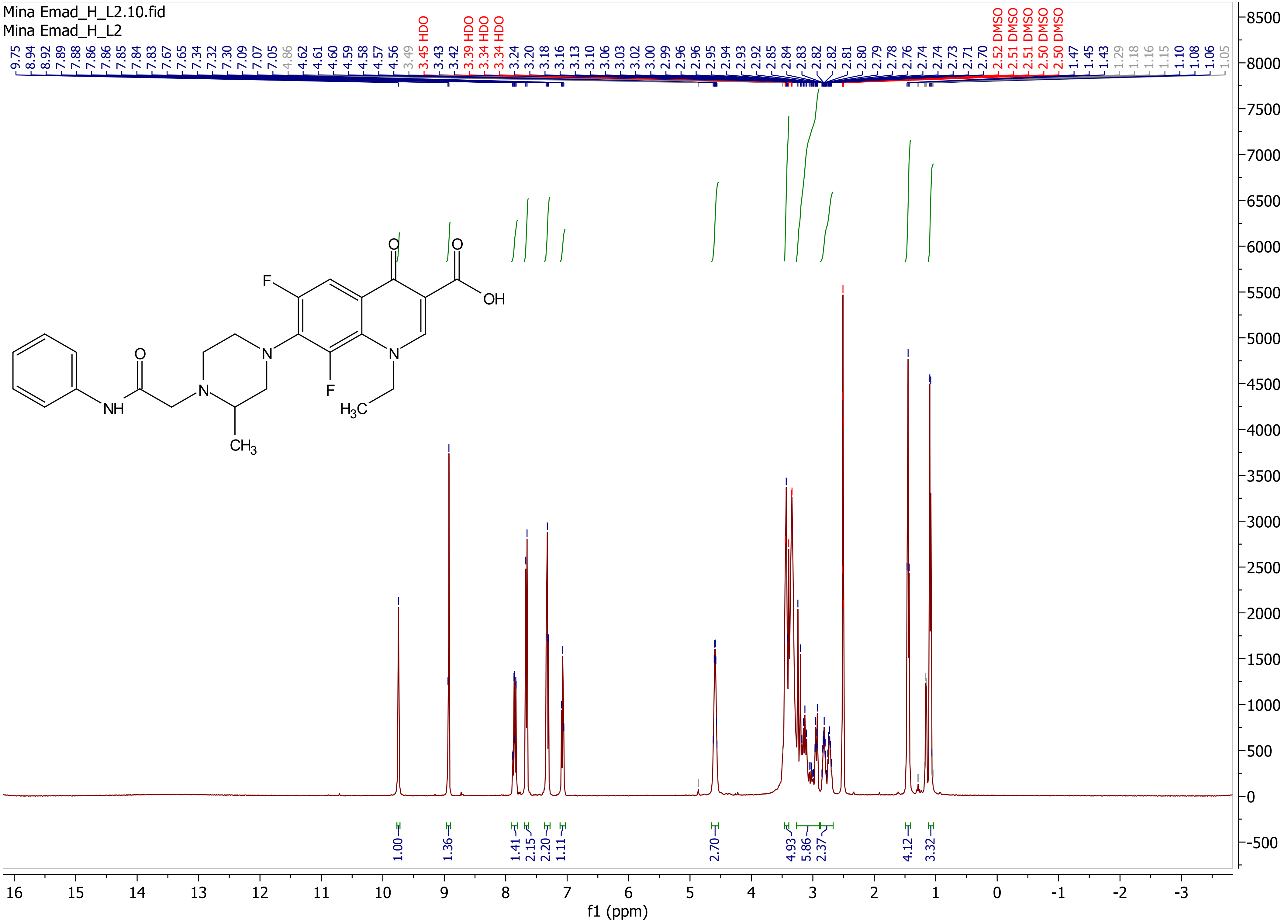

Mina Emad_H_L2.10.fid
Mina Emad_H_L2
9.75 8.94 8.92 7.89 7.88 7.86 7.86 7.85 7.84 7.83 7.67 7.65 7.34 7.32 7.30 7.09 7.07 7.05 4.86 4.62 4.61 4.60 4.59 4.58 4.57 4.56 3.49 3.45 HDO 3.43 3.42 3.39 HDO 3.34 HDO 3.34 HDO 3.24 3.20 3.18 3.16 3.13 3.10 3.06 3.03 3.02 3.00 2.99 2.96 2.96 2.95 2.94 2.93 2.92 2.85 2.84 2.83 2.82 2.82 2.82 2.81 2.80 2.79 2.78 2.76 2.74 2.74 2.73 2.71 2.70 2.52 DMSO 2.51 DMSO 2.51 DMSO 2.50 DMSO 2.50 DMSO 1.47 1.45 1.43 1.29 1.18 1.16 1.15 1.10 1.08 1.06 1.05
1.00 1.36 1.41 2.15 2.20 1.11 2.70 4.93 5.86 2.37 4.12 3.32
f1 (ppm)

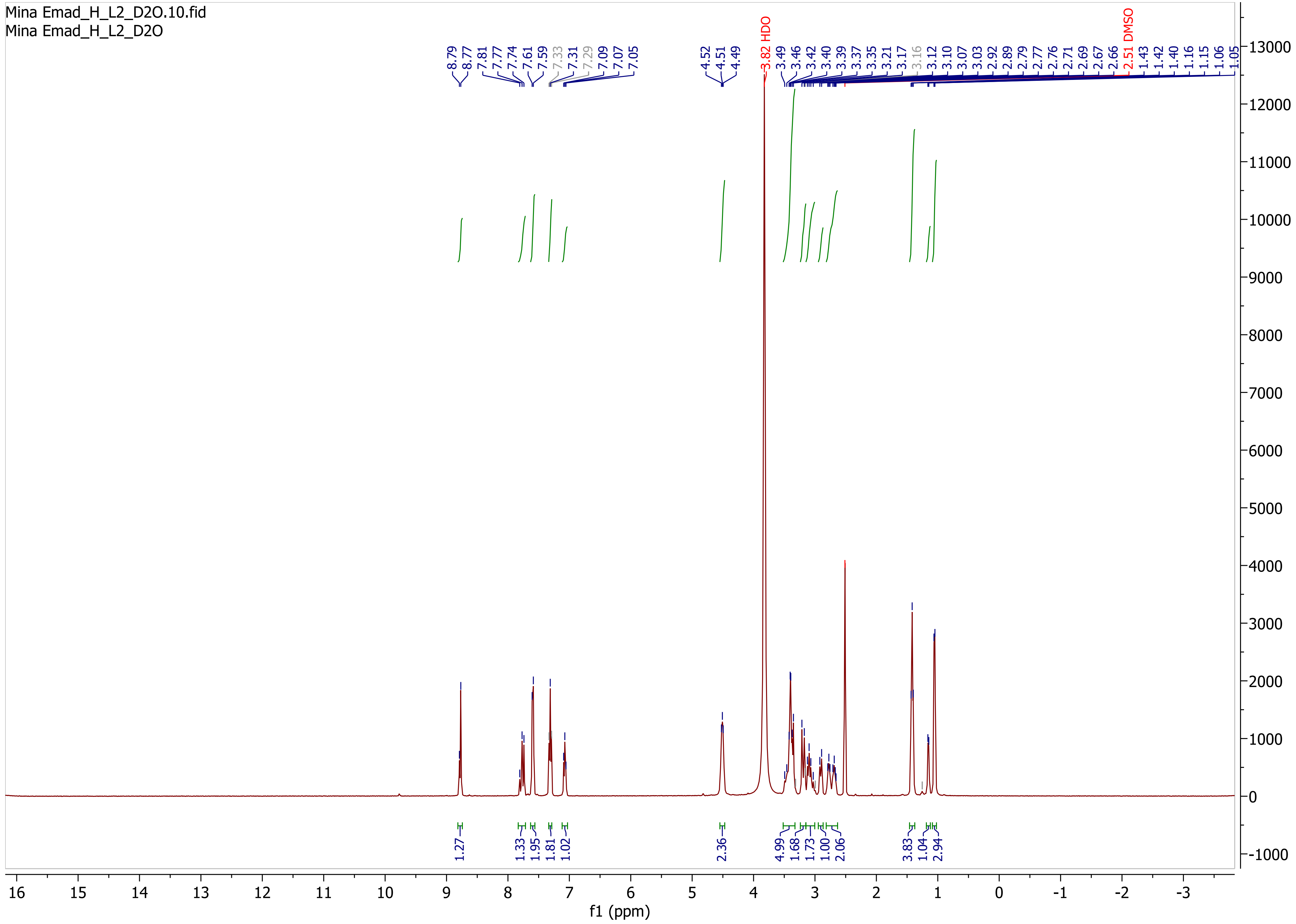

Mina Emad_H_L2_D2O.10.fid
Mina Emad_H_L2_D2O
8.79 8.77 7.81 7.77 7.74 7.61 7.59 7.33 7.31 7.29 7.09 7.07 7.05
4.52 4.51 4.49
3.82 HDO
3.49 3.46 3.42 3.40 3.39 3.37 3.35 3.21 3.17 3.16 3.12 3.10 3.07 3.03 2.92 2.89 2.79 2.77 2.76 2.71 2.69 2.67 2.66
2.51 DMSO
1.43 1.42 1.40 1.16 1.15 1.06 1.05
1.27 1.33 1.95 1.81 1.02 2.36 4.99 1.68 1.73 1.00 2.06 3.83 1.04 2.94
f1 (ppm)

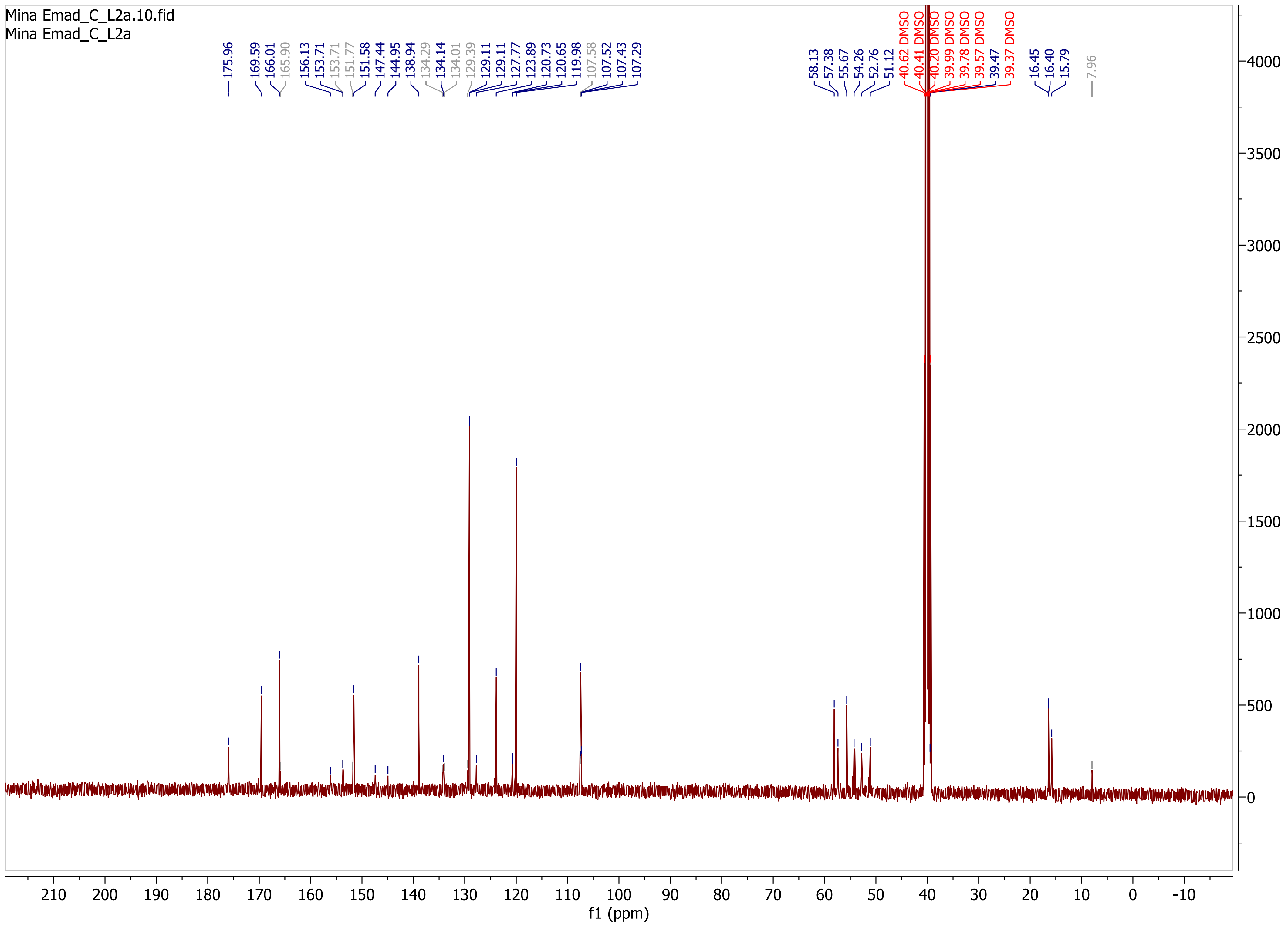
Mina Emad_C_L2a.10.fid
Mina Emad_C_L2a
175.96
169.59
166.01
165.90
156.13
153.71
153.71
151.77
151.58
147.44
144.95
138.94
134.29
134.14
134.01
129.39
129.11
129.11
127.77
123.89
120.73
120.65
119.98
107.58
107.52
107.43
107.29
58.13
57.38
55.67
54.26
52.76
51.12
40.62 DMSO
40.41 DMSO
40.20 DMSO
39.99 DMSO
39.78 DMSO
39.57 DMSO
39.47
39.37 DMSO
16.45
16.40
15.79
7.96
f1 (ppm)

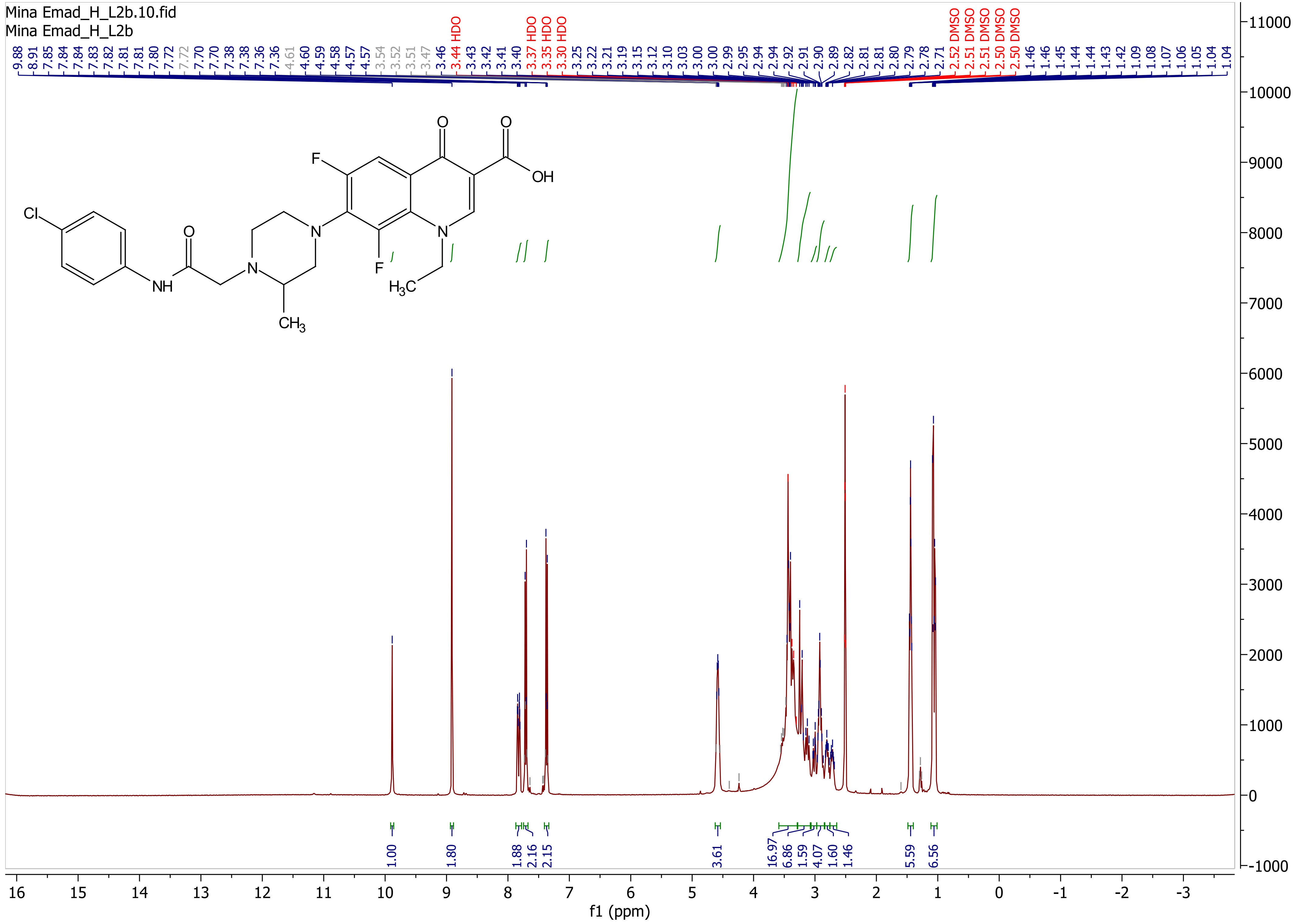
Mina Emad_H_L2b.10.fid
Mina Emad_H_L2b
9.88 8.91 7.85 7.84 7.84 7.83 7.82 7.81 7.81 7.80 7.72 7.72 7.70 7.70 7.38 7.38 7.36 7.36 4.61 4.60 4.59 4.58 4.57 4.57 3.54 3.52 3.51 3.47 3.46 3.44 HDO 3.43 3.42 3.41 3.40 3.37 HDO 3.35 HDO 3.30 HDO 3.25 3.22 3.21 3.19 3.15 3.12 3.10 3.03 3.00 3.00 2.99 2.95 2.94 2.94 2.92 2.91 2.90 2.89 2.82 2.81 2.80 2.79 2.78 2.71 2.52 DMSO 2.51 DMSO 2.51 DMSO 2.50 DMSO 2.50 DMSO 1.46 1.46 1.45 1.44 1.44 1.43 1.42 1.09 1.08 1.07 1.06 1.05 1.04 1.04
Cl
NH
O
N
N
CH3
F
F
O
O
OH
N
H3C
1.00 1.80 1.88 2.16 2.15 3.61 16.97 6.86 1.59 4.07 1.60 1.46 5.59 6.56
f1 (ppm)

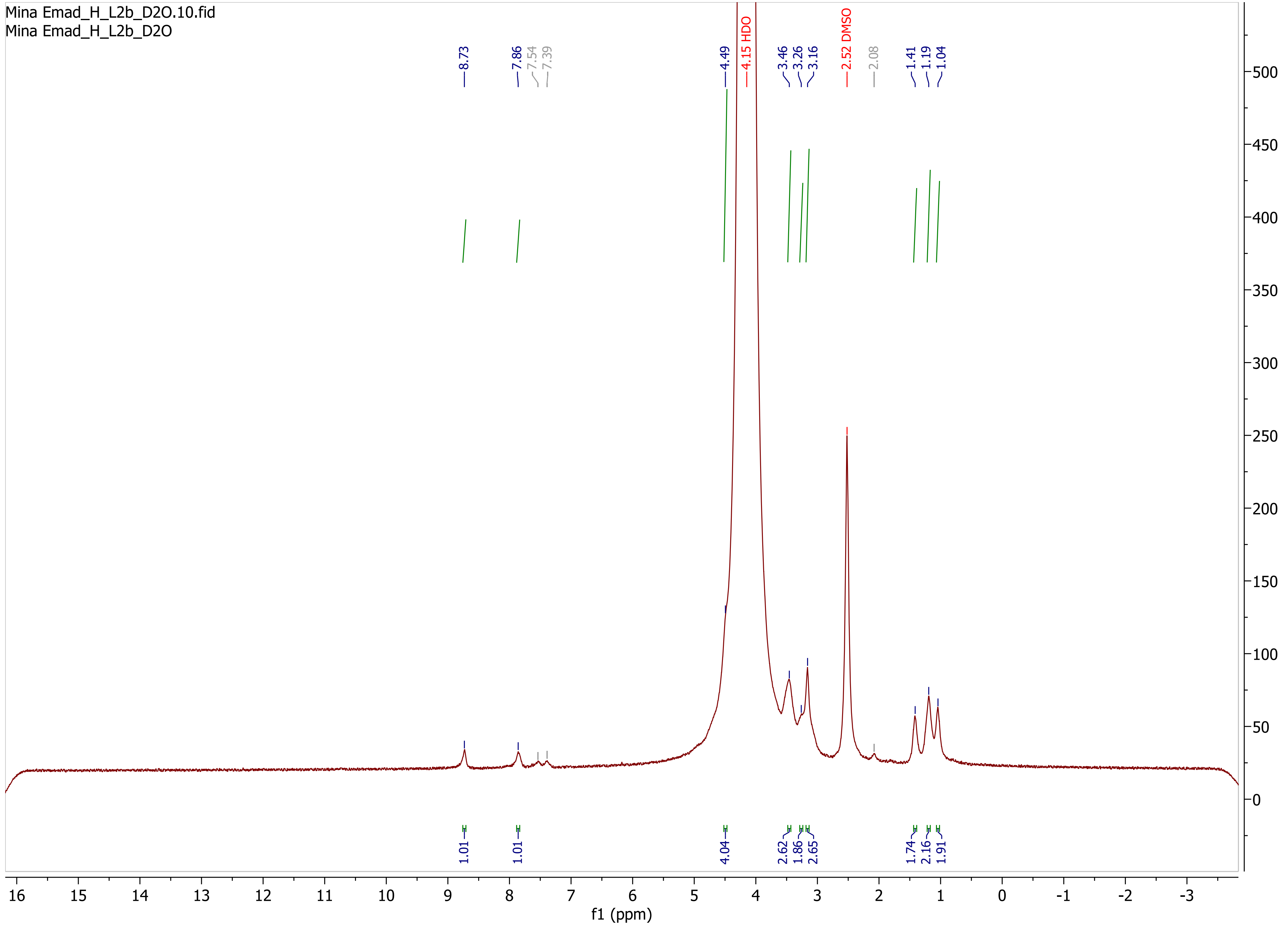
Mina Emad_H_L2b_D2O.10.fid
Mina Emad_H_L2b_D2O
8.73
7.86
7.54
7.39
4.49
4.15 HDO
3.46
3.26
3.16
2.52 DMSO
2.08
1.41
1.19
1.04
1.01
1.01
4.04
2.62
1.86
2.65
1.74
2.16
1.91
f1 (ppm)

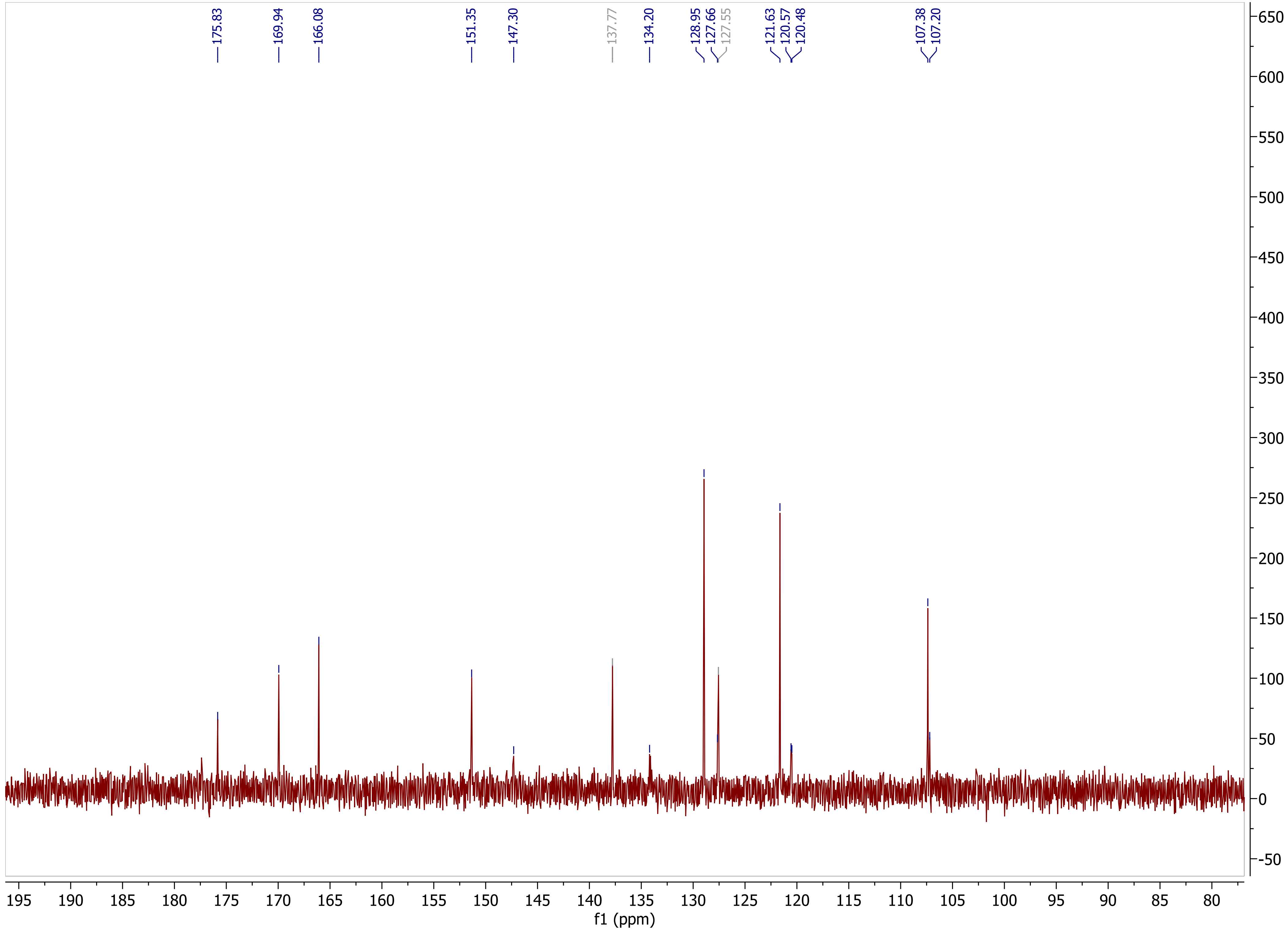
175.83
169.94
166.08
151.35
147.30
137.77
134.20
128.95
127.66
127.55
121.63
120.57
120.48
107.38
107.20
f1 (ppm)

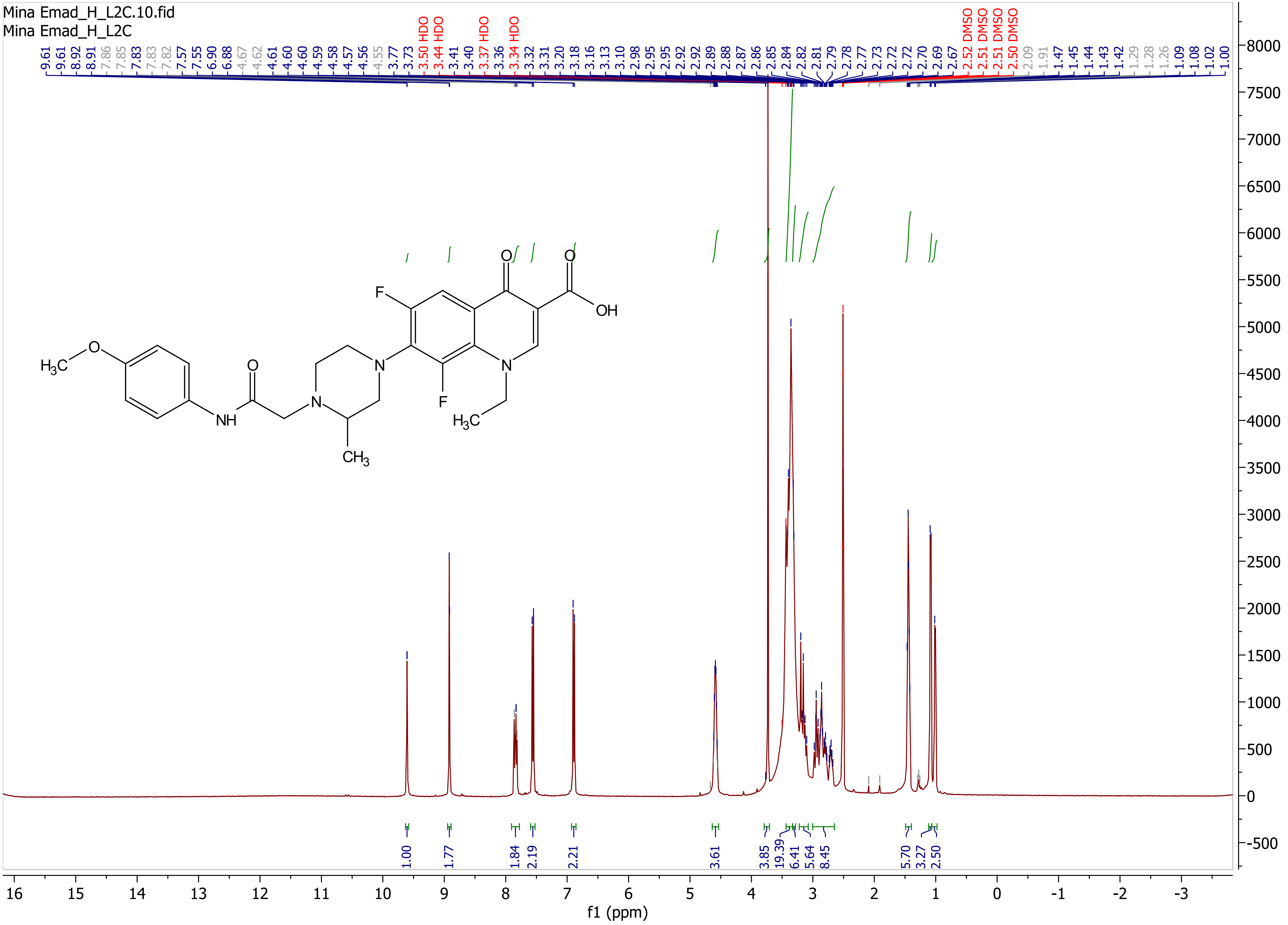
Mina Emad_H_L2C.10.fid
Mina Emad_H_L2C
9.61 9.61 8.92 8.91 7.86 7.85 7.83 7.83 7.82 7.57 7.55 6.90 6.88 4.67 4.62 4.61 4.60 4.60 4.59 4.58 4.57 4.56 4.55 3.77 3.73
3.50 HDO 3.44 HDO 3.41 3.40 3.37 HDO 3.36 3.34 HDO 3.32 3.31 3.20 3.18 3.16 3.13 3.10 2.98 2.95 2.95 2.92 2.92 2.89 2.88 2.87 2.86 2.85 2.84 2.82 2.81 2.79 2.78 2.77 2.73 2.72 2.72 2.70 2.69 2.67
2.52 DMSO 2.51 DMSO 2.51 DMSO 2.50 DMSO 2.09 1.91 1.47 1.45 1.44 1.43 1.42 1.29 1.28 1.26 1.09 1.08 1.02 1.00
F
O
O
OH
N
N
N
H3C
O
NH
F
H3C
CH3
1.00 1.77 1.84 2.19 2.21 3.61 3.85 19.39 6.41 5.64 8.45 5.70 3.27 2.50
16 15 14 13 12 11 10 9 8 7 6 5 4 3 2 1 0 -1 -2 -3
f1 (ppm)
8000 7500 7000 6500 6000 5500 5000 4500 4000 3500 3000 2500 2000 1500 1000 500 0 -500

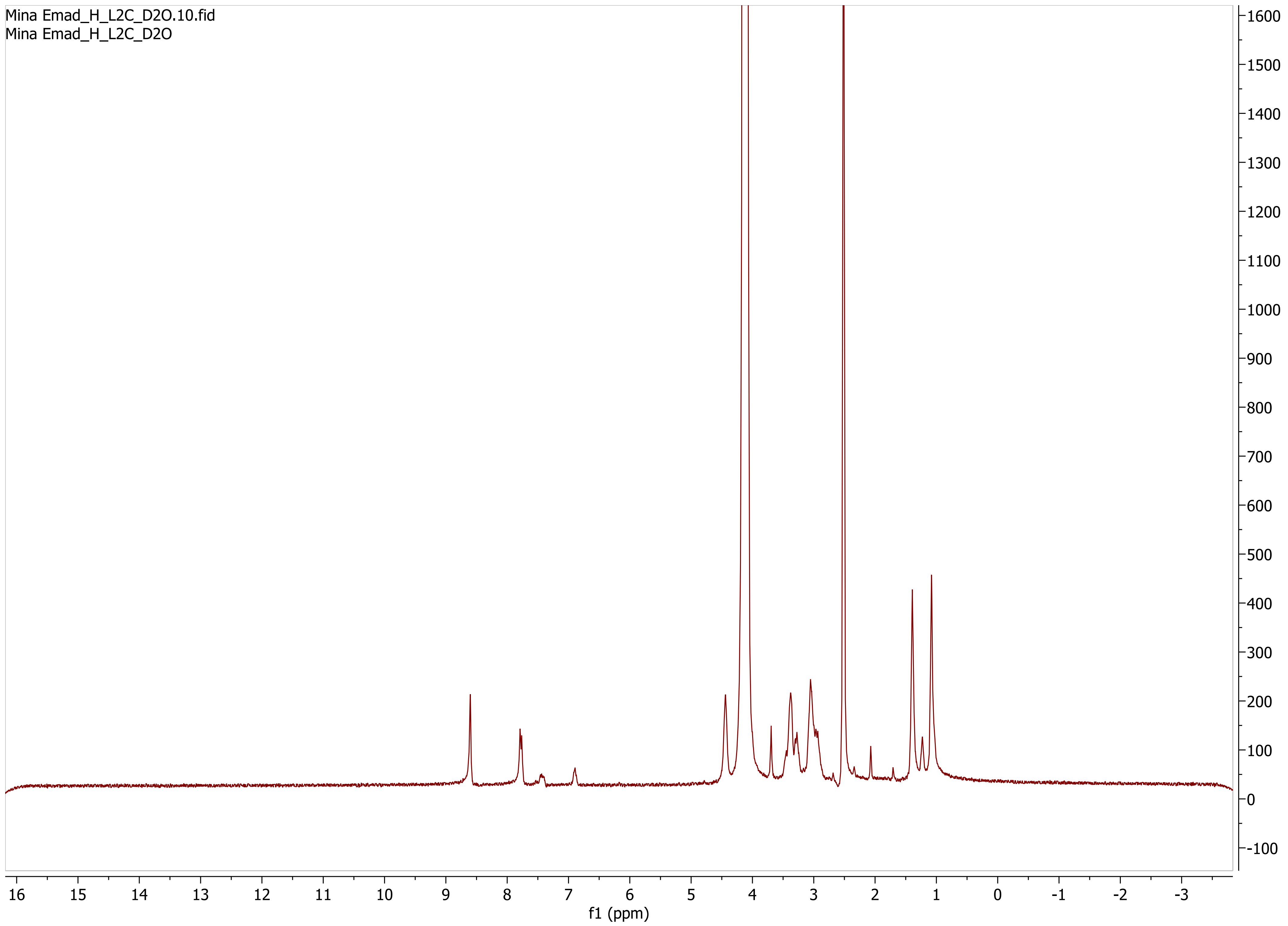
Mina Emad_H_L2C_D2O.10.fid
Mina Emad_H_L2C_D2O
f1 (ppm)

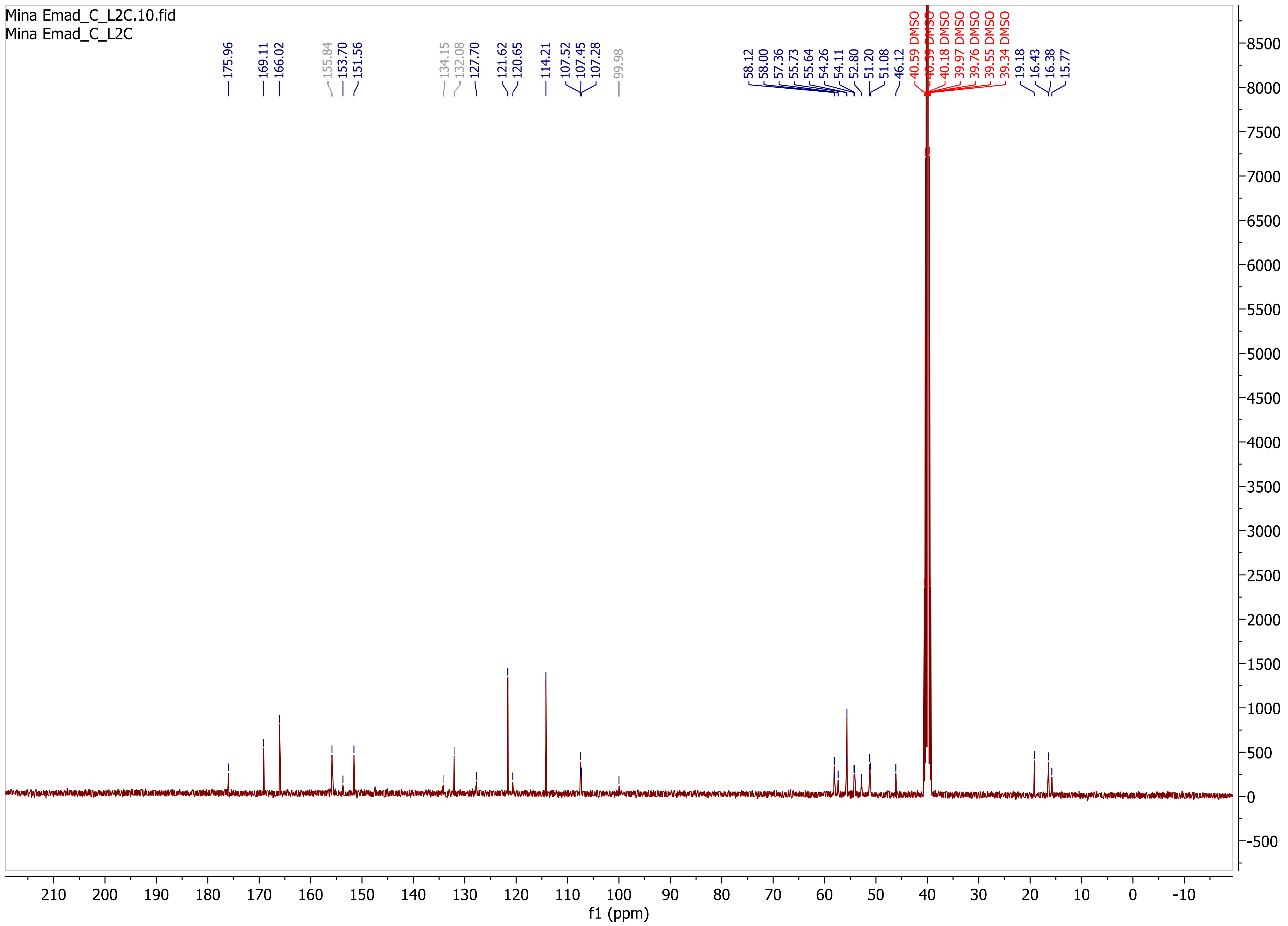

Mina Emad_C_L2C.10.fid
Mina Emad_C_L2C
175.96
169.11
166.02
155.84
153.70
151.56
134.15
132.08
127.70
121.62
120.65
114.21
107.52
107.45
107.28
99.98
58.12
58.00
57.36
55.73
55.64
54.26
54.11
52.80
51.20
51.08
46.12
40.59 DMSO
40.39 DMSO
40.18 DMSO
39.97 DMSO
39.76 DMSO
39.55 DMSO
39.34 DMSO
19.18
16.43
16.38
15.77
f1 (ppm)

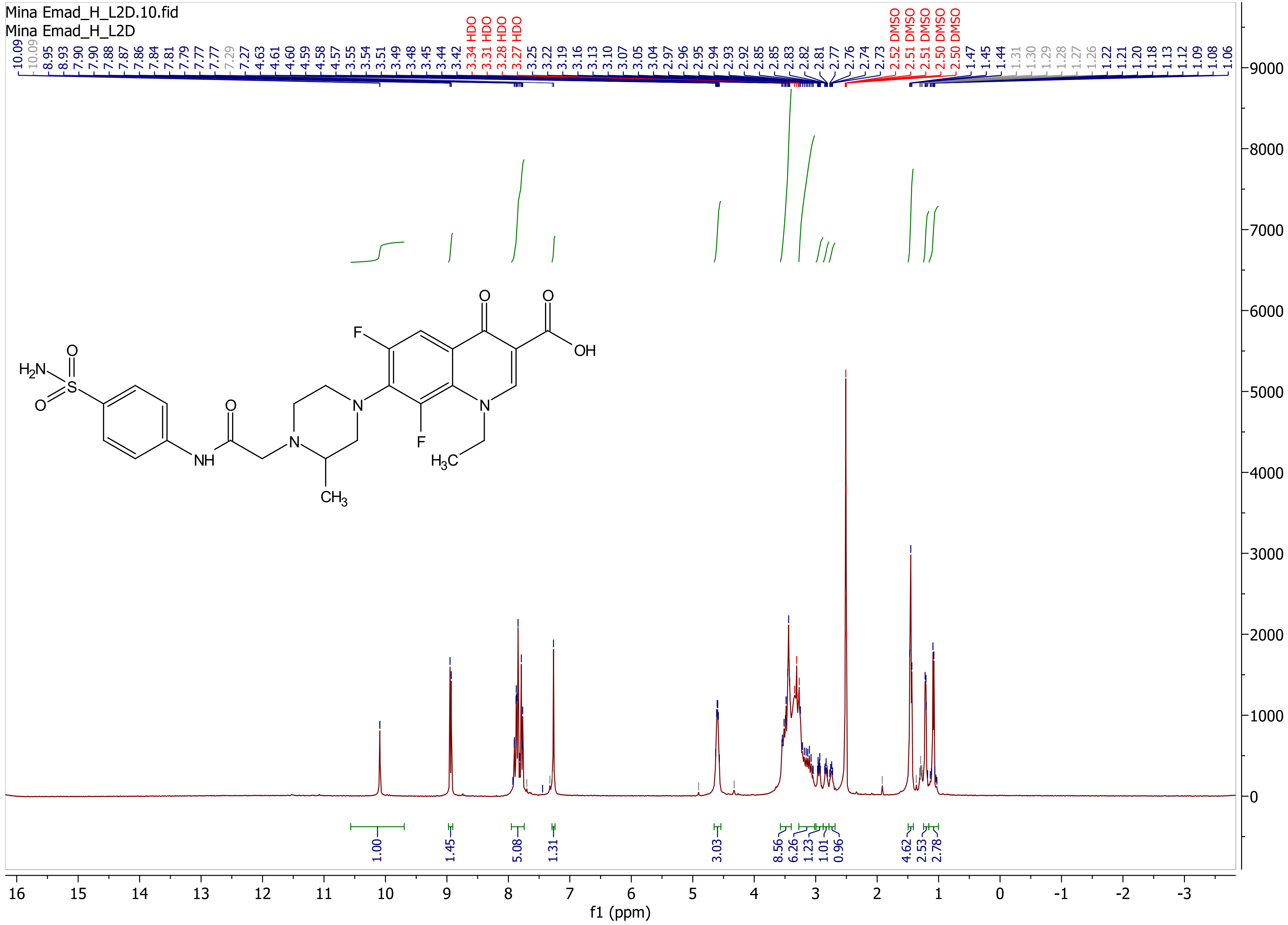

Mina Emad_H_L2D.10.fid
Mina Emad_H_L2D
10.09 10.09 8.95 8.93 7.90 7.90 7.88 7.87 7.86 7.84 7.81 7.79 7.77 7.77 7.29 7.27 4.63 4.61 4.60 4.59 4.58 4.57 3.55 3.54 3.51 3.49 3.48 3.45 3.44 3.42
3.34 HDO 3.31 HDO 3.28 HDO 3.27 HDO
3.25 3.22 3.19 3.16 3.13 3.10 3.07 3.05 3.04 2.97 2.96 2.95 2.94 2.93 2.92 2.85 2.85 2.83 2.82 2.81 2.77 2.76 2.74 2.73
2.52 DMSO 2.51 DMSO 2.51 DMSO 2.50 DMSO 2.50 DMSO
1.47 1.45 1.44 1.31 1.30 1.29 1.28 1.27 1.26 1.22 1.21 1.20 1.18 1.13 1.12 1.09 1.08 1.06
O
H2N
S
O
O
F
O
O
OH
N
N
O
NH
N
F
H3C
CH3
1.00
1.45
5.08
1.31
3.03
8.56
6.26
1.23
1.01
0.96
4.62
2.53
2.78
16 15 14 13 12 11 10 9 8 7 6 5 4 3 2 1 0 -1 -2 -3
f1 (ppm)
9000 8000 7000 6000 5000 4000 3000 2000 1000 0

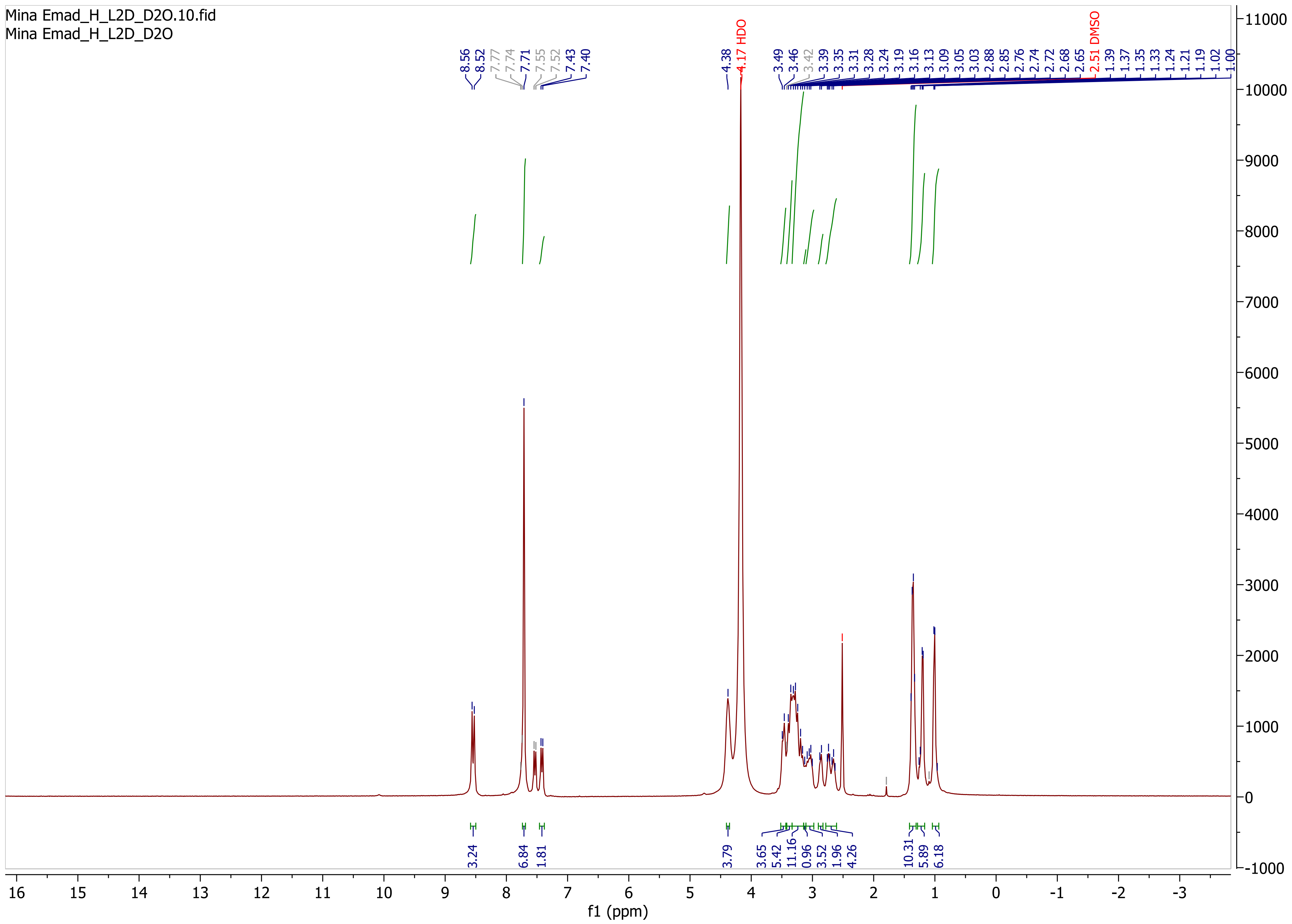

Mina Emad_H_L2D_D2O.10.fid
Mina Emad_H_L2D_D2O
8.56 8.52 7.77 7.74 7.71 7.55 7.52 7.43 7.40
4.38 4.17 HDO
3.49 3.46 3.42 3.39 3.35 3.31 3.28 3.24 3.19 3.16 3.13 3.09 3.05 3.03 2.88 2.85 2.76 2.74 2.72 2.68 2.65 2.51 DMSO 1.39 1.37 1.35 1.33 1.24 1.21 1.19 1.02 1.00
3.24 6.84 1.81 3.79 3.65 5.42 11.16 0.96 3.52 1.96 4.26 10.31 5.89 6.18
f1 (ppm)

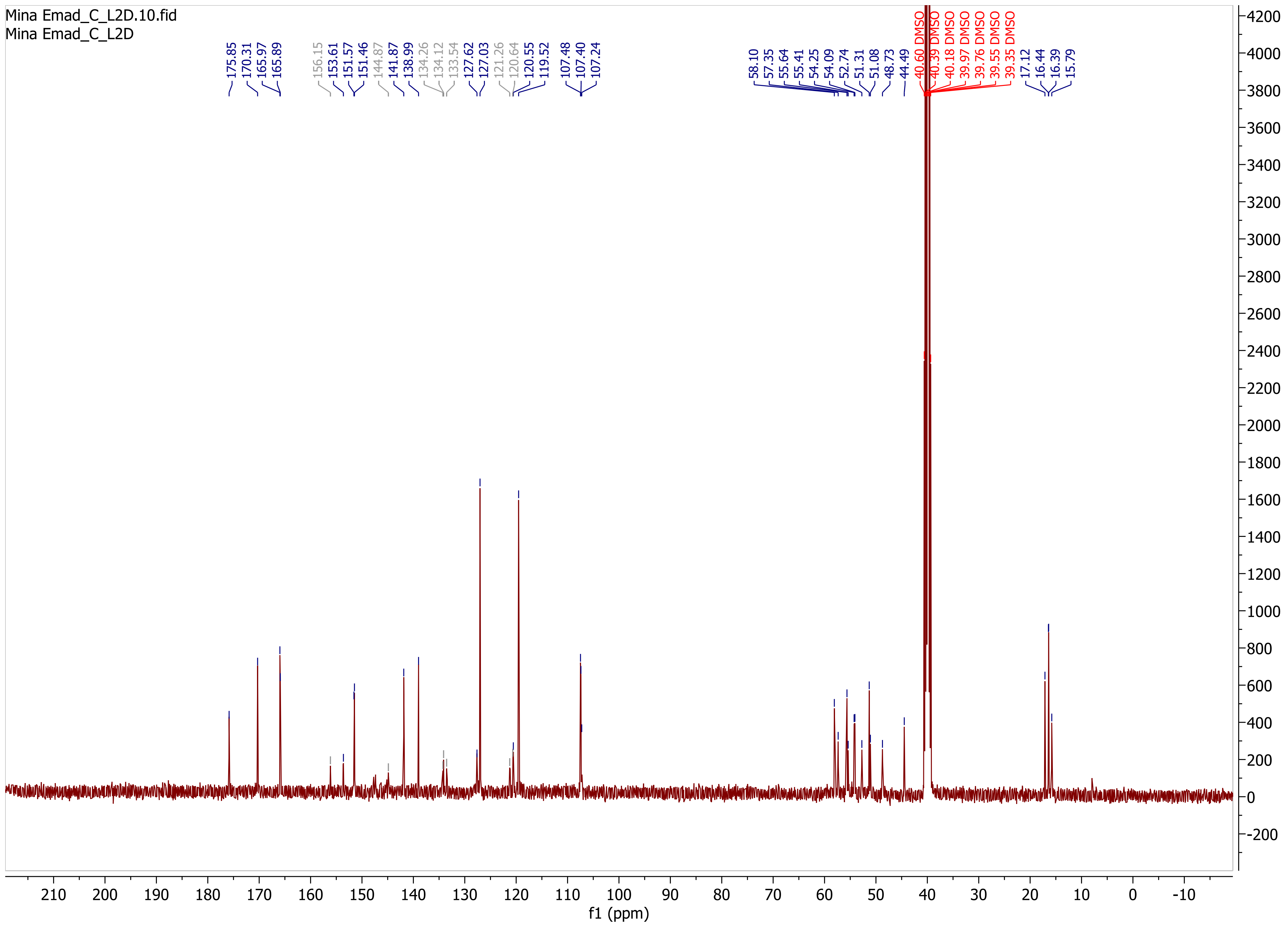
Mina Emad_C_L2D.10.fid
Mina Emad_C_L2D
175.85
170.31
165.97
165.89
156.15
153.61
151.57
151.46
144.87
141.87
138.99
134.26
134.12
133.54
127.62
127.03
121.26
120.64
120.55
119.52
107.48
107.40
107.24
58.10
57.35
55.64
55.41
54.25
54.09
52.74
51.31
51.08
48.73
44.49
40.60 DMSO
40.39 DMSO
40.18 DMSO
39.97 DMSO
39.76 DMSO
39.55 DMSO
39.35 DMSO
17.12
16.44
16.39
15.79
f1 (ppm)

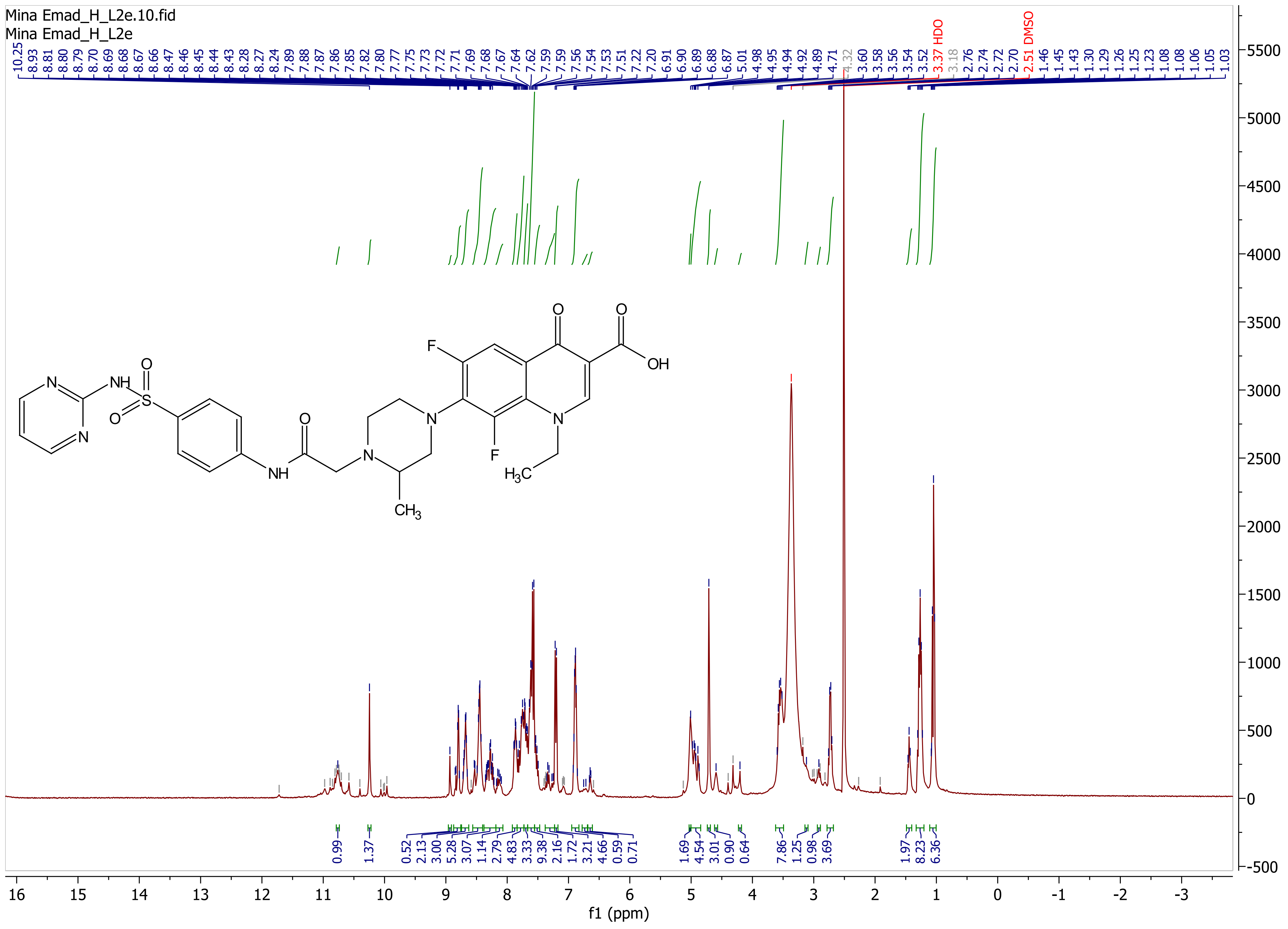

Mina Emad_H_L2e.10.fid
Mina Emad_H_L2e
10.25 8.93 8.81 8.80 8.79 8.70 8.69 8.68 8.67 8.66 8.47 8.46 8.45 8.44 8.43 8.28 8.27 8.24 7.89 7.88 7.87 7.86 7.85 7.82 7.80 7.77 7.75 7.73 7.72 7.71 7.69 7.68 7.67 7.64 7.62 7.59 7.59 7.56 7.54 7.53 7.51 7.22 7.20 6.91 6.90 6.89 6.88 6.87 5.01 4.98 4.95 4.94 4.92 4.89 4.71 4.32 3.60 3.58 3.56 3.54 3.52 3.37 HDO 3.18 2.76 2.74 2.72 2.70 2.51 DMSO 1.46 1.45 1.43 1.30 1.29 1.26 1.25 1.23 1.08 1.08 1.06 1.05 1.03
0.99 1.37 0.52 2.13 3.00 5.28 3.07 1.14 2.79 4.83 3.33 9.38 2.16 1.72 3.21 4.66 0.59 0.71 1.69 4.54 3.01 0.90 0.64 7.86 1.25 0.98 3.69 1.97 8.23 6.36
f1 (ppm)

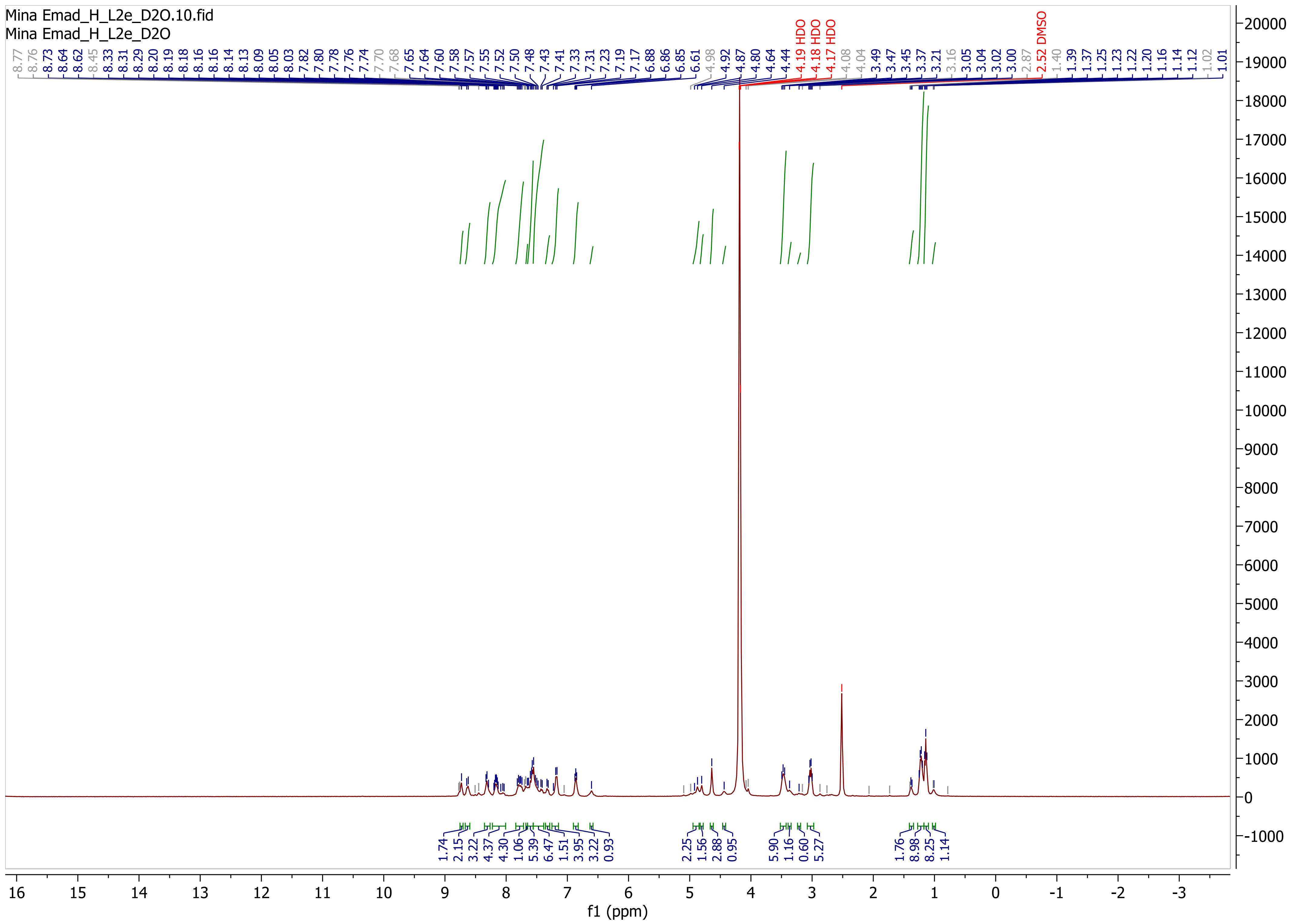

Mina Emad_H_L2e_D2O.10.fid
Mina Emad_H_L2e_D2O
4.19 HDO
4.18 HDO
4.17 HDO
2.52 DMSO
f1 (ppm)

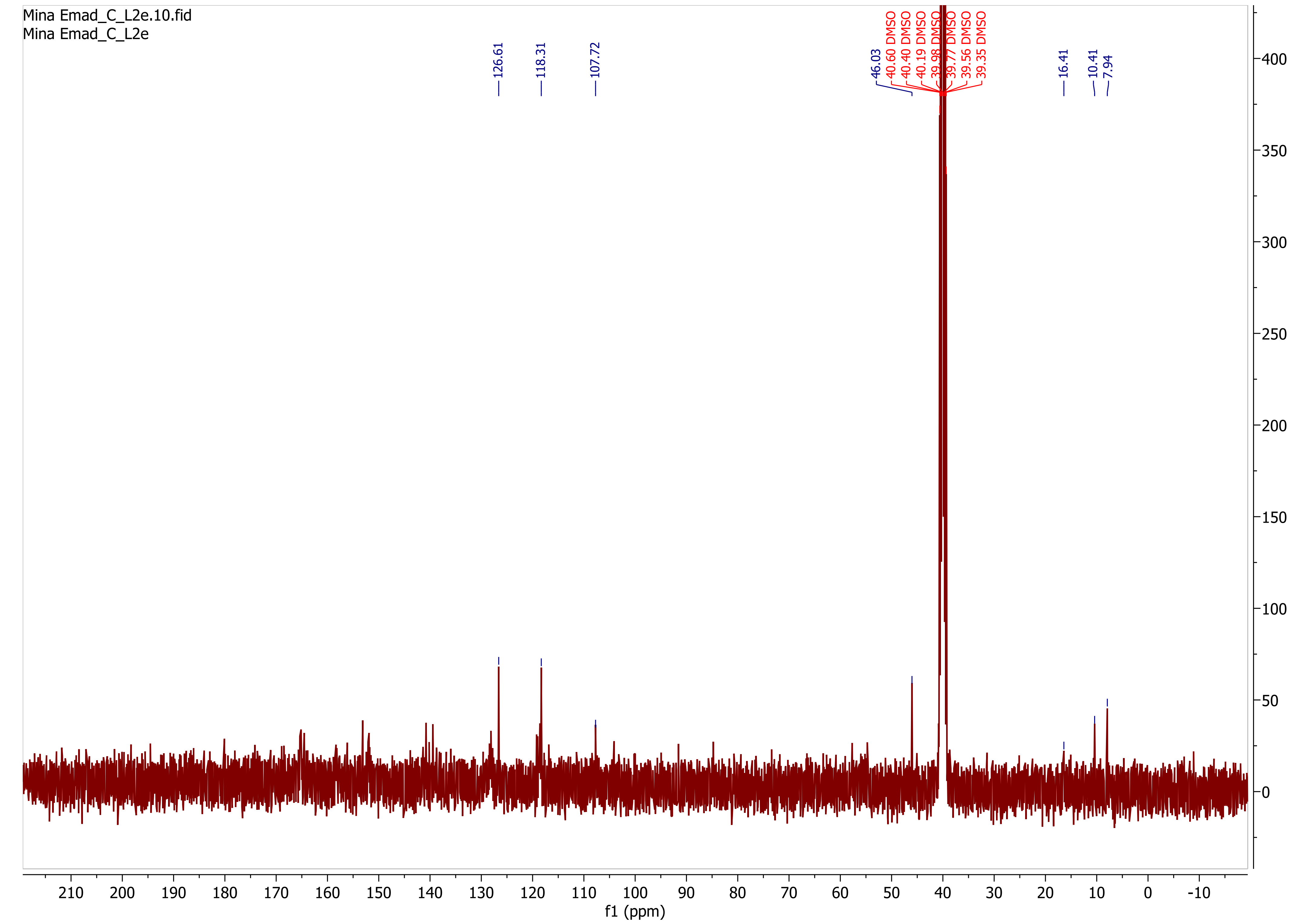

Mina Emad_C_L2e.10.fid
Mina Emad_C_L2e
126.61
118.31
107.72
46.03
40.60 DMSO
40.40 DMSO
40.19 DMSO
39.98 DMSO
39.77 DMSO
39.56 DMSO
39.35 DMSO
16.41
10.41
7.94
f1 (ppm)

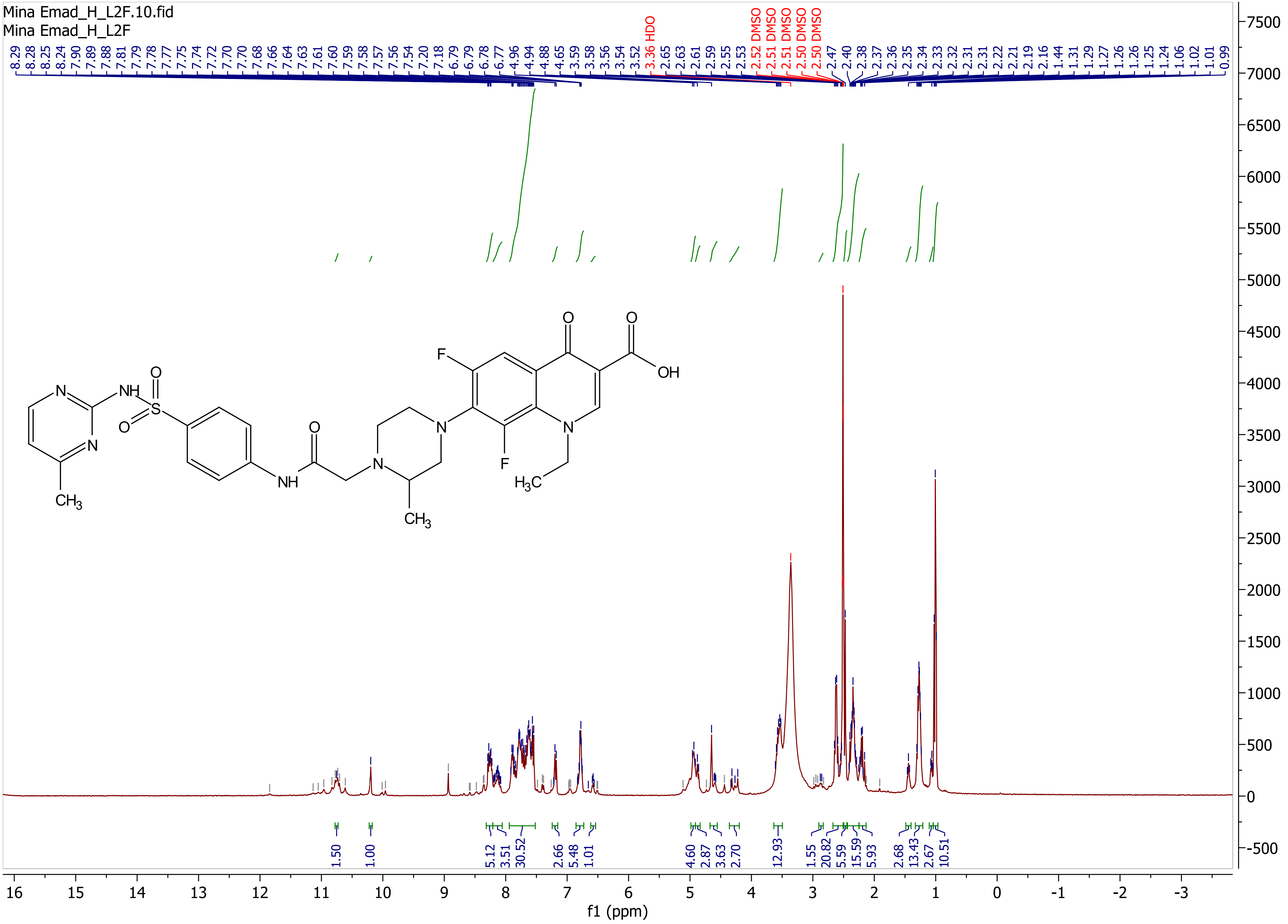

Mina Emad_H_L2F.10.fid
Mina Emad_H_L2F
8.29 8.28 8.25 8.24 7.90 7.89 7.88 7.81 7.79 7.78 7.77 7.75 7.74 7.72 7.70 7.70 7.68 7.66 7.64 7.63 7.61 7.60 7.59 7.58 7.57 7.56 7.54 7.20 7.18 6.79 6.79 6.78 6.77 4.96 4.94 4.88 4.65 3.59 3.58 3.56 3.54 3.52
3.36 HDO
2.65 2.63 2.61 2.59 2.55 2.53
2.52 DMSO
2.51 DMSO
2.51 DMSO
2.50 DMSO
2.50 DMSO
2.47 2.40 2.38 2.37 2.36 2.35 2.34 2.33 2.32 2.31 2.31 2.22 2.21 2.19 2.16 1.44 1.31 1.29 1.27 1.26 1.26 1.25 1.24 1.06 1.02 1.01 0.99
1.50 1.00 5.12 3.51 30.52 2.66 5.48 1.01 4.60 2.87 3.63 2.70 12.93 1.55 20.82 5.59 15.59 5.93 2.68 13.43 2.67 10.51
f1 (ppm)

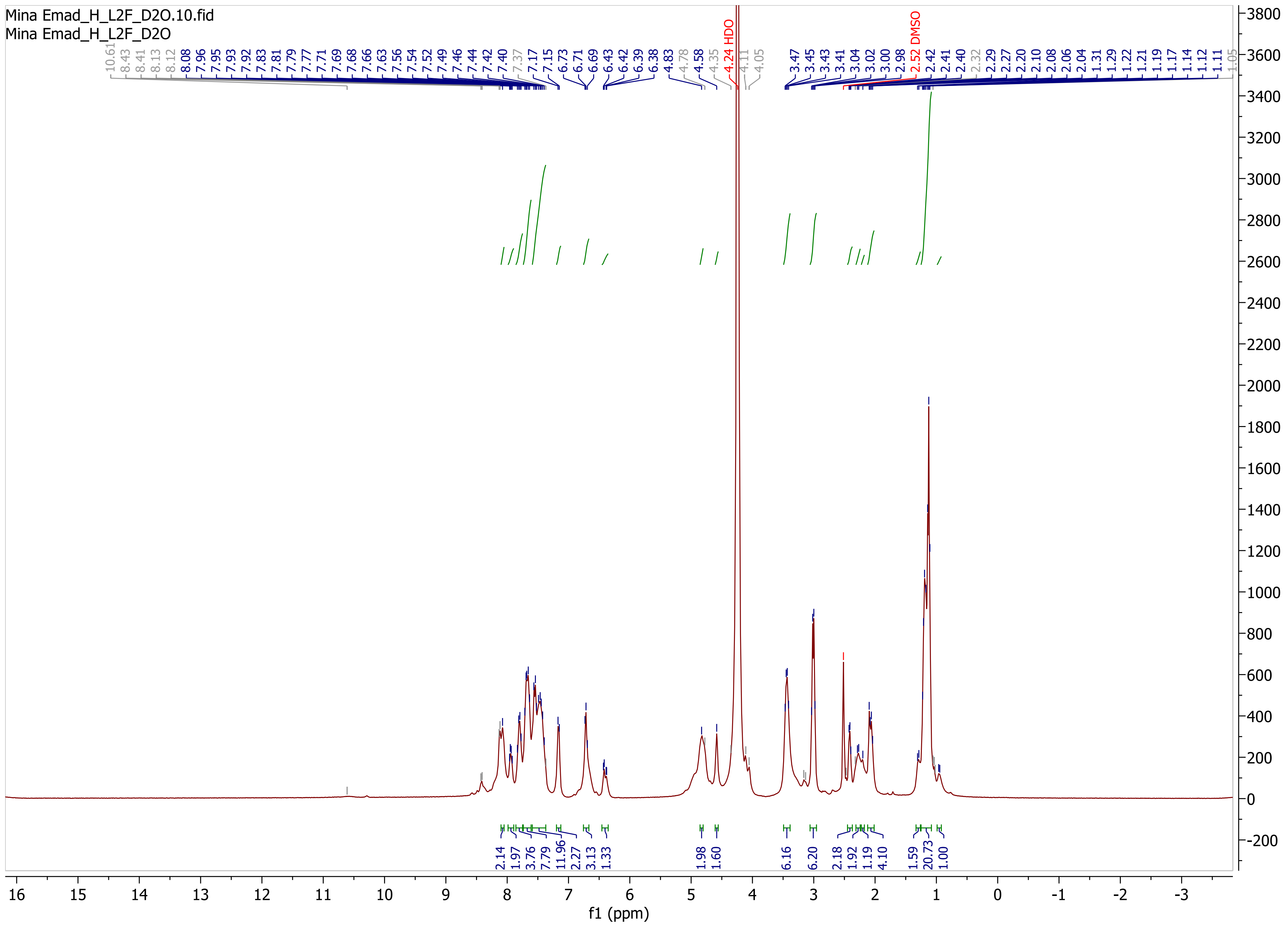

Mina Emad_H_L2F_D2O.10.fid
Mina Emad_H_L2F_D2O
10.61 8.43 8.41 8.13 8.12 8.08 7.96 7.95 7.93 7.92 7.83 7.81 7.79 7.77 7.71 7.69 7.68 7.66 7.63 7.56 7.54 7.52 7.49 7.46 7.44 7.42 7.40 7.37 7.17 7.15 6.73 6.71 6.69 6.43 6.42 6.39 6.38 4.83 4.78 4.58 4.35 4.24 HDO 4.11 4.05 3.47 3.45 3.43 3.41 3.04 3.02 3.00 2.98 2.52 DMSO 2.42 2.41 2.40 2.32 2.29 2.27 2.20 2.10 2.08 2.06 2.04 1.31 1.29 1.22 1.21 1.19 1.17 1.14 1.12 1.11 1.05
2.14 1.97 3.76 7.79 11.96 2.27 3.13 1.33 1.98 1.60 6.16 6.20 2.18 1.92 1.19 4.10 1.59 20.73 1.00
f1 (ppm)

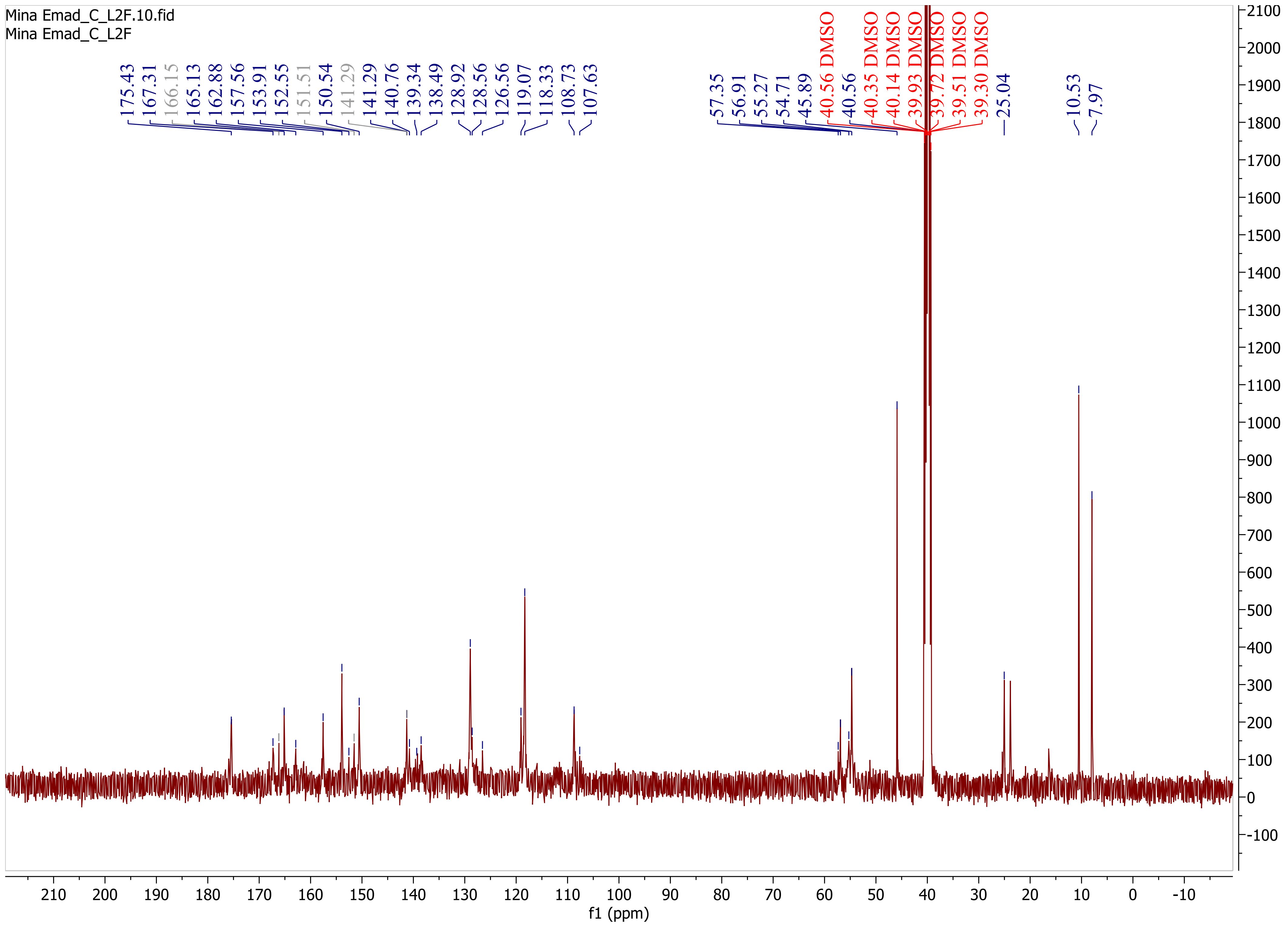
Mina Emad_C_L2F.10.fid
Mina Emad_C_L2F
175.43
167.31
166.15
165.13
162.88
157.56
153.91
152.55
151.51
150.54
141.29
141.29
140.76
139.34
138.49
128.92
128.56
126.56
119.07
118.33
108.73
107.63
57.35
56.91
55.27
54.71
45.89
40.56 DMSO
40.56
40.35 DMSO
40.14 DMSO
39.93 DMSO
39.72 DMSO
39.51 DMSO
39.30 DMSO
25.04
10.53
7.97
f1 (ppm)

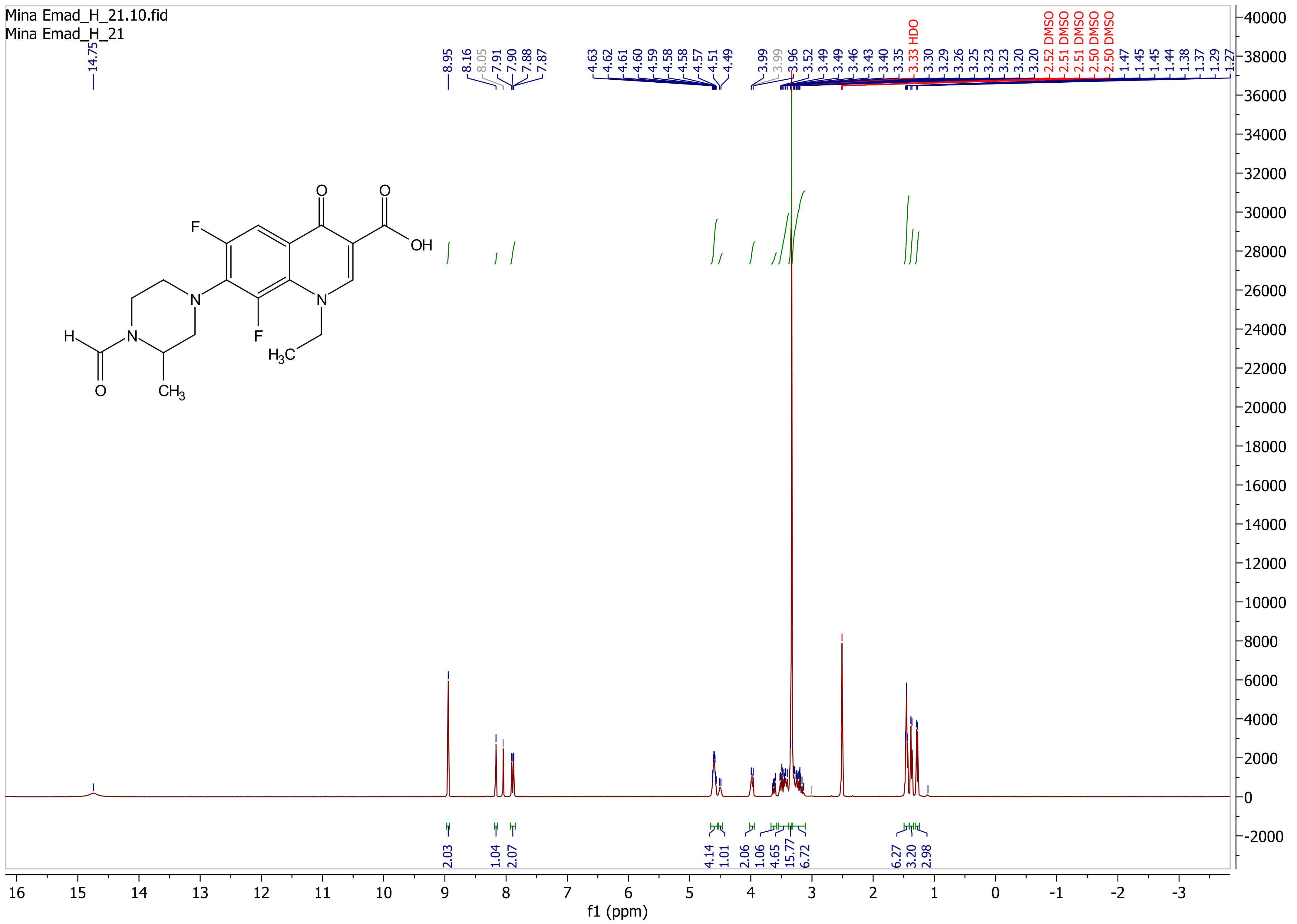

Mina Emad_H_21.10.fid
Mina Emad_H_21
14.75
8.95
8.16
8.05
7.91
7.90
7.88
7.87
4.63
4.62
4.61
4.60
4.59
4.58
4.58
4.57
4.51
4.49
3.99
3.99
3.96
3.52
3.49
3.49
3.46
3.43
3.40
3.35
3.33 HDO
3.30
3.29
3.26
3.25
3.23
3.23
3.20
3.20
2.52 DMSO
2.51 DMSO
2.51 DMSO
2.50 DMSO
2.50 DMSO
1.47
1.45
1.45
1.44
1.38
1.37
1.29
1.27
F
F
O
O
OH
N
N
H
N
O
CH3
H3C
2.03
1.04
2.07
4.14
1.01
2.06
1.06
4.65
15.77
6.72
6.27
3.20
2.98
f1 (ppm)

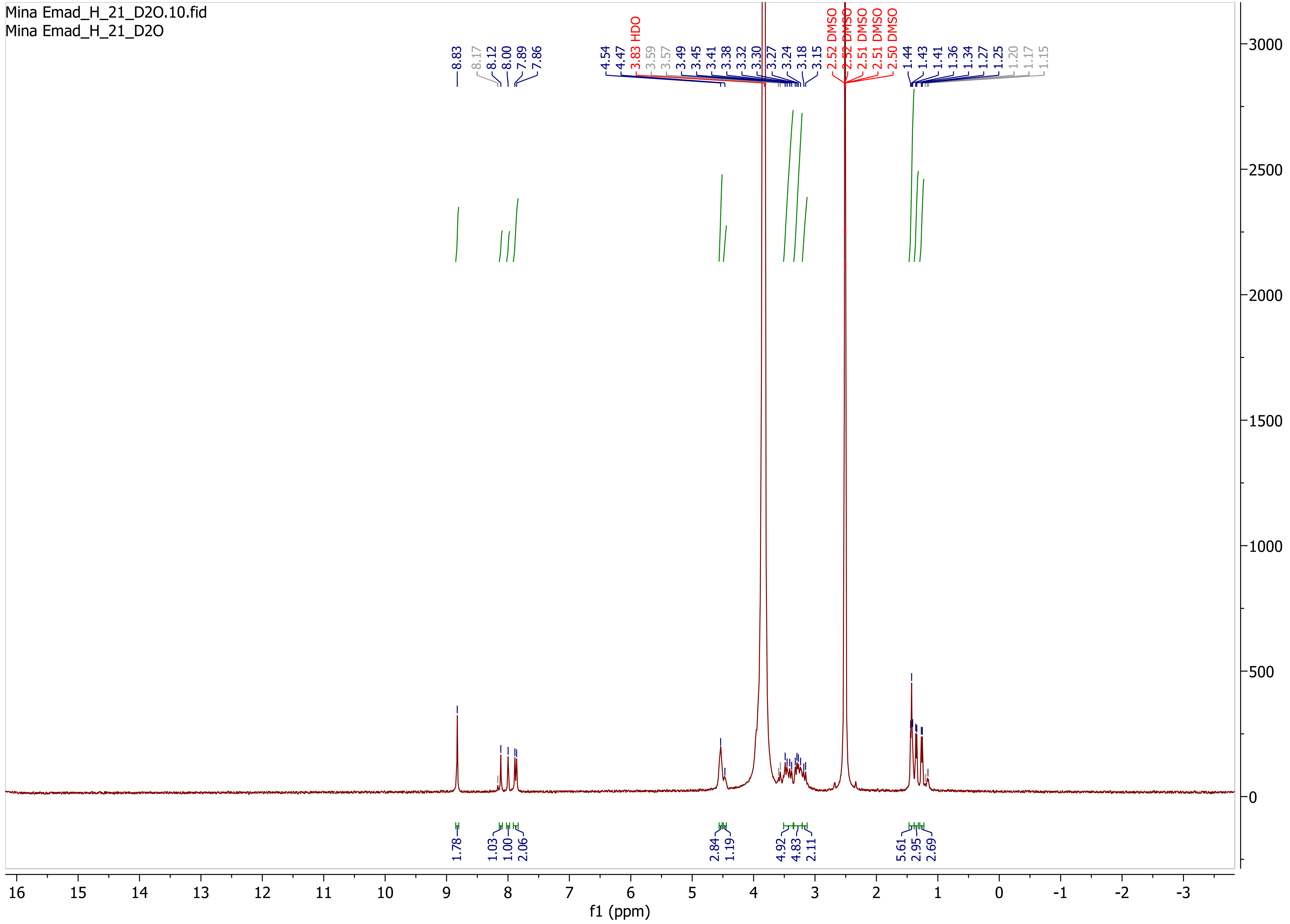

Mina Emad_H_21_D2O.10.fid
Mina Emad_H_21_D2O
8.83
8.17
8.12
8.00
7.89
7.86
4.54
4.47
3.83 HDO
3.59
3.57
3.49
3.45
3.41
3.38
3.32
3.30
3.27
3.24
3.18
3.15
2.52 DMSO
2.52 DMSO
2.51 DMSO
2.51 DMSO
2.50 DMSO
1.44
1.43
1.41
1.36
1.34
1.27
1.25
1.20
1.17
1.15
1.78
1.03
1.00
2.06
2.84
1.19
4.92
4.83
2.11
5.61
2.95
2.69
f1 (ppm)

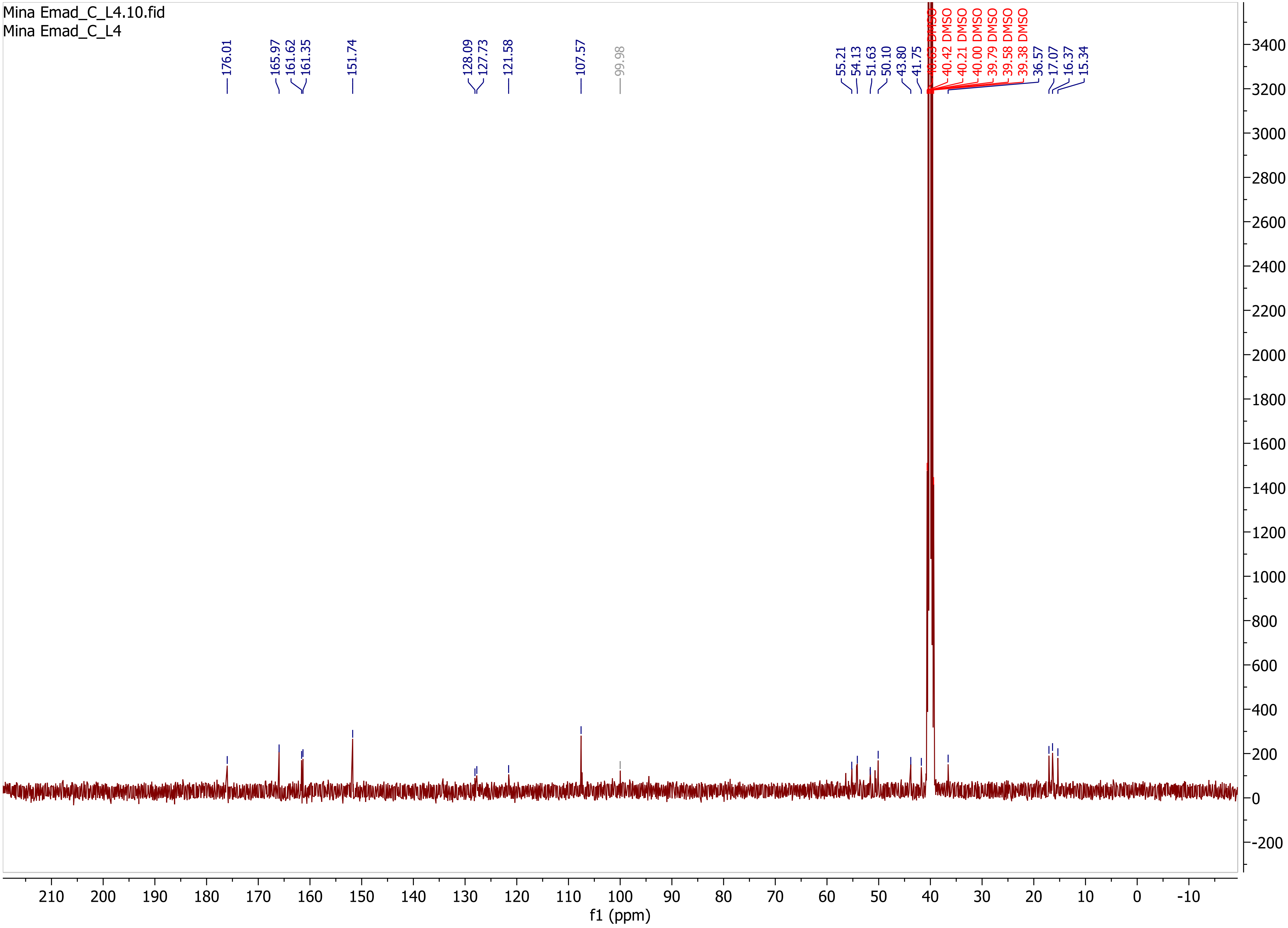
Mina Emad_C_L4.10.fid
Mina Emad_C_L4
176.01
165.97
161.62
161.35
151.74
128.09
127.73
121.58
107.57
99.98
55.21
54.13
51.63
50.10
43.80
41.75
40.42 DMSO
40.21 DMSO
40.00 DMSO
39.79 DMSO
39.58 DMSO
39.38 DMSO
36.57
17.07
16.37
15.34
f1 (ppm)

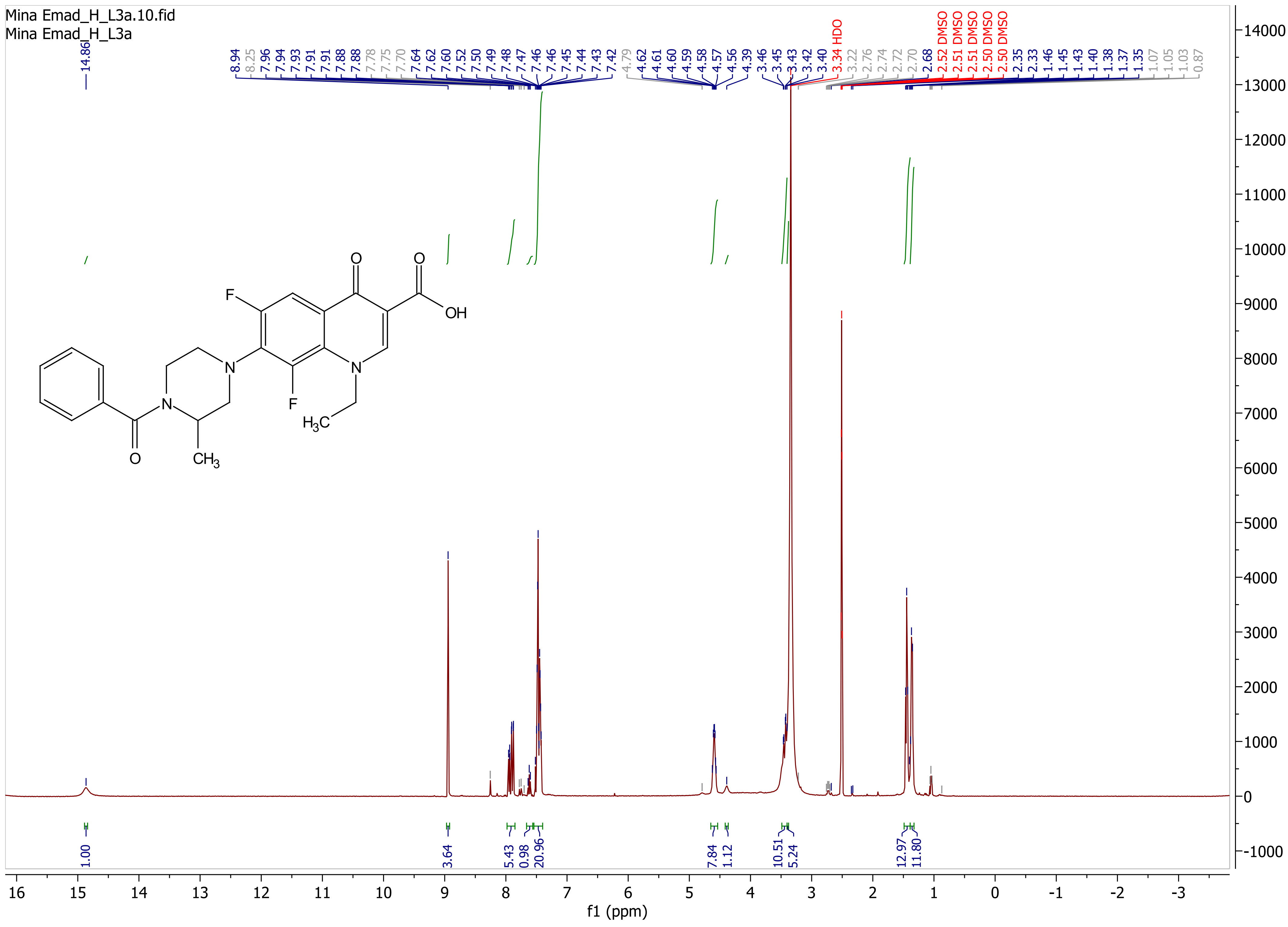
Mina Emad_H_L3a.10.fid
Mina Emad_H_L3a
14.86
8.94 8.25 7.96 7.94 7.93 7.91 7.91 7.88 7.88 7.78 7.75 7.70 7.64 7.62 7.60 7.52 7.50 7.49 7.48 7.47 7.46 7.46 7.45 7.44 7.43 7.42
4.79 4.62 4.61 4.60 4.59 4.58 4.57 4.56 4.39
3.46 3.45 3.43 3.42 3.40
3.34 HDO
3.22 2.76 2.74 2.72 2.70 2.68
2.52 DMSO
2.51 DMSO
2.51 DMSO
2.50 DMSO
2.50 DMSO
2.35 2.33 1.46 1.45 1.43 1.40 1.38 1.37 1.35 1.07 1.05 1.03 0.87
1.00 3.64 5.43 0.98 20.96 7.84 1.12 10.51 5.24 12.97 11.80
F
O
O
OH
N
N
N
F
H3C
O
CH3
f1 (ppm)

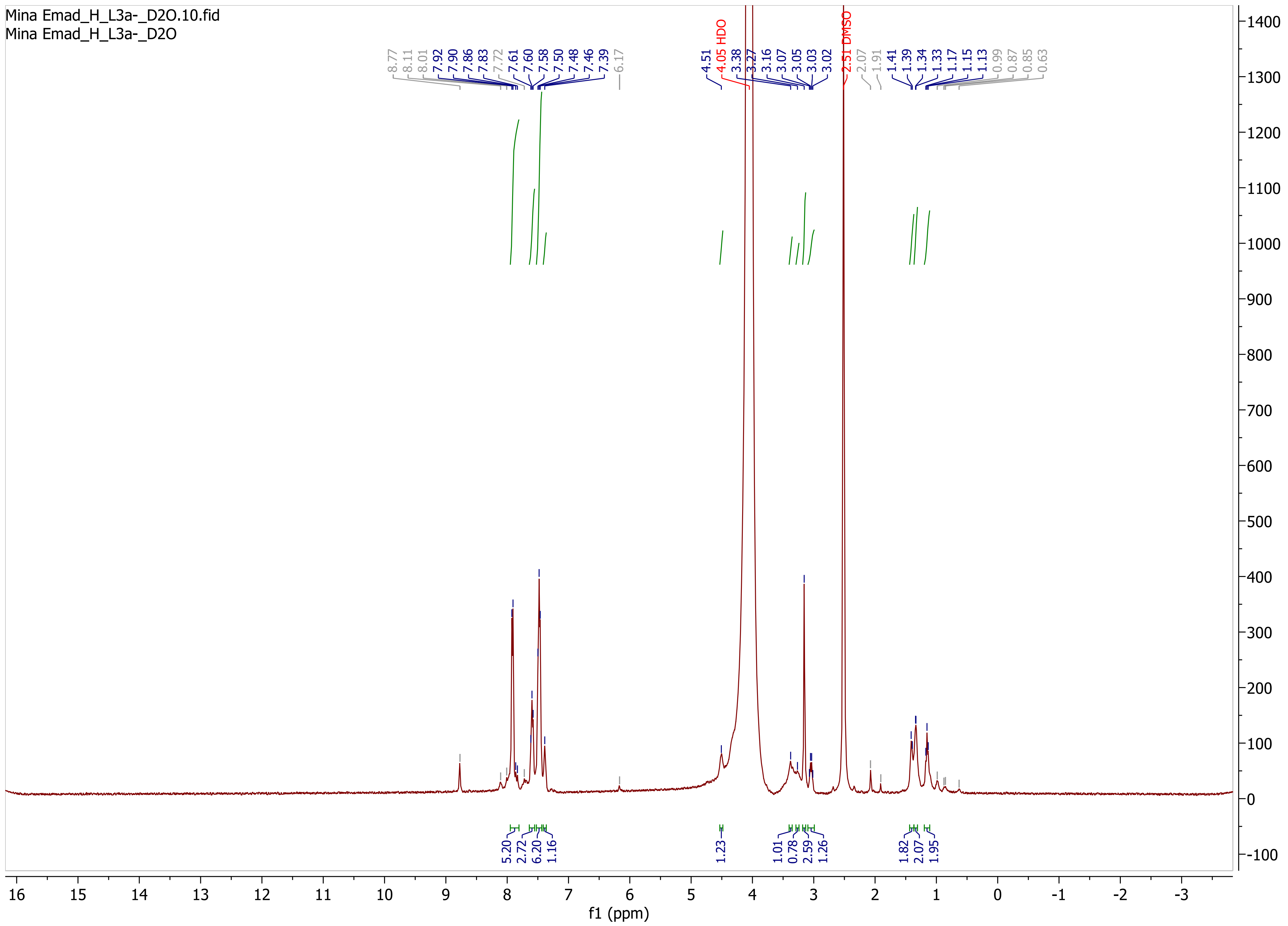

Mina Emad_H_L3a-_D2O.10.fid
Mina Emad_H_L3a-_D2O
8.77 8.11 8.01 7.92 7.90 7.86 7.83 7.72 7.61 7.60 7.58 7.50 7.48 7.46 7.39 6.17
4.51 4.05 HDO 3.38 3.27 3.16 3.07 3.05 3.03 3.02 2.51 DMSO 2.07 1.91 1.41 1.39 1.34 1.33 1.17 1.15 1.13 0.99 0.87 0.85 0.63
5.20 2.72 6.20 1.16 1.23 1.01 0.78 2.59 1.26 1.82 2.07 1.95
f1 (ppm)

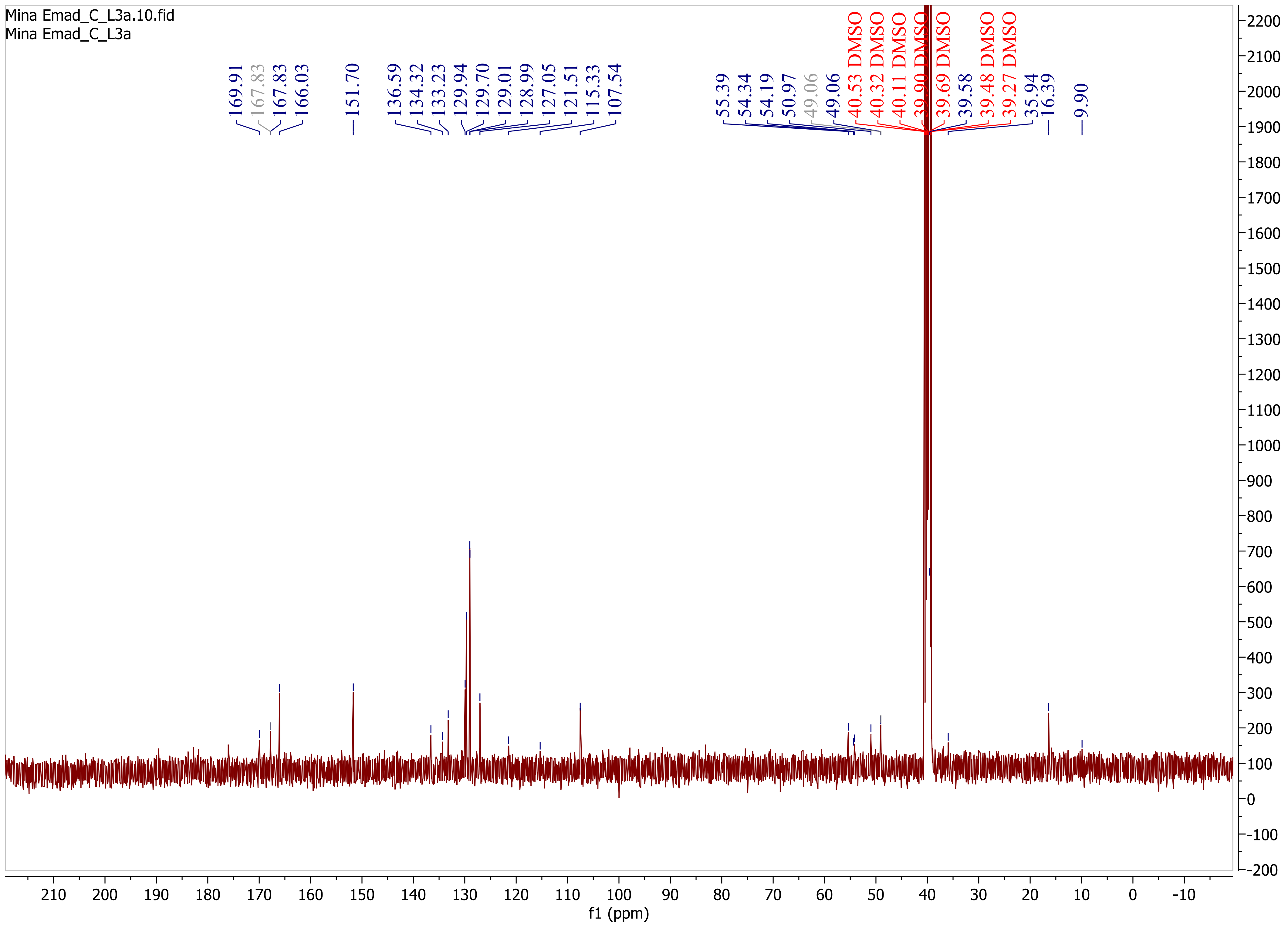
Mina Emad_C_L3a.10.fid
Mina Emad_C_L3a
169.91
167.83
167.83
166.03
151.70
136.59
134.32
133.23
129.94
129.70
129.01
128.99
127.05
121.51
115.33
107.54
55.39
54.34
54.19
50.97
49.06
49.06
40.53 DMSO
40.32 DMSO
40.11 DMSO
39.90 DMSO
39.69 DMSO
39.58
39.48 DMSO
39.27 DMSO
35.94
16.39
9.90
f1 (ppm)

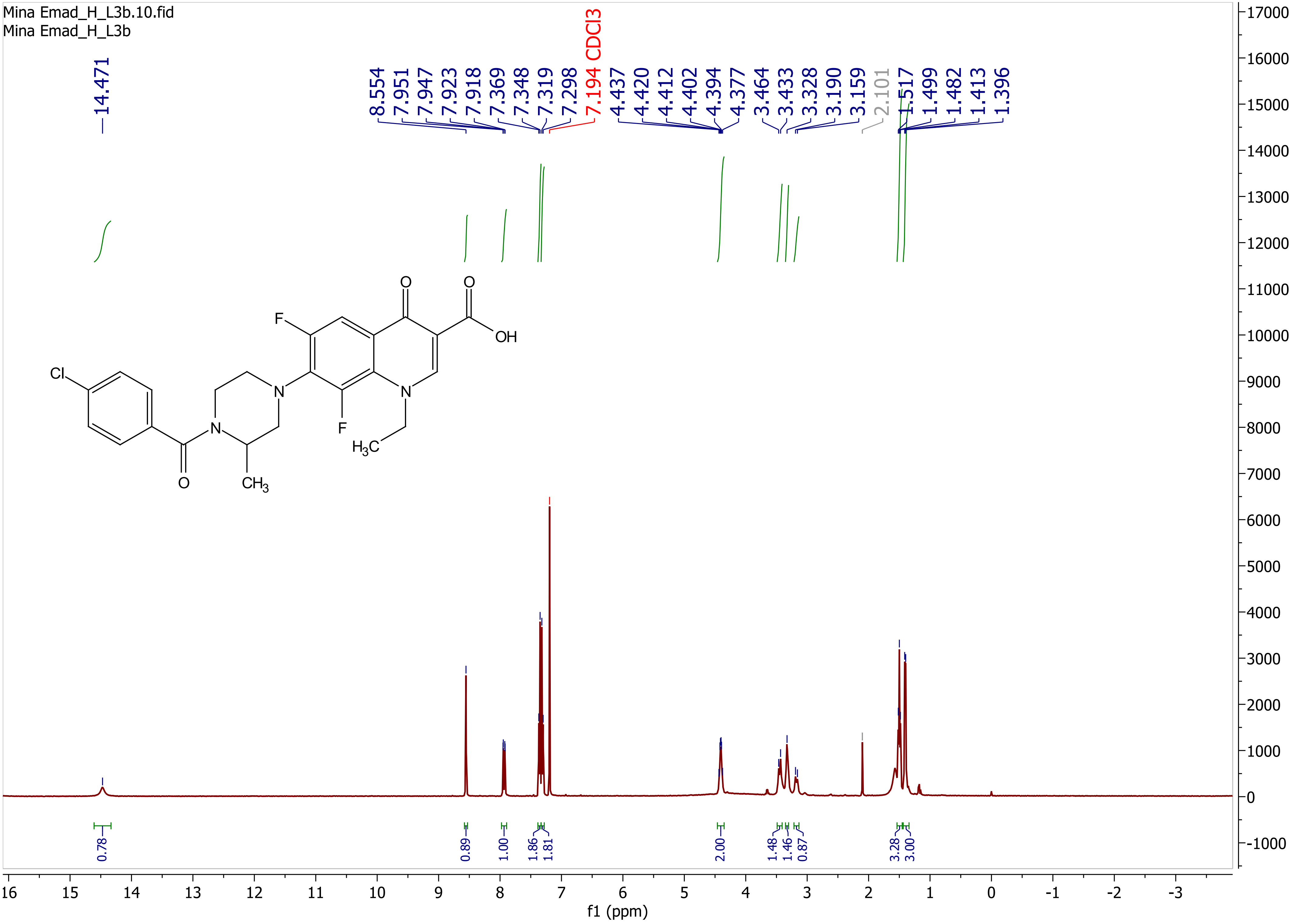
Mina Emad_H_L3b.10.fid
Mina Emad_H_L3b
14.471
8.554
7.951
7.947
7.923
7.918
7.369
7.348
7.319
7.298
7.194 CDCl3
4.437
4.420
4.412
4.402
4.394
4.377
3.464
3.433
3.328
3.190
3.159
2.101
1.517
1.499
1.482
1.413
1.396
Cl
F
O
O
OH
N
N
N
F
H3C
O
CH3
0.78
0.89
1.00
1.86
1.81
2.00
1.48
1.46
0.87
3.28
3.00
f1 (ppm)

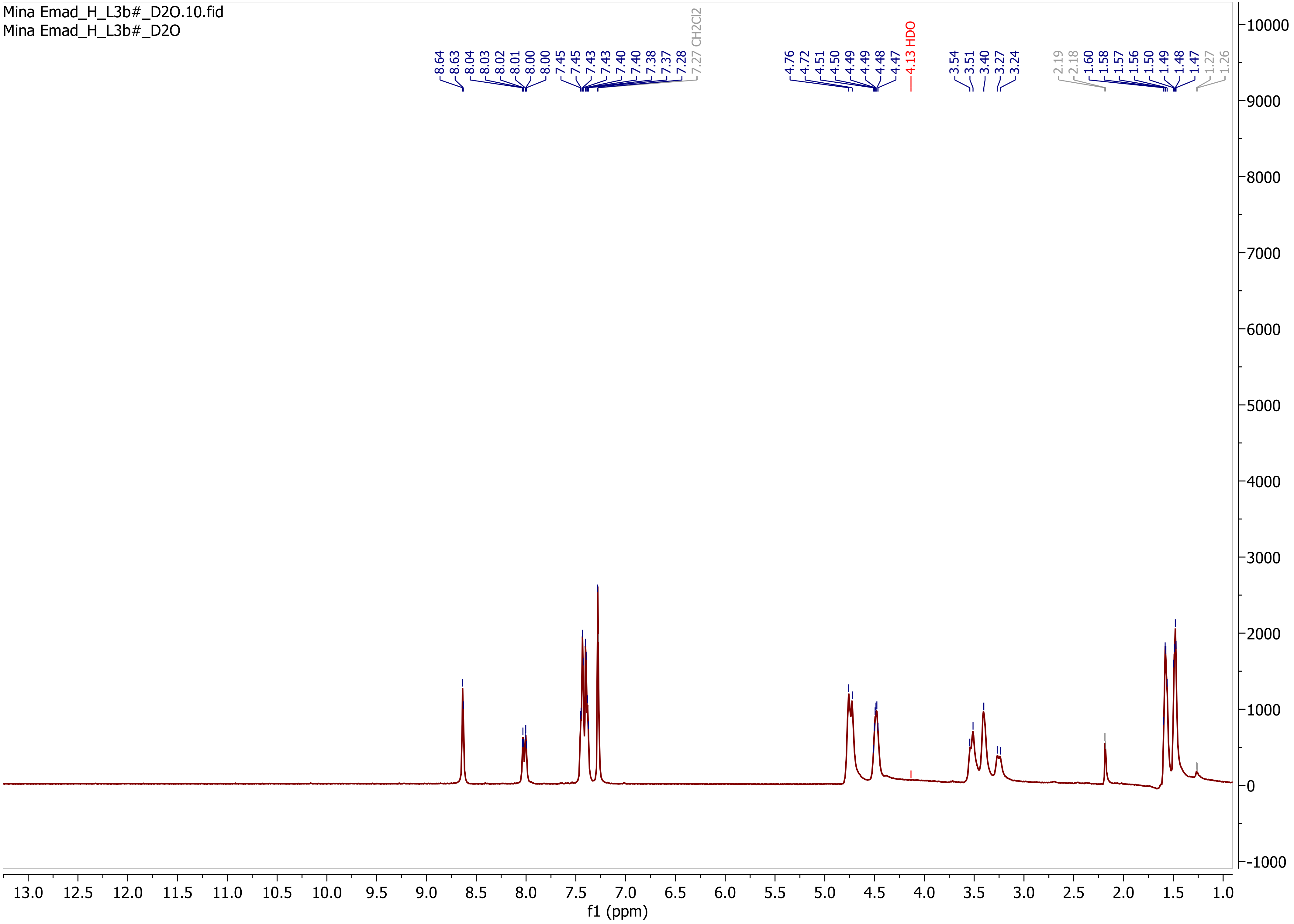
Mina Emad_H_L3b#_D2O.10.fid
Mina Emad_H_L3b#_D2O
8.64
8.63
8.04
8.03
8.02
8.01
8.00
8.00
7.45
7.45
7.43
7.43
7.40
7.40
7.38
7.37
7.28
7.27 CH2Cl2
4.76
4.72
4.51
4.50
4.49
4.49
4.48
4.47
4.13 HDO
3.54
3.51
3.40
3.27
3.24
2.19
2.18
1.60
1.58
1.57
1.56
1.50
1.49
1.48
1.47
1.27
1.26
f1 (ppm)

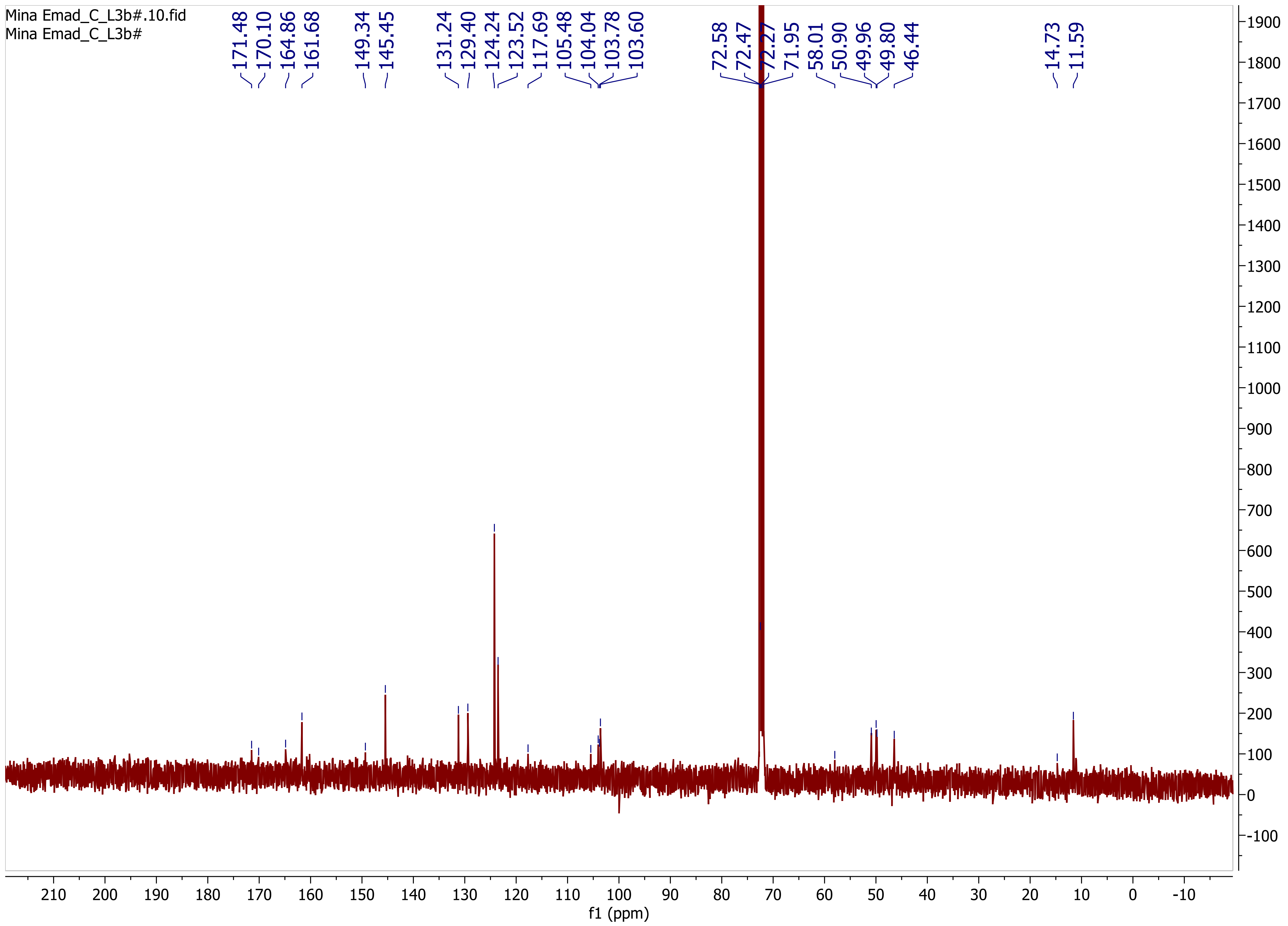
Mina Emad_C_L3b#.10.fid
Mina Emad_C_L3b#
171.48
170.10
164.86
161.68
149.34
145.45
131.24
129.40
124.24
123.52
117.69
105.48
104.04
103.78
103.60
72.58
72.47
72.27
71.95
58.01
50.90
49.96
49.80
46.44
14.73
11.59
f1 (ppm)

Mina Emad_H_L3C#.10.fid
Mina Emad_H_L3C#

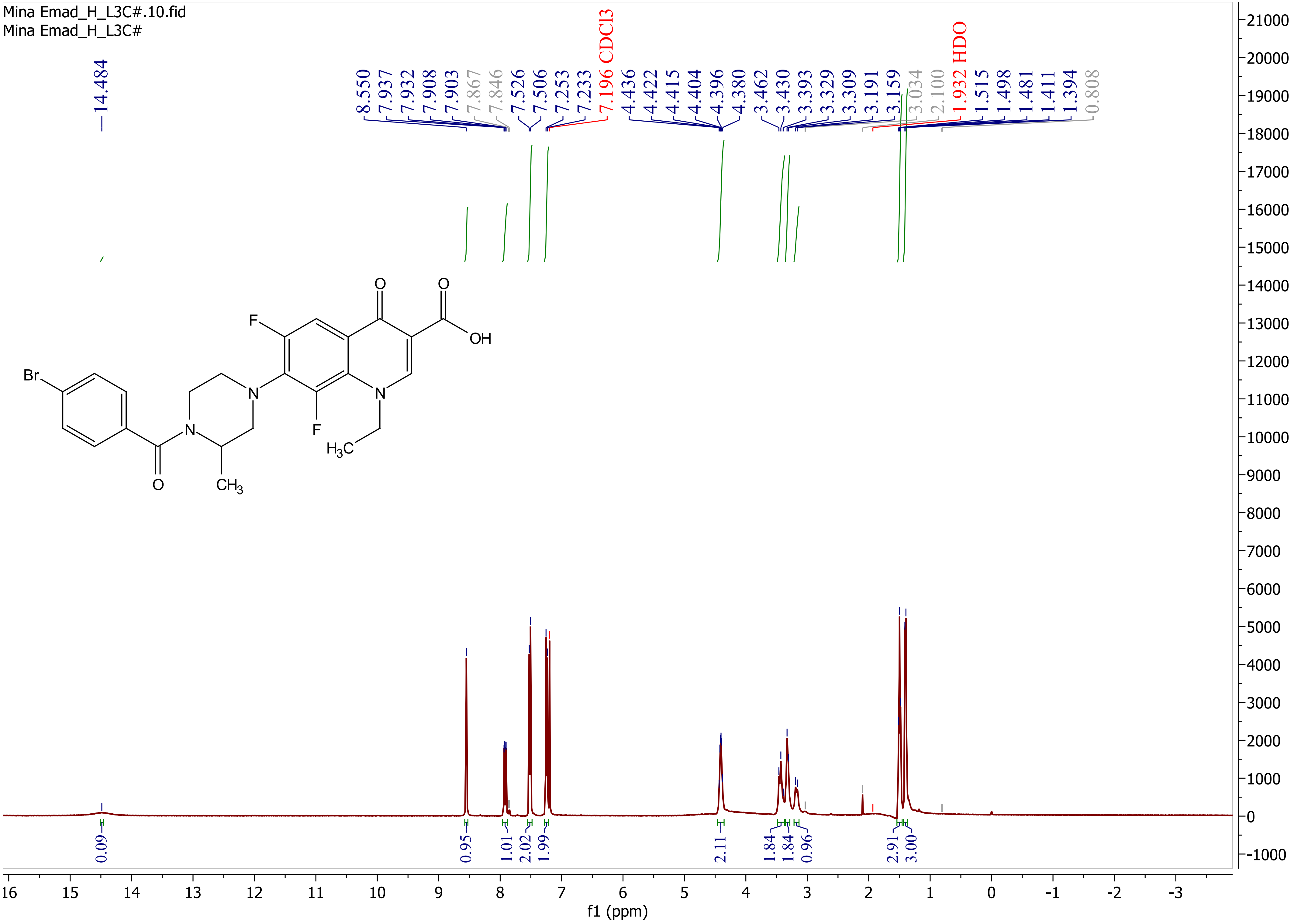

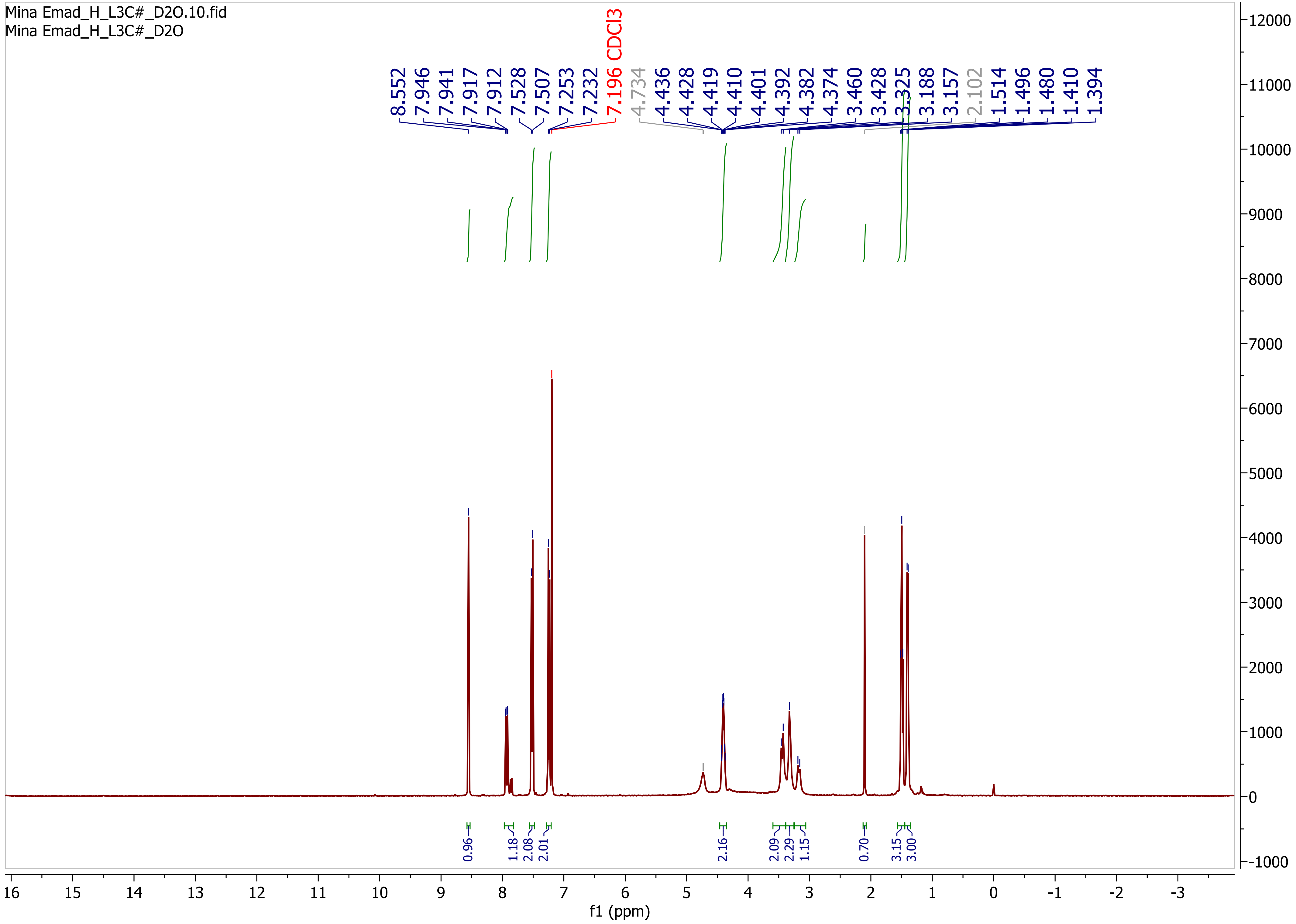

Mina Emad_H_L3C#_D2O.10.fid
Mina Emad_H_L3C#_D2O
8.552
7.946
7.941
7.917
7.912
7.528
7.507
7.253
7.232
7.196 CDCl3
4.734
4.436
4.428
4.419
4.410
4.401
4.392
4.382
4.374
3.460
3.428
3.325
3.188
3.157
2.102
1.514
1.496
1.480
1.410
1.394
0.96
1.18
2.08
2.01
2.16
2.09
2.29
1.15
0.70
3.15
3.00
f1 (ppm)

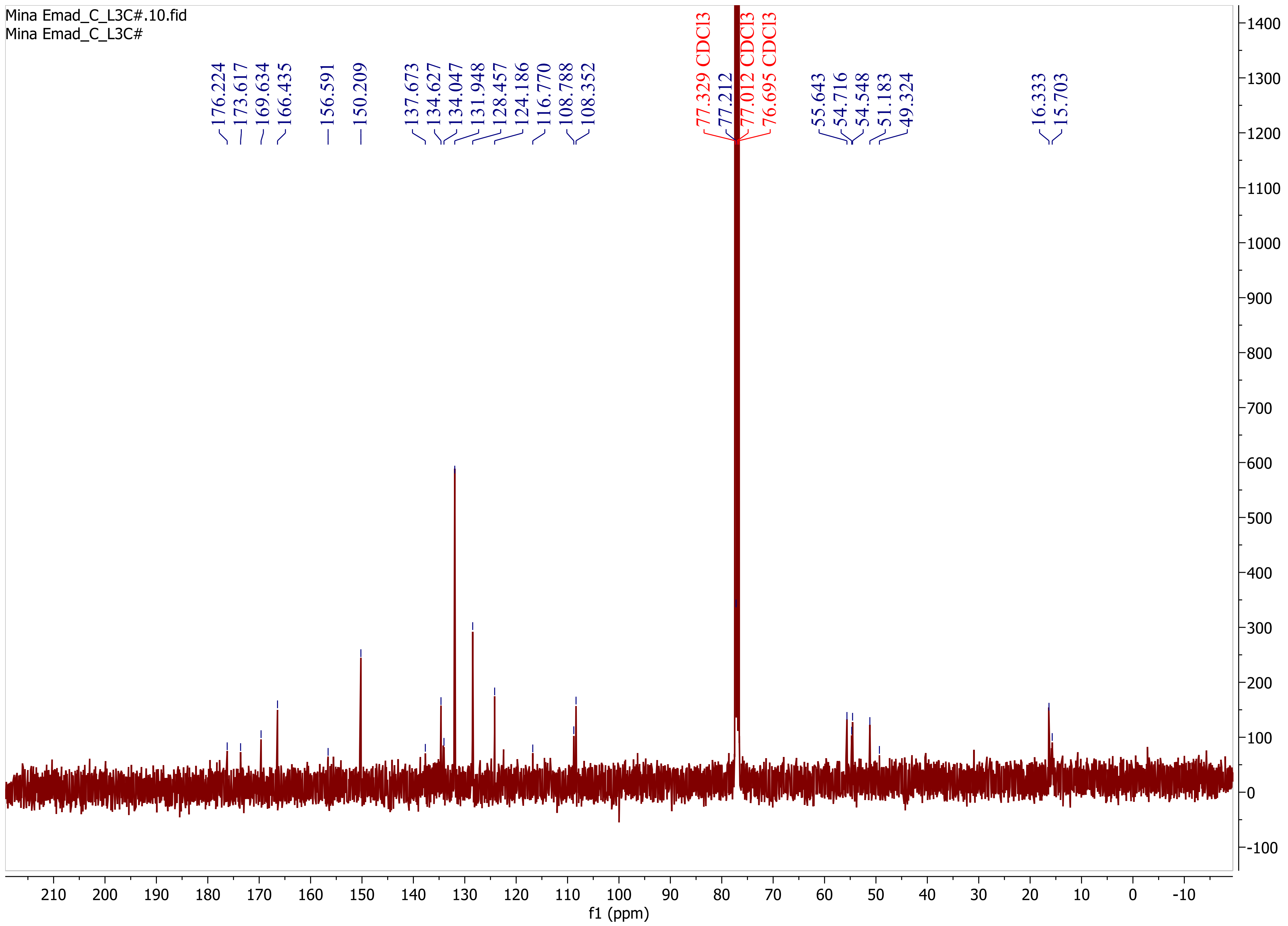
Mina Emad_C_L3C#.10.fid
Mina Emad_C_L3C#
176.224
173.617
169.634
166.435
156.591
150.209
137.673
134.627
134.047
131.948
128.457
124.186
116.770
108.788
108.352
77.329 CDCl3
77.212
77.012 CDCl3
76.695 CDCl3
55.643
54.716
54.548
51.183
49.324
16.333
15.703
f1 (ppm)

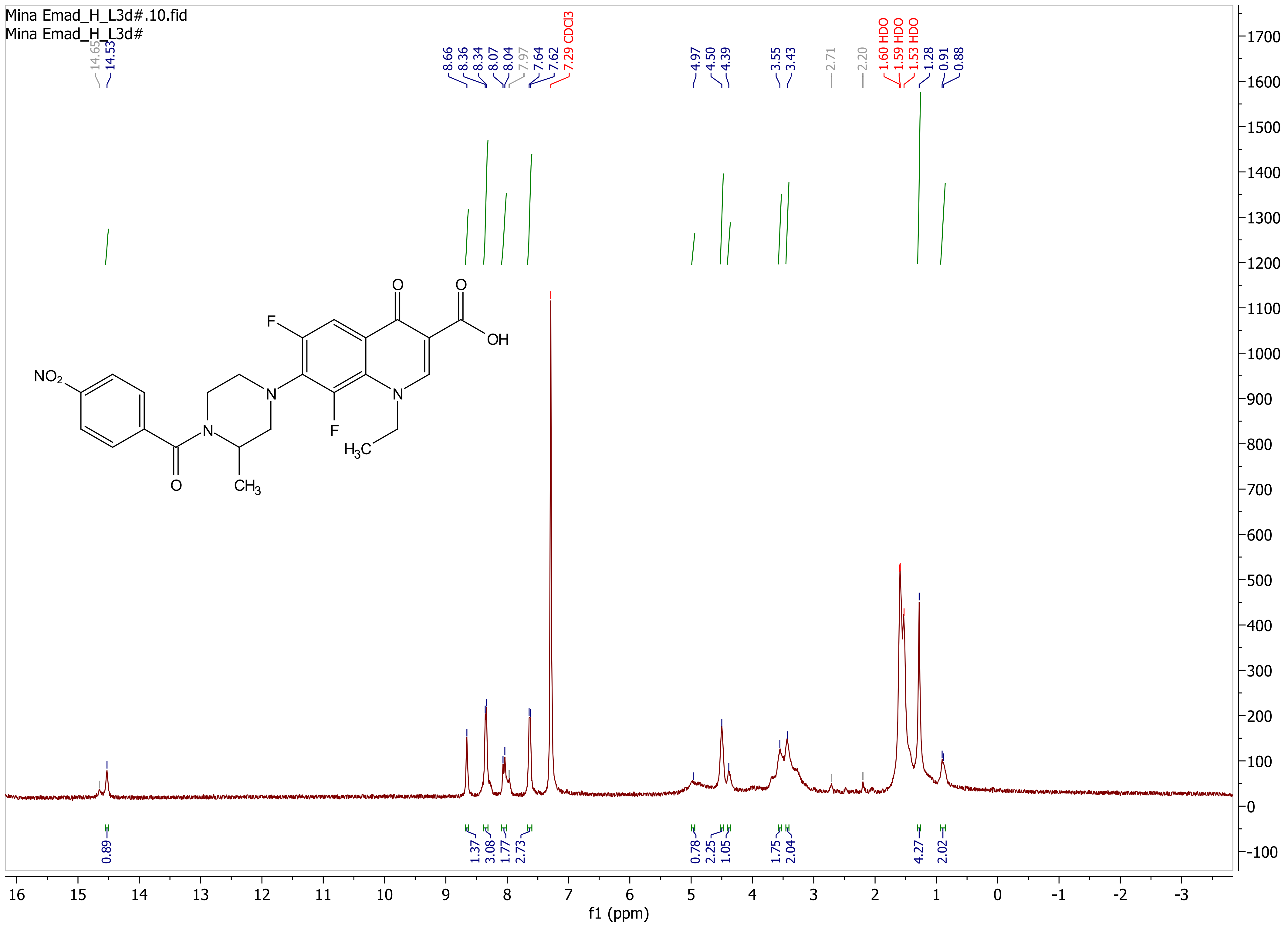

Mina Emad_H_L3d#.10.fid
Mina Emad_H_L3d#
14.65
14.53
8.66
8.36
8.34
8.07
8.04
7.97
7.64
7.62
7.29 CDCl3
4.97
4.50
4.39
3.55
3.43
2.71
2.20
1.60 HDO
1.59 HDO
1.53 HDO
1.28
0.91
0.88
0.89
1.37
3.08
1.77
2.73
0.78
2.25
1.05
1.75
2.04
4.27
2.02
f1 (ppm)
F
F
O
O
OH
N
N
N
NO2
O
CH3
H3C

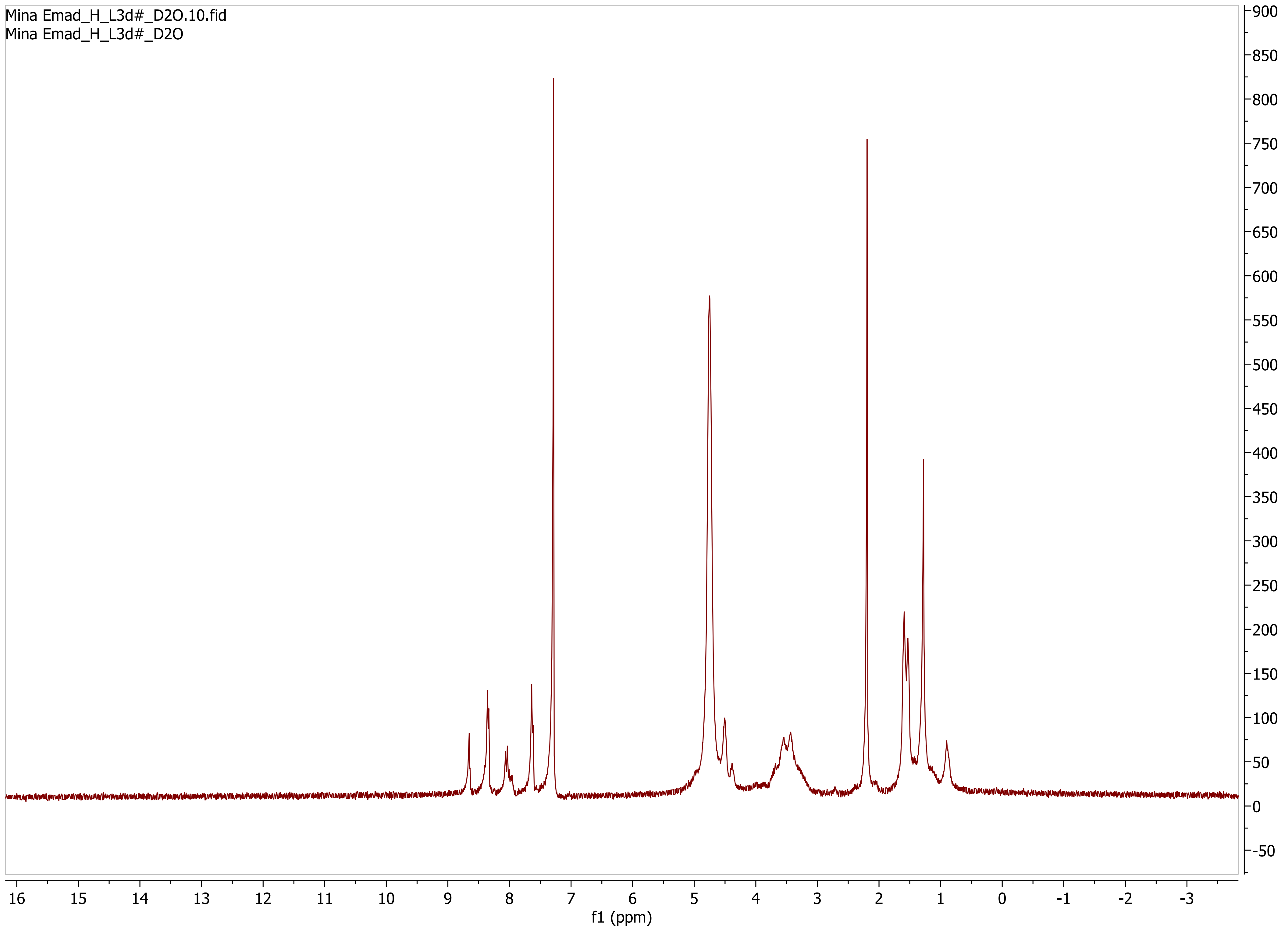

Mina Emad_H_L3d#_D2O.10.fid
Mina Emad_H_L3d#_D2O
900
850
800
750
700
650
600
550
500
450
400
350
300
250
200
150
100
50
0
-50
16
15
14
13
12
11
10
9
8
7
6
5
4
3
2
1
0
-1
-2
-3
f1 (ppm)

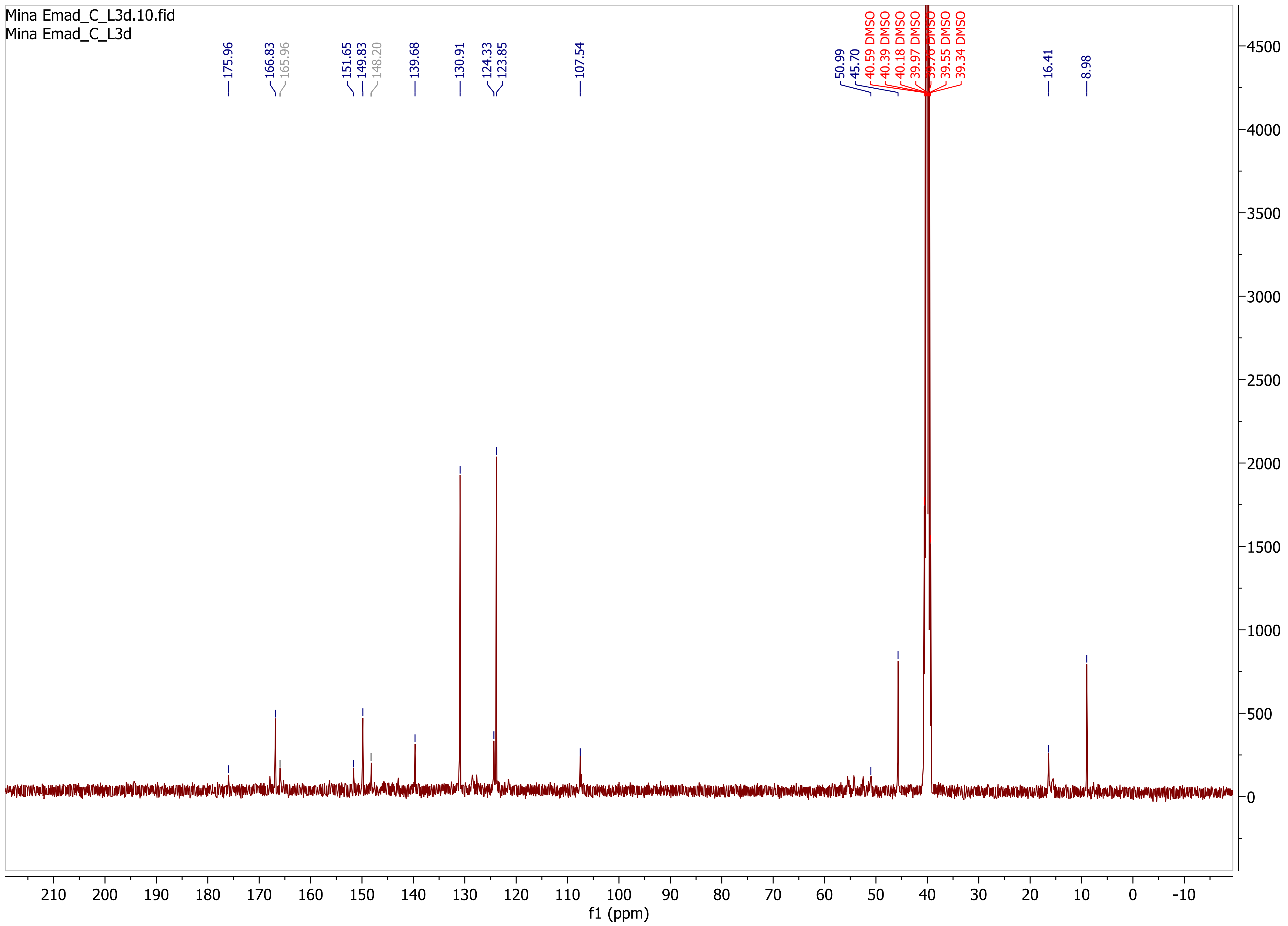
Mina Emad_C_L3d.10.fid
Mina Emad_C_L3d
175.96
166.83
165.96
151.65
149.83
148.20
139.68
130.91
124.33
123.85
107.54
50.99
45.70
40.59 DMSO
40.39 DMSO
40.18 DMSO
39.97 DMSO
39.76 DMSO
39.55 DMSO
39.34 DMSO
16.41
8.98
f1 (ppm)
